# Catalytic Enantioselective Access to Dihydroquinoxalinones via Formal α‐Halo Acyl Halide Synthon in One Pot

**DOI:** 10.1002/anie.202110173

**Published:** 2021-09-30

**Authors:** Chiara Volpe, Sara Meninno, Carlo Crescenzi, Michele Mancinelli, Andrea Mazzanti, Alessandra Lattanzi

**Affiliations:** ^1^ Dipartimento di Chimica e Biologia “A. Zambelli” Università di Salerno Via Giovanni Paolo II 132-84084 Fisciano Italy; ^2^ Dipartimento di Farmacia Università di Salerno Via Giovanni Paolo II 132-84084 Fisciano Italy; ^3^ Dipartimento di Chimica Industriale Università di Bologna Viale Risorgimento 4-40136 Bologna Italy

**Keywords:** asymmetric catalysis, domino ring-opening cyclization, epoxidation, heterocycles, one-pot reactions

## Abstract

An enantioselective one‐pot catalytic strategy to dihydroquinoxalinones, featuring novel 1‐phenylsulfonyl‐1‐cyano enantioenriched epoxides as masked α‐halo acyl halide synthons, followed by a domino ring‐opening cyclization (DROC), is documented. A popular quinine‐derived urea served as the catalyst in two out of the three steps performed in the same solvent using commercially available aldehydes, (phenylsulfonyl)acetonitrile, cumyl hydroperoxide and 1,2‐phenylendiamines. Medicinally relevant 3‐aryl/alkyl‐substituted heterocycles are isolated in generally good to high overall yield and high enantioselectivity (up to 99 % *ee*). A rare example of excellent reusability of an organocatalyst at higher scale, subjected to oxidative conditions, is demonstrated. Mechanistically, labile α‐ketosulfone has been detected as the intermediate involved in the DROC process. Theoretical calculations on the key epoxidation step rationalize the observed stereocontrol, highlighting the important role played by the sulfone group.

## Graphical abstract

A one‐pot catalytic and scalable Knoevenagel/asymmetric epoxidation/domino ring‐opening cyclization (DROC) strategy towards medicinally significant dihydroquinoxalinones, featuring 1‐phenylsulfonyl‐1‐cyano epoxides as new masked α‐halo acyl halide synthons, is reported. Two out of the three steps are catalysed by highly robust, recyclable and easily available quinine‐derived urea.

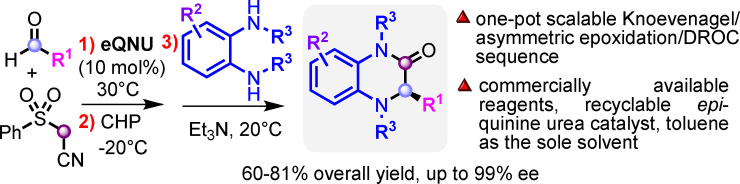

## Introduction

Nowadays the development of one‐pot routes to heterocyclic compounds represents a recurrent and convenient strategy, in line with the principles of green chemistry and the economies of organic synthesis.^[1]^ Optically active *N*‐heterocycles are of particular interest, achieving around 60 % of FDA approved drugs, with a relevant portion occupied by simple six‐membered rings and their fused analogues.^[2]^ In the last decades, the one‐pot organocatalytic approach proved to be an excellent and increasingly applied tool to efficiently prepare important drugs and natural products under mild conditions at reduced costs, time and environmental impact.^[3]^ The remarkable examples reported by the groups of Hayashi,^[4a]^ Zhu,^[4b]^ Aggarwal,^[4c]^ Paião,^[4d]^ Zhang,^[4e]^ Masson,^[4f]^ Dixon^[4g]^ showed additional key‐features of this tool, in securing wide molecular diversity and rapid access to libraries of compounds necessary for bioassay and drug discovery, thus resulting particularly appealing from an industrial perspective.^[4]^

**Scheme 1:**
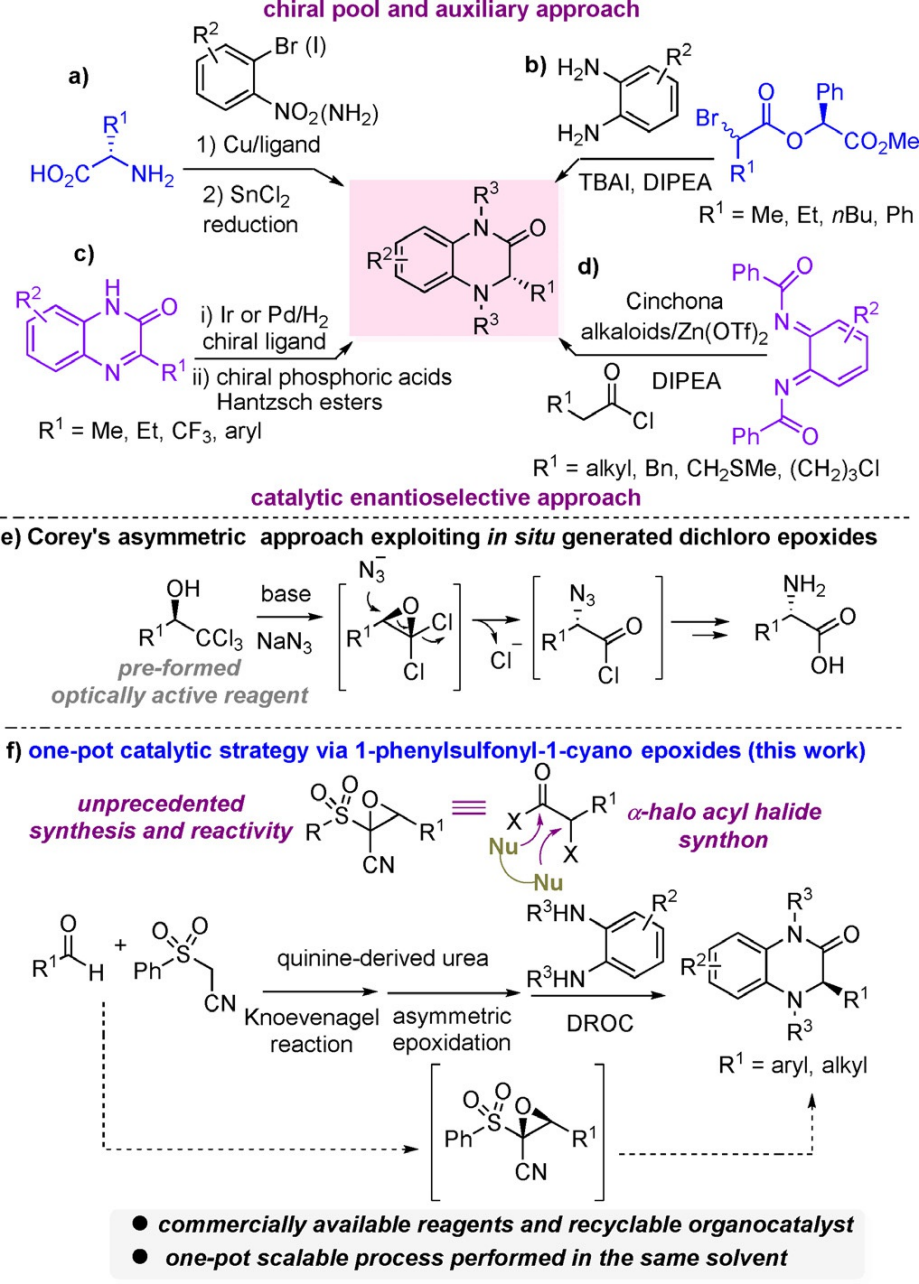
Comparison of the state‐of‐the‐art and this work.

Chiral dihydroquinoxalinones are recognized as privileged pharmacophores of broad interest in medicinal chemistry, found in a variety of bioactive compounds and pharmaceuticals^[5]^ displaying anti‐HIV,^[6]^ antineoplastic,^[7]^ anti‐inflammatory^[8]^ activities, fungicidal properties^[9]^ and used in the control of cardiovascular disease.^[10]^ Moreover, they can serve as precursors of enantioenriched tetrahydroquinoxalines, which are another class of heterocycles endowed with several biological activities.^[11]^ Expectedly, significant efforts have been devoted to preparing optically active dihydroquinoxalinones, through the development of stepwise metal‐catalysed and organocatalysed strategies. The most representative and distinct approaches can be grouped in chiral pool or auxiliary based methods (a,b) and catalytic enantioselective processes (c,d) (Scheme 1).

The first ones were based on natural α‐amino acids as reagents used in Cu^I^‐catalysed coupling with *ortho*‐nitro, or *ortho*‐amino aryl halides followed by reduction and cyclization (Scheme 1 a).^[12]^ (*S*)‐Mandelate α‐bromoesters were also found to be competent substrates for a dynamic kinetic resolution in nucleophilic substitution with 1,2‐phenylendiamines, followed by cyclization (Scheme 1 b).^[13]^ Both approaches, although based on cheap chiral sources, somewhat suffered of substrate‐dependent applicability or partial racemization.^[14]^ More recently, Ir‐ and Pd‐catalysed hydrogenation of quinoxalinones enabled to achieve consistently high level of asymmetric induction (Scheme 1 c,i).^[15]^ The process was also successfully expanded, employing chiral phosphoric acids/Hantzsch ester systems (Scheme 1 c,ii).^[16]^ Leckta and co‐workers developed a Cinchona alkaloids/Lewis acid promoted hetero‐ Diels–Alder reaction of ketene enolates and *ortho*‐benzoquinone diimides to construct the heterocycles in excellent enantioselectivity (Scheme 1 d).^[17]^

Epoxides are undoubtedly recognized as highly versatile intermediates in organic synthesis^[18]^ and those bearing two identical electron‐withdrawing groups with good leaving abilities (EWG=Cl, CN, SO_2_R) positioned at the same carbon atom, behave as masked α‐halo acyl halide synthons.^[19]^

This reactivity has been exploited to obtain racemic α‐substituted carboxylic acid derivatives from different epoxide sources. Corey pioneered an interesting asymmetric variant, involving ring‐opening of highly reactive *gem*‐dichloro epoxides, in situ generated from chiral non racemic trichloromethyl carbinols as the starting reagent (Scheme 1 e).^[20]^ Despite the undisputed value of the methodologies illustrated in Scheme 1, multi‐step preparation of reagents and heterocyclic precursors somewhat limited the practicality and pot‐economy of the catalytic variants. Therefore, a one‐pot process to dihydroquinoxalinones, starting from commercially available compounds and a reusable catalyst, would significantly fill a gap in addressing these issues. Building on our research interest in the development of stereocontrolled one‐pot methodologies to prepare heterocyclic compounds,^[21]^ we envisaged the possibility of setting up a new streamlined catalytic asymmetric strategy based on Knoevenagel reaction/asymmetric epoxidation/domino ring‐opening cyclization, trusting in the ability of a chiral non racemic bifunctional organocatalyst to promote the first two steps in a stereoselective manner (Scheme 1 f). 1‐Phenylsulfonyl‐1‐cyano epoxides were targeted as new potential masked α‐halo acyl halide synthons,^[22]^ although either the epoxidation of 1‐phenylsulfonyl‐1‐cyano alkenes and the reactivity of 1‐phenylsulfonyl‐1‐cyano epoxides were unprecedented. This choice has been guided by the following considerations: i) the suitability of the Knoevenagel reaction to access (*E*)‐configured 1‐phenylsulfonyl‐1‐cyano alkenes from aldehydes under mild reaction conditions,^[23]^ ii) the known strong hydrogen‐bonding acceptor nature of the sulfone group,^[24]^ likely useful to act as chemical handle for the stereocontrol in the nucleophilic epoxidation. Herein, we illustrate the results of this study to straightforwardly access optically active dihydroquinoxalinones, exploiting a readily available quinine‐derived urea as reusable catalyst, commercial aldehydes, (phenylsulfonyl)acetonitrile, cumyl hydroperoxide (CHP) and 1,2‐phenylendiamines. The DFT study confirmed the crucial role played by the sulfone group in directing the stereoselectivity of the epoxidation step.

## Results and Discussion

We commenced our study on the enantioselective epoxidation of model alkene **1 a**, employing *tert*‐butyl hydroperoxide (TBHP) as the oxidant and readily available amine‐derived thioureas, urea and squaramide in toluene at room temperature (Table 1).

**Table 1:** Optimization of the asymmetric epoxidation reaction on alkene **1 a**.^[a]^ 
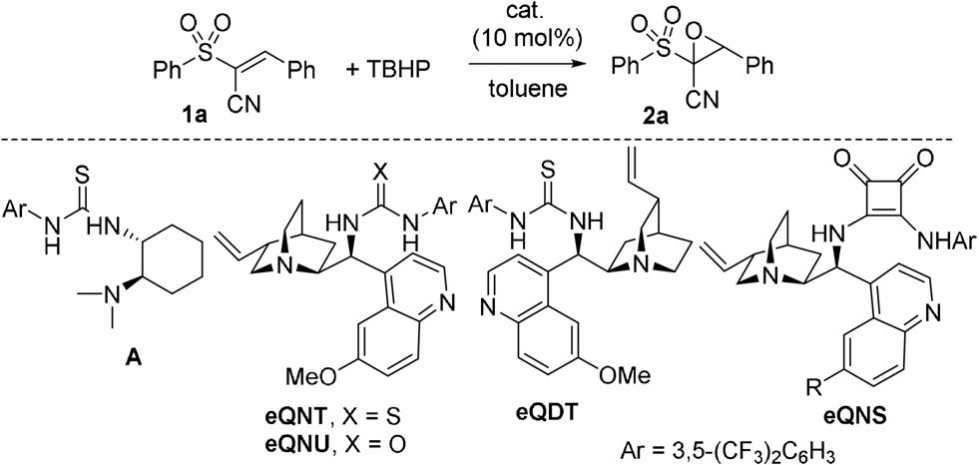

Entry	Cat.	*T* [°C]	Yield [%]^[b]^(*t* [h])	*ee* [%]^[c]^
1	**A**	25	62 (4)	41
2	**eQNT**	25	75 (19)	−74
3	**eQDT**	25	49 (19)	38
4	**eQNS**	25	95 (7)	−64
5	**eQNU**	25	90 (3)	−70
6^[d]^	**eQNU**	25	90 (1)	−86
7^[d,e]^	**eQNU**	25	95 (4)	−91
8^[d,e]^	**eQNU**	−20	94 (14)	−93
9^[d,f]^	**eQNU**	−30	91 (15)	−92
10^[d,g]^	**eQNU**	−20	90 (14)	−92
11^[d,g]^	**eQNT**	−20	87 (14)	−91
12^[d,h]^	**eQNU**	−20	78 (40)	−89

[a] **1 a** (0.10 mmol), TBHP (0.12 mmol), catalyst (0.010 mmol) in anhydrous toluene (0.5 mL). [b] Yields are of isolated product, dr >95:5. [c] HPLC analysis on a chiral stationary phase. [d] CHP used as oxidant. [e] Performed at *C*=0.02 M [**1 a**]. [f] Performed at *C*=0.05 M [**1 a**]. [g] Performed at *C*=0.05 M [**1 a**] with 5 mol % of catalyst. [h] Performed at *C*=0.05 M [**1 a**] with 2 mol % of catalyst.

**Scheme 2:**
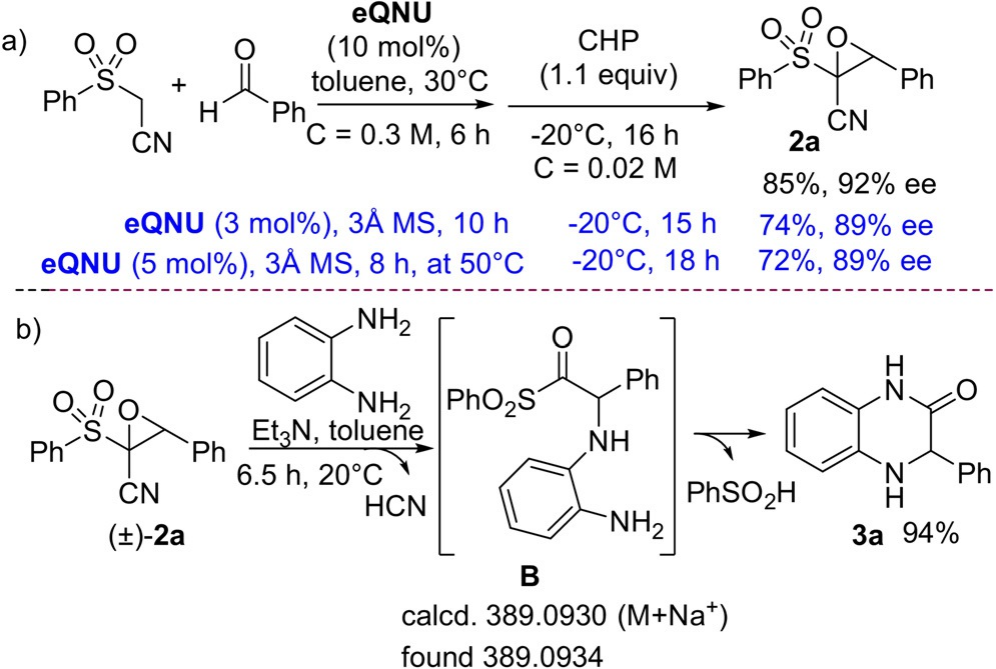
a) One‐pot Knoevenagel/asymmetric epoxidation catalysed by **eQNU**. b) DROC of racemic epoxide.

When using 10 mol % of thiourea amine **A**, we were pleased to isolate *trans*‐epoxide **2 a** in 62 % yield, with complete diastereoselectivity and 41 % *ee* (entry 1). Interestingly, *epi*‐quinine derived thiourea (**eQNT**) provided the epoxide with 74 % *ee* (entry 2). However, *epi*‐quinidine derived thiourea (**eQDT**) proved to be less effective, affording the opposite enantiomer of **2 a** with 38 % *ee* (entry 3). *epi*‐Quinine derived squaramide (**eQNS**) and urea (**eQNU**) performed more actively, furnishing the epoxide, after short reaction times, in high yields, with 64 % *ee* and 70 % *ee*, respectively (entries 4 and 5). **eQNU** was then chosen as the catalyst for further optimization in toluene as the solvent (see Table S1), using CHP as the oxidant. We were delighted to observe a rapid formation of the epoxide, isolated in 90 % yield and 86 % *ee* (entry 6). Dilution of the reaction mixture was beneficial, leading to the product in 95 % yield and 91 % *ee* (entry 7), with further improvement to 93 % *ee*, observed when working at −20 °C (entry 8). However, no additional enhancement was detected operating at −30 °C (entry 9). The epoxidations performed at lower catalyst loadings (down to 2 mol %), proceeded slowly and with a slightly decreased level of enantioselectivity (entries 10 and 12). We proved that, under the same conditions, **eQNU** was the catalyst of choice with respect to **eQNT** (entries 10 and 11). At this point, sequential Knoevenagel reaction/epoxidation was performed using **eQNU** at 10 mol % under the most effective conditions achieved in Table 1 for the epoxidation (entry 8), using benzaldehyde and (phenylsulfonyl)acetonitrile (Scheme 2). To our delight, **eQNU** efficiently catalyzed the Knoevenagel reaction in reasonable time and *trans*‐epoxide **2 a** was recovered in 85 % isolated yield and 92 % *ee*. The one‐pot process performed at 3 or 5 mol % catalyst loadings, in the presence of molecular sieves to foster the Knoevenagel step either at 30 °C and 50 °C, proceeded in a less effective manner.

Key to the success of the one‐pot sequence from aldehydes to dihydroquinoxalinones was to attest the feasibility of the DROC process. Accordingly, the investigation was carried on racemic **2 a** using *ortho*‐phenylendiamine and triethyl amine as acid scavenger in toluene at room temperature (Scheme 2).

We were delighted to observe a clean conversion to the expected dihydroquinoxalinone **3 a**, which was isolated in 94 % yield. Reaction progress profile monitored by ^1^H‐NMR analysis showed the exclusive presence of compound **3 a**, supporting fast ring‐opening of the epoxide and intramolecular capture of an elusive acyl intermediate. Nevertheless, we were able to identify the involvement of α‐ketosulfone **B** species by high resolution mass spectrometry analysis of the reaction mixture (see Table S3 and Figure S1). α‐Ketosulfones have been previously reported to be labile intermediates, prone to react with nucleophiles.^[25]^ Results reported in Table 1 and Scheme 2 favorably suggested the viability of a sequential one‐pot process, starting from commercially available aldehydes, (phenylsulfonyl)acetonitrile, CHP and *ortho*‐phenylendiamines, to be conveniently conducted in toluene as the sole solvent.

With the optimized conditions in hand, the scope and limitations of the one‐pot approach were firstly studied using *ortho*‐phenylendiamine (Table 2). Model dihydroquinoxalinone (*R*)‐**3 a** was isolated in 76 % and 89 % *ee*, proving that clean and high conversions occurred in all steps, with enantioselectivity essentially maintained, as expected for a S_N_2 ring‐opening reaction. Aromatic aldehydes, bearing electron‐donating or electron‐withdrawing substituents at different positions (*para*, *meta* and *ortho*), were well tolerated, including sterically encumbered 1‐naphthyl unit, leading to the heterocycles (**3 b**–**k**) in high to excellent overall yields (61–81 %). High enantioselectivity was observed (86–96 % *ee*), with the exception of the *ortho*‐substituted compounds **3 g** and **3 k**, isolated, respectively with 65 % and 62 % *ee*, likely ascribed to steric hindrance. To introduce an alkenyl group at the chiral center of the heterocycle, (*E*)‐cinnamaldehyde was checked as the starting reagent under usual conditions. Although the alkene was formed, this challenging substrate proved to be inert under the epoxidation conditions, even after prolonged reaction time. The absolute configuration of the stereocenter for compounds **3** was assigned as (*R*) upon comparison with optical rotations reported in the literature for known products **3 a**, **3 b,d**, **3 r,s** and the configuration of the other heterocycles was assigned in analogy.

**Table 2:** Scope of the enantioselective one‐pot catalytic synthesis of dihydroquinoxalinones.^[a–d]^ 
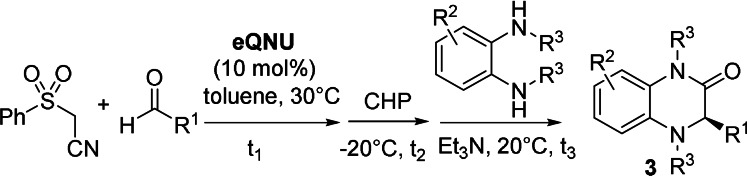

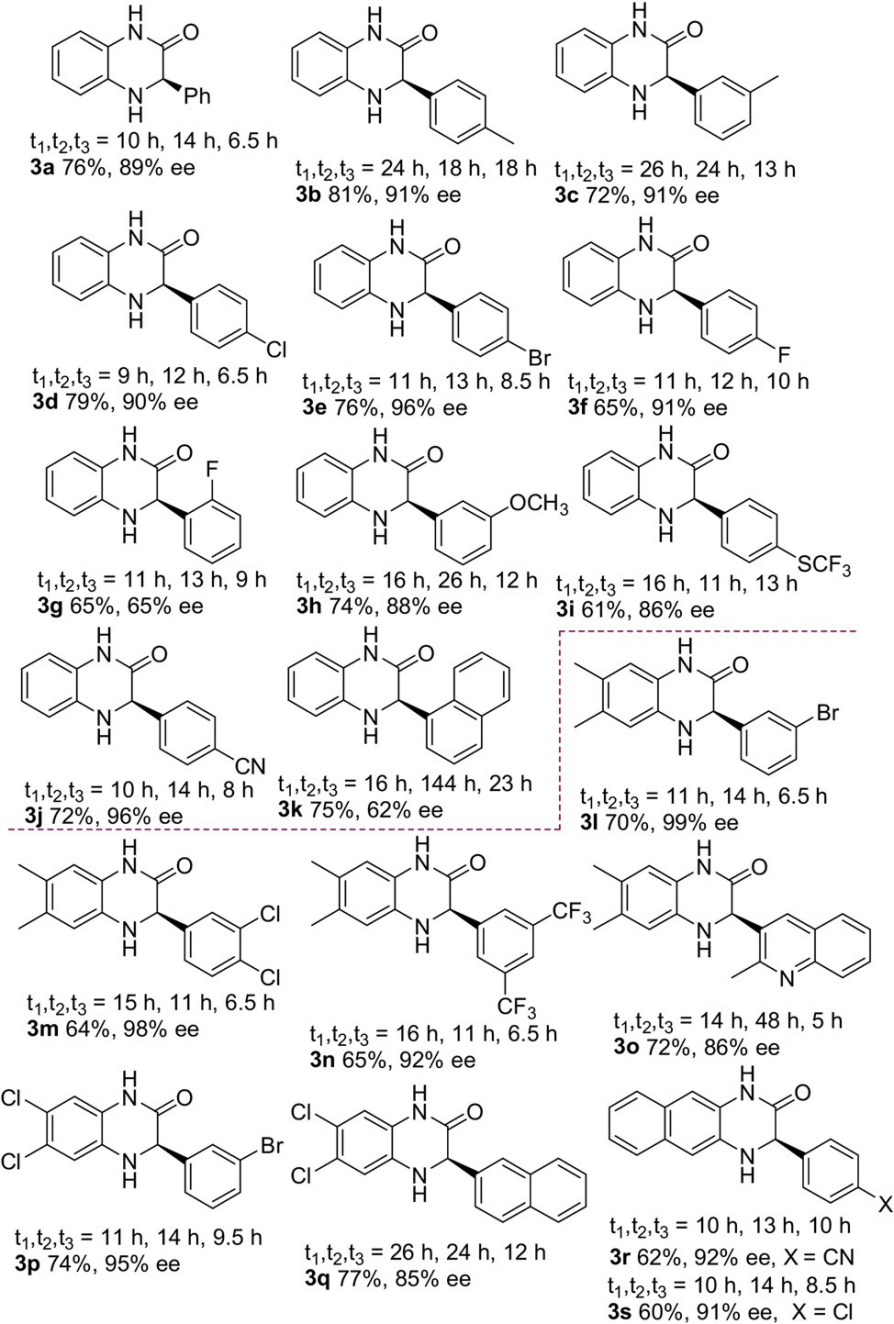
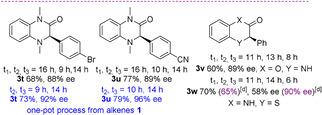

[a] i) Knoevenagel step: (phenylsulfonyl)acetonitrile (0.12 mmol), aldehyde (0.12 mmol), **eQNU** (0.012 mmol) in anhydrous toluene (0.4 mL). ii) Epoxidation step: the reaction mixture is diluted with toluene (5.6 mL) placed at −20 °C and CHP (1.1 equiv, 0.132 mmol) added. DROC step: iii) *o*‐phenylendiamines or 1,2‐amino(thio)phenol (0.132 mmol) and Et_3_N (0.24 mmol) added at 20 °C. [b] Yields are of isolated product. [c] HPLC analysis on a chiral stationary phase. [d] Diisopropylethyl amine was used in the DROC.

Then, commercially available substituted *ortho*‐phenylendiamines^[26]^ were coupled with either mono‐, di‐substituted, 2‐naphthyl and heteroaromatic aldehydes providing diversely substituted dihydroquinoxalinones (**3 l**–**s**) with high yields (60–77 %) and high to excellent enantioselectivity (85–99 % *ee*). The feasibility of DROC process with more sterically hindered *N*‐methyl *ortho*‐phenylendiamine was next examined, with a view to directly install typical *N*‐methyl substitution found in some pharmaceutically active derivatives, thus avoiding a following sensitive *N*‐alkylation step.^[27]^ Gratifyingly, the corresponding *N*‐methyl derived dihydroquinoxalinones **3 t** and **3 u** were isolated in 68 %, 77 % yield and with 88 % *ee*, 89 % *ee*, respectively. When the one‐pot process was carried out from the corresponding alkenes, heterocycles **3 t** and **3 u** were isolated with 92 % *ee* and 96 % *ee*, showing that a slight erosion of the enantioselectivity occurred when starting from aldehydes. Preliminary investigations were then carried out using *ortho*‐amino phenol and *ortho*‐amino thiophenol with benzaldehyde as the reagent. Pleasingly, dihydrobenzoxazinone (*R*)‐**3 v** was obtained in 60 % overall yield and 89 % *ee*.[^16b^, ^16d^] Even more relevant, challenging dihydrobenzothiazinone **3 w** was selectively recovered in 70 % yield, although with 58 % *ee*. However, racemization was successfully suppressed by using more sterically demanding Hünig's base in the DROC step (see Table S2). The enantioselectivity increased to 90 % *ee*, which represents the highest value reached so far for compound **3 w**.^[13a]^ Hence, the mild and tunable reaction conditions employed in the one‐pot protocol, appear to be of wider applicability to access different families of related enantioenriched heterocycles.

Direct synthesis of compounds **3** from aliphatic aldehydes is hampered by the occurrence of competitive self‐condensation/oligomerization pathways in the Knoevenagel reaction.^[23]^

**Scheme 3:**
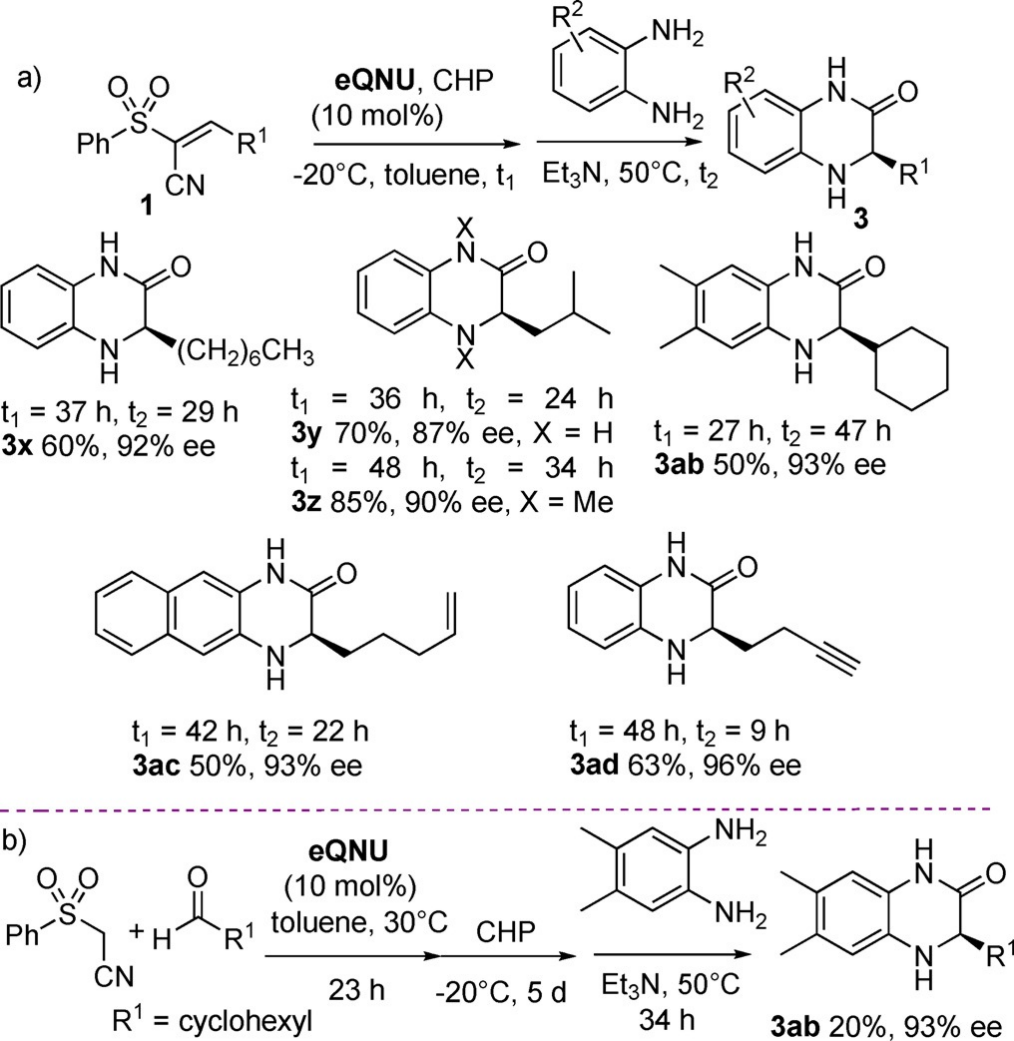
One‐pot asymmetric sequence to 3‐alkyl‐substituted dihydroquinoxalinones from a) aliphatic alkenes and from b) aldehyde and (phenylsulfonyl)acetonitrile.

Consequently, the viability of a catalytic one‐pot asymmetric synthesis of 3‐alkyl‐substituted dihydroquinoxalinones was investigated starting from alkenes **1** and different *ortho*‐phenylendiamines (Scheme 3 a). This strategy proved to be versatile, since it could be extended to prepare linear, sterically encumbered alkyl and cycloalkyl‐substituted heterocycles **3 x**–**ab** in good to high yields (50–85 %) and high enantioselectivity (87–93 % *ee*).^[28]^ Alkyl moieties, bearing carbon‐carbon double and triple bonds were also installed, offering opportunities for hitherto unachievable post‐functionalizations onto the alkyl chain of compounds **3**. In both examples, the heterocycles **3 ac**–**3 ad** were isolated in good yields (50–63 %) and excellent enantiocontrol (93–96 % *ee*).

Finally, the one‐pot process was attempted under working conditions, starting directly from cyclohexanecarboxaldehyde and (phenylsulfonyl)acetonitrile (Scheme 3 b). Predictably, they sluggishly reacted in the Knoevenagel step, observing a low conversion to the alkene. Compound **3 ab** was isolated in 20 % overall yield, although the enantioselectivity was maintained, showing that the organocatalyst was able to work effectively under more complex reaction conditions.

**Scheme 4:**
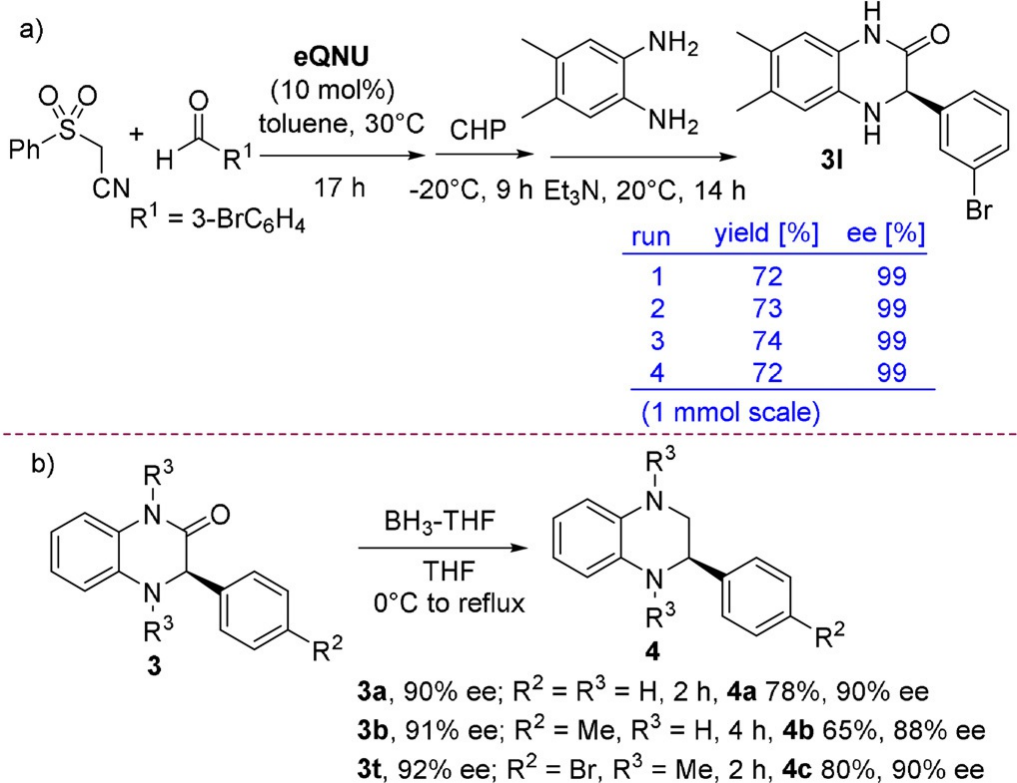
a) 1 mmol scale‐up one‐pot synthesis of dihydroquinoxalinone **3 l** with recyclability of **eQNU**. b) Reduction of compounds **3** to tetrahydroquinoxalines.

To demonstrate the practicality and scalability of this process as well as the recyclability of **eQNU**, the synthesis of compound **3 l** was performed at 1 mmol scale of 3‐bromobenzaldehyde and (phenylsulfonyl)acetonitrile for different runs (Scheme 4 a). To our delight, heterocycle **3 l** was always isolated in high yields and 99 % *ee*, highlighting complete recovering of **eQNU** after chromatography and a remarkable robustness of this organocatalyst toward repetitive oxidative conditions up to four runs. It is interesting to note that **eQNU** and similar Cinchona alkaloids derived organocatalysts have been recently classified according to their E‐factor amongst the greenest organocatalysts to prepare.^[29]^

Despite the importance of chiral non racemic tetrahydroquinoxalines as bioactive compounds,^[11]^ to the best of our knowledge, their synthesis via reduction of enantioenriched 3‐aryl‐substituted dihydroquinoxalinones has not been reported. Indeed, the most recurrent approach involves metal‐catalyzed enantioselective hydrogenation or transfer hydrogenation of quinoxalines.[^16a^, ^30^]

To perform the reduction, enantioenriched NH‐free and *N*‐Me‐substituted compounds **3 a**, **3 b** and **3 t**, were treated with BH_3_‐THF complex in THF under reflux (Scheme 4 b).^[17b]^ This reductive procedure proved to be synthetically useful, delivering the corresponding tetrahydroquinoxalines **4** in good yields and *ee* values practically maintained.

**Figure 1:**
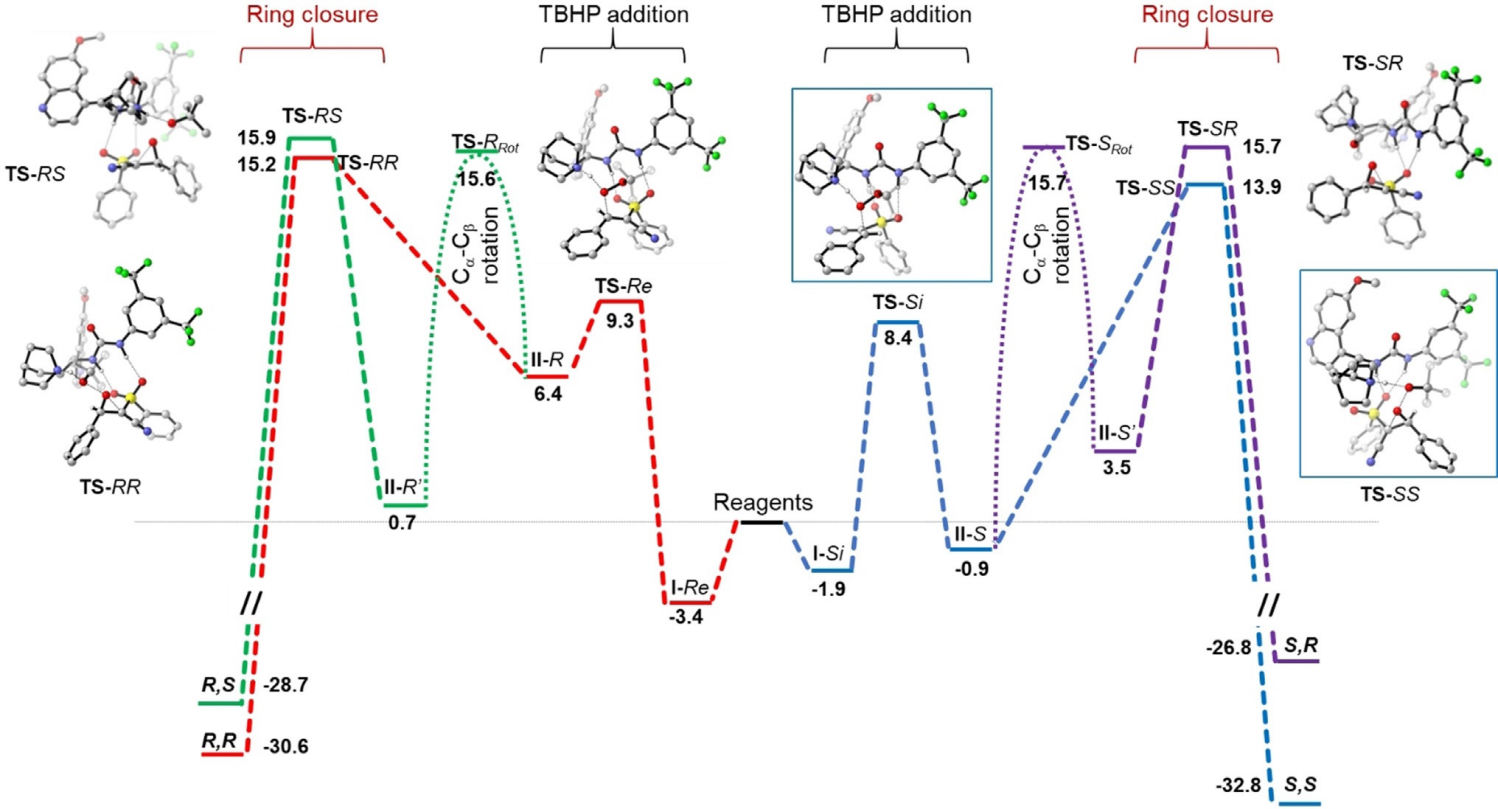
Full reaction pathway for the asymmetric epoxidation of alkene **1 a** to epoxide **2 a** with TBHP. Free energies are relative to reagents, in kcal mol^−1^ at the IEF‐PCM B3LYP‐GD3(BJ)/6–311+G(2d,p)//B3LYP/6‐31G(d) level of theory. Structures in blue boxes are the two TSs for the formation of the (*S*,*S*) stereoisomer.

To shed light on the experimental stereocontrol achieved in the epoxidation step, a theoretical study was performed by DFT calculations, using **2 a** as model compound. To reduce the conformational space, CHP was replaced by TBHP and the vinyl group of **eQNU** was omitted. Optimization of the ground states (GS) and transition states (TS) geometries was obtained at the B3LYP‐GD3(BJ)/6–311+g(2d,p)//B3LYP/6‐31G(d) level of theory, including toluene with the IEF‐PCM^[31]^ solvent approach (see the Supporting Information for further details). The nucleophilic epoxidation pathway was modelled considering three steps: i) formation of the electrophilic complex from **eQNU** and alkene, ii) oxa‐Michael addition of the alkyl hydroperoxide to yield the peroxyenolate complexed with protonated catalyst; iii) ring closure by intramolecular S_N_2 reaction with release of *tert*‐butanol (see Figure 1 for the whole catalytic cycle).

Being toluene the reaction solvent, the formation of the reactive complex between catalyst and the alkene should be driven mainly by hydrogen bonding between the urea group with the sulfone moiety,^[24]^ leaving the quinuclidinic basic nitrogen free to assist the addition of TBHP within the subsequent reaction step. GS geometries of the complex were built following this bias and by orienting the complexed alkene in such a way to expose the two enantiotopic faces of the β‐carbon (see Figure S2) to the nucleophilic attack. A conformational analysis was performed to search for the best catalyst geometry, being the lowest energy conformation found in agreement with a similar case.^[32]^ The addition of TBHP to the β‐carbon was modelled assuming assistance of the quinuclidinic lone pair nitrogen to boost TBHP nucleophilicity.^[33]^ Two geometries were optimized for the addition to both faces of the β‐carbon, with activation energies of 8.4 kcal mol^−1^ (**TS**‐*Si*) and 9.3 kcal mol^−1^ (**TS**‐*Re*, see Figure S3). In both cases, the TS involves the weakening of the O−H bond by the lone pair on nitrogen, and the simultaneous formation of the C−O bond. After the formation of the C−O bond, two intermediates (**II**‐*Si* and **II**‐*Re*) were reached by shift of the hydrogen onto the quinuclidinic nitrogen (see Figure S4). Now the reaction can fork towards different directions: i) ring closure by intramolecular S_N_2 reaction straightforwardly yields the (*S*,*S*) and (*R*,*R*) epoxides by release of *tert*‐butanol as the leaving group; ii) rotation around the C_α_‐C_β_ bond yields two additional intermediates (**II**‐*S*′ and ***II***‐*R*′) with the cyano and the phenyl groups at opposite side of the original C−C double bond. The subsequent ring closure step eventually leads to the (*S*,*R*) and (*R*,*S*) stereoisomers.

The two TS geometries for ring closure to (*S*,*S*) and (*R*,*R*) epoxides from **II**‐*Si* and **II**‐*Re* were built considering the assistance of the acidic NH^+^ site in the protonation of the *tert*‐butoxide to *tert*‐butanol leaving group. Full optimization and subsequent frequency analysis yielded the **TS**‐*SS* and **TS**‐*RR* geometries (Figure S5). The TS geometry involves the simultaneous weakening of the O−O bond, forging of the epoxide and release of proton from NH^+^ to *tert*‐butoxide. The C−C−O bond angles in the TSs are 86° for **TS**‐*SS* and 81° for **TS**‐*RR*, with the O‐O distances being very similar (1.92 and 1.97 Å, respectively). In the **TS**‐*SS* geometry, a change in the hydrogen bonding network is required to allow a better geometry for the ring closure. The two hydrogens of the urea are indeed hydrogen bonded with the same oxygen of the sulfone moiety (2.04 and 2.20 Å). Nevertheless, **TS**‐*SS* (Δ*G* ^≠^=13.9 kcal mol^−1^) is lower in energy with respect to **TS**‐*RR* (Δ*G* ^≠^=15.2 kcal mol^−1^). These energy values suggest that ring closure is the rate‐determining step and the observed enantioselectivity is well reproduced by calculations (ΔΔ*G* ^≠^=1.3 kcal mol^−1^ corresponds to 80 % *ee* vs. the experimentally observed 70 % *ee* at 25 °C).

To reach a suitable ring closure geometry for the formation of the (R*,S*) diastereoisomers, a 180° rotation around the C_α_‐C_β_ bond is required, before the intramolecular S_N_2 reaction occurs. A stepwise mechanism, involving decomplexation followed by rotation within the free anion and subsequent complexation seemed to be unlikely, being toluene the solvent. Two rough TS geometries for C_α_‐C_β_ bond rotation were found, using a relaxed scan of the torsion angle, followed by full optimization (Figure S6). In **TS**‐*R*_rot_ the torsion angle O‐C_β_‐C_α_‐CN is ≈165° and the activation energy is slightly above **TS**‐*RR* (15.6 kcal mol^−1^). The same situation happens on the other direction (**TS**‐*S*_rot_=15.7 kcal mol^−1^). Once **II**‐*S*′ and **II**‐*R*′ are formed (Figure S7), the ring closure T*Ss* across **TS**‐*SR* and **TS**‐*RS* leads to the (*S*,*R*) and (*R*,*S*) stereoisomers (see Figure S8). Both were found to be higher in energy with respect to the main pathway within each branch of the reaction. These findings agree with the observed formation of *trans*‐epoxide **2 a** (3D structures of the four stereoisomers of **2 a**, complexed with the catalyst, are reported in Figure S9). Within the branches towards (R*,S*) stereoisomers, the transition state energies for the C_α_‐C_β_ bond rotation and for the two ring closures are very similar. It is thus difficult to predict which is the rate‐determining transition state for the formation of the (R*,S*) diastereoisomer.

## Conclusion

In conclusion, we developed a first catalytic and scalable single‐pot process to efficiently prepare dihydroquinoxalinones in enantioselective and practical manner, capitalizing on optically active 1‐phenylsulfonyl‐1‐cyano epoxides, as novel masked α‐halo acyl halide synthons. This attractive protocol is further simplified by using toluene as the sole reaction solvent, commercial reagents and completely recyclable and readily available organocatalyst. The approach demonstrated to be versatile, giving facile access to differently aryl/alkyl 3‐substituted dihydroquinoxalinones in high enantioselectivity and the potential to access other important related heterocycles has been assessed. Computational investigation suggests that the enantioselectivity is driven by a network of hydrogen bonding between the urea and the sulfone moieties, with the key involvement of the basic tertiary nitrogen to activate the oxidant and to assist ring closure by protonation of the leaving group. The ring closure step of the catalytic oxidative cycle has been found to be rate‐determining. We believe this strategy will open up opportunities for convenient one‐pot asymmetric syntheses of other biologically active heterocycles and carboxylic acid derivatives. Current investigations along this line are ongoing and the results will be reported in due course.

## Conflict of interest

The authors declare no conflict of interest.

## Supporting information

As a service to our authors and readers, this journal provides supporting information supplied by the authors. Such materials are peer reviewed and may be re‐organized for online delivery, but are not copy‐edited or typeset. Technical support issues arising from supporting information (other than missing files) should be addressed to the authors.

Supporting Information

Supporting Information
