# Supplementary material for: Catalytic Enantioselective Access to Dihydroquinoxalinones via Formal α‐Halo Acyl Halide Synthon in One Pot
Source: Angew Chem Int Ed Engl. 2021 Sep 30;60(44):23819–26. doi: 10.1002/anie.202110173 (PMC8596509; doi:10.1002/anie.202110173)
Supplement: Supplementary file 1 — Supporting Information [file ANIE-60-23819-s002.pdf]

Angewandte Chemie
Eine Zeitschrift der Gesellschaft Deutscher Chemiker

Supporting Information

Catalytic Enantioselective Access to Dihydroquinoxalinones via Formal α-Halo Acyl Halide Synthon in One Pot

*Chiara Volpe[+], Sara Meninno[+], Carlo Crescenzi, Michele Mancinelli, Andrea Mazzanti, and Alessandra Lattanzi**

anie_202110173_sm_miscellaneous_information.pdf
anie_202110173_sm_pdb.zip

General Methods S2

Experimental Procedures and Compounds Characterization S3

Synthesis of catalysts S3
General procedure for the synthesis of alkenes 1 .... S3
General procedure for the synthesis of alkenes 1x, 1y, 1ac, 1ad S3
General procedure for the synthesis of alkene 1ab S4
General procedure for the synthesis of racemic epoxide 2a from alkene 1a S7
General procedure for the asymmetric synthesis of epoxide 2a from alkene 1a S8
Table S1. Solvent screening S8
General procedure for the asymmetric one-pot synthesis of epoxide 2a S8
General procedure for the racemic synthesis of heterocycles 3a-z and 3ab-ad S9
General procedure for one-pot asymmetric Knoevenagel/epoxidation/ring-opening reaction to heterocycles 3a-w S9
General procedure for one-pot asymmetric epoxidation/ring-opening reaction to heterocycles 3t-u, 3x-z and 3ab-ad S10
Table S2. Screening of the bases for the asymmetric one-pot synthesis of compound **3w** S11
Synthesis of products 3 by reacting a non-symmetrical *o*-phenylenediamine S23
General procedure for the reduction of 3 to products 4 .... S23
Scale-up of the one-pot asymmetric Knoevenagel/epoxidation/ring-opening reaction to heterocycle 3k with catalyst recycling S25
Mechanistic investigations S26
Table S3. Monitoring of epoxide ring opening reaction over time in toluene-d_8 at rt. S26

NMR spectra S29

HPLC chromatograms S121

DFT calculations S154

General Methods

All reactions requiring dry or inert conditions were conducted in flame-dried glassware under a positive pressure of nitrogen. Anhydrous THF, toluene, *m*-xylene, chlorobenzene, 2-methyltetrahydrofuran were purchased from Aldrich and used as received, all other solvents were dried over molecular sieves. Molecular sieves (Aldrich Molecular Sieves, 3 Å, 1.6 mm pellets) were activated under vacuum at 200 °C overnight. Reactions were monitored by thin layer chromatography (TLC) on Macherey-Nagel pre-coated silica gel plates (0.25 mm) and visualized by UV light. Flash chromatography was performed on Merck silica gel (60, particle size: 0.040–0.063 mm). ^{1}H NMR and ^{13}C NMR spectra were recorded on Bruker Avance III HD 600, Bruker Avance-400, Bruker Avance-300 or Bruker Avance-250 spectrometer in $CDCl_3$. Chemical shifts for protons are reported using residual solvent protons (δ = 7.26 ppm for $CDCl_3$, δ = 3.31 ppm for MeOD) as internal standard. Carbon spectra were referenced to the shift of the ^{13}C signal of $CDCl_3$ (δ = 77.0 ppm) or MeOD (δ = 49.0 ppm).

The following abbreviations are used to indicate the multiplicity in NMR spectra: s - singlet; d - doublet; t - triplet; q - quartet; dd – double doublet; ddd – doublet of doublet of doublets; dtd - doublet of triplets of doublets; dq – doublet of quartets; td – triplet of doublets; qd – quartet of doublets; m - multiplet; bs - broad signal.

Optical rotation of compounds was performed on a Jasco P-2000 digital polarimeter using WI (Tungsten-Halogen) lamp (λ = 589 nm). FTIR spectra were recorded as thin films on KBr plates using Bruker Tensor 27 spectrometer and absorption maxima are reported in wavenumber (cm^{-1}). High resolution mass spectra (HRMS) were acquired using a Bruker solariX XR Fourier transform ion cyclotron resonance mass spectrometer (Bruker Daltonik GmbH, Bremen, Germany) equipped with a 7 T refrigerated actively-shielded superconducting magnet. The samples were ionized in positive ion mode using a MALDI or ESI ionization sources. Melting points were measured with a Stuart Model SMP 30 melting point apparatus and are uncorrected.

Petrol ether (PE) refers to light petroleum ether (boiling point 40-60 °C). All starting materials (unless otherwise noted) were purchased from Merck-SigmaAldrich or TCI-Europe and used as received.

Alkenes **1a-b**,[1a] **1d-e**,[1a] **1h**,[1c] **1j**,[1a] **1k-l**,[1b] are known compounds, they were prepared according to the literature. 4,5-dimethyl-*o*-phenylenediamine, 4,5-dichloro-*o*-phenylenediamine and *o*-aminothiophenol were purchased from TCI and used as received. *N*,*N*-methylphenylenediamine is a known compound, it was prepared according to the literature.[2] (Phenylsulfonyl)acetonitrile, *o*-

---

[1] (a) Pandit, K. S.; Kupwade, R. V.; Chavan, P. V.; Desai, U. V.; Wadgaonkar, P. P.; Kodam, K. M. *ACS Sustainable Chem. Eng.* **2016**, *4*, 3450; (b) Rajkumar, S.; Shankland, K.; Goodman, J. M.; Cobb, A. J. A. *Org. Lett.* **2013**, *15*, 1386; (c) Nemcsok, T.; Rapi, Z.; Bagi, P.; Guan, Y. H.; Orbán, I.; Keglevich, G.; Bakó, P. *Tetrahedron* **2020**, *76*, 130965.

[2] Shen, G.-B.; Xia, K.; Li, X.-T.; Li, J.-L.; Fu, Y.-H.; Yuan, L.; Zhu, X.-Q. *J. Phys. Chem. A* **2016**, *120*, 1779.

phenylenediamine, 2,3-diaminonaphthalene and *o*-aminophenol were purchased from Merck-SigmaAldrich and catalyst **I** from Strem Chemicals and they were used as received. Enantiomeric excess of epoxide **2a**, heterocycles **3** and products **4** was determined by HPLC (Waters-Breeze 2487, UV dual λ absorbance detector and 1525 Binary HPLC Pump) using Daicel chiral columns.

Experimental Procedures and Compounds Characterization

Synthesis of catalysts

Cinchona alkaloids were purchased from Aldrich and used as received. Amine-thioureas **eQNT**, **eCDT**, **eHQNT**, **eQDT** were synthesized according to the literature.[3] Catalysts **eQNS**[4] and **eQNU**[5] are known compounds, they were prepared according to the literature.

General procedure for the synthesis of alkenes 1

Alkenes **1** were prepared according to the literature.[1a]

To a suspension of (phenylsulfonyl)acetonitrile (240.4 mg, 1.3 mmol; 37.0 mg, 0.2 mmol for alkene **1o**) and appropriate aromatic aldehyde (1.3 mmol; 34.2 mg, 0.2 mmol for alkene **1o**) in anhydrous ethanol (7 mL; 1 mL for alkene **1o**), pyrrolidine (33 µL, 0.39 mmol; 5 µL, 0.06 mmol for alkene **1o**) was added. The reaction mixture was stirred at rt for 2-7 hours, monitored by TLC (eluent PE/ethyl acetate 8/2). Upon completion, water was added (≈ 20 mL) until the precipitation of the solid that was filtered, washed with hexane/chloroform mixture 8/2 (3x10 mL) and dried. The products were isolated in pure form and no further purification was needed (40-81% yield).

General procedure for the synthesis of alkenes 1x, 1y, 1ac, 1ad

Alkenes **1x, 1y, 1ac** and **1ad** were prepared according to a slightly modified literature procedure.[6]

(Phenylsulfonyl)acetonitrile (333 mg, 1.8 mmol for **1x**, 836 mg, 4.6 mmol for **1y**, 670 mg, 3.6 mmol for **1ac**, 4.0 mmol for **1ad**, respectively) was dissolved in dichloromethane (C = 0.7 M) in a round bottomed flask under nitrogen atmosphere. Then, octanal (284 µL, 1.8 mmol) or isovaleraldehyde (663 µL, 6 mmol) or hex-5-enal (362 mg, 3.6 mmol) or pent-4-ynal (370 mg, 4.5 mmol) and Ti(O-*i*-Pr)$_4$ (20-50 mol%) were added and the solution was stirred at room temperature overnight. The reaction mixture was then poured into 1N HCl and vigorously stirred at 0 °C for 30 minutes. The aqueous phase was extracted with dichloromethane (3x30 mL) and then the combined organic phases were washed with sodium bicarbonate (1x50 mL) and brine (1x50 mL). The organic layer was dried over Na_2SO_4, filtered and concentrated under vacuum. The crude reaction mixture was then purified

---

[3] Vakulya, B.; Varga S.; Csámpai, A.; Soós T. *Org. Lett.* **2005**, *7*, 1967.
[4] Yang, W.; Du, D.-M. *Org. Lett.* **2010**, *12*, 5450.
[5] (a) Miyaji, R.; Asano K.; Matsubara, S. *Org. Lett.* **2013**, *15*, 3658. (b) Amere, M.; Lasne, M.-C.; Rouden, J. *Org. Lett.* **2007**, *9*, 2621. (c) Wu, W.; Min, L.; Zhu, L.; Lee, C.-S. *Adv. Synth. Catal.* **2013**, *353*, 1135.
[6] Yamashita, K.; Tanaka, T.; Hayashi, M. *Tetrahedron* **2005**, *61*, 7981.

by flash chromatography (eluting from PE/ethyl acetate 100/0 to 80/20) affording the corresponding alkenes in 10-31% yield.

General procedure for the synthesis of alkene 1ab

Alkene **1ab** was prepared according to a slightly modified literature procedure.[1b]

To a suspension of (phenylsulfonyl)acetonitrile (240.4 mg, 1.3 mmol) and cyclohexanecarboxaldehyde (162 µL, 1.3 mmol) in toluene (4.3 mL), 3 Å molecular sieves (~800 mg), piperidine (26 µL, 0.26 mmol) and acetic acid (22 µL, 0.39 mmol) were added. The resulting mixture was heated at 50 °C. After 63 h, the reaction was cooled to room temperature and water was added. This was then extracted with ethyl acetate (4x30 mL) and the combined organic layers were dried over Na_2SO_4, filtered and concentrated under vacuum. The crude reaction mixture was then purified by flash chromatography (eluting from PE/ethyl acetate 100/0 to 96/4) affording alkene **1ab** in 40% yield (143 mg).

(*E*)-2-(phenylsulfonyl)-3-(*m*-tolyl)acrylonitrile (1c)

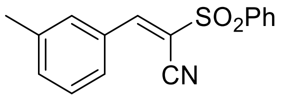

White solid. **mp** 121.5-122.8 °C. **FTIR**ν_{max}(KBr)/cm^{-1}: 3448, 1579, 1330, 1157, 1086, 747. **^{1}H NMR** ($CDCl_3$, 600 MHz): δ 8.20 (s, 1H), 8.02 (d, 2H, J = 8.0 Hz), 7.74-7.73 (m, 1H), 7.72-7.69 (m, 2H), 7.61 (t, 2H, J = 8.0 Hz), 7.39-7.38 (m, 2H), 2.40 (s, 3H). **^{13}C NMR** ($CDCl_3$, 150 MHz): δ 151.7, 139.4, 138.0, 135.0, 134.5, 131.6, 130.1, 129.6, 129.3, 128.6, 128.1, 114.3, 113.1, 21.2. **HRMS (ESI-FT ICR)** exact mass [M+Na]$^+$ calculated for $C_{16}H_{13}NNaO_2S$: 306.0559, found: 306.0563.

(*E*)-3-(4-fluorophenyl)-2-(phenylsulfonyl)acrylonitrile (1f)

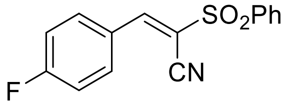

White solid. **mp** 137.9-139.3 °C. **FTIR**ν_{max}(KBr)/cm^{-1}: 3440, 1610, 1470, 1333, 1150, 730, 687. **^{1}H NMR** ($CDCl_3$, 600 MHz): δ 8.20 (s, 1H), 8.02 (dd, 2H, J = 8.0, 1.4 Hz), 7.97 (d, 1H, J = 5.2 Hz), 7.96 (d, 1H, J = 5.2 Hz), 7.71 (tt, 1H, J = 8.0, 1.4 Hz), 7.62 (td, 2H, J = 8.0, 1.4 Hz), 7.19 (t, 2H, J = 8.6 Hz). **^{13}C NMR** ($CDCl_3$, 150 MHz): δ 165.9 (d, $^1J_{CF}$ = 257.3 Hz), 150.0, 137.8, 134.7, 133.6 (d, $^3J_{CF}$ = 9.3 Hz), 129.7, 128.6, 126.5 (d, $^4J_{CF}$ = 3.1 Hz), 117.0 (d, $^2J_{CF}$ = 22.2 Hz), 114.3, 113.0. **HRMS (ESI-FT ICR)** exact mass [M+Na]$^+$ calculated for $C_{15}H_{10}FNNaO_2S$: 310.0308, found: 310.0312.

(*E*)-3-(2-fluorophenyl)-2-(phenylsulfonyl)acrylonitrile (1g)

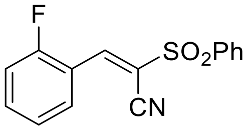

White solid. **mp** 111.7-112.9 °C. **FTIR**ν_{max}(KBr)/cm^{-1}: 3460, 1609, 1333, 1159, 1087, 755, 685. **^{1}H NMR** ($CDCl_3$, 400 MHz): *δ* 8.54 (s, 1H), 8.21 (t, 1H, *J* = 8.2 Hz), 8.03 (d, 2H, *J* = 7.8 Hz), 7.73 (td, 1H, *J* = 7.8, 0.8 Hz), 7.63 (t, 2H, *J* = 7.8 Hz), 7.59-7.56 (m, 1H), 7.27 (t, 1H, *J* = 8.2 Hz), 7.21 (t, 1H, *J* = 8.2 Hz). **^{13}C NMR** ($CDCl_3$, 100 MHz): *δ* 161.9 (d, $^{1}J_{CF}$ = 257.6 Hz), 143.0 (d, $^{3}J_{CF}$ = 8.1 Hz), 137.5, 136.1 (d, $^{3}J_{CF}$ = 9.3 Hz), 134.8, 129.7, 128.8, 128.7, 125.1 (d, $^{4}J_{CF}$ = 3.6 Hz), 118.6 (d, $^{2}J_{CF}$ = 10.6 Hz), 116.45 (d, $^{2}J_{CF}$ = 21.4 Hz), 116.43, 112.8. **HRMS (ESI-FT ICR)** exact mass $[M+Na]^+$ calculated for $C_{15}H_{10}FNNaO_2S$: 310.0309, found: 310.0313.

(*E*)-2-(phenylsulfonyl)-3-(4-(trifluoromethylthio)phenyl)acrylonitrile (1i)

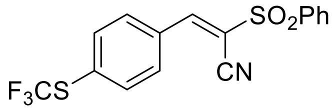

White solid. **mp** 113.6-114.5 °C. **FTIR**ν_{max}(KBr)/cm^{-1}: 3445, 1604, 1331, 1157, 1084, 756. **^{1}H NMR** ($CDCl_3$, 600 MHz): *δ* 8.24 (s, 1H), 8.02 (dd, 2H, *J* = 8.4, 1.2 Hz), 7.95 (d, 2H, *J* = 8.4 Hz), 7.76-7.72 (m, 3H), 7.64 (d, 1H, *J* = 7.5 Hz), 7.62 (d, 1H, *J* = 7.5 Hz). **^{13}C NMR** ($CDCl_3$, 150 MHz): *δ* 149.5, 137.3, 136.0, 134.9, 131.9, 131.4, 129.1 (q, $^{1}J_{CF}$ = 307.0 Hz), 131.2 (q, $^{3}J_{CF}$ = 1.6 Hz), 129.8, 128.0, 117.1, 112.6. **HRMS (ESI-FT ICR)** exact mass $[M+Na]^+$ calculated for $C_{16}H_{10}F_3NNaO_2S_2$: 391.9997, found: 391.9995.

(*E*)-3-(3,4-dichlorophenyl)-2-(phenylsulfonyl)acrylonitrile (1m)

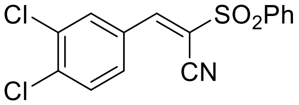

White solid. **mp** 110.0-111.8 °C. **FTIR**ν_{max}(KBr)/cm^{-1}: 3446, 1606, 1472, 1331, 1158, 726, 685. **^{1}H NMR** ($CDCl_3$, 600 MHz): *δ* 8.13 (s, 1H), 8.02-8.01 (m, 2H), 7.95 (d, 1H, *J* = 2.1 Hz), 7.80 (dd, 1H, *J* = 8.3, 2.1 Hz), 7.74 (tt, 1H, *J* = 7.7, 1.0 Hz), 7.63 (t, 2H, *J* = 7.7 Hz), 7.58 (d, 1H, *J* = 8.3 Hz). **^{13}C NMR** ($CDCl_3$, 150 MHz): *δ* 148.4, 138.5, 137.3, 134.9, 134.1, 132.6, 131.5, 129.83, 129.79, 129.2, 128.8, 116.7, 112.5. **HRMS (ESI-FT ICR)** exact mass $[M+Na]^+$ calculated for $C_{15}H_9Cl_2NNaO_2S$: 359.9623, found: 359.9620.

(*E*)-3-(3,5-bis(trifluoromethyl)phenyl)-2-(phenylsulfonyl)acrylonitrile (1n)

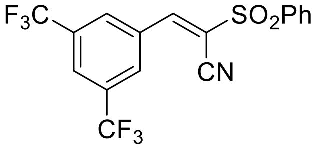

White solid. **mp** 135.9-136.9 °C. **FTIR**ν_{max}(KBr)/cm^{-1}: 2920, 1450, 1333, 1138, 920, 736. **^{1}H NMR** ($CDCl_3$, 600 MHz): *δ* 8.34 (s, 2H), 8.31 (s, 1H), 8.05-8.03 (m, 3H), 7.77 (t, 1H, *J* = 7.4 Hz), 7.66 (t, 2H, *J* = 7.4 Hz). **^{13}C NMR** ($CDCl_3$, 150 MHz): *δ* 147.3, 136.8, 135.3, 133.3 (q, $^{2}J_{CF}$ = 34.1 Hz), 132.0, 130.1 (q, $^{3}J_{CF}$ = 2.7 Hz), 130.0, 129.0, 126.6 (q, $^{3}J_{CF}$ = 3.5 Hz), 122.5 (q, $^{1}J_{CF}$ = 271.6 Hz),

119.5, 112.0. **HRMS (ESI-FT ICR)** exact mass $[M+Na]^+$ calculated for $C_{17}H_9F_6NNaO_2S$: 428.0156, found: 428.0161.

(*E*)-3-(2-methylquinolin-3-yl)-2-(phenylsulfonyl)acrylonitrile (1o)

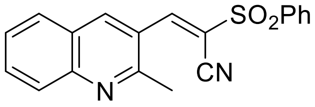

Light brown solid. **mp** 159.9-162.2 °C. **FTIR**ν_{max}(KBr)/cm^{-1}: 3449, 1686, 1449, 1333, 1160, 785, 687. ^{1}**H NMR** ($CDCl_3$, 600 MHz): δ 8.81 (s, 1H), 8.64 (s, 1H), 8.07 (d, 2H, *J* = 7.5 Hz), 8.02 (d, 1H, *J* = 7.9 Hz), 7.86 (d, 1H, *J* = 7.9 Hz), 7.81 (t, 1H, *J* = 7.9 Hz), 7.76 (t, 1H, *J* = 7.5 Hz), 7.66 (t, 2H, *J* = 7.9 Hz), 7.56 (t, 1H, *J* = 7.5 Hz), 2.88 (s, 3H). ^{13}**C NMR** ($CDCl_3$, 150 MHz): δ 157.4, 149.2, 147.9, 137.4, 137.0, 134.9, 132.7, 129.9, 128.90, 128.85, 128.7, 127.2, 125.9, 123.1, 117.9, 112.7, 23.8. **HRMS (ESI-FT ICR)** exact mass $[M+Na]^+$ calculated for $C_{19}H_{14}N_2NaO_2S$: 357.0668, found: 357.0660.

(*E*)-3-(naphthalen-2-yl)-2-(phenylsulfonyl)acrylonitrile (1q)

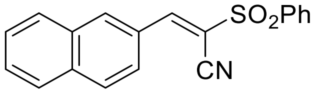

Light yellow solid. **mp** 134.2-135.1 °C. **FTIR**ν_{max}(KBr)/cm^{-1}: 3445, 1635, 1330, 1152, 596. ^{1}**H NMR** ($CDCl_3$, 600 MHz): δ 8.38 (s, 1H), 8.31 (s, 1H), 8.07-8.05 (m, 3H), 7.92 (d, 1H, *J* = 8.0 Hz), 7.89 (d, 1H, *J* = 8.0 Hz), 7.86 (d, 1H, *J* = 8.0 Hz), 7.71 (tt, 1H, *J* = 7.6, 1.1 Hz), 7.65-7.61 (m, 3H), 7.57 (td, 1H, *J* = 8.0, 1.1 Hz). ^{13}**C NMR** ($CDCl_3$, 150 MHz): δ 151.5, 138.0, 135.7, 134.7, 134.6, 132.7, 129.6, 129.5, 129.44, 129.40, 128.6, 127.9, 127.6, 127.4, 124.5, 114.2, 113.3. **HRMS (ESI-FT ICR)** exact mass $[M+Na]^+$ calculated for $C_{19}H_{13}NNaO_2S$: 342.0559, found: 342.0562.

(*E*)-2-(phenylsulfonyl)dec-2-enenitrile (1x)

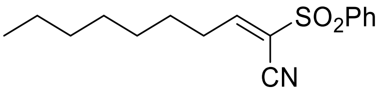

Colourless oil. **FTIR**ν_{max}(KBr)/cm^{-1}: 2929, 1614, 1333, 1161, 1089, 772. ^{1}**H NMR** ($CDCl_3$, 400 MHz): δ 7.95 (d, 2H, *J* = 7.6 Hz), 7.71 (t, 1H, *J* = 7.6 Hz), 7.65 (t, 1H, *J* = 7.7 Hz), 7.60 (t, 2H, *J* = 7.6 Hz), 2.51 (q, 2H, *J* = 7.7 Hz), 1.59-1.52 (m, 2H), 1.29-1.25 (m, 6H), 0.88-0.85 (m, 5H). ^{13}**C NMR** ($CDCl_3$, 100 MHz): δ 160.5, 137.6, 134.6, 129.6, 128.5, 120.8, 111.2, 31.8, 31.5, 29.0, 28.7, 27.6, 22.5, 14.0. **HRMS (ESI-FT ICR)** exact mass $[M+H]^+$ calculated for $C_{16}H_{22}NO_2S$: 292.1366, found: 292.1370.

(*E*)-5-methyl-2-(phenylsulfonyl)hex-2-enenitrile (1y)

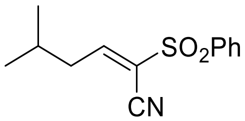

Colourless oil. **FTIR**ν_{max}(KBr)/cm^{-1}: 2960, 2873, 2225, 1615, 1332, 1149, 766, 733, 686, 621, 597. **^{1}H NMR** ($CDCl_3$, 400 MHz): δ 7.93 (d, 2H, *J* = 7.6 Hz), 7.71-7.64 (m, 2H), 7.58 (t, 2H, *J* = 7.6 Hz), 2.40 (t, 2H, *J* = 7.4 Hz), 1.91 (ept, 1H, *J* = 6.7 Hz), 0.94 (d, 6H, *J* = 6.7 Hz). **^{13}C NMR** ($CDCl_3$, 100 MHz): δ 159.5, 137.6, 134.6, 129.6, 128.3, 121.4, 111.1, 40.3, 28.0, 22.1. **HRMS (MALDI-FT ICR)** exact mass $[M+Na]^+$ calculated for $C_{13}H_{15}NNaO_2S$: 272.0716, found: 272.0756.

(*E*)-3-cyclohexyl-2-(phenylsulfonyl)acrylonitrile (1ab)

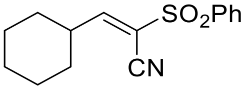

Colourless oil. **FTIR**ν_{max}(KBr)/cm^{-1}: 2928, 1610, 1330, 1167, 1088, 747, 686. **^{1}H NMR** ($CDCl_3$, 300 MHz): δ 7.94 (d, 2H, *J* = 7.5 Hz), 7.70 (t, 1H, *J* = 7.5 Hz), 7.59 (t, 2H, *J* = 7.5 Hz), 7.49 (d, 1H, *J* = 10.3 Hz), 2.61-2.58 (m, 1H), 1.76-1.73 (m, 5H), 1.34-1.25 (m, 5H). **^{13}C NMR** ($CDCl_3$, 75 MHz): δ 164.1, 137.7, 134.6, 129.7, 128.5, 118.8, 111.3, 41.2, 31.0, 25.1, 24.7. **HRMS (ESI-FT ICR)** exact mass $[M+Na]^+$ calculated for $C_{15}H_{17}NNaO_2S$: 298.0872, found: 298.0876.

(*E*)-2-(phenylsulfonyl)octa-2,7-dienenitrile (1ac)

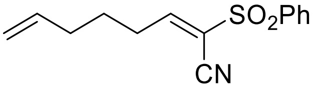

Colourless oil. **FTIR**ν_{max}(KBr)/cm^{-1}: 2927, 2851, 2200, 1726, 1641, 1333, 1159, 757, 729, 687, 621, 598. **^{1}H NMR** ($CDCl_3$, 400 MHz): δ 7.96 (d, 2H, *J* = 7.1 Hz), 7.72 (t, 1H, *J* = 7.5 Hz), 7.67-7.59 (m, 3H), 5.79-5.68 (m, 1H), 5.04-5.00 (m, 2H), 2.53 (q, J = 2H, *J* = 7.6), 2.15 (q, 2H, *J* = 7.0 Hz), 1.72 – 1.64 (m, 2H). **^{13}C NMR** ($CDCl_3$, 150 MHz): δ 160.0, 137.6, 136.6, 134.7, 129.7, 128.5, 121.1, 116.3, 111.1., 32.9, 31.1, 26.7. **HRMS (MALDI-FT ICR)** exact mass $[M+Na]^+$ calculated for $C_{14}H_{15}NNaO_2S$: 284.0716, found: 284.0764.

(*E*)-2-(phenylsulfonyl)hept-2-en-6-ynenitrile (1ad)

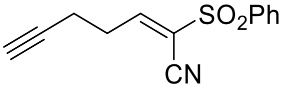

Colourless oil. **FTIR**ν_{max}(KBr)/cm^{-1}: 2958, 2860, 2227, 1640, 1332, 1150, 760, 730, 685, 620, 599. **^{1}H NMR** ($CDCl_3$, 600 MHz): 7.98-7.95 (m, 2H), 7.73-7.70 (m, 2H), 7.62-7.60 (m, 2H), 2.73 (ddd, 2H, *J* = *J* = *J* = 7.0 Hz), 2.48 (td, 2H, *J* = 6.7, 2.7 Hz), 2.03 (t, 1H, *J* = 2.7 Hz). **^{13}C NMR** ($CDCl_3$, 150 MHz): δ 157.3, 137.4, 134.8, 129.7, 128.7, 122.4, 110.9, 80.5, 71.2, 30.3. **HRMS (MALDI-FT ICR)** exact mass $[M+H]^+$ calculated for $C_{13}H_{12}NO_2S$: 246.0583, found: 246.0595.

General procedure for the synthesis of racemic epoxide 2a from alkene 1a

In a sample vial containing a solution of alkene **1a** (0.2 mmol) and triethylamine (8 µL, 0.06 mmol) in anhydrous toluene (1 mL), TBHP (~5.5 M in decane, 47 µL, 0.26 mmol) was added. The reaction

was stirred at rt for 3 hours, monitored by TLC (eluent PE/ethyl acetate 80/20). The product was isolated by flash chromatography (eluting from PE/ethyl acetate 100/0 to 90/10).

General procedure for the asymmetric synthesis of epoxide 2a from alkene 1a

In a sample vial containing a solution of alkene **1a** (0.1 mmol) and **eQNU** (5.8 mg, 0.01 mmol) in anhydrous toluene (5 mL) at -20°C, CHP (tech. 80%, 24 μL, 0.13 mmol) was added. The reaction was stirred at -20°C for 14 hours, monitored by TLC (eluent PE/ethyl acetate 80/20). Purification of the crude reaction mixture by flash chromatography (eluting with PE/ethyl acetate 100/0 to 85/15) gave enantioenriched epoxide **2a** in 94% yield and 93% ee.

Table S1. Solvent screening[a]

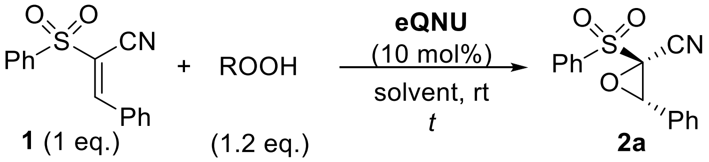

Entry	ROOH	solvent	*t* [h]	Yield **2a** [%][b]	ee **2a** [%][c]
1	TBHP	toluene	3	90	-70
2	TBHP	*m*-xilene	3	84	-67
3	TBHP	chlorobenzene	3	87	-67
4	TBHP	dichloromethane	4.5	94	-68
5	TBHP	2-MeTHF	15	78	-66
6	TBHP	toluene/hexane 1/1	4	91	-70
7	TBHP	mesitylene	3	95	-70
8	CHP	toluene	1	90	-86

[a] *Reaction conditions*: **1** (0.1 mmol), ROOH (0.12 mmol) and **eQNU** (0.01 mmol) in anhydrous solvent (0.5 mL). [b] Yields of isolated product. [c] HPLC analysis on a chiral stationary phase.

General procedure for the asymmetric one-pot synthesis of epoxide 2a

A sample vial was charged with (phenylsulfonyl)acetonitrile (0.12 mmol) and **eQNU** (6.9 mg, 0.012 mmol) in anhydrous toluene (0.4 mL). Then benzaldehyde (0.12 mmol) was added and the solution was stirred at 30 °C for 6.5 hours (TLC eluent PE/ ethyl acetate 80/20). At the end of the first step, toluene (5.6 mL) and cumene hydroperoxide (tech. 80%, 24 μL, 0.132 mmol) were added and the solution was stirred at -20 °C for 17 hours (TLC eluent PE/ ethyl acetate 80/20). Purification of the crude reaction mixture by flash chromatography (eluting with PE/ethyl acetate 100/0 to 85/15) gave enantioenriched epoxide **2a** in 85% yield and 92% ee.

(2*S*,3*S*)-3-phenyl-2-(phenylsulfonyl)oxirane-2-carbonitrile (2a)

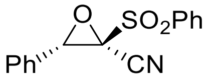

White solid, 26.8 mg, 94% yield. **mp** 115.2-116.6 °C. $[\alpha]_D^{21}$ = -33.9 (C = 0.33, $CHCl_3$), 93% ee. **FTIR**ν_{max}(KBr)/cm^{-1}: 3068, 1448, 1350, 1166, 1087, 915, 757. **^{1}H NMR** ($CDCl_3$, 300 MHz): δ 8.07-8.06 (m, 2H), 7.87-7.81 (m, 1H), 7.71 (t, 2H, J = 7.7 Hz), 7.48-7.38 (m, 5H), 5.05 (s, 1H). **^{13}C NMR** ($CDCl_3$, 75 MHz): δ 135.9, 134.2, 130.8, 129.9, 129.0, 128.4, 126.7, 111.0, 67.5, 63.7. **HRMS (ESI-FT ICR)** exact mass $[M+Na]^+$ calculated for $C_{15}H_{11}NNaO_3S$: 308.0352, found: 308.0357. HPLC analysis with Chiralcel OD-H column, 90:10 *n*-hexane:2-propanol, 1 mL/min, 220 nm; minor enantiomer t_R = 9.5 min, major enantiomer t_R = 12.7 min.

General procedure for the racemic synthesis of heterocycles 3a-z and 3ab-ad

In a sample vial containing the opportune alkene **1** (0.12 mmol) and anhydrous toluene (0.6 mL), TBHP (~5.5 M in decane, 28 µL, 0.16 mmol) and triethylamine (5 µL, 0.036 mmol) were added. The reaction mixture was stirred until consumption of the alkene **1** (TLC eluent PE/ethyl acetate 8/2). Then *o*-phenylenediamines, *o*-aminothiophenol or *o*-aminophenol (0.132 mmol) and triethylamine (17 µL, 0.24 mmol, 2 eq.) were added. The reaction mixture was stirred at room temperature (50 °C for products **3x-z** and **3ab-ad**) and monitored by TLC (eluent PE/ ethyl acetate 8/2 to check if the epoxide has been consumed). After completion, the mixture was diluted with ethyl acetate and water and HCl 1N was added to pH 7. The aqueous layer was extracted with ethyl acetate (3x20 mL). The combined organic layers were washed with saturated NaCl solution (1x40 mL), dried over anhydrous Na_2SO_4 and concentrated under vacuum. The reaction mixture was purified by flash chromatography (eluent: PE/ethyl acetate 90/10 to 70/30; PE/ethyl acetate 90/10 to 50/50 for product **3o**) to give racemic products **3** in 30-90% yield.

General procedure for one-pot asymmetric Knoevenagel/epoxidation/ring-opening reaction to heterocycles 3a-w

In a sample vial containing (phenylsulfonyl)acetonitrile (22.2 mg, 0.12 mmol) and **eQNU** (6.9 mg, 0.012 mmol) in anhydrous toluene (0.4 mL), the opportune aldehyde (0.12 mmol) was added. The reaction was stirred at 30 °C for 7.5-24 hours, monitored by TLC (eluent PE/ethyl acetate 8/2). After completion, toluene (5.6 mL) was added. Then 1.1 equivalents of cumene hydroperoxide (tech. 80%, 24 µL, 0.132 mmol) was added at – 20 °C and the solution was stirred at -20 °C until consumption of the alkene **1** (TLC eluent PE/ ethyl acetate 8/2). Then *o*-phenylenediamines, *o*-aminothiophenol or *o*-aminophenol (0.132 mmol) and triethylamine (33 µL, 0.24 mmol; DIPEA 42 µL, 0.24 mmol for product **3w**) were added and the reaction mixture was stirred for 5-23 hours at room temperature, monitored by TLC (eluent PE/ ethyl acetate 8/2) to check the conversion of epoxide into the product.

After completion, the mixture was diluted with ethyl acetate and water and HCl 1N was added to pH 7. The aqueous layer was extracted with ethyl acetate (3x20 mL). The combined organic layers were washed with saturated NaCl solution (1x40 mL), dried over anhydrous Na_2SO_4 and concentrated under reduced pressure. The reaction mixture was purified by flash chromatography (eluent: PE/ethyl acetate 90/10 to 70/30, PE/ethyl acetate 90/10 to 50/50 for product **3o**) to give enantioenriched products **3** in 60-81% yield and 62-99% ee.

General procedure for one-pot asymmetric epoxidation/ring-opening reaction to heterocycles 3t-u, 3x-z and 3ab-ad

In a sample vial containing the opportune alkene **1** (0.12 mmol) and **eQNU** (6.9 mg, 0.012 mmol) in anhydrous toluene (6.0 mL), CHP (tech. 80%, 24 μL, 0.132 mmol) was added at -20 °C and the solution was stirred at -20 °C for 9-42 hours until consumption of the alkene **1** (TLC eluent PE/ethyl acetate 8/2). Then the opportune *o*-phenylenediamine (0.132 mmol) and triethylamine (33 μL, 0.24 mmol) were added and the reaction mixture was stirred at room temperature (50 °C for products **3x-z** and **3ab-ad**), monitored by TLC (eluent PE/ethyl acetate 8/2) to check the conversion of epoxide into the product. After completion, the mixture was diluted with ethyl acetate and water and HCl 1N was added to pH 7. The aqueous layer was extracted with ethyl acetate (3x20 mL). The combined organic layers were washed with saturated NaCl solution (1x40 mL), dried over anhydrous Na_2SO_4 and concentrated under reduced pressure. The reaction mixture was purified by flash chromatography (eluent: PE/ethyl acetate 90/10 to 70/30) to give enantioenriched products **3** in 50-79% yield and 87-96% ee.

Table S2. Screening of the bases for the asymmetric one-pot synthesis of compound **3w**[a]

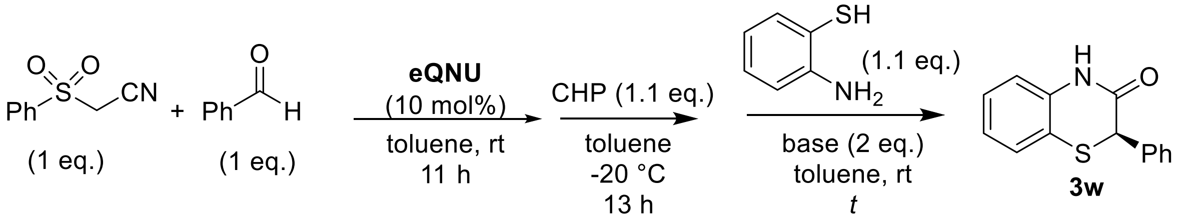

Entry	base	*t* [h]	Yield **3w** [%][b]	ee **3w** [%][c]
1	pyridine	7	28	80
2	2,6-lutidine[d]	32	30	79
3	morpholine[e]	8	27	87
4	DABCO	9	40	34
5	4-methylmorpholine	9	55	76
6	DIPEA	8	65	90

[a] *Reaction conditions.* Knoevenagel step: (phenylsulfonyl)acetonitrile (0.1 mmol), benzaldehyde (0.1 mmol) and **eQNU** (0.01 mmol) in anhydrous toluene (0.3 mL). Epoxidation step: the reaction mixture is diluted with toluene (4.7 mL), placed at –20 °C and CHP (0.11 mmol) is added. DROC step: 1,2-aminothiophenol (0.11 mmol) and base (0.2 mmol) are added at rt. [b] Yields of isolated product. [c] HPLC analysis on a chiral stationary phase. [d] 3 eq. of the base were used. [e] The base was added in two portions, adding the second portion after 3 h.

(*R*)-3-phenyl-3,4-dihydroquinoxalin-2(1H)-one (3a)

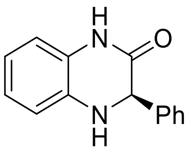

Data for this compound are consistent with those reported in the literature.[7]

White solid, 20.5 mg, 76% yield. **mp** 142.0-145.8 °C. $[\alpha]_D^{24}$ = -87.8 (c 0.33, $CHCl_3$), 89% ee. **FTIR**ν_{max}(KBr)/cm^{-1}: 2917, 2850, 1675, 1605, 1507, 1378, 699. **^{1}H NMR** ($CDCl_3$, 400 MHz): δ 8.57 (bs, 1H), 7.43-7.41 (m, 2H), 7.36-7.31 (m, 3H), 6.92 (td, 1H, J = 7.4, 1.3 Hz), 6.78-6.70 (m, 3H), 5.08 (s, 1H), 4.29 (bs, 1H). **^{13}C NMR** ($CDCl_3$, 100 MHz): δ 167.2, 139.0, 132.9, 128.8, 128.5, 127.2, 124.7, 124.1, 119.4, 115.6, 113.7, 60.7. **HRMS (ESI-FT ICR)** exact mass [M+Na]$^+$ calculated for $C_{14}H_{12}N_2NaO$: 247.0842, found: 247.0844. HPLC analysis with Chiralcel OD-H column, 80:20 *n*-hexane:2-propanol, 1 mL/min, 230 nm; minor enantiomer t_R = 15.9 min, major enantiomer t_R = 23.8 min.

[7] Shi, F.; Tan, W.; Zhang, H.-H.; Li, M.; Ye, Q.; Ma, G.-H.; Tu, S.-J.; Li, G. *Adv. Synth. Catal.* **2013**, *355*, 3715.

(*R*)-3-(*p*-tolyl)-3,4-dihydroquinoxalin-2(1H)-one (3b)

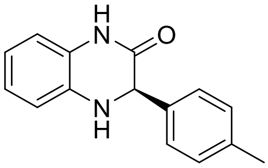

Data for this compound are consistent with those reported in the literature.[8]

Light brown solid, 23.2 mg, 81% yield. **mp** 166.6-169.6 °C. $[\alpha]_D^{25}$ = -94.7 (c 0.33, $CHCl_3$), 91% ee. **FTIR**ν_{max}(KBr)/cm^{-1}: 3307, 1675, 1510, 1460, 1422, 730. **^{1}H NMR** ($CDCl_3$, 400 MHz): δ 8.99 (bs, 1H), 7.29 (d, 2H, *J* = 8.0 Hz), 7.14 (d, 2H, *J* = 8.0 Hz), 6.93-6.88 (m, 1H), 6.74-6.73 (m, 2H), 6.69 (d, 1H, *J* = 7.8 Hz), 5.03 (s, 1H), 4.27 (bs, 1H), 2.32 (s, 3H). **^{13}C NMR** ($CDCl_3$, 100 MHz): δ 167.5, 138.3, 136.1, 133.0, 129.5, 127.1, 124.8, 124.0, 119.3, 115.6, 113.7, 60.4, 21.2. **HRMS (ESI-FT ICR)** exact mass $[M+H]^+$ calculated for $C_{15}H_{15}N_2O$: 239.1179, found: 239.1183. HPLC analysis with Chiralcel OD-H column, 85:15 *n*-hexane:2-propanol, 1 mL/min, 220 nm; minor enantiomer t_R = 28.1 min, major enantiomer t_R = 31.1 min.

(*R*)-3-(m-tolyl)-3,4-dihydroquinoxalin-2(1H)-one (3c)

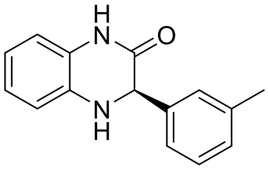

White solid, 20.6 mg, 72% yield. **mp** 200.1-201.8 °C. $[\alpha]_D^{21}$ = -32.6 (c 0.10, MeOH), 91% ee. **FTIR**ν_{max}(KBr)/cm^{-1}: 3300, 1680, 1515, 1460, 1421, 730. **^{1}H NMR** (MeOD, 400 MHz): δ 7.19 (bs, 1H), 7.16-7.14 (m, 2H), 7.08-7.07 (m, 1H), 6.86 (td, 1H, *J* = 7.7, 1.3 Hz), 6.78 (dd, 1H, *J* = 7.7, 1.3 Hz), 6.75 (dd, 1H, *J* = 7.7, 1.3 Hz), 6.67 (td, 1H, *J* = 7.7, 1.3 Hz), 4.92 (s, 1H), 2.32 (s, 3H). **^{13}C NMR** (MeOD, 150 MHz): δ 169.4, 141.2, 139.3, 135.3, 129.7, 129.4, 128.7, 126.4, 125.1, 125.0, 119.5, 116.3, 114.8, 61.6, 21.5. **HRMS (ESI-FT ICR)** exact mass $[M+Na]^+$ calculated for $C_{15}H_{14}N_2NaO$: 261.0998, found: 261.1000. HPLC analysis with Chiralpak AD-H column, 90:10 *n*-hexane:2-propanol, 1 mL/min, 220 nm; minor enantiomer t_R = 32.0 min, major enantiomer t_R = 34.0 min.

(*R*)-3-(4-chlorophenyl)-3,4-dihydroquinoxalin-2(1H)-one (3d)

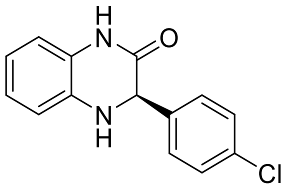

Data for this compound are consistent with those reported in the literature.[7]

[8] Núñez-Rico, J. L.; Vidal-Ferran, A. *Org. Lett.* **2013**, *15*, 2066.

White solid, 24.5 mg, 79% yield. **mp** 124.7-127.5 °C. $[\alpha]_D^{19}$ = -94.2 (c 0.33, $CHCl_3$), 90% ee. **FTIR**ν_{max}(KBr)/cm^{-1}: 2923, 2840, 1610, 1515, 1505, 1470, 1421, 732. **^{1}H NMR** ($CDCl_3$, 400 MHz): δ 8.91 (bs, 1H), 7.36 (d, 2H, J = 8.6 Hz), 7.30 (d, 2H, J = 8.6 Hz), 6.93 (td, 1H, J = 7.5, 1.6 Hz), 6.77 (td, 1H, J = 7.5, 0.9 Hz), 6.74-6.71 (m, 2H), 5.05 (d, 1H, J = 1.6 Hz), 4.27 (bs, 1H). **^{13}C NMR** ($CDCl_3$, 100 MHz): δ 166.8, 137.3, 134.4, 132.6, 128.9, 128.6, 124.6, 124.2, 119.6, 115.7, 113.8, 60.0 . **HRMS (ESI-FT ICR)** exact mass [M+Na]$^+$ calculated for $C_{14}H_{11}ClN_2NaO$: 281.0452, found: 281.0456. HPLC analysis with Chiralpak AD-H column, 90:10 *n*-hexane:2-propanol, 1 mL/min, 220 nm; minor enantiomer t_R = 35.8 min, major enantiomer t_R = 33.1 min.

(*R*)-3-(4-bromophenyl)-3,4-dihydroquinoxalin-2(1H)-one (3e)

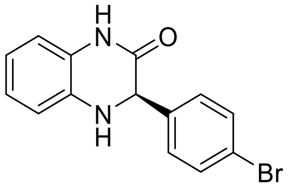

Light brown solid, 27.6 mg, 76% yield. **mp** 202.3-205.6 °C. $[\alpha]_D^{22}$ = -51.7 (c 0.14, $CHCl_3$), 96% ee. **FTIR**ν_{max}(KBr)/cm^{-1}: 2932, 2860, 1612, 1515, 1504, 1470, 1420, 730. **^{1}H NMR** ($CDCl_3$, 400 MHz): δ 7.66 (bs, 1H), 7.46 (d, 2H, J = 8.3 Hz), 7.30 (d, 2H, J = 8.3 Hz), 6.93 (t, 1H, J = 7.3 Hz), 6.78 (t, 1H, J = 7.3 Hz), 6.74-6.72 (m, 2H), 5.04 (s, 1H), 4.27 (bs, 1H). **^{13}C NMR** ($CDCl_3$, 100 MHz): δ 166.5, 137.8, 132.6, 131.9, 128.9, 124.6, 124.2, 122.6, 119.7, 115.6, 113.9, 60.1. **HRMS (ESI-FT ICR)** exact mass [M+Na]$^+$ calculated for $C_{14}H_{11}BrN_2NaO$: 324.9947, found: 324.9950. HPLC analysis with Chiralpak AD-H column, 80:20 *n*-hexane:2-propanol, 1 mL/min, 210 nm; minor enantiomer t_R = 15.1 min, major enantiomer t_R = 13.7 min.

(*R*)-3-(4-fluorophenyl)-3,4-dihydroquinoxalin-2(1H)-one (3f)

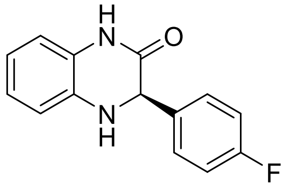

Light brown solid, 18.9 mg, 65% yield. **mp** 166.3-169.2 °C. $[\alpha]_D^{19}$ = -98.8 (c 0.33, $CHCl_3$), 91% ee. **FTIR**ν_{max}(KBr)/cm^{-1}: 3393, 2917, 1675, 1604, 1507, 1376, 745. **^{1}H NMR** ($CDCl_3$, 400 MHz): δ 8.82 (bs, 1H), 7.41.7.38 (m, 2H), 7.02 (t, 2H, J = 8.4 Hz), 6.93 (t, 1H, J = 7.3 Hz), 6.79-6.71 (m, 3H), 5.06 (s, 1H), 4.27 (bs, 1H). **^{13}C NMR** ($CDCl_3$, 100 MHz): δ 167.2, 162.7 (d, $^1J_{CF}$ = 245.6 Hz), 134.7 (d, $^4J_{CF}$ = 2.6 Hz), 132.7, 129.0 (d, $^3J_{CF}$ = 8.1 Hz), 124.7, 124.2, 119.6, 115.72, 115.71 (d, $^2J_{CF}$ = 21.4 Hz), 113.8. 60.0. **HRMS (ESI-FT ICR)** exact mass [M+Na]$^+$ calculated for $C_{14}H_{11}FN_2NaO$: 265.0748, found: 265.0750. HPLC analysis with Chiralcel OD-H column, 80:20 *n*-hexane:2-propanol, 1 mL/min, 220 nm; minor enantiomer t_R = 16.0 min, major enantiomer t_R = 19.3 min.

(*R*)-3-(2-fluorophenyl)-3,4-dihydroquinoxalin-2(1H)-one (3g)

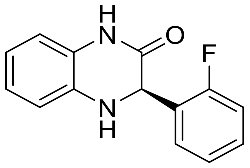

Light yellow solid, 18.9 mg, 65% yield. **mp** 192.4-195.7 °C. $[\alpha]_D^{21}$ = +26.7 (c 0.34, $CHCl_3$), 65% ee. **FTIR**ν_{max}(KBr)/cm^{-1}: 3448, 1669, 1636, 772, 736. **^{1}H NMR** ($CDCl_3$, 600 MHz): δ 8.45 (bs, 1H), 7.38 (t, 1H, J = 7.5 Hz), 7.32-7.29 (m, 1H), 7.12-7.09 (m, 2H), 6.90 (t, 1H, J = 7.3 Hz), 6.79-6.75 (m, 2H), 6.66 (d, 1H, J = 7.7 Hz), 5.42 (s, 1H), 4.20 (bs, 1H). **^{13}C NMR** ($CDCl_3$, 150 MHz): δ 166.1, 160.8 (d, $^1J_{CF}$ = 246.4 Hz), 132.8, 130.2 (d, $^3J_{CF}$ = 8.3 Hz), 128.74, 128.71, 124.9, 124.5 (d, $^4J_{CF}$ = 3.6 Hz), 124.1, 119.7, 115.8 (d, $^2J_{CF}$ = 21.4 Hz), 115.5, 114.1, 54.7. **HRMS (ESI-FT ICR)** exact mass $[M+Na]^+$ calculated for $C_{14}H_{11}FN_2NaO$: 265.0748, found: 265.0748. HPLC analysis with Chiralpak AD-H column, 80:20 *n*-hexane:2-propanol, 1 mL/min, 220 nm; minor enantiomer t_R = 12.1 min, major enantiomer t_R = 14.5 min.

(*R*)-3-(3-methoxyphenyl)-3,4-dihydroquinoxalin-2(1H)-one (3h)

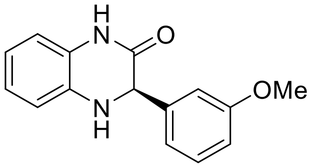

Yellow solid, 22.6 mg, 74% yield. **mp** 131.6 – 133.0 °C. $[\alpha]_D^{25}$ = -72.3 (c 0.53, MeOH), 88% ee. **FTIR**ν_{max}(KBr)/cm^{-1}: 3400, 1672, 1606, 1043, 740. **^{1}H NMR** ($CDCl_3$, 400 MHz): δ 8.55 (bs, 1H), 7.23 (d, 1H, J = 7.9 Hz), 7.00-6.98 (m, 2H), 6.91 (tt, 1H, J = 7.4, 1.3 Hz), 6.84 (dd, 1H, J = 8.2, 2.2 Hz), 6.78-6.70 (m, 3H), 5.05 (s, 1H), 4.29 (bs, 1H), 3.75 (s, 3H). **^{13}C NMR** ($CDCl_3$, 100 MHz): δ 166.8, 159.9, 140.5, 132.9, 129.8, 124.7, 124.1, 119.4, 115.5, 114.0, 113.7, 112.7, 60.6, 55.2. **HRMS (MALDI-FT ICR)** exact mass $[M+H]^+$ calculated for $C_{15}H_{15}N_2O_2$: 255.1128, found: 255.1152. HPLC analysis with Chiralpak IC column, 70:30 *n*-hexane:2-propanol, 1 mL/min, 220 nm; minor enantiomer t_R = 13.7 min, major enantiomer t_R = 9.5 min.

(*R*)-3-(4-((trifluoromethyl)thio)phenyl)-3,4-dihydroquinoxalin-2(1H)-one (3i)

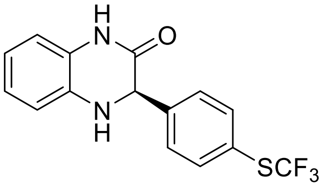

White solid, 23.7 mg, 61% yield. **mp** 193.1-196.3 °C. $[\alpha]_D^{22}$ = -69.6 (c 0.32, $CHCl_3$), 86% ee. **FTIR**ν_{max}(KBr)/cm^{-1}: 3306, 1662, 1596, 1381, 1117, 747, 614. **^{1}H NMR** ($CDCl_3$, 400 MHz): δ 8.19 (bs, 1H), 7.63 (d, 2H, J = 8.1 Hz), 7.50 (d, 2H, J = 8.1 Hz), 6.96 (t, 1H, J = 7.5 Hz), 6.80 (t, 1H, J =

7.5 Hz), 6.75 (t, 2H, *J* = 8.2 Hz), 5.14 (s, 1H), 4.29 (bs, 1H). **^{13}C NMR** ($CDCl_3$, 150 MHz): δ 166.2, 141.8, 136.6, 132.5, 129.5 (q, $^1J_{CF}$ = 306.3 Hz), 128.3, 124.6, 124.5, 124.4, 119.8, 115.7, 113.9, 60.3. **HRMS (ESI-FT ICR)** exact mass $[M+Na]^+$ calculated for $C_{15}H_{11}F_3N_2NaOS$: 347.0436, found: 347.0439. HPLC analysis with Chiralpak AD-H column, 80:20 *n*-hexane:2-propanol, 1 mL/min, 220 nm; minor enantiomer t_R = 11.9 min, major enantiomer t_R = 9.9 min.

(*R*)-4-(3-oxo-1,2,3,4-tetrahydroquinoxalin-2-yl)benzonitrile (3j)

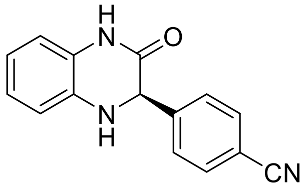

Ochre yellow solid, 21.5 mg, 72% yield. **mp** 177.7-180.3 °C. $[\alpha]_D^{25}$ = -103.3 (c 0.33, $CHCl_3$), 96% ee. **FTIR**ν_{max}(KBr)/cm^{-1}: 3350, 2922, 1675, 1630, 1514, 1460, 730. **^{1}H NMR** ($CDCl_3$, 400 MHz): δ 8.97 (bs, 1H), 7.61 (d, 2H, *J* = 8.3 Hz), 7.56 (d, 2H, *J* = 8.3 Hz), 6.95 (td, 1H, *J* = 7.5, 1.2 Hz), 6.81-6.72 (m, 3H), 5.15 (d, 1H, *J* = 1.6 Hz), 4.36 (bs, 1H). **^{13}C NMR** ($CDCl_3$, 100 MHz): δ 165.9, 143.9, 132.5, 132.2, 128.0, 124.5, 124.4, 120.0, 118.5, 115.8, 114.0, 112.3, 60.2. **HRMS (ESI-FT ICR)** exact mass $[M+Na]^+$ calculated for $C_{15}H_{11}N_3NaO$: 272.0794, found: 272.0797. HPLC analysis with Chiralpak AD-H column, 70:30 *n*-hexane:2-propanol, 1 mL/min, 220 nm; minor enantiomer t_R = 11.7 min, major enantiomer t_R = 9.2 min.

(*R*)-3-(naphthalen-1-yl)-3,4-dihydroquinoxalin-2(1H)-one (3k)

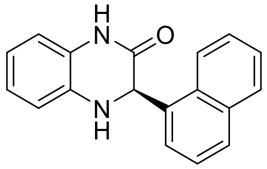

White solid, 24.7 mg, 75% yield. **mp** 215 °C Decomp. $[\alpha]_D^{20}$ = -15.7 (c 0.13, $CHCl_3$), 62% ee. **FTIR**ν_{max}(KBr)/cm^{-1}: 3446, 1674, 1652, 1506, 1375, 1309, 778. **^{1}H NMR** ($CDCl_3$, 400 MHz): δ 8.32 (d, 1H, *J* = 5.4 Hz), 7.90-7.84 (m, 2H), 7.57-7.49 (m, 3H), 7.42 (t, 1H, *J* = 5.4 Hz), 6.92 (td, 1H, *J* = 7.5, 1.5 Hz), 6.80 (t, 1H, *J* = 7.5 Hz), 6.77 (dd, 1H, *J* = 7.6, 1.3 Hz), 6.67 (d, 1H, *J* = 8.0 Hz), 5.77 (s, 1H), 4.24 (d, 1H, *J* = 0.6 Hz). **^{13}C NMR** ($CDCl_3$, 150 MHz): δ 167.3, 134.3, 134.1, 133.4, 131.2, 129.5, 128.9, 126.5, 126.3, 125.9, 125.3, 125.0, 124.11, 124.06, 119.5, 115.5, 113.9, 58.8. **HRMS (ESI-FT ICR)** exact mass $[M+Na]^+$ calculated for $C_{18}H_{14}N_2NaO$: 297.0998, found: 297.1000. HPLC analysis with Chiralpak AD-H column, 70:30 *n*-hexane:2-propanol, 1 mL/min, 220 nm; minor enantiomer t_R = 18.1 min, major enantiomer t_R = 12.8 min.

(*R*)-3-(3-bromophenyl)-6,7-dimethyl-3,4-dihydroquinoxalin-2(1H)-one (3l)

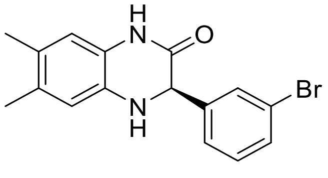

Yellow solid, 27.8 mg, 70% yield. **mp** 158.4-160.6 °C. $[\alpha]_D^{21}$ = -103.4 (c 0.33, $CHCl_3$), 99% ee. **FTIR**ν_{max}(KBr)/cm^{-1}: 2919, 1683, 1620, 1520. **^{1}H NMR** (MeOD, 600 MHz): δ 7.40 (s, 1H), 7.40 (d, 1H, *J* = 7.8 Hz), 7.33 (d, 1H, *J* = 7.8 Hz), 7.20 (t, 1H, *J* = 7.8 Hz), 6.57 (s, 1H), 6.56 (s, 1H), 4.98 (s, 1H), 2.14 (s, 3H), 2.13 (s, 3H). **^{13}C NMR** (MeOD, 150 MHz): δ 168.6, 143.7, 133.2, 132.6, 132.0, 131.3, 131.2, 127.9, 127.0, 124.2, 123.4, 117.6, 116.4, 61.2, 19.4, 19.0. **HRMS (ESI-FT ICR)** exact mass $[M+H]^+$ calculated for $C_{16}H_{16}BrN_2O$: 331.0441, found: 331.0435. HPLC analysis with Chiralpak AD-H column, 80:20 *n*-hexane:2-propanol, 1 mL/min, 220 nm; minor enantiomer t_R = 14.5 min, major enantiomer t_R = 12.4 min.

(*R*)-3-(3,4-dichlorophenyl)-6,7-dimethyl-3,4-dihydroquinoxalin-2(1H)-one (3m)

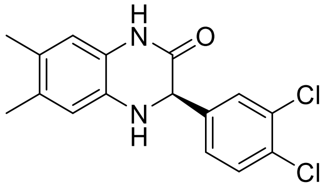

Brown solid, 24.7 mg, 64% yield. **mp** 260 °C Decomp. $[\alpha]_D^{21}$ = -91.8 (c 0.10, MeOH), 98% ee. **FTIR**ν_{max}(KBr)/cm^{-1}: 3447, 1634, 1515, 625. **^{1}H NMR** (MeOD, 600 MHz): δ 7.54 (d, 1H, *J* = 2.0 Hz), 7.44 (d, 1H, *J* = 8.3 Hz), 7.30 (dd, 1H, *J* = 8.3, 2.0 Hz), 6.59 (s, 1H), 6.57 (s, 1H), 4.91 (s, 1H), 2.15 (s, 3H), 2.13 (s, 3H). **^{13}C NMR** (MeOD, 150 MHz): δ 168.3, 141.9, 133.31, 133.25, 132.8, 132.5, 131.5, 130.4, 128.1, 124.2, 117.6, 116.4, 60.7, 19.4, 19.0. **HRMS (ESI-FT ICR)** exact mass $[M+Na]^+$ calculated for $C_{16}H_{14}Cl_2N_2NaO$: 343.0375, found: 343.0376. HPLC analysis with Chiralcel OD-H column, 80:20 *n*-hexane:2-propanol, 1 mL/min, 220 nm; minor enantiomer t_R = 13.0 min, major enantiomer t_R = 20.5 min.

(*R*)-3-(3,5-bis(trifluoromethyl)phenyl)-6,7-dimethyl-3,4-dihydroquinoxalin-2(1H)-one (3n)

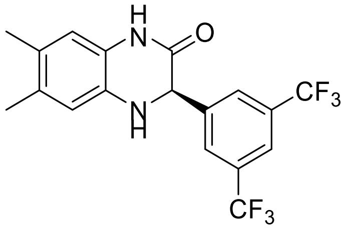

White solid, 30.3 mg, 65% yield. **mp** 290 °C Decomp. $[\alpha]_D^{20}$ = -32.6 (c 0.10, MeOH), 92% ee. **FTIR**ν_{max}(KBr)/cm^{-1}: 2918, 1734, 1635, 1559, 1472, 868, 709. **^{1}H NMR** (MeOD, 400 MHz): δ 8.01 (s, 2H), 7.90 (s, 1H), 6.65 (s, 1H), 6.60 (s, 1H), 5.14 (s, 1H), 2.15 (s, 6H). **^{13}C NMR** (MeOD, 100

MHz): δ 167.9, 144.2, 133.4, 132.7 (q, $^{2}J_{CF}$ = 33.7 Hz), 132.5, 129.1, 128.5, 124.8 (q, $^{1}J_{CF}$ = 270.3 Hz), 124.3, 122.7 (q, $^{3}J_{CF}$ = 4.3 Hz), 117.7, 116.5, 60.9, 19.4, 19.0. **HRMS (ESI-FT ICR)** exact mass $[M+H]^+$ calculated for $C_{18}H_{15}F_6N_2O$: 389.1083, found: 389.1082. HPLC analysis with Chiralpak AD-H column, 90:10 *n*-hexane:2-propanol, 1 mL/min, 220 nm; minor enantiomer t_R = 8.2 min, major enantiomer t_R = 7.6 min.

(*R*)-6,7-dimethyl-3-(2-methylquinolin-3-yl)-3,4-dihydroquinoxalin-2(1H)-one (3o)

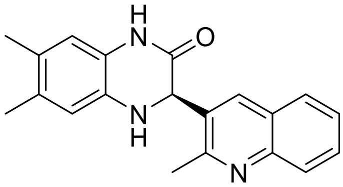

Light yellow solid, 27.4 mg, 72% yield. **mp** 170 °C Decomp. $[\alpha]_D^{20}$ = -164.1 (c 0.15, $CHCl_3$), 86% ee. **FTIR**ν_{max}(KBr)/cm^{-1}: 3459, 1653, 1500, 750. **^{1}H NMR** ($CDCl_3$, 400 MHz): δ 8.71 (bs, 1H), 8.12 (s, 1H), 8.02 (d, 1H, J = 8.4 Hz), 7.70-7.65 (m, 2H), 7.47-7.43 (m, 1H), 6.54 (s, 1H), 6.50 (s, 1H), 5.34 (d, 1H, J = 1.6 Hz), 4.08 (bs, 1H), 2.87 (s, 3H), 2.15 (s, 3H), 2.13 (s, 3H). **^{13}C NMR** ($CDCl_3$, 75 MHz): δ 166.6, 158.2, 147.3, 135.6, 132.3, 130.8, 130.5, 129.8, 128.3, 128.1, 127.6, 126.7, 126.1, 122.8, 116.7, 115.5, 58.8, 23.8, 19.3, 18.9. **HRMS (ESI-FT ICR)** exact mass $[M+H]^+$ calculated for $C_{20}H_{20}N_3O$: 318.1601, found: 318.1601. HPLC analysis with Chiralpak AD-H column, 70:30 *n*-hexane:2-propanol, 1 mL/min, 220 nm; minor enantiomer t_R = 9.8 min, major enantiomer t_R = 8.3 min.

(*R*)-3-(3-bromophenyl)-6,7-dichloro-3,4-dihydroquinoxalin-2(1H)-one (3p)

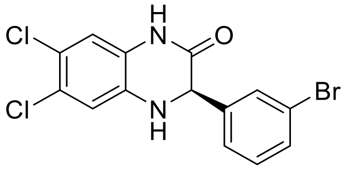

Brown solid, 33.0 mg, 74% yield. **mp** 260 °C Decomp. $[\alpha]_D^{19}$ = -65.5 (c 0.10, MeOH), 95% ee. **FTIR**ν_{max}(KBr)/cm^{-1}: 3445, 1634, 1500, 1370, 531. **^{1}H NMR** (MeOD, 400 MHz): δ 7.55 (s, 1H), 7.46 (d, 1H, J = 7.7 Hz), 7.34 (d, 1H, J = 7.7 Hz), 7.26 (t, 1H, J = 7.7 Hz), 6.89 (s, 2H), 5.01 (s, 1H). **^{13}C NMR** (MeOD, 100 MHz): δ 167.9, 143.1, 135.0, 132.3, 131.5, 131.2, 127.4, 126.9, 126.4, 123.5, 121.6, 117.3, 115.4, 60.4. **HRMS (ESI-FT ICR)** exact mass $[M+H]^+$ calculated for $C_{14}H_{10}BrCl_2N_2O$: 370.9348, found: 370.9355. HPLC analysis with Chiralpak AD-H column, 90:10 *n*-hexane:2-propanol, 1 mL/min, 220 nm; minor enantiomer t_R = 26.3 min, major enantiomer t_R = 23.7 min.

(*R*)-6,7-dichloro-3-(naphthalen-2-yl)-3,4-dihydroquinoxalin-2(1H)-one (3q)

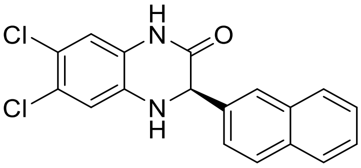

Ochre yellow solid, 31.7 mg, 77% yield. **mp** 215.2-217.1 °C. $[\alpha]_D^{21}$ = -83.1 (c 0.10, MeOH), 85% ee. **FTIR**ν_{max}(KBr)/cm^{-1}: 3460, 1675, 1650, 1510, 1373, 1308, 770. **^{1}H NMR** (MeOD, 300 MHz): δ 7.85-7.80 (m, 4H), 7.51-7.45 (m, 3H), 6.91 (d, 2H, J = 1.4 Hz), 5.18 (s, 1H). **^{13}C NMR** (MeOD, 150 MHz): δ 168.5, 138.2, 135.4, 134.7, 129.6, 129.0, 128.7, 127.43, 127.39, 127.3, 127.2, 126.5, 125.8, 121.3, 117.3, 115.3, 61.3. **HRMS (ESI-FT ICR)** exact mass $[M+Na]^+$ calculated for $C_{18}H_{12}Cl_2N_2NaO$: 365.0219, found: 365.0225. HPLC analysis with Chiralpak AD-H column, 80:20 *n*-hexane:2-propanol, 1 mL/min, 220 nm; minor enantiomer t_R = 13.6 min, major enantiomer t_R = 15.5 min.

(*R*)-4-(3-oxo-1,2,3,4-tetrahydrobenzo[g]quinoxalin-2-yl)benzonitrile (3r)

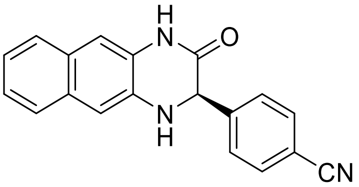

Data for this compound are consistent with those reported in the literature.[7]

Brown solid, 22.3 mg, 62% yield. **mp** 236.2-238.2 °C. $[\alpha]_D^{25}$ = -202.7 (c 0.33, $CHCl_3$), 92% ee. **FTIR**ν_{max}(KBr)/cm^{-1}: 3370, 2922, 1674, 1630, 1515, 1459, 732. **^{1}H NMR** ($CDCl_3$, 400 MHz): δ 8.97 (bs, 1H), 7.65-7.57 (m, 6H), 7.35 (t, 1H, J = 7.4 Hz), 7.29 (t, 1H, J = 7.4 Hz), 7.14 (s, 1H), 7.09 (s, 1H), 5.23 (s, 1H), 4.61 (bs, 1H). **^{13}C NMR** ($CDCl_3$, 100 MHz): δ 166.6, 143.9, 132.6, 132.1, 131.4, 128.4, 127.9, 126.9, 125.8, 125.6, 124.0, 118.4, 112.4, 112.2, 108.6, 60.3. **HRMS (ESI-FT ICR)** exact mass $[M+H]^+$ calculated for $C_{19}H_{14}N_3O$: 300.1132, found: 300.1137. HPLC analysis with Chiralpak IC column, 70:30 *n*-hexane:2-propanol, 1 mL/min, 254 nm; minor enantiomer t_R = 9.0 min, major enantiomer t_R = 11.6 min.

(*R*)-3-(4-chlorophenyl)-3,4-dihydrobenzo[g]quinoxalin-2(1H)-one (3s)

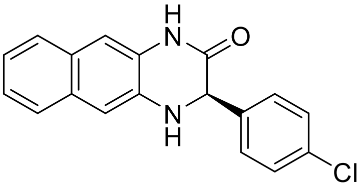

Data for this compound are consistent with those reported in the literature.[7]

Brown solid, 22.2 mg, 60% yield. **mp** 240.0-242.0 °C. $[\alpha]_D^{24}$ = -198.8 (c 0.33, $CHCl_3$), 91% ee. **FTIR**ν_{max}(KBr)/cm^{-1}: 3300, 2924, 1675, 1530, 1489, 1393, 743. **^{1}H NMR** ($CDCl_3$, 400 MHz): δ 8.20

(bs, 1H), 7.64 (d, 1H, J = 8.3 Hz), 7.60 (d, 1H, J = 8.3 Hz), 7.38 (d, 2H, J = 8.5 Hz), 7.35-7.33 (m, 1H), 7.30 (d, 2H, J = 8.5 Hz), 7.29-7.27 (m, 1H), 7.12 (s, 1H), 7.07 (s, 1H), 5.15 (s, 1H), 4.53 (bs, 1H). **^{13}C NMR** ($CDCl_3$, 150 MHz): δ 166.8, 137.3, 134.6, 132.6, 131.5, 129.1, 128.5, 128.4, 126.8, 126.1, 125.7, 125.4, 123.8, 111.7, 108.5, 60.2. **HRMS (ESI-FT ICR)** exact mass $[M+H]^+$ calculated for $C_{18}H_{14}ClN_2O$: 309.0789, found: 309.0791. HPLC analysis with Chiralpak AD-H column, 75:25 *n*-hexane:2-propanol, 1 mL/min, 254 nm; minor enantiomer t_R = 12.1 min, major enantiomer t_R = 14.5 min.

(*R*)-3-(4-bromophenyl)-1,4-dimethyl-3,4-dihydroquinoxalin-2(1H)-one (3t)

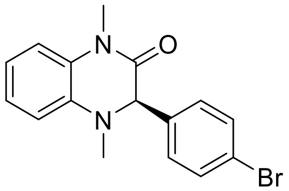

Light yellow solid, 29.0 mg, 73% yield. **mp** 109.1-111.2 °C. $[\alpha]_D^{20}$ = -50.1 (c 0.32, $CHCl_3$), 93% ee. **FTIR**ν_{max}(KBr)/cm^{-1}: 2932, 2840, 1632, 1510, 1380, 1421, 750. **^{1}H NMR** ($CDCl_3$, 300 MHz): δ 7.36 (d, 2H, J = 8.4 Hz), 7.07 (td, 1H, J = 7.8, 1.1 Hz), 7.02 (d, 2H, J = 8.4 Hz), 6.96 (d, 1H, J = 7.8 Hz), 6.88 (td, 1H, J = 7.8, 1.1 Hz), 6.69 (d, 1H, J = 7.8 Hz), 4.96 (s, 1H), 3.40 (s, 3H), 2.87 (s, 3H). **^{13}C NMR** ($CDCl_3$, 100 MHz): δ 165.1, 135.9, 135.7, 131.8, 128.6, 128.5, 124.4, 122.4, 118.7, 114.4, 111.5, 67.4, 36.0, 29.2. **HRMS (ESI-FT ICR)** exact mass $[M+H]^+$ calculated for $C_{16}H_{16}BrN_2O$: 331.0441, found: 331.0443. HPLC analysis with Chiralpak AD-H column, 70:30 *n*-hexane:2-propanol, 1 mL/min, 220 nm; minor enantiomer t_R = 9.9 min, major enantiomer t_R = 6.1 min.

(*R*)-4-(1,4-dimethyl-3-oxo-1,2,3,4-tetrahydroquinoxalin-2-yl)benzonitrile (3u)

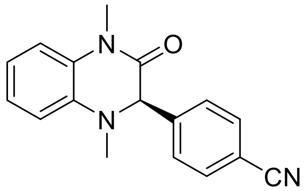

Light brown oil, 26.3 mg, 79% yield. $[\alpha]_D^{20}$ = -49.6 (c 0.33, $CHCl_3$), 96% ee. **FTIR**ν_{max}(KBr)/ cm^{-1}: 3443, 2918, 1664, 1508, 1390, 1216, 769. **^{1}H NMR** ($CDCl_3$, 400 MHz): δ 7.53 (d, 2H, J = 8.0 Hz), 7.28 (d, 2H, J = 8.0 Hz), 7.10 (t, 1H, J = 7.5 Hz), 6.97 (d, 1H, J = 7.5 Hz), 6.90 (t, 1H, J = 7.5 Hz), 6.73 (d, 1H, J = 7.5 Hz), 5.07 (s, 1H), 3.40 (s, 3H), 2.90 (s, 3H). **^{13}C NMR** ($CDCl_3$, 100 MHz): δ 164.4, 142.0, 135.6, 132.5, 128.2, 127.6, 124.6, 119.0, 118.4, 114.6, 112.2, 111.5, 67.6, 36.2, 29.3. **HRMS (ESI-FT ICR)** exact mass $[M+H]^+$ calculated for $C_{17}H_{16}N_3O$: 278.1288, found: 278.1288. HPLC analysis with Chiralpak AD-H column, 70:30 *n*-hexane:2-propanol, 1 mL/min, 220 nm; minor enantiomer t_R = 12.9 min, major enantiomer t_R = 7.5 min.

(*R*)-3-phenyl-3,4-dihydro-2H-benzo[b][1,4]oxazin-2-one (3v)

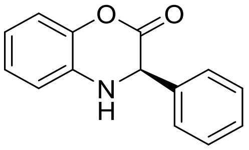

Data for this compound are consistent with those reported in the literature.[9]

White solid, 16.2 mg, 60% yield. **mp** 87.5-89.9 °C. $[\alpha]_D^{18}$ = -108.6 (c 0.41, $CHCl_3$), 89% ee. **FTIR**ν_{max}(KBr)/cm^{-1}: 3445, 1761, 1637, 1500, 1296, 1193, 746. **^{1}H NMR** ($CDCl_3$, 600 MHz): δ 7.42-7.36 (m, 5H), 7.06-7.02 (m, 2H), 6.87 (td, 1H, *J* = 7.8, 1.3 Hz), 6.82 (dd, 1H, *J* = 7.8, 1.3 Hz), 5.07 (d, 1H, *J* = 1.7 Hz), 4.25 (bs, 1H). **^{13}C NMR** ($CDCl_3$, 150 MHz): δ 165.1, 140.69, 136.3, 132.3, 129.0, 127.5, 125.2, 120.4, 117.0, 114.8, 59.3. **HRMS (ESI-FT ICR)** exact mass $[M+Na]^+$ calculated for $C_{14}H_{11}NNaO_2$: 248.0682, found: 248.0687. HPLC analysis with Chiralcel OD-H column, 80:20 *n*-hexane:2-propanol, 0.6 mL/min, 220 nm; minor enantiomer t_R = 25.6 min, major enantiomer t_R = 18.3 min.

(*R*)-2-phenyl-2H-benzo[b][1,4]thiazin-3(4H)-one (3w)

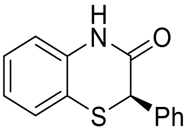

Data for this compound are consistent with those reported in the literature.[10]

Light yellow solid, 18.8 mg, 65% yield. **mp** 194.2-197.0 °C. $[\alpha]_D^{16}$ = +192.0 (c 0.35, $CHCl_3$), 90% ee. **FTIR**ν_{max}(KBr)/cm^{-1}: 3448, 2917, 1674, 1584, 1478, 1368, 749, 695. **^{1}H NMR** ($CDCl_3$, 400 MHz): δ 8.42 (bs, 1H), 7.38-7.37 (m, 2H), 7.33-7.28 (m, 4H), 7.16 (t, 1H, *J* = 7.5 Hz), 7.01 (t, 1H, *J* = 7.5 Hz), 6.84 (d, 1H, *J* = 7.5 Hz), 4.70 (s, 1H). **^{13}C NMR** ($CDCl_3$, 100 MHz): δ 166.6, 135.9, 134.8, 128.7, 128.2, 127.91, 127.87, 127.3, 124.0, 119.3, 117.0, 46.3. **HRMS (ESI-FT ICR)** exact mass $[M+Na]^+$ calculated for $C_{14}H_{11}NNaOS$: 264.0454, found: 264.0453. HPLC analysis with Chiralpak IC column, 80:20 *n*-hexane:2-propanol, 1 mL/min, 254 nm; minor enantiomer t_R = 8.6 min, major enantiomer t_R = 7.4 min.

(*R*)-3-heptyl-3,4-dihydroquinoxalin-2(1H)-one (3x)

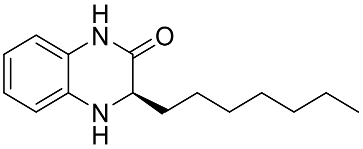

[9] Xue, Z.-Y.; Jiang, Y.; Peng, X.-Z.; Yuan, W.-C.; Zhang, X.-M.; *Adv. Synth. Catal.* **2010**, 352, 2132.

[10] (a) Kamila, S.; Koh, B.; Khan, O.; Zhang, H.; Biehl, E. R. *J. Heterocycl. Chem.* **2006**, *43*, 1641; (b) Lee Y. M.; Park, Y. S.; *Heterocycles* **2009**, *78*, 2233.

Ochre yellow solid, 17.7 mg, 60% yield. **mp** 54.0-55.9 °C $[\alpha]_D^{17}$ = -15.6 (c 0.35, $CHCl_3$), 92% ee. **FTIR**ν_{max}(KBr)/cm^{-1}: 2920, 2851, 1675, 1507, 1466, 1378, 770. **^{1}H NMR** ($CDCl_3$, 400 MHz): δ 8.26-8.23 (bs, 1H), 6.89 (t, 1H, J = 7.5 Hz), 6.77-6.67 (m, 3H), 3.94 (bs, 1H), 3.90 (dd, 1H, J = 8.0, 4.9 Hz), 1.84-1.71 (m, 2H), 1.44-1.42 (m, 2H), 1.30-1.26 (m, 6H), 0.88-0.85 (m, 5H). **^{13}C NMR** ($CDCl_3$, 100 MHz): δ 168.9, 133.0, 125.2, 123.8, 119.3, 115.2, 114.1, 56.4, 31.8, 31.7, 29.3, 29.1, 25.3, 22.6, 14.1. **HRMS (ESI-FT ICR)** exact mass $[M+H]^+$ calculated for $C_{15}H_{23}N_2O$: 247.1805, found: 247.1809. HPLC analysis with Chiralpak AD-H column, 80:20 *n*-hexane:2-propanol, 0.8 mL/min, 220 nm; minor enantiomer t_R = 8.2 min, major enantiomer t_R = 9.0 min.

(*R*)-3-isobutyl-3,4-dihydroquinoxalin-2(1H)-one (3y)

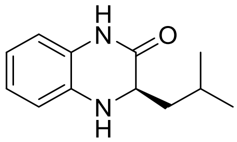

Data for this compound are consistent with those reported in the literature.[11]

Yellow oil, 17.2 mg, 70% yield. $[\alpha]_D^{27}$ = -39.6 (c 0.75, MeOH), 87% ee. **FTIR**ν_{max}(KBr)/cm^{-1}: 3400, 2953, 1661, 1645, 751. **^{1}H NMR** ($CDCl_3$, 400 MHz): δ 8.96 (bs, 1H), 6.91-6.86 (m, 1H), 6.77-6.76 (m, 2H), 6.68 (d, 1H, J = 7.8 Hz), 3.97-3.93 (m, 2H), 1.82-1.59 (m, 3H), 0.98 (d, 3H, J = 6.4 Hz), 0.96 (d, 3H, J = 6.4 Hz). **^{13}C NMR** ($CDCl_3$, 100 MHz): δ 169.5, 132.8, 125.5, 123.8, 119.4, 115.4, 114.2, 54.5, 40.2, 24.2, 23.3, 21.5. **HRMS (MALDI-FT ICR)** exact mass $[M+H]^+$ calculated for $C_{12}H_{17}N_2O$: 205.1335, found: 205.1361. HPLC analysis with Chiralpak IC column, 70:30 *n*-hexane:2-propanol, 1 mL/min, 220 nm; minor enantiomer t_R = 5.9 min, major enantiomer t_R = 6.9 min.

(*R*)-3-isobutyl-1,4-dimethyl-3,4-dihydroquinoxalin-2(1H)-one (3z)

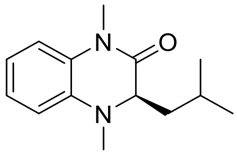

Yellow rubber, 17.2 mg, 85% yield. $[\alpha]_D^{28}$ = -94.7 (c 0.22, $CHCl_3$), 90% ee. **FTIR**ν_{max}(KBr)/cm^{-1}: 3400, 2950, 1660, 1646, 748 . **^{1}H NMR** ($CDCl_3$, 400 MHz): δ 7.02 (t, 1H, J = 7.7 Hz), 6.92 (d, 1H, J = 7.7 Hz), 6.85 (t, 1H, J = 7.7 Hz), 6.64 (d, 1H, J = 7.7 Hz), 3.92 (dd, 1H, J = J = 7.2 Hz), 3.37 (s, 3H), 2.91 (s, 3H), 1.68-1.63 (m, 1H), 1.32-1.28 (m, 2H), 0.95 (d, 3H, J = 6.6 Hz), 0.86 (d, 3H, J = 6.6 Hz). **^{13}C NMR** ($CDCl_3$, 100 MHz): δ 167.1, 136.3, 129.6, 123.8, 118.5, 114.2, 112.3, 62.7, 36.23, 36.18, 29.0, 24.9, 23.1, 22.1. **HRMS (MALDI-FT ICR)** exact mass $[M+H]^+$ calculated for $C_{14}H_{21}N_2O$: 233.1648, found: 233.1651. HPLC analysis with Chiralpak ADH column, 90:10 *n*-

[11] Li, D.; Ollevier, T. *Eur. J. Org. Chem.* **2019**, 1273.

hexane:2-propanol, 1 mL/min, 220 nm; minor enantiomer t_R = 6.5 min, major enantiomer t_R = 6.0 min.

(*R*)-3-cyclohexyl-6,7-dimethyl-3,4-dihydroquinoxalin-2(1H)-one (3ab)

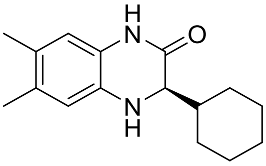

Brown solid, 15.5 mg, 50% yield. **mp** 220 °C Decomp. $[\alpha]_D^{20}$ = -7.5 (c 0.34, $CHCl_3$), 93% ee. **FTIR**ν_{max}(KBr)/cm^{-1}: 3438, 2920, 2851, 1659, 591. **^{1}H NMR** ($CDCl_3$, 300 MHz): δ 7.92 (bs, 1H), 6.46 (s, 2H), 3.89 (bs, 1H), 3.68 (d, 1H, *J* = 5.7 Hz), 2.15 (s, 3H), 2.14 (s, 3H), 1.72-1.62 (m, 4H), 1.26-1.11 (m, 6H), 0.88-0.83 (m, 1H). **^{13}C NMR** ($CDCl_3$, 75 MHz): δ 167.6, 131.8, 130.8, 126.7, 122.6, 116.2, 115.1, 61.8, 40.1, 29.5, 27.8, 26.1, 26.0, 25.8, 19.3, 18.8. **HRMS (ESI-FT ICR)** exact mass $[M+H]^+$ calculated for $C_{16}H_{23}N_2O$: 259.1805, found: 259.1806. HPLC analysis with Chiralcel OD-H column, 70:30 *n*-hexane:2-propanol, 1 mL/min, 220 nm; minor enantiomer t_R = 5.7 min, major enantiomer t_R = 7.7 min.

(*R*)-3-(pent-4-en-1-yl)-3,4-dihydrobenzo[g]quinoxalin-2(1H)-one (3ac)

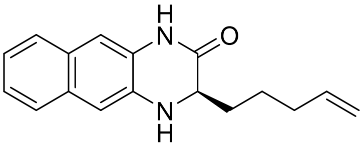

Yellow solid, 16.0 mg, 50% yield. **mp** 129.3-131.4 °C $[\alpha]_D^{26}$ = -30.0 (c 0.34, $CHCl_3$), 93% ee. **FTIR**ν_{max}(KBr)/cm^{-1}: 3400, 2927, 2849, 1664, 1634, 744. **^{1}H NMR** ($CDCl_3$, 250 MHz): δ 8.58 (bs, 1H), 7.64-7.56 (m, 2H), 7.34-7.21 (m, 2H), 7.11 (s, 1H), 7.01 (s, 1H), 5.87-5.70 (m, 1H), 5.05-4.95 (m, 2H), 4.22 (s, 1H), 4.02 (dd, 1H, *J* = *J* = 5.7 Hz), 2.10 (q, 2H, *J* = 7.0 Hz), 1.92-1.76 (m, 1H), 1.64-1.53 (m, 3H). **^{13}C NMR** ($CDCl_3$, 62.5 MHz): δ 169.3, 137.9, 133.0, 131.3, 128.3, 126.7, 125.6, 125.1, 123.5, 115.2, 111.3, 108.6, 56.4, 33.3, 32.0, 24.5. **HRMS (MALDI-FT ICR)** exact mass $[M+H]^+$ calculated for $C_{17}H_{19}N_2O$: 267.1492, found: 267.1531. HPLC analysis with Chiralpak IC column, 80:20 *n*-hexane:2-propanol, 1 mL/min, 220 nm; minor enantiomer t_R = 16.4 min, major enantiomer t_R = 9.6 min.

(*R*)-3-(but-3-yn-1-yl)-3,4-dihydroquinoxalin-2(1H)-one (3ad)

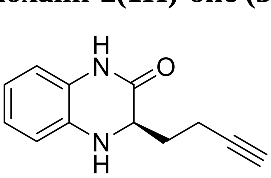

Yellow wax, 15.1 mg, 63% yield. $[\alpha]_D^{26}$ = +8.7 (c 0.53, $CHCl_3$), 96% ee. **FTIR**ν_{max}(KBr)/cm^{-1}: 3400, 2919, 2853, 1671, 1604, 1502, 1377, 1302, 752. **^{1}H NMR** ($CDCl_3$, 300 MHz): δ 7.76 (bs, 1H), 6.91 (t, 1H, J = 7.5 Hz), 6.77 (t, 1H, J = 7.5 Hz), 6.72-6.68 (m, 2H), 4.20 (bs, 1H), 4.09 (dd, 1H, *J* = 7.0,

4.5, 1.4 Hz), 2.45 – 2.40 (m, 2H), 2.11 (dtd, 1H, *J* = 11.4, 7.0, 4.5 Hz,), 2.05 (t, 1H, *J* = 2.6 Hz), 1.97 – 1.89 (m, 1H). **^{13}C NMR** ($CDCl_3$, 100 MHz): δ 168.3, 132.8, 125.2, 124.0, 119.6, 115.4, 114.4, 83.0, 69.9, 55.6, 31.6, 15.0. **HRMS (MALDI-FT ICR)** exact mass $[M+H]^+$ calculated for $C_{12}H_{13}N_2O$: 201.1022, found: 201.1026. HPLC analysis with Chiralpak ADH column, 80:20 *n*-hexane:2-propanol, 1 mL/min, 220 nm; minor enantiomer t_R = 10.0 min, major enantiomer t_R = 11.1 min.

Synthesis of products 3 by reacting a non-symmetrical *ortho*-phenylenediamine

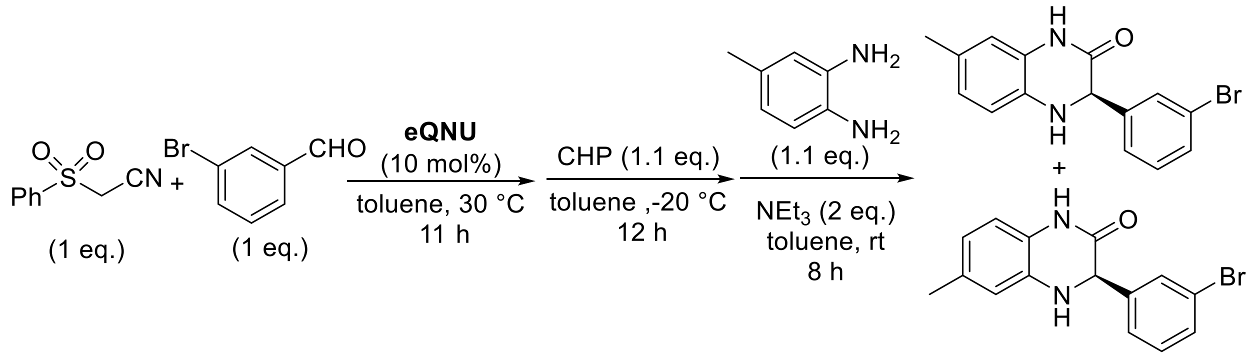

In a sample vial containing (phenylsulfonyl)acetonitrile (22.2 mg, 0.12 mmol) and **eQNU** (6.9 mg, 0.012 mmol) in anhydrous toluene (0.4 mL), 3-Bromobenzaldehyde (14 µL,0.12 mmol) was added. The reaction was stirred at 30 °C for 11 hours, monitored by TLC (eluent PE/ethyl acetate 8/2). After completion, toluene (5.6 mL) was added. Then 1.1 equivalents of CHP (tech. 80%, 24 µL, 0.132 mmol) was added at –20 °C and the solution was stirred at -20 °C for 12 hours. Then 3,4-Diaminotoluene (16.6 mg, 0.132 mmol) and triethylamine (33 µL, 0.24 mmol) were added and the reaction mixture was stirred for 8 hours at room temperature, monitored by TLC (eluent PE/ethyl acetate 8/2) to check the conversion of epoxide into the product. After completion, the mixture was diluted with ethyl acetate and water and HCl 1N was added to pH 7. The aqueous layer was extracted with ethyl acetate (3x20 mL). The combined organic layers were washed with saturated NaCl solution (1x40 mL), dried over anhydrous Na_2SO_4 and concentrated under reduced pressure. ^{1}H-NMR of the crude reaction mixture showed a conversion of 90% of the (phenylsulfonyl)acetonitrile and the presence of a 50/50 mixture of an inseparable mixture of the regioisomeric heterocycles.

General procedure for the reduction of 3 to products 4

Reduction of products **3** was carried out according to the literature.[12]

Compound **3** (0.1 mmol) was dissolved in THF (10 mL) and the solution was cooled to 0 °C. Then 13 eq. of borane-tetrahydrofuran complex solution (1.3 mL, 1M in THF) was added dropwise. The mixture was warmed up and refluxed at 70 °C until consumption of **3** (monitored by TLC, eluent PE/ ethyl acetate 9/1). The reaction was then cooled to 0 °C, quenched by the slow addition of 10% aqueous NaOH and extracted with ethyl acetate (3x20 mL). The combined organic phases were dried

[12] Huang, R.; Chen, X.; Mou, C.; Luo, G.; Li, Y.; Li, X.; Xue, W.; Jin, Z.; Chi, Y. R. *Org. Lett.* **2019**, *21*, 4340.

over Na_2SO_4 and concentrated under reduced pressure. The crude product was purified by flash chromatography (eluent: PE/ethyl acetate 100/0 to 90/10) to give products **4** in 65-90% yield and 88-90% ee.

(*R*)-2-phenyl-1,2,3,4-tetrahydroquinoxaline (4a)

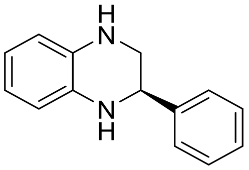

Data for this compound are consistent with those reported in the literature.[13]

Yellow oil, 15.8 mg, 75% yield. $[\alpha]_D^{21}$ = -54.5 (c 0.30, $CHCl_3$), 90% ee. **FTIR**ν_{max}(KBr)/cm^{-1}: 3380, 3357, 3061, 3030, 2917, 2850, 1949, 1869, 1595. **^{1}H NMR** ($CDCl_3$, 400 MHz): δ 7.41-7.32 (m, 5H), 6.66-6.57 (m, 4H), 4.49 (dd, 1H, *J* = 8.2, 3.0 Hz), 3.88 (bs, 2H), 3.47 (dd, 1H, *J* = 11.0, 3.0 Hz), 3.33 (dd, 1H, *J* = 11.0, 8.2 Hz). **^{13}C NMR** ($CDCl_3$, 75 MHz): δ 141.8, 134.1, 132.8, 128.6, 127.9, 127.0, 118.9, 118.8, 114.7, 114.4, 54.7, 49.1. **HRMS (ESI-FT ICR)** exact mass $[M+H]^+$ calculated for $C_{14}H_{15}N_2$: 211.1230, found: 211.1228. HPLC analysis with Chiralcel OD-H column, 80:20 *n*-hexane:2-propanol, 1 mL/min, 220 nm; minor enantiomer t_R = 24.8 min, major enantiomer t_R = 16.1 min.

(*R*)-2-(p-tolyl)-1,2,3,4-tetrahydroquinoxaline (4b)

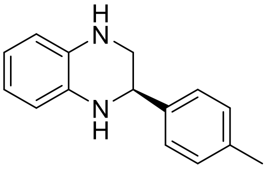

Data for this compound are consistent with those reported in the literature.[13]

Light yellow solid, 14.6 mg, 65% yield. **mp** 104.9-107.4 °C. $[\alpha]_D^{22}$ = -43.0 (c 0.71, $CHCl_3$), 88% ee. **FTIR**ν_{max}(KBr)/cm^{-1}: 3356, 2916, 2849, 1594, 1505, 1451, 1338, 1300, 1106, 813, 736. **^{1}H NMR** ($CDCl_3$, 400 MHz): δ 7.29 (d, 2H, *J* = 8.0 Hz), 7.19 (d, 2H, *J* = 8.0 Hz), 6.66-6.57 (m, 4H), 4.45 (dd, 1H, *J* = 8.2, 3.0 Hz), 3.86 (bs, 2H), 3.44 (dd, 1H, *J* = 11.0, 3.0 Hz), 3.32 (dd, 1H, *J* = 11.0, 8.2 Hz), 2.37 (s, 3H). **^{13}C NMR** ($CDCl_3$, 100 MHz): δ 138.9, 137.6, 134.2, 132.8, 129.3, 126.9, 118.8, 118.7, 114.7, 114.4, 54.4, 49.2, 21.1. **HRMS (ESI-FT ICR)** exact mass $[M+H]^+$ calculated for $C_{15}H_{17}N_2$: 225.1386, found: 225.1387. HPLC analysis with Chiralcel OD-H column, 80:20 *n*-hexane:2-propanol, 1 mL/min, 220 nm; minor enantiomer t_R = 17.4 min, major enantiomer t_R = 11.3 min.

[13] Rueping, M.; Tato, F.; Schoepke, F. R. *Chem. Eur. J.* **2010**, *16*, 2688.

(*R*)-2-(4-bromophenyl)-1,4-dimethyl-1,2,3,4-tetrahydroquinoxaline (4c)

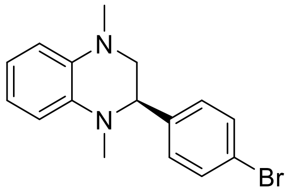

Light yellow solid, 23.8 mg, 75% yield. **mp** 60.3-61.9 °C. $[\alpha]_D^{21}$ = 80.6 (c 0.33, $CHCl_3$), 90% ee. **FTIR**ν_{max}(KBr)/cm^{-1}: 3254, 2851, 1594, 1509, 1484, 1301, 1260, 1209, 1010, 829, 734. **^{1}H NMR** ($CDCl_3$, 400 MHz): δ 7.44 (d, 2H, *J* = 8.4 Hz), 7.13 (d, 2H, *J* = 8.4 Hz), 6.80 (td, 1H, *J* = 7.7, 1.4 Hz), 6.73 (td, 1H, *J* = 7.7, 1.4 Hz), 6.63 (dd, 1H, *J* = 7.7, 1.4 Hz) overlapped with 6.61 (dd, 1H, *J* = 7.7, 1.4 Hz), 4.44 (t, 1H, *J* = 4.3 Hz), 3.37 (dd, 1H, *J* = 11.1, 4.3 Hz), 3.09 (dd, 1H, *J* = 11.1, 4.3 Hz), 2.79 (s, 3H), 2.78 (s, 3H). **^{13}C NMR** ($CDCl_3$, 100 MHz): δ 141.5, 136.7, 136.4, 131.5, 128.8, 121.1, 119.2, 117.3, 111.0, 110.1, 62.5, 56.2, 39.3, 37.2. **HRMS (ESI-FT ICR)** exact mass $[M+H]^+$ calculated for $C_{16}H_{18}BrN_2$: 317.0648, found: 317.0651. HPLC analysis with Chiralcel OD-H column, 95:5 *n*-hexane:2-propanol, 1 mL/min, 254 nm; minor enantiomer t_R = 8.8 min, major enantiomer t_R = 9.4 min.

Scale-up of the one-pot asymmetric Knoevenagel/epoxidation/ring-opening reaction to heterocycle 3k with catalyst recycling

In a round bottomed flask containing a solution of (phenylsulfonyl)acetonitrile (184.9 mg, 1.0 mmol) and **eQNU** (59.6 mg, 0.10 mmol) in anhydrous toluene (3.3 mL), 3-bromobenzaldehyde (119 μL, 1 mmol) was added. The reaction was stirred at 30 °C for 17 hours, monitored by TLC (eluent PE/ethyl acetate 80/20). After completion, the solution was diluted with toluene (46.7 mL) and 1.1 equivalents of cumene hydroperoxide (tech. 80%, 203 μL, 1.1 mmol) were added at – 20 °C. The mixture was stirred at -20 °C for 9 hours, monitored by TLC (eluent PE/ethyl acetate 80/20). Then 4,5-dimethyl-*o*-phenylenediamine (152.9 mg, 1.1 mmol) and triethylamine (279 μL, 2 mmol, 2 eq.) were added and the reaction mixture was stirred at room temperature for 14 hours, monitored by TLC (eluent PE/ethyl acetate 8/2 to check if the epoxide has been consumed). After completion, the mixture was diluted with ethyl acetate and water adding HCl 1N to pH 7. The aqueous layer was extracted with ethyl acetate (3x50 mL). The combined organic layers were washed with saturated NaCl solution (1x80 mL), dried over anhydrous Na_2SO_4 and concentrated under reduced pressure. The reaction mixture was purified by flash chromatography (eluent: PE/ethyl acetate 90/10 to 70/30 for product **3k**; ethyl acetate/MeOH 100/0 to 95/5 for **eQNU**) to give enantioenriched product **3k** in 72% yield (238.5 mg, 0.72 mmol) and 99% ee, while catalyst was recovered in quantitative yield. The recovered **eQNU** was reused 3 times under the same conditions without observing any reduction of the catalytic activity (see Scheme 4 in the manuscript).

Mechanistic investigations

Experiment 1

Table S3. Monitoring of epoxide ring opening reaction over time in toluene-d8 at rt.

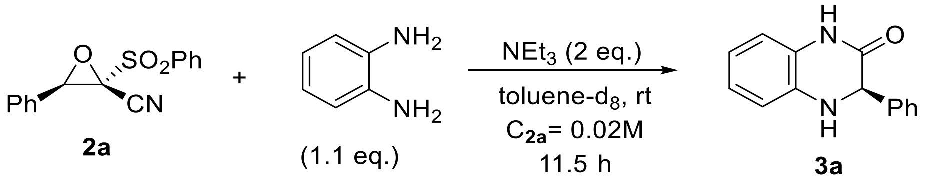

Experimental procedure: An NMR tube was charged with epoxide **2a** (5.7 mg, 0.02 mmol), *o*-phenylenediamine (2.4 mg, 0.022 mmol), toluene-d8 (1 mL) and triethylamine (6 µL, 0.04 mmol). The reaction mixture was analyzed by ^{1}H NMR at 600 MHz over 11.5 hours (see below).

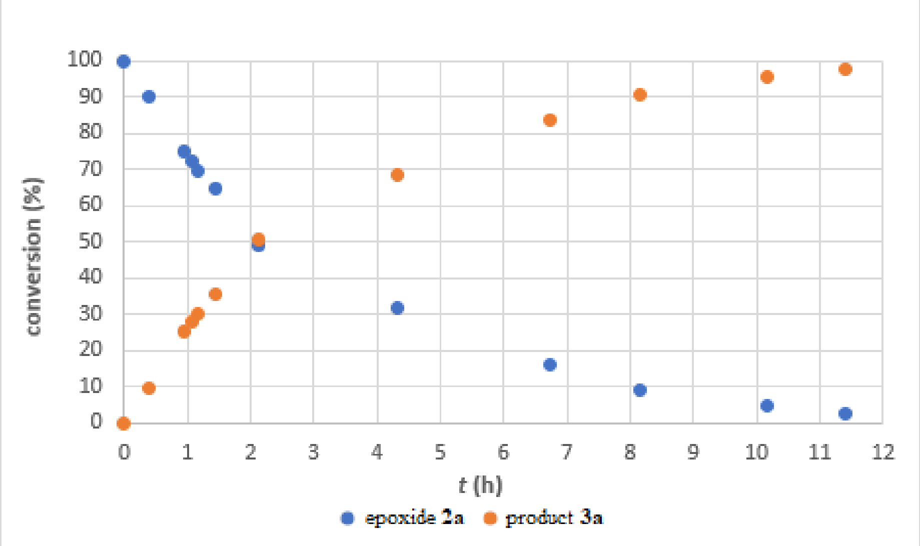

Entry	*t* [h]	**2a** [%]	Conversion **3a** [%]
1	0	100	0
2	0.38	90.3	9.7
3	0.96	75	25
4	1.06	72.2	27.8
5	1.15	69.7	30.3
6	1.44	64.6	35.4
7	2.11	49.1	50.9
8	4.32	31.5	68.5
9	6.72	16.3	83.7
10	8.16	9.14	90.86
11	10.18	4.6	95.4
12	11.4	2.3	97.7

Experiment 2: HRMS-ESI spectrum

Figure S1. High resolution mass spectrometry analysis of the crude reaction mixture of racemic epoxide **2a** with *o*-phenylenediamine in toluene at room temperature after 0.5 hours.

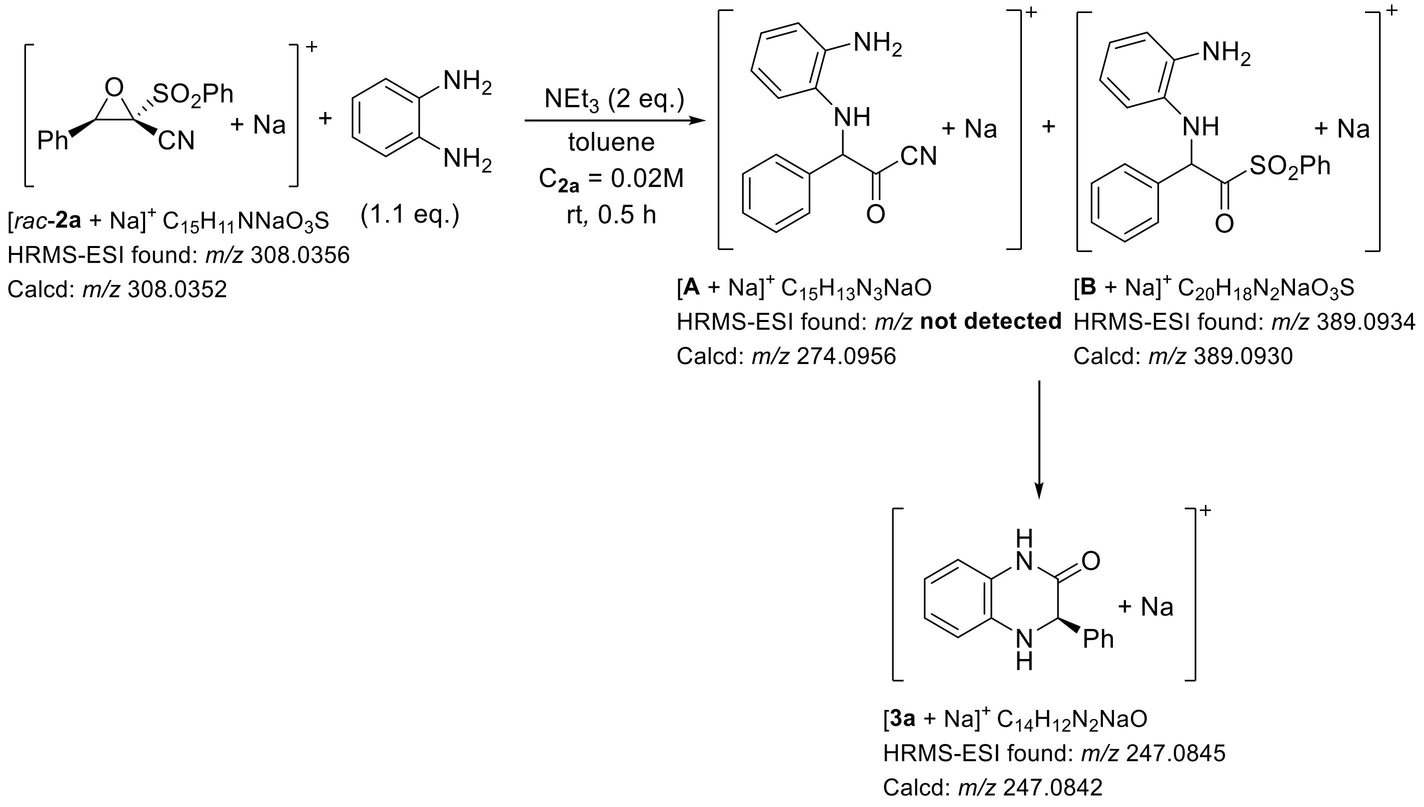

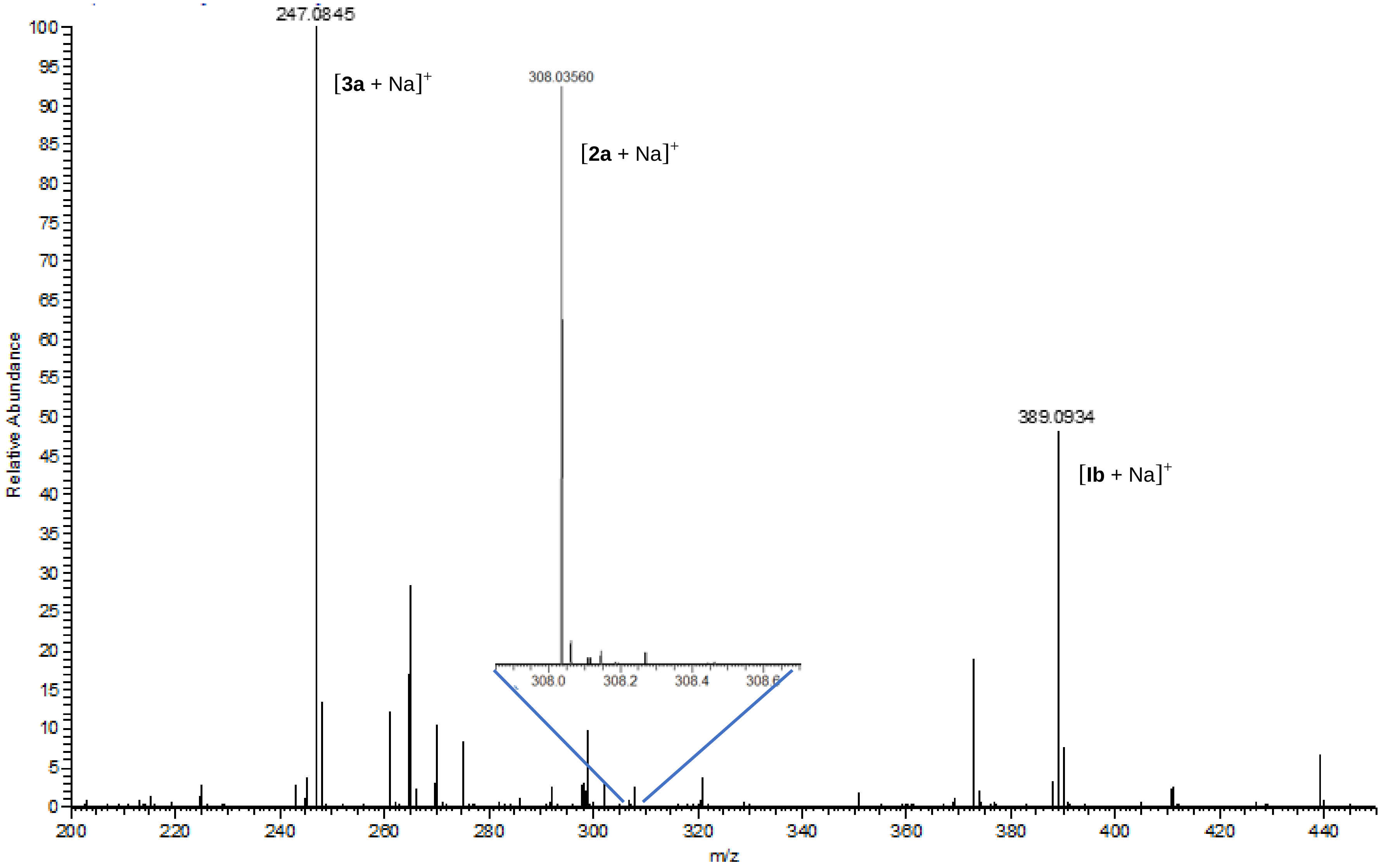

247.0845
[3a + Na]+
308.03560
[2a + Na]+
389.0934
[Ib + Na]+
Relative Abundance
m/z
308.0
308.2
308.4
308.6

NMR spectra

^{1}H NMR in $CDCl_3$ (600 MHz)

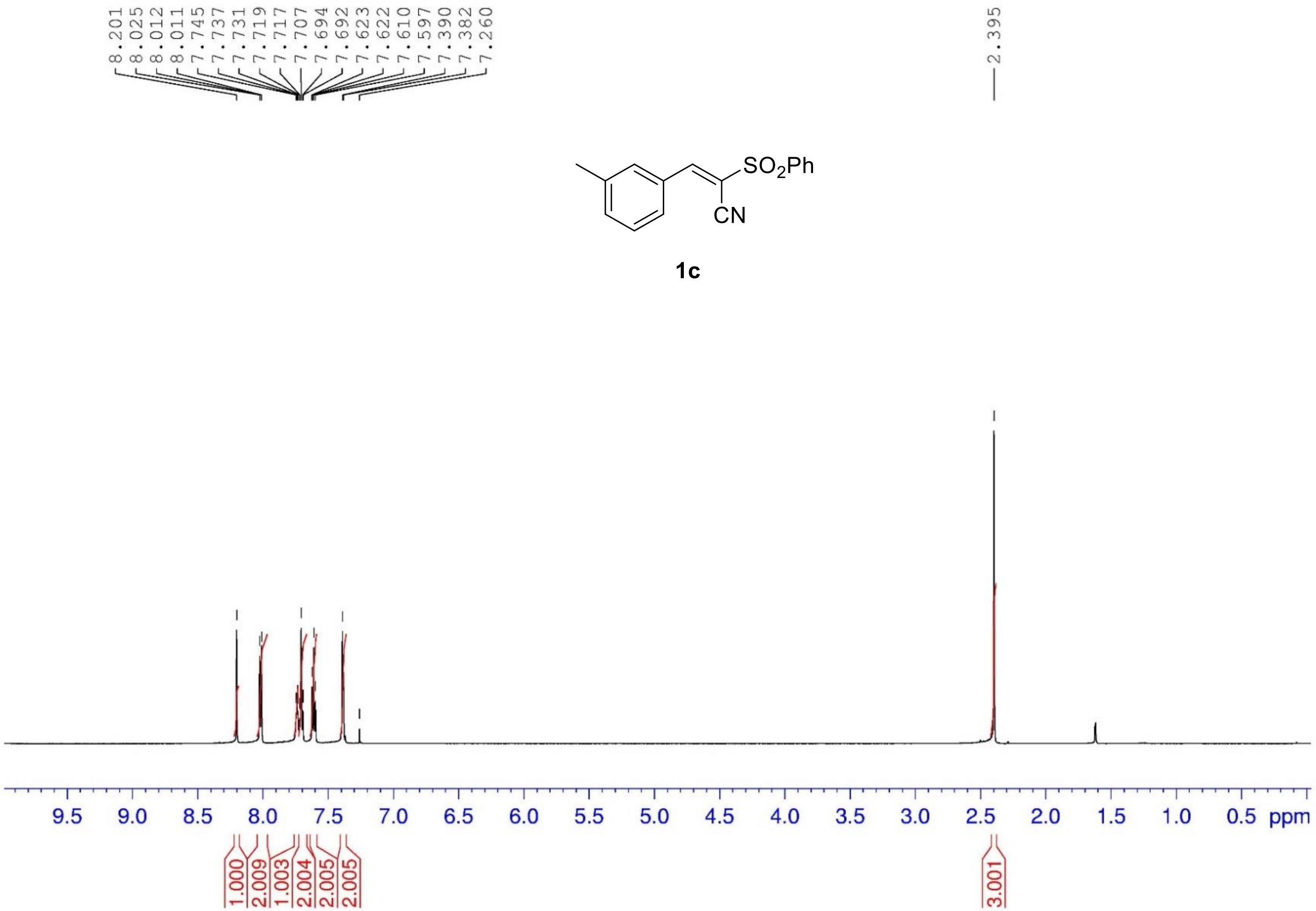

^{13}C NMR in $CDCl_3$ (150 MHz)

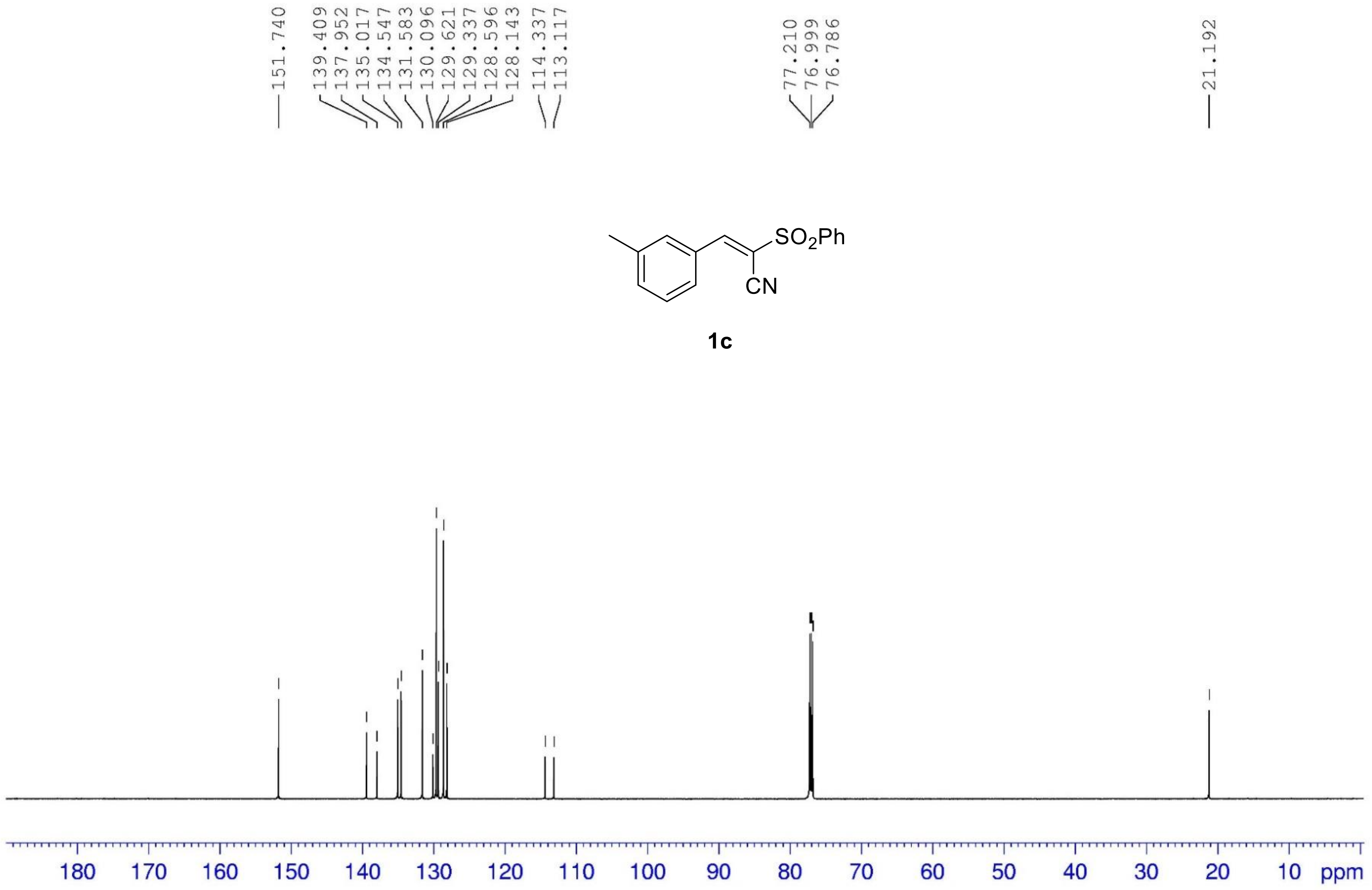

^{1}H NMR in $CDCl_3$ (600 MHz)

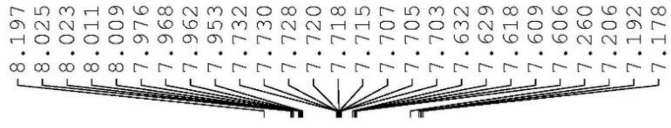

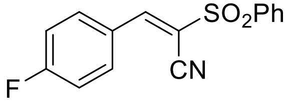

1f

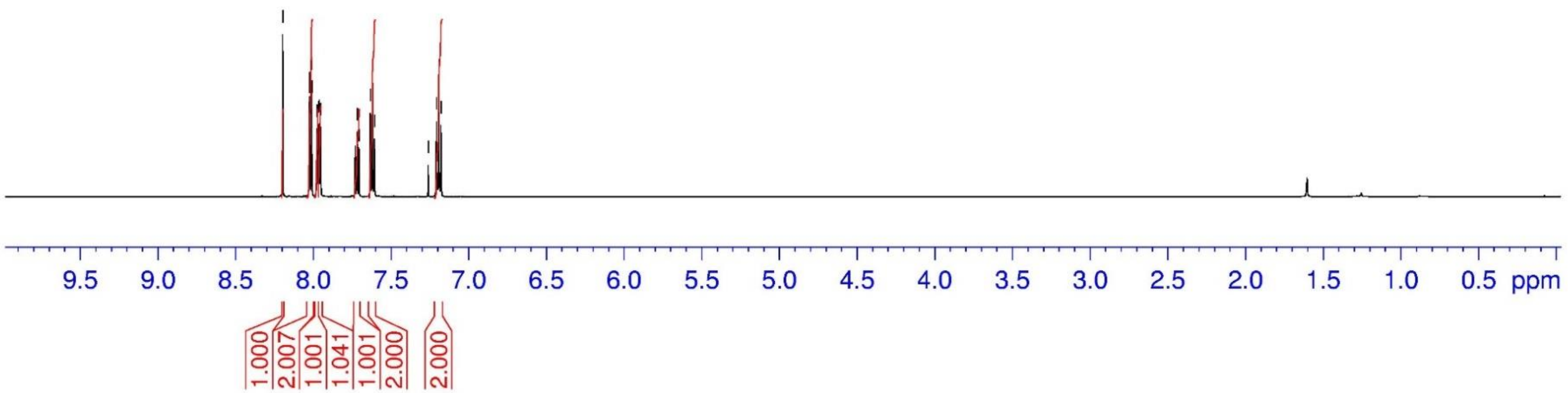

^{13}C NMR in $CDCl_3$ (150 MHz)

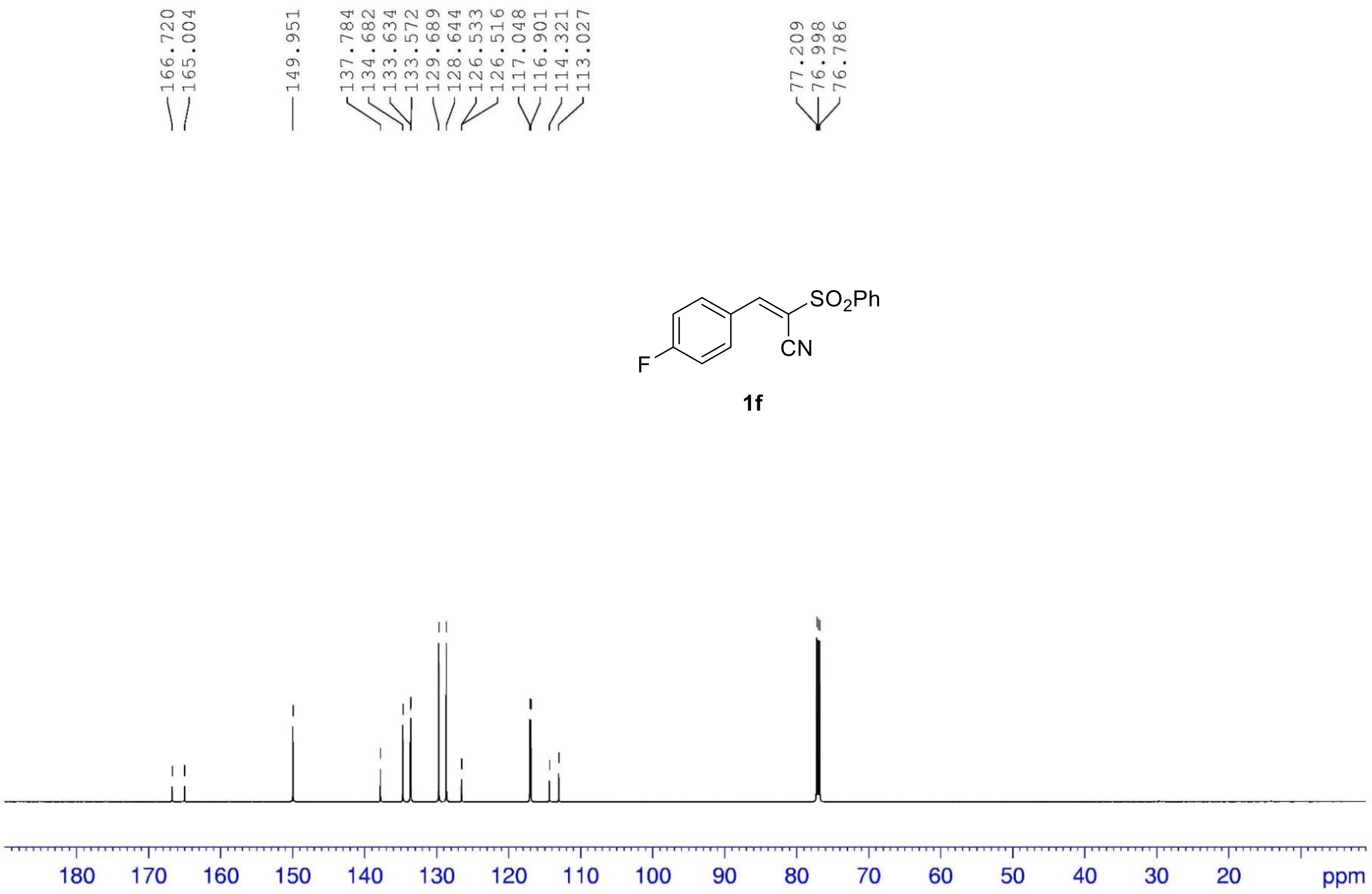

^{1}H NMR in $CDCl_3$ (400 MHz)

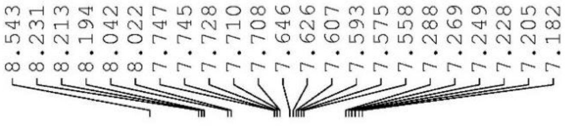

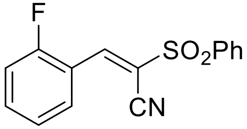

1g

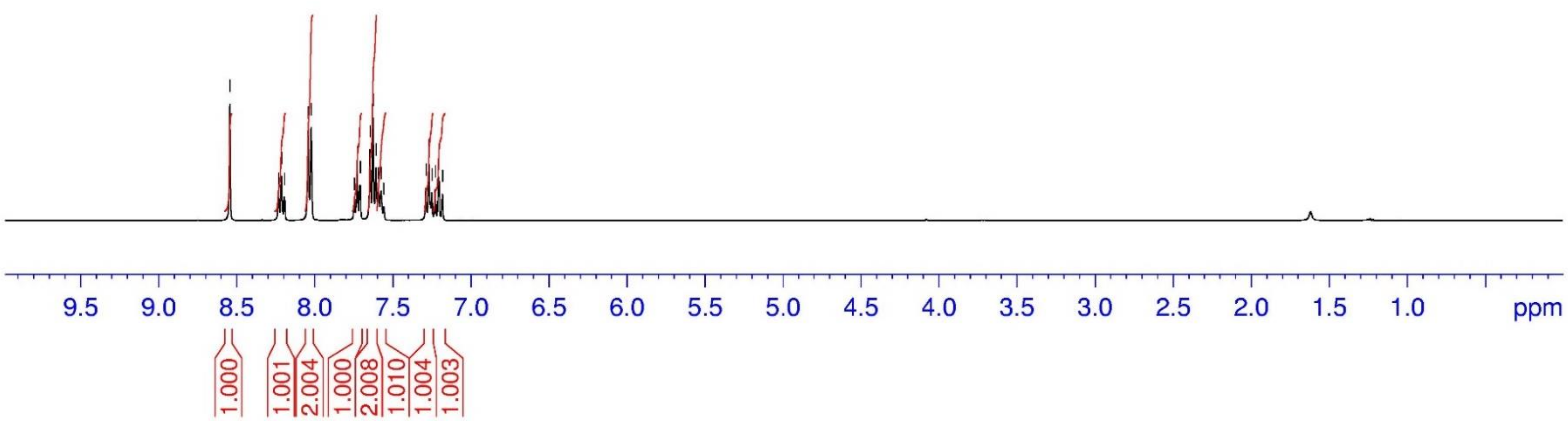

^{13}C NMR in $CDCl_3$ (100 MHz)

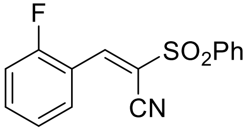

1g

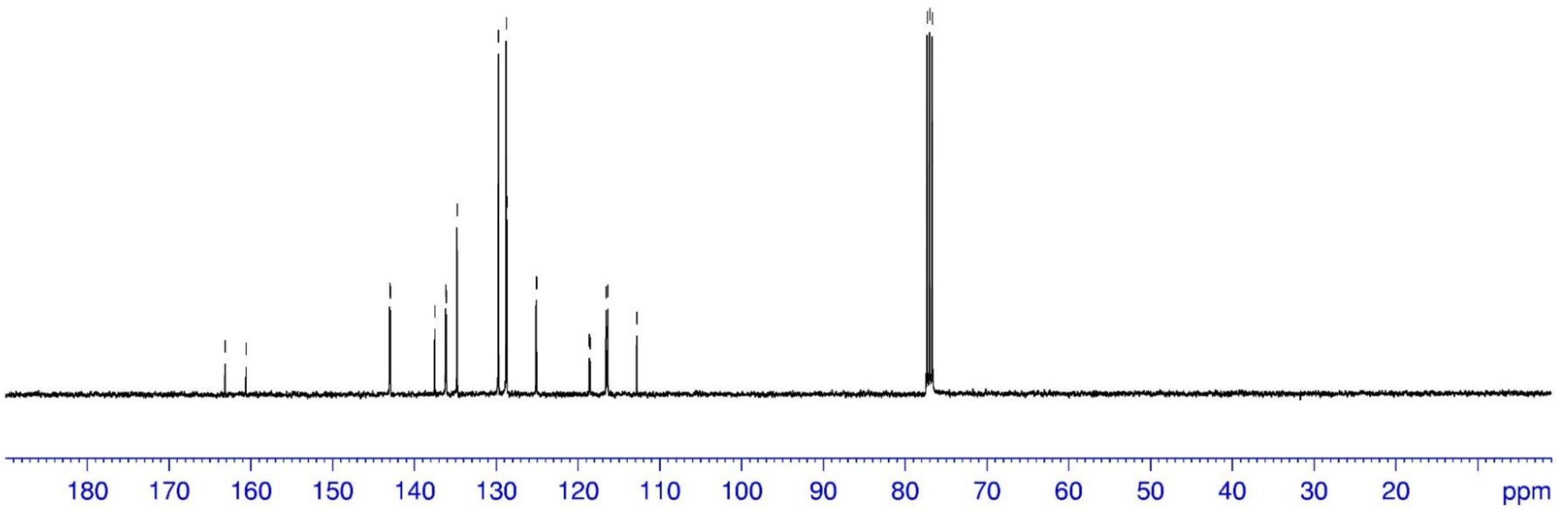

^1H NMR in $CDCl_3$ (600 MHz)

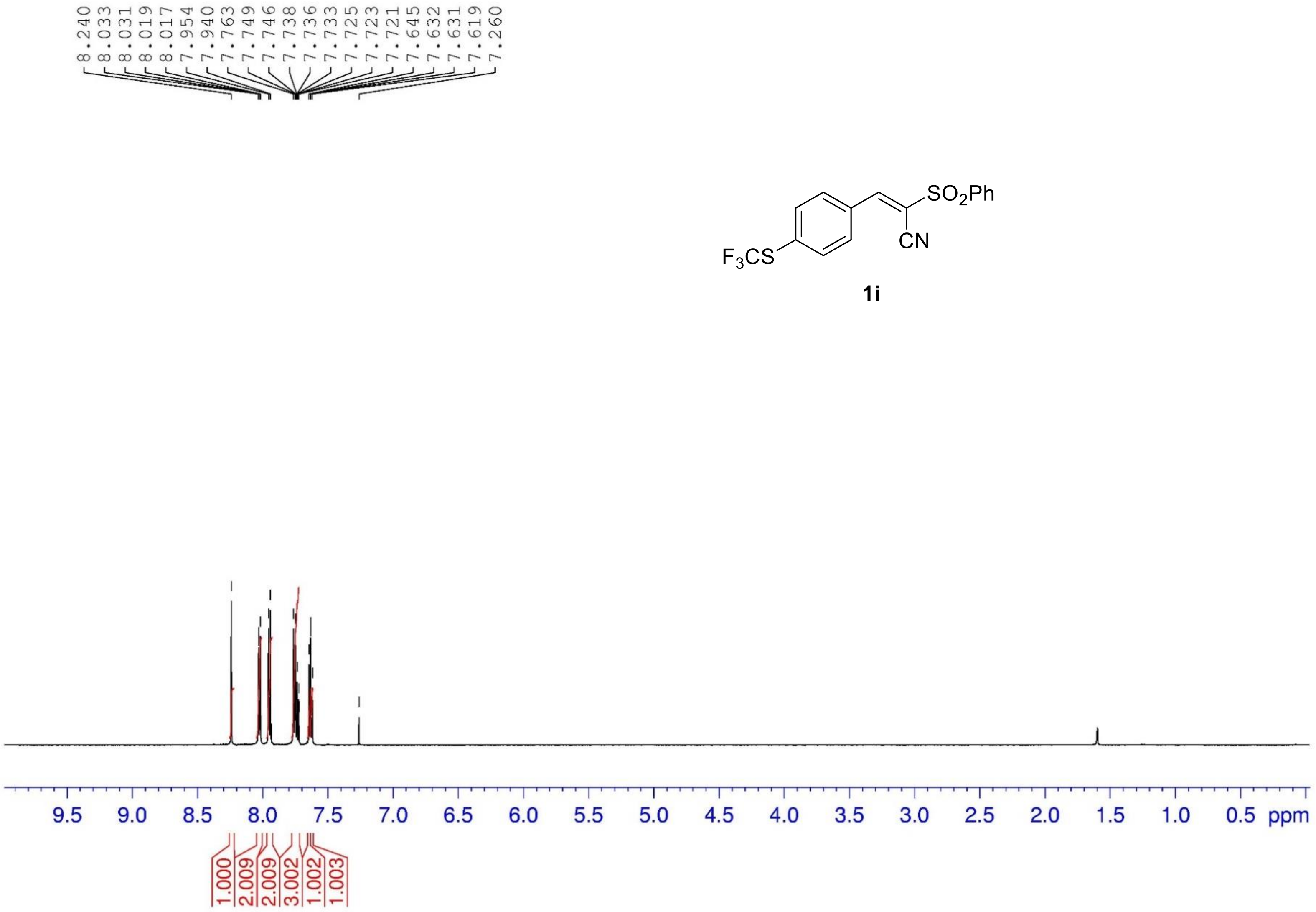

^{13}C NMR in $CDCl_3$ (150 MHz)

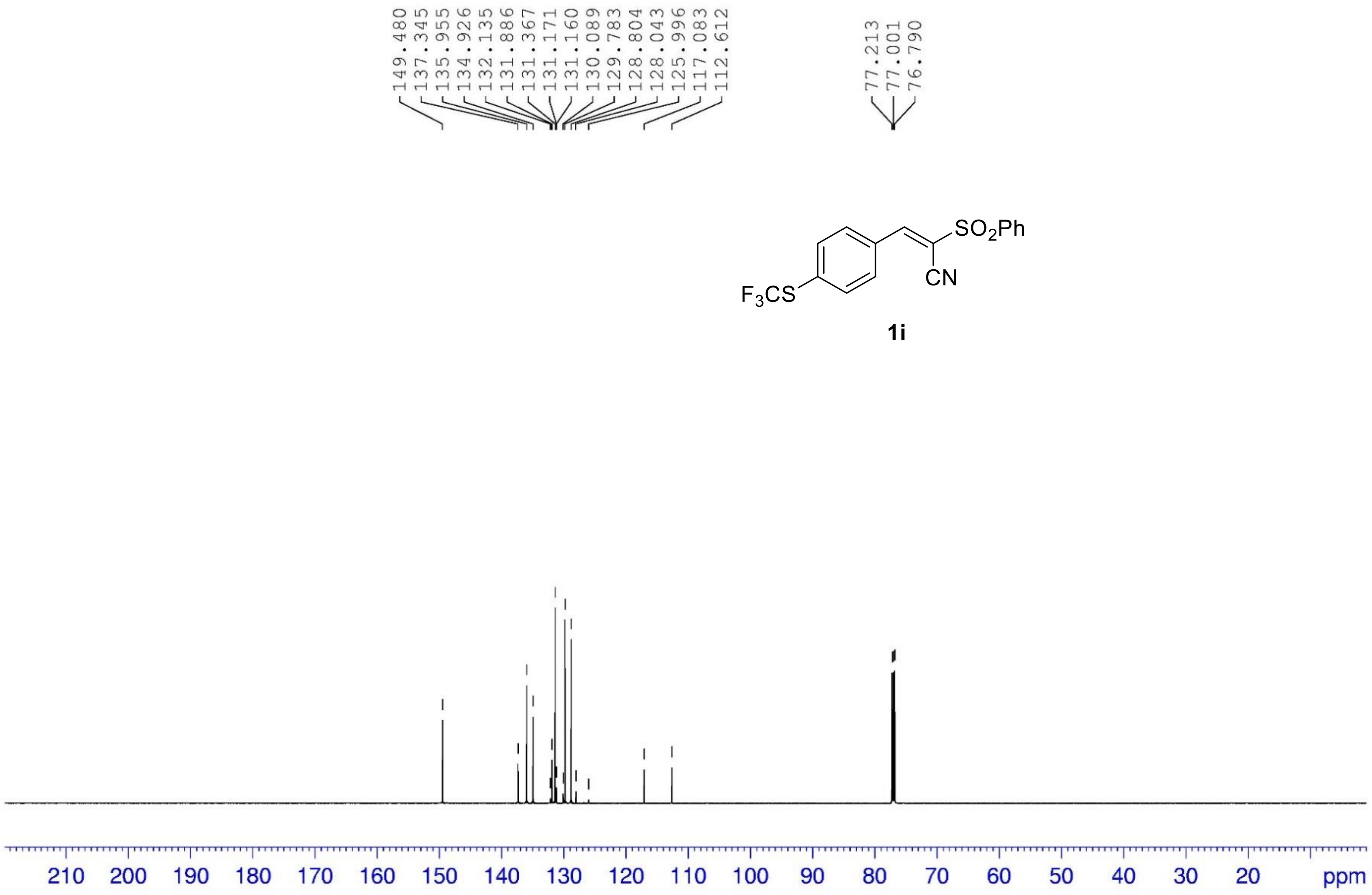

^{1}H NMR in $CDCl_3$ (600 MHz)

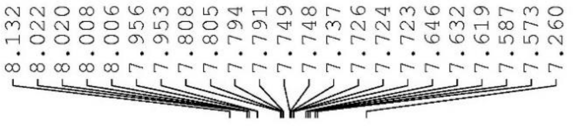

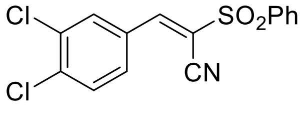

1m

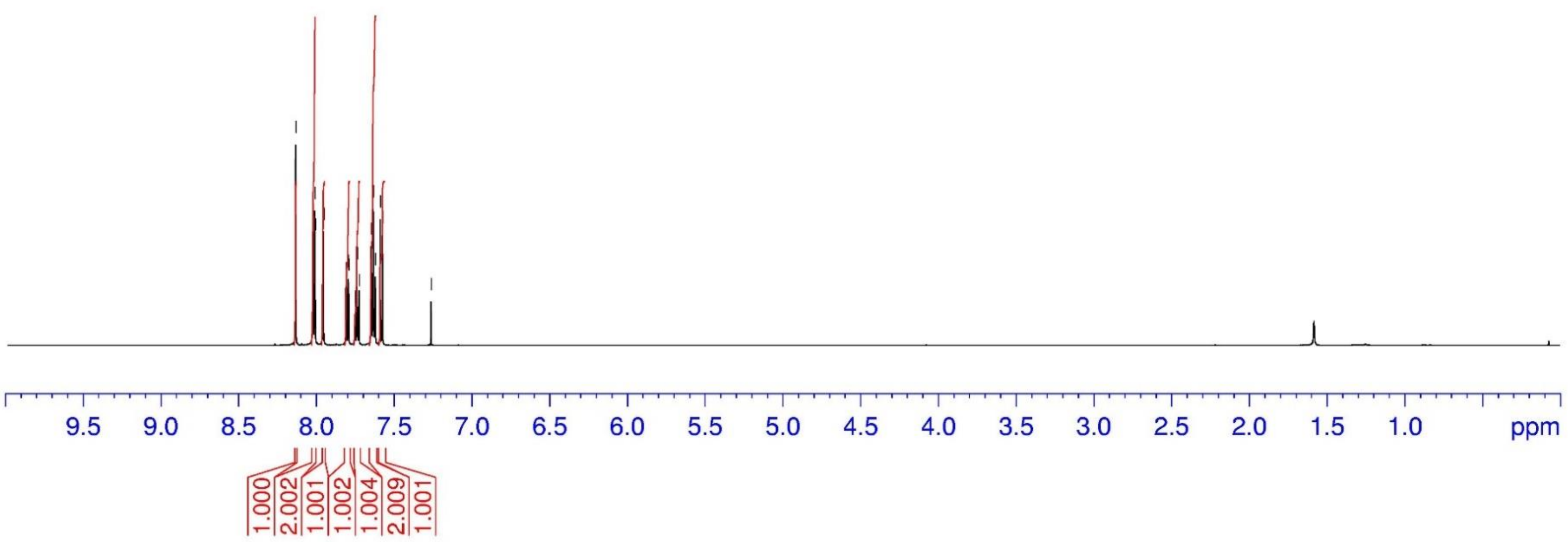

^{13}C NMR in $CDCl_3$ (150 MHz)

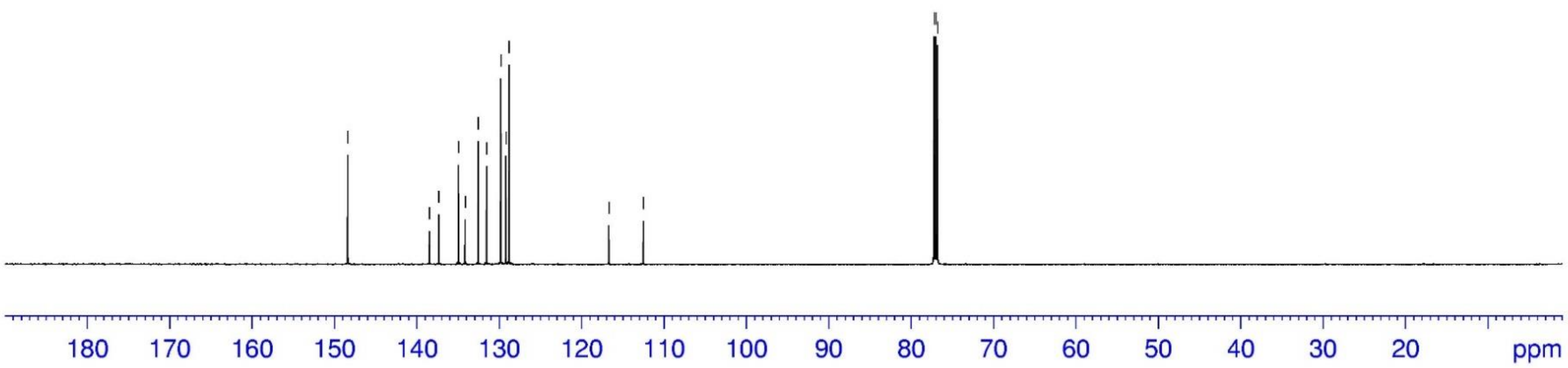

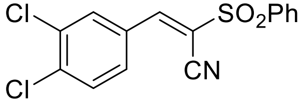

1m

^{1}H NMR in $CDCl_3$ (600 MHz)

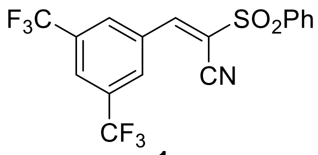

1n

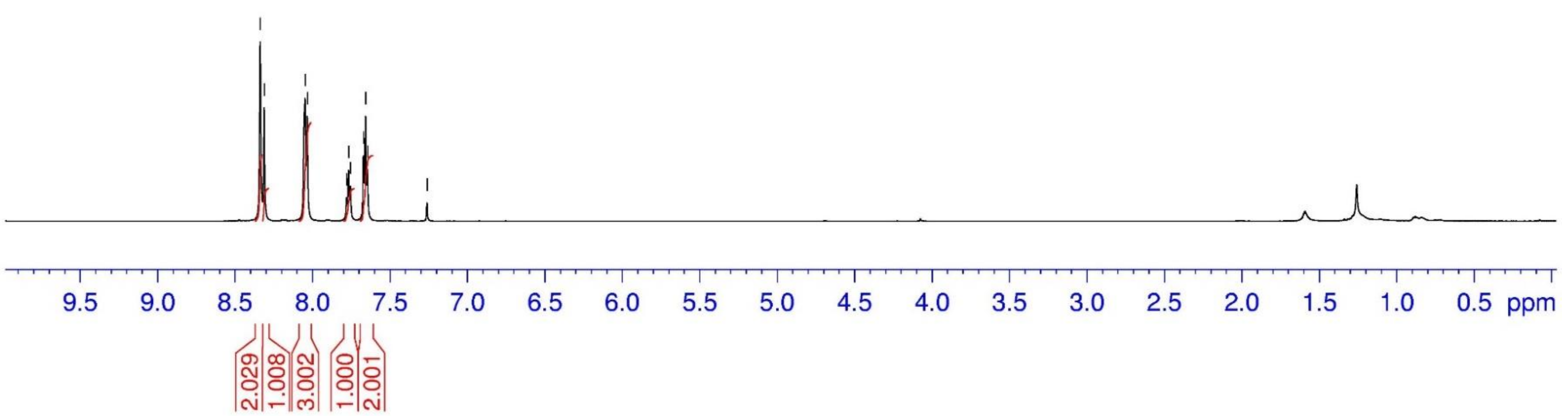

^{13}C NMR in $CDCl_3$ (150 MHz)

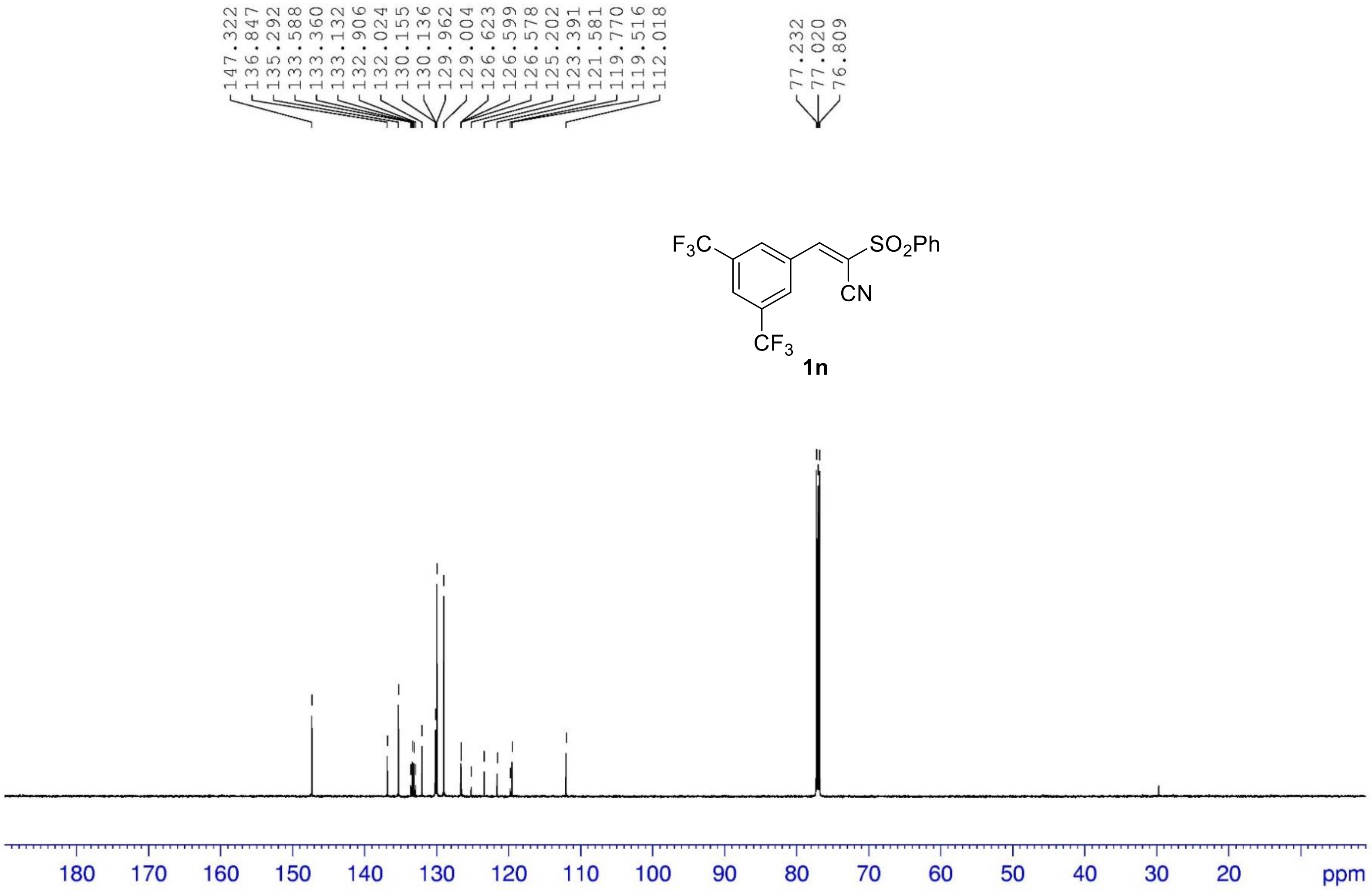

1n

^{1}H NMR in $CDCl_3$ (600 MHz)

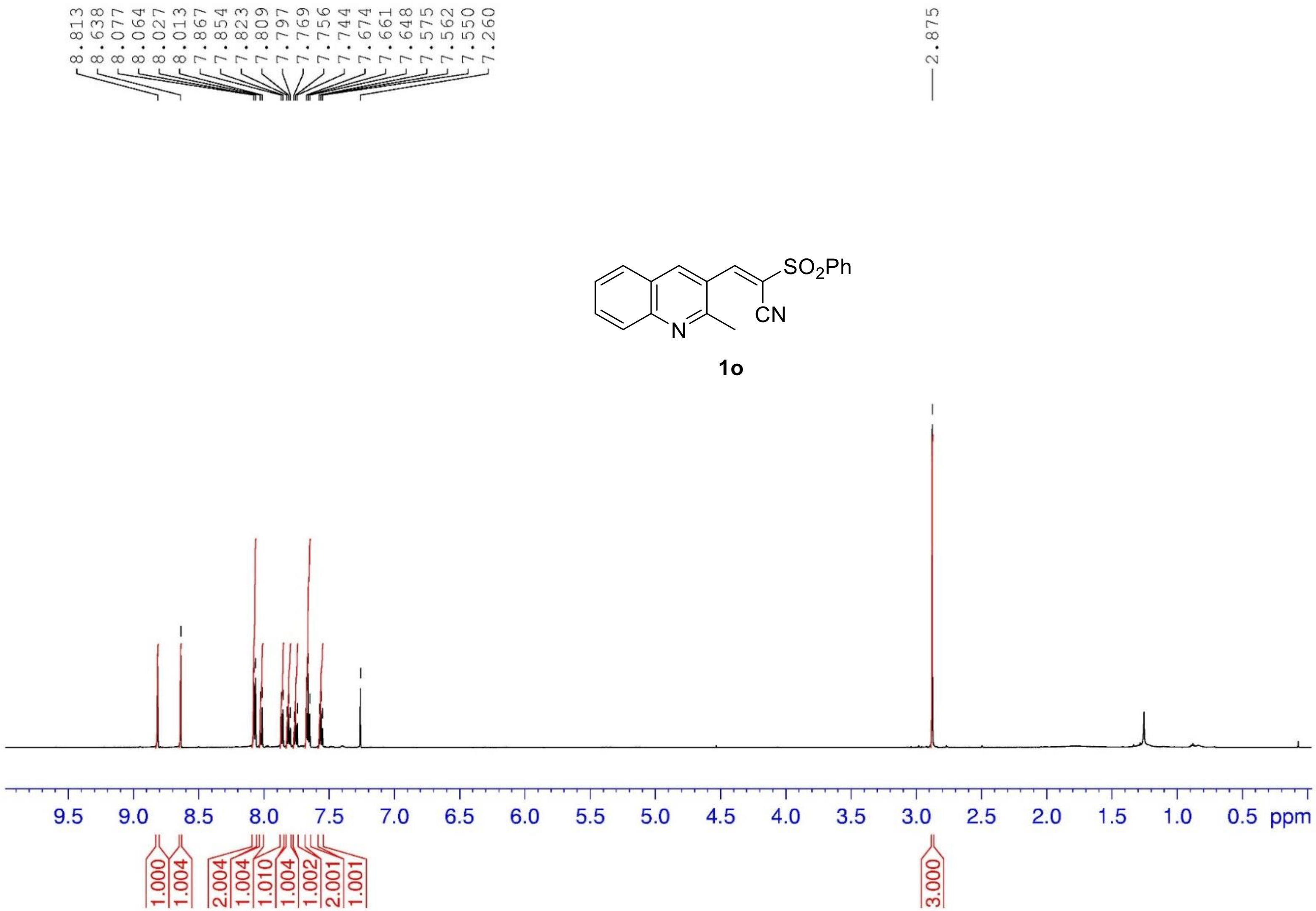

^{13}C NMR in $CDCl_3$ (150 MHz)

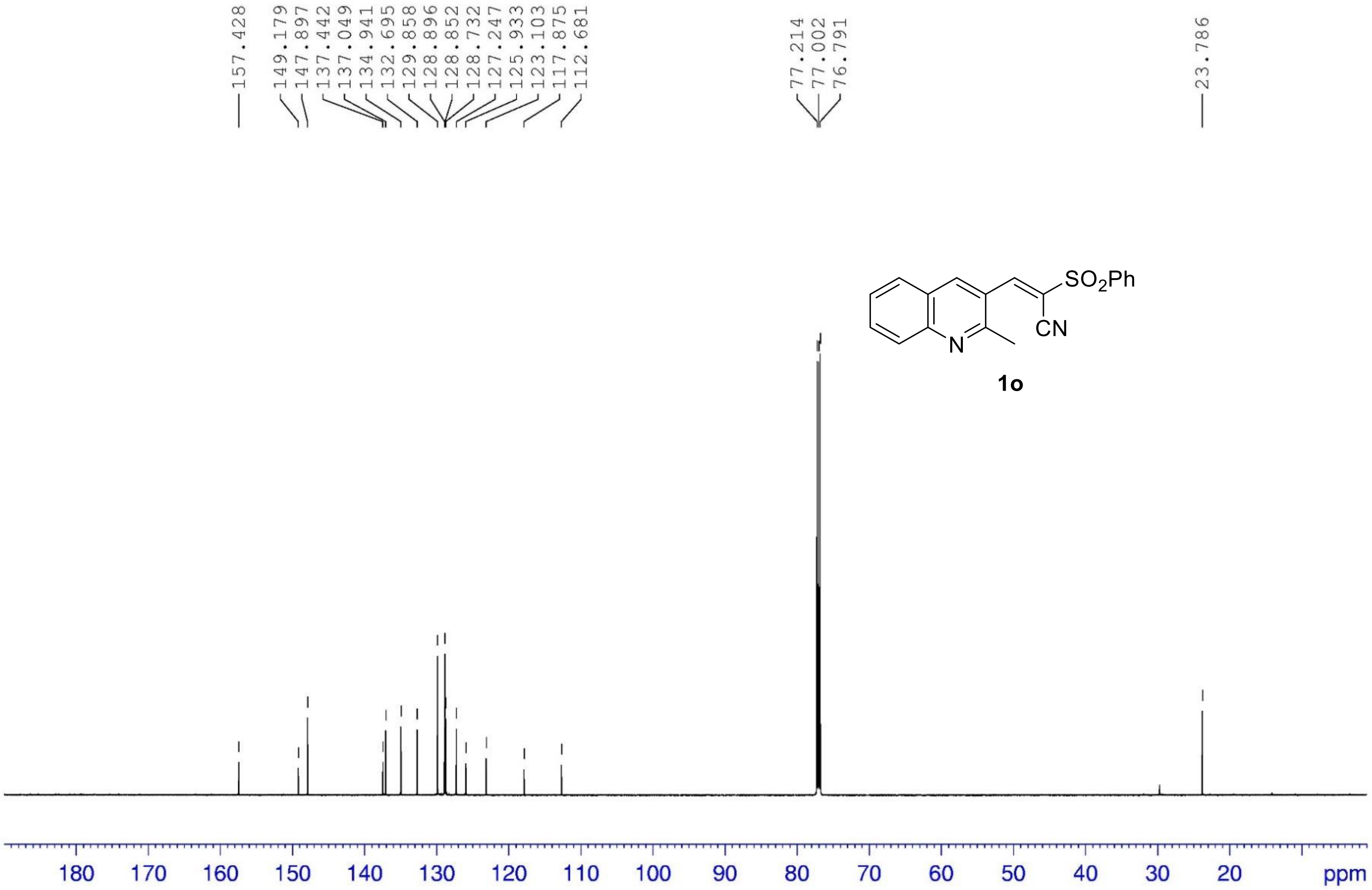

^{1}H NMR in $CDCl_3$ (600 MHz)

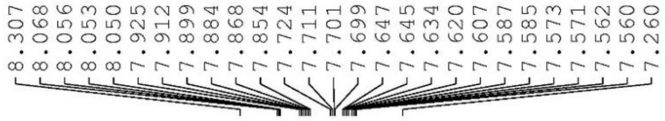

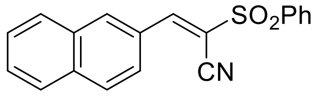

1q

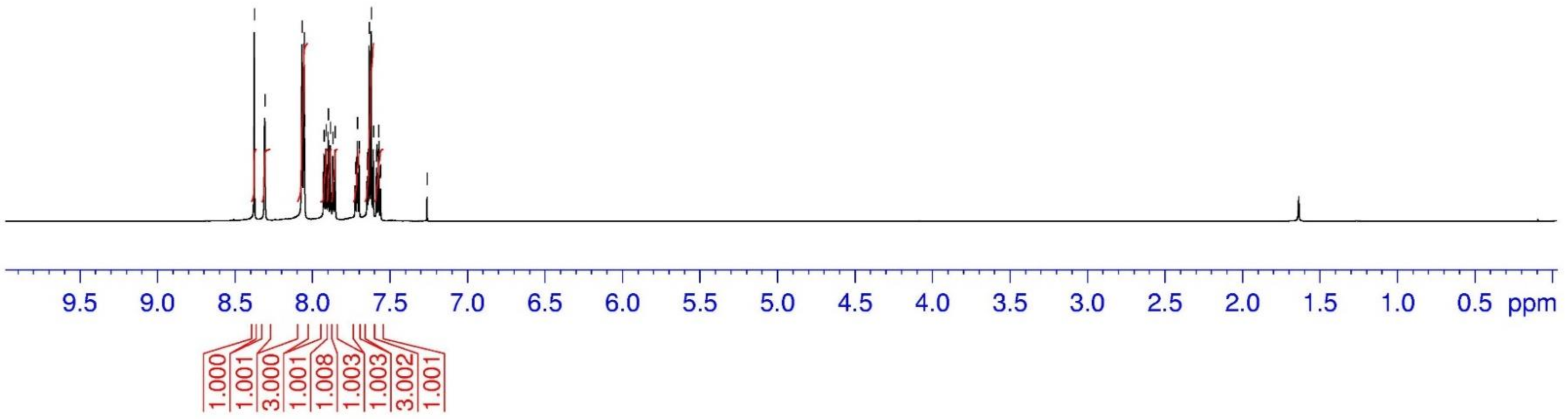

^{13}C NMR in $CDCl_3$ (150 MHz)

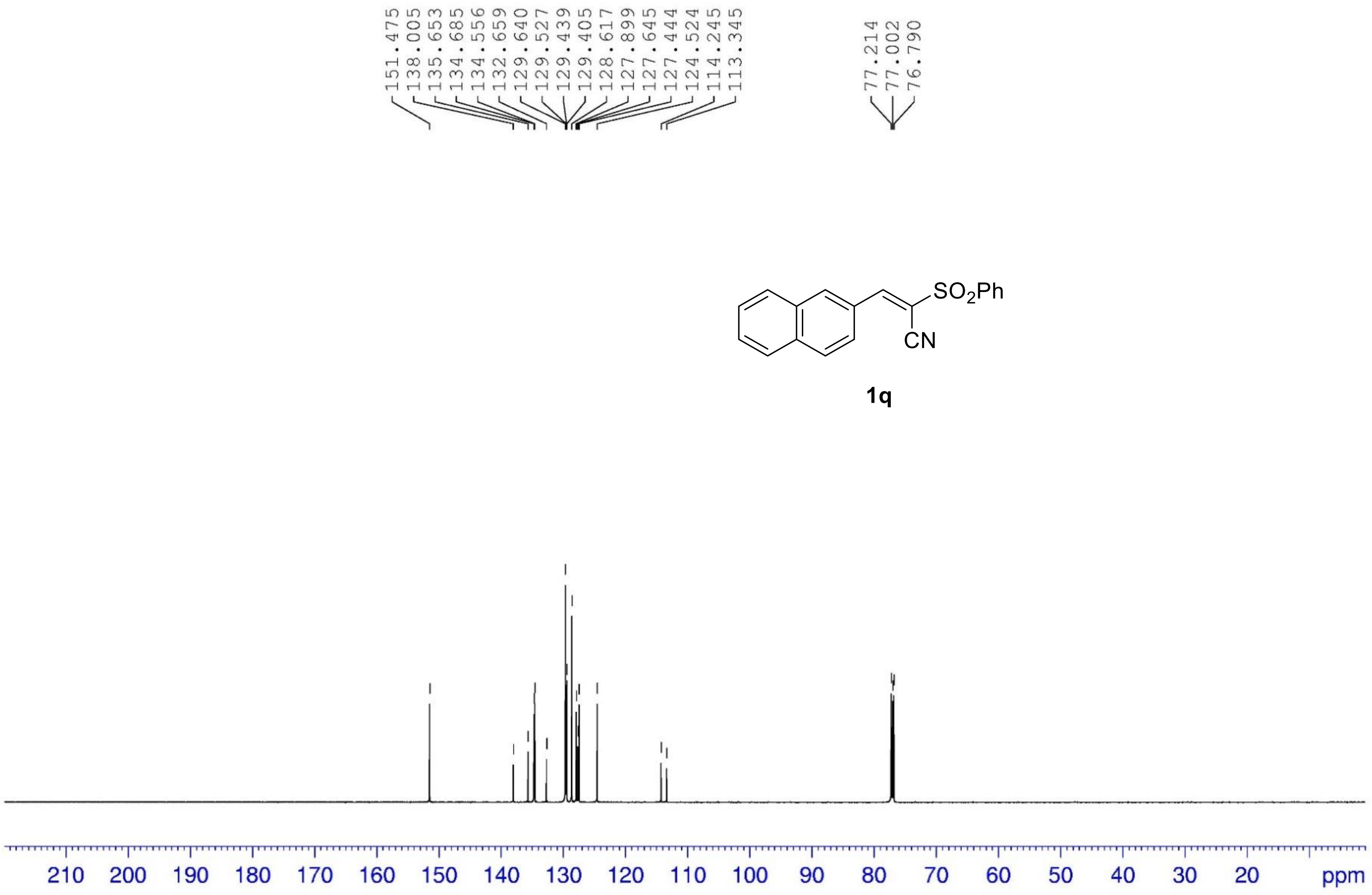

1q

^1H NMR in $CDCl_3$ (400 MHz)

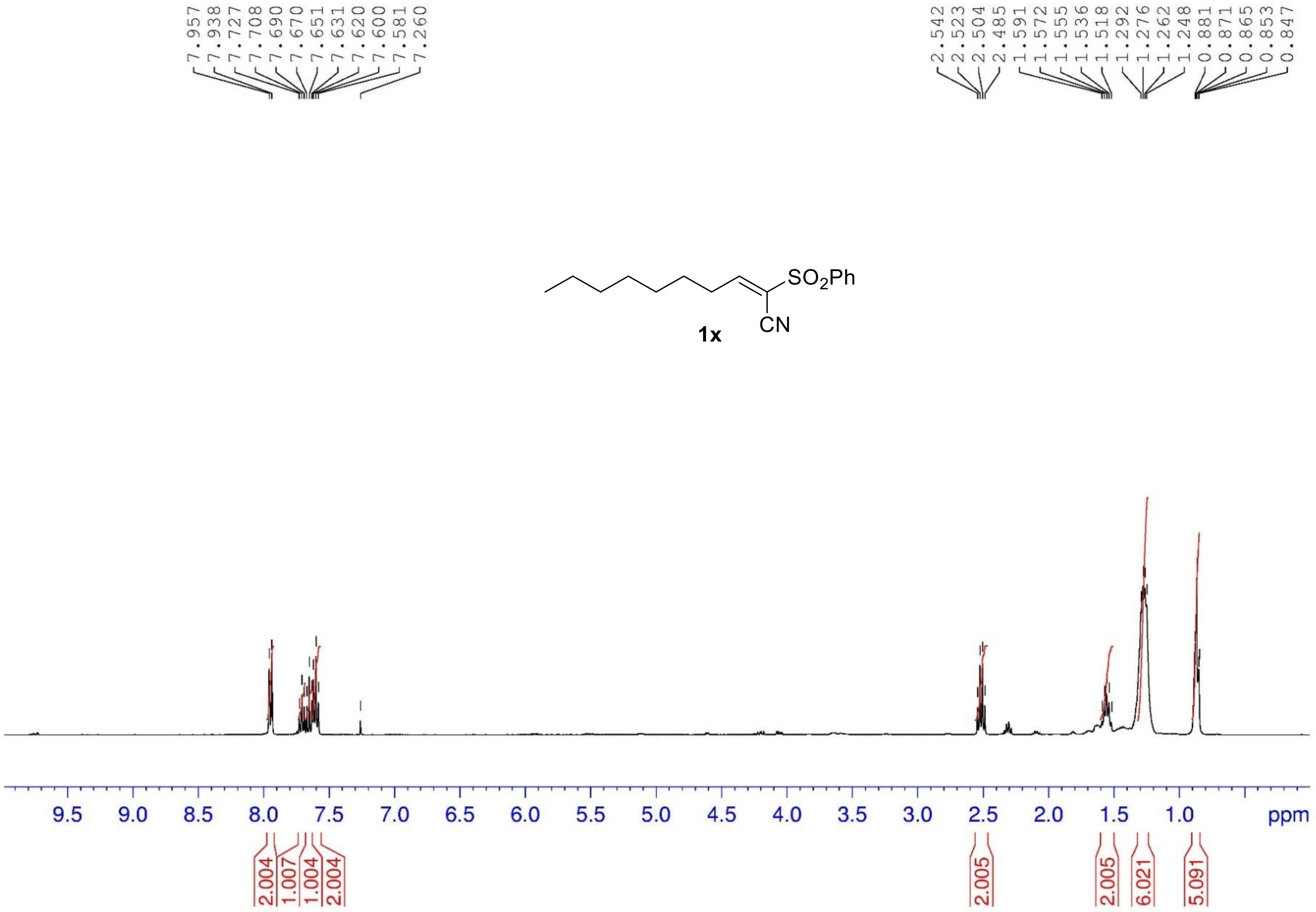

^{13}C NMR in $CDCl_3$ (100 MHz)

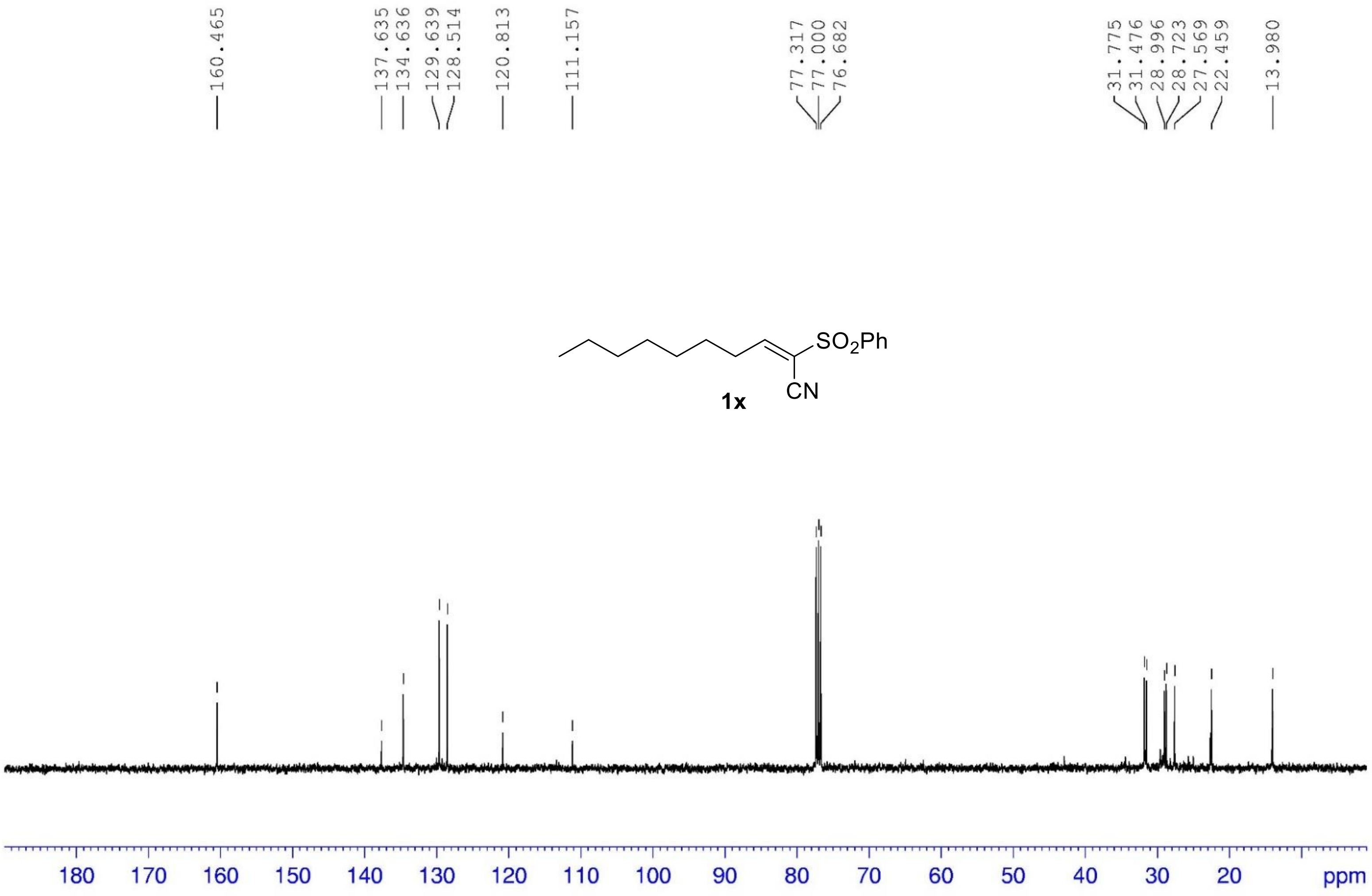

^{1}H NMR in $CDCl_3$ (400 MHz)

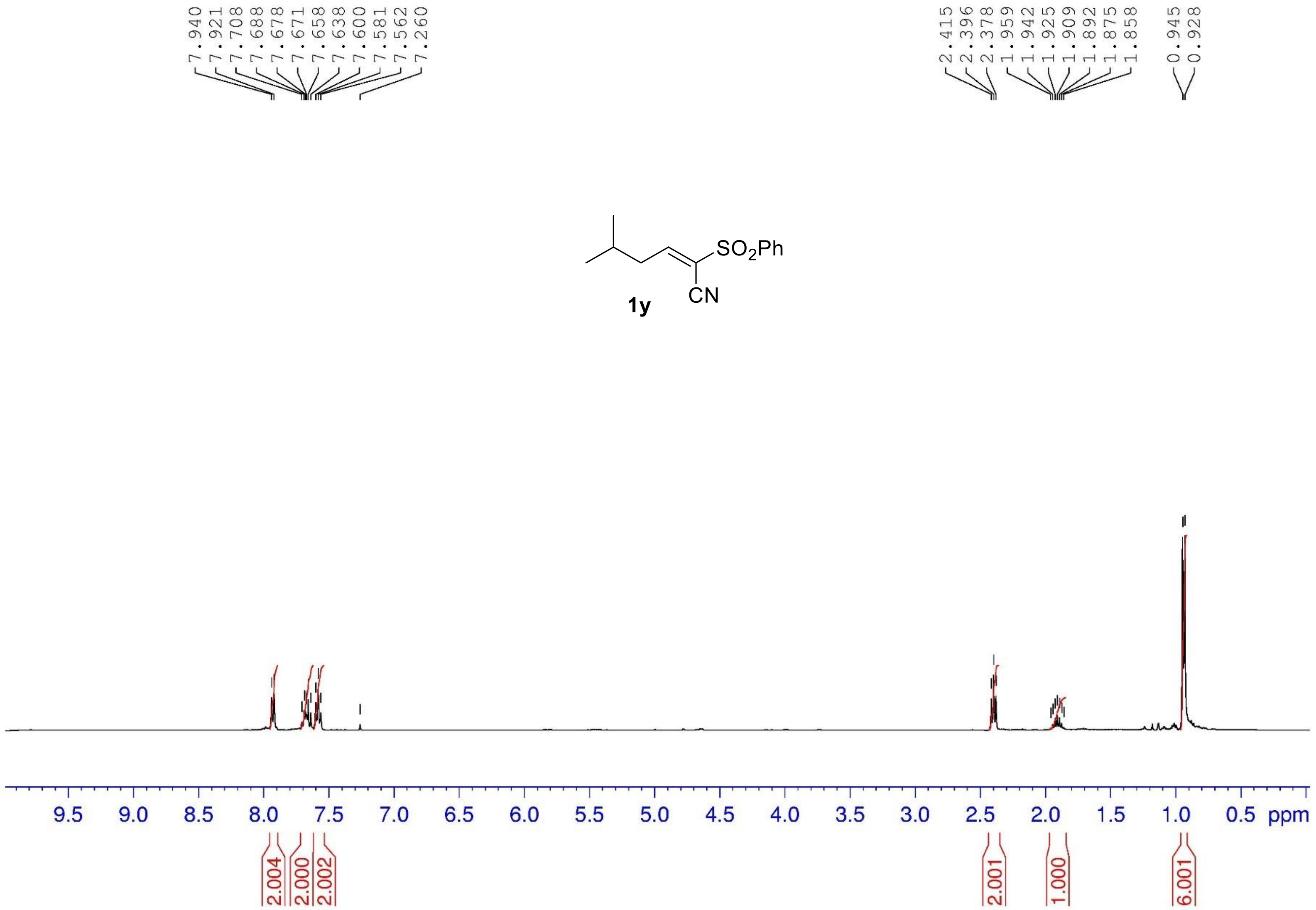

^{13}C NMR in $CDCl_3$ (100 MHz)

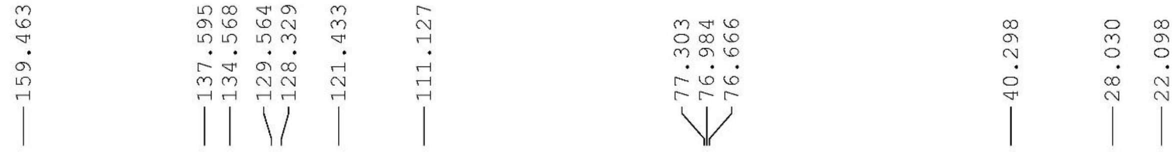

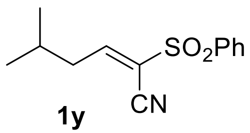

1y

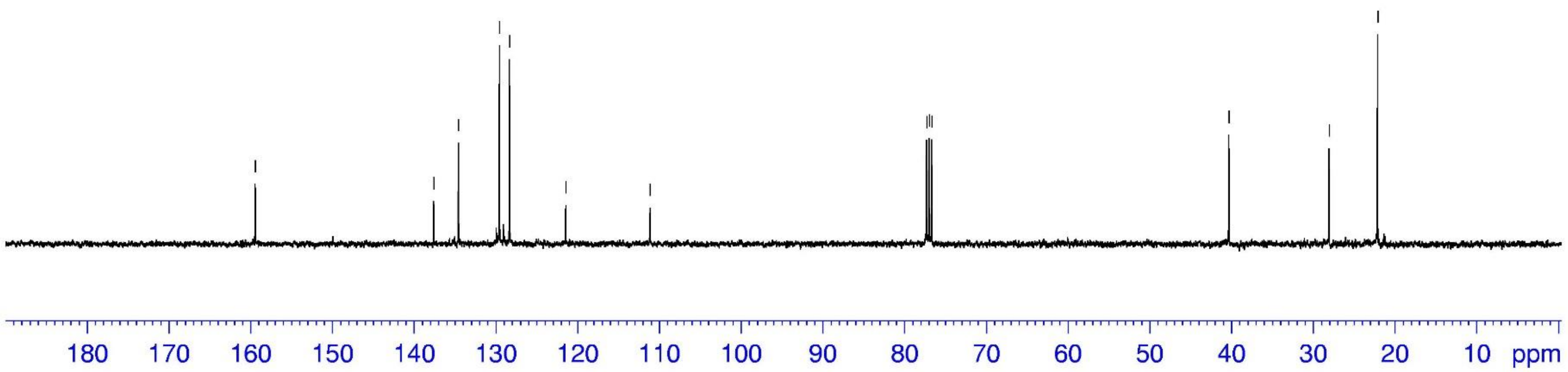

^{1}H NMR in $CDCl_3$ (300 MHz)

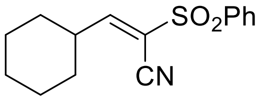

1ab

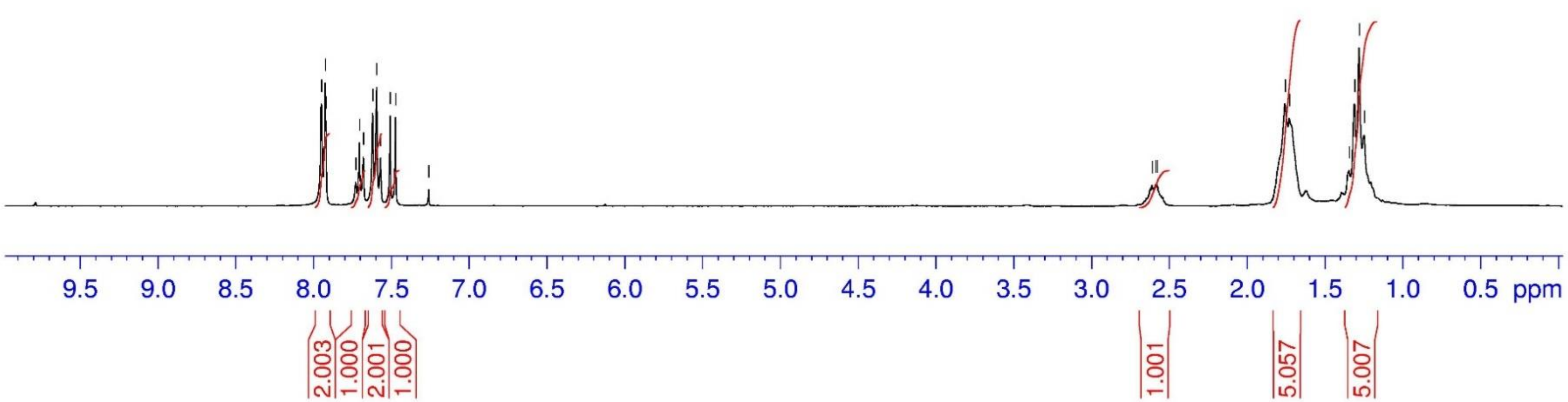

^{13}C NMR in $CDCl_3$ (75 MHz)

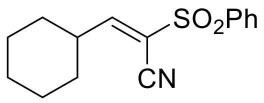

1ab

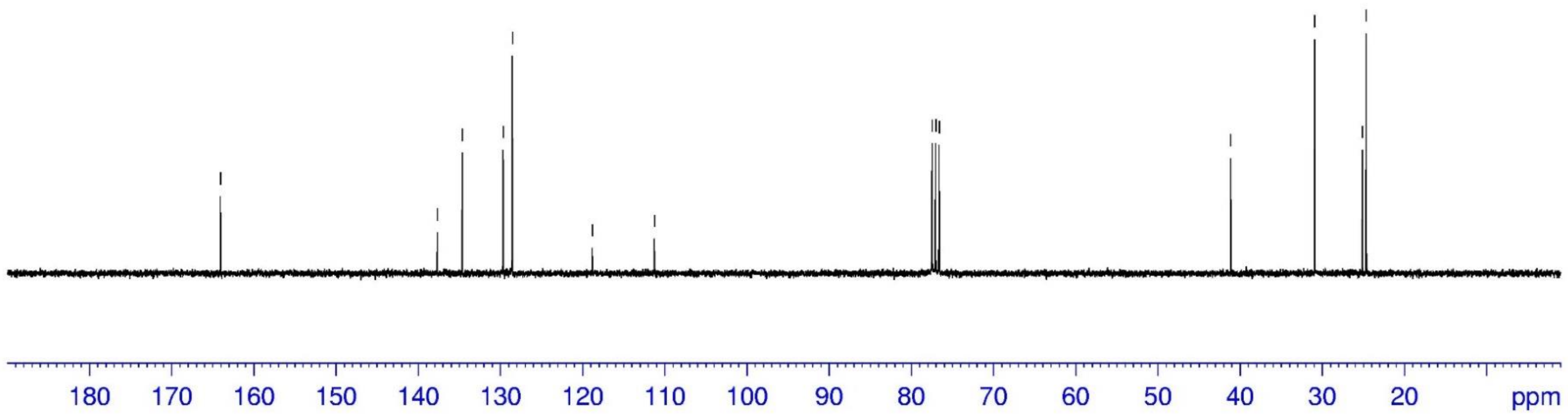

^{1}H NMR in $CDCl_3$ (400 MHz)

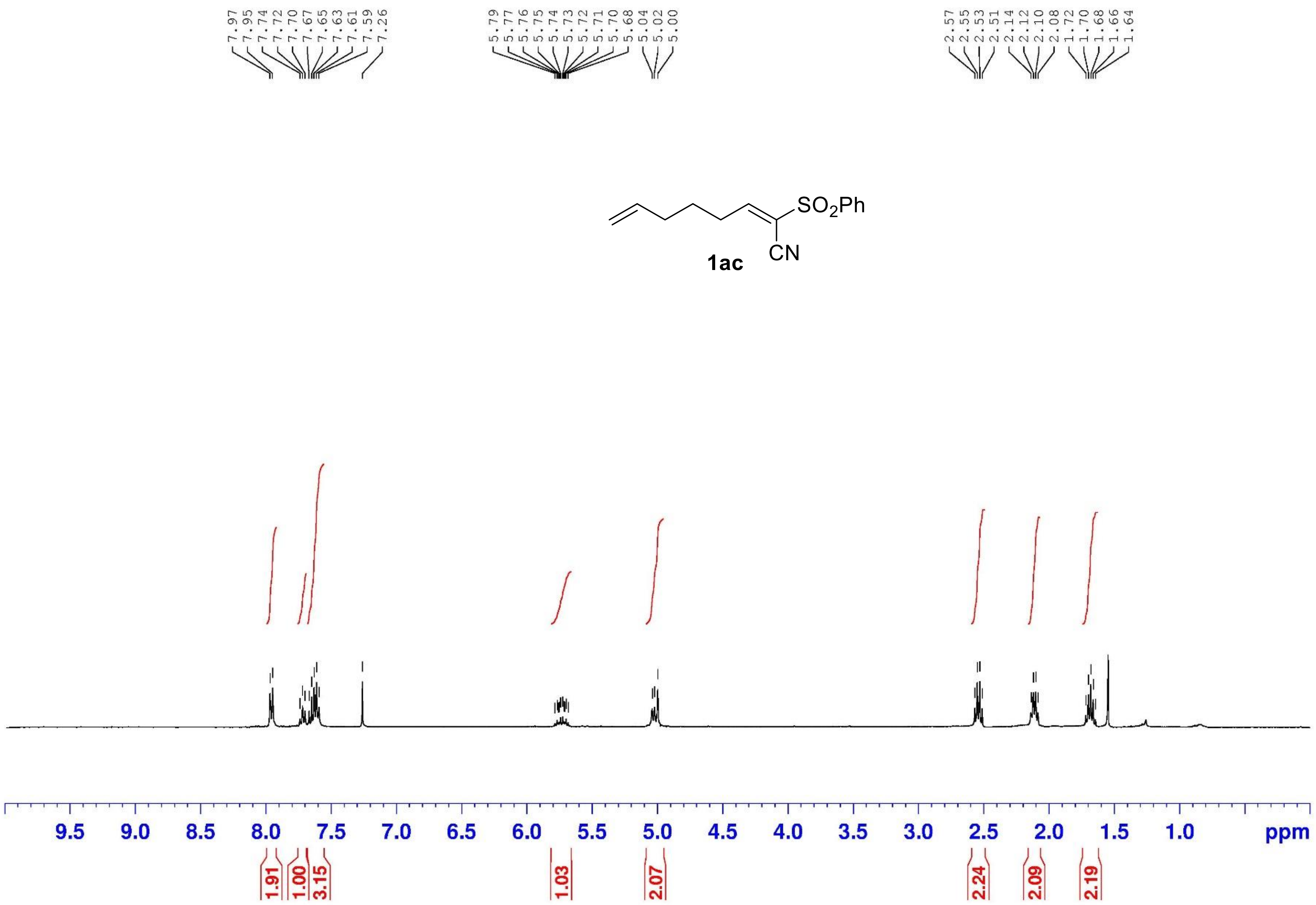

^{13}C NMR in $CDCl_3$ (150 MHz)

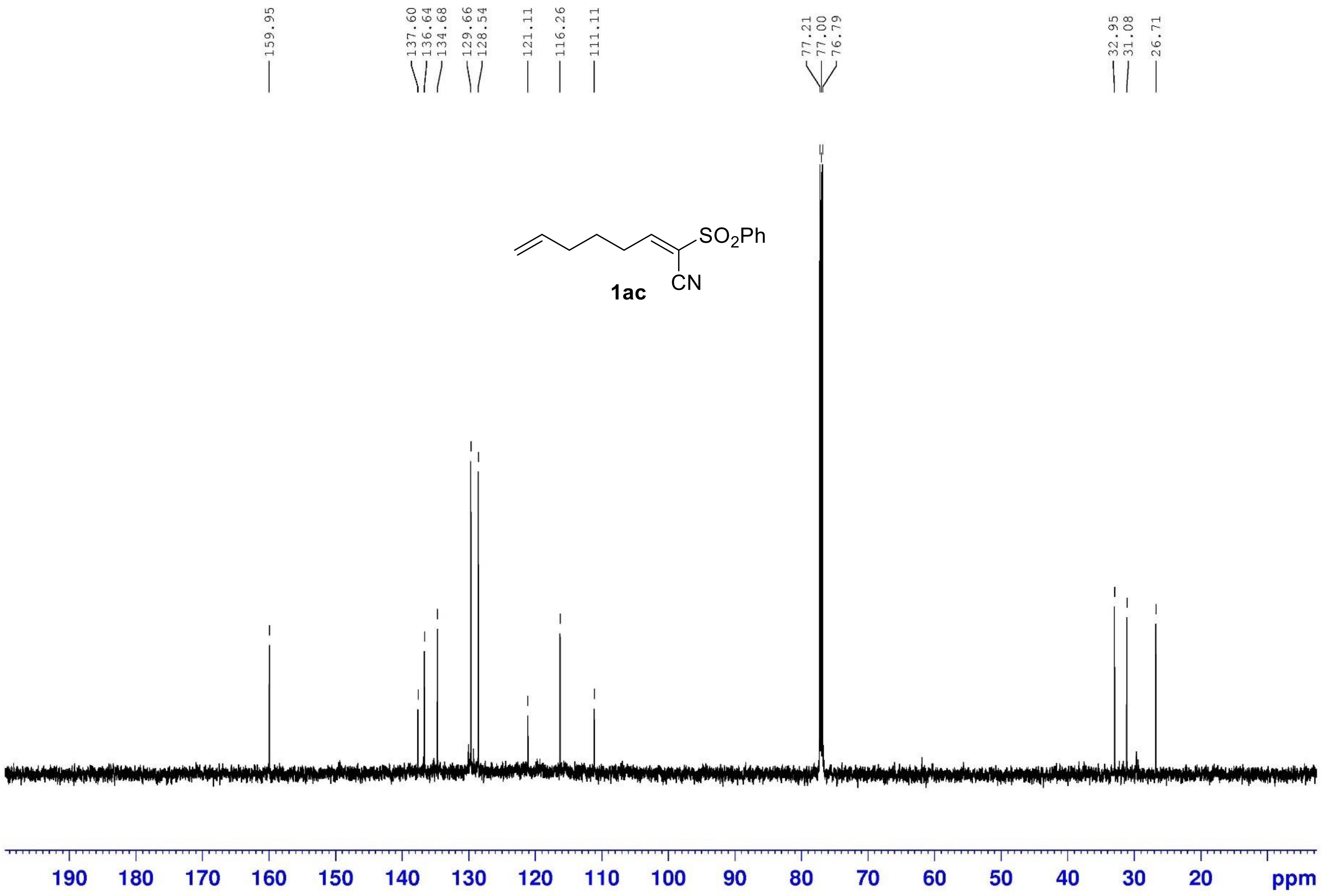

^{1}H NMR in $CDCl_3$ (600 MHz)

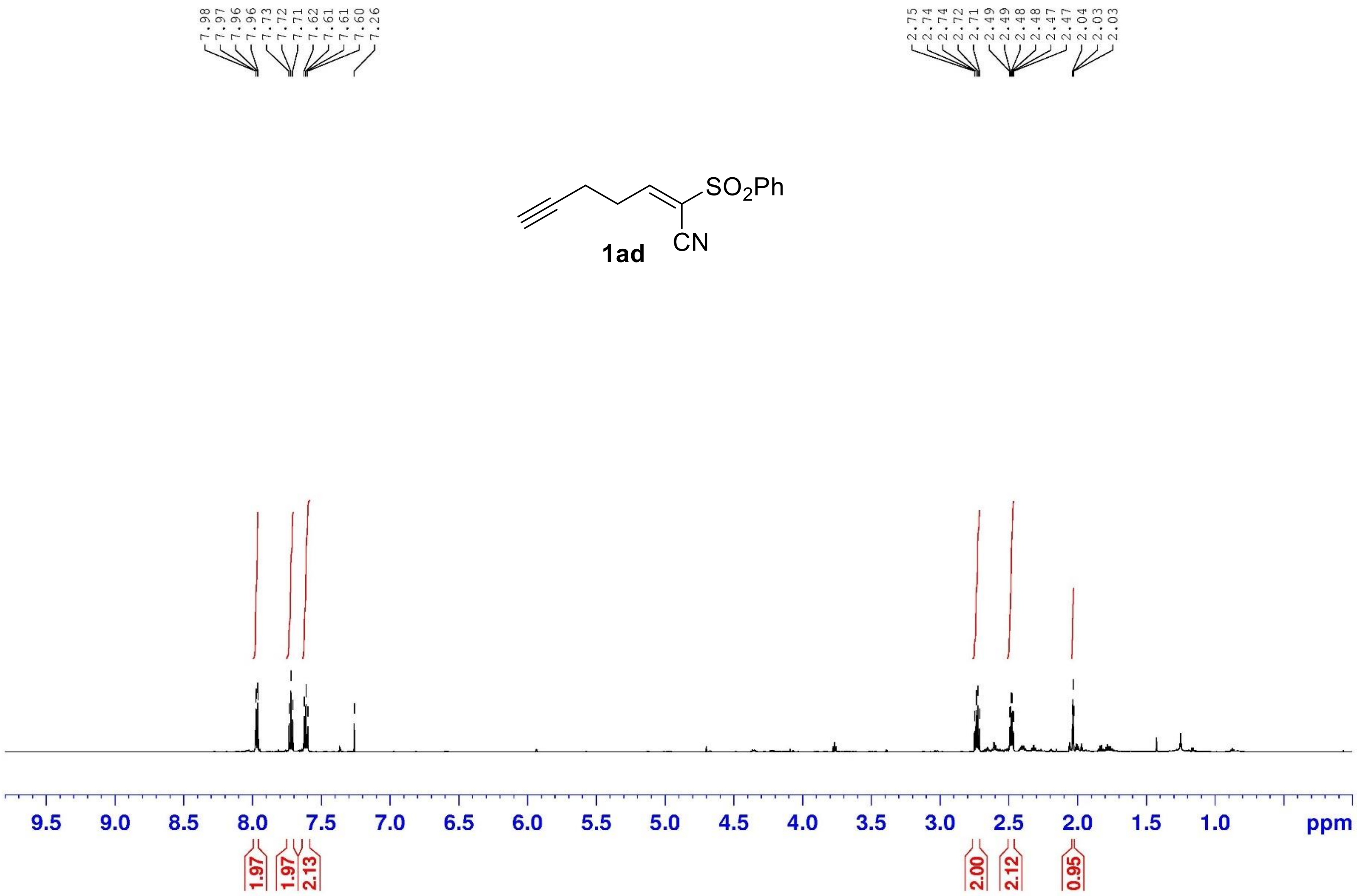

^{13}C NMR in $CDCl_3$ (150 MHz)

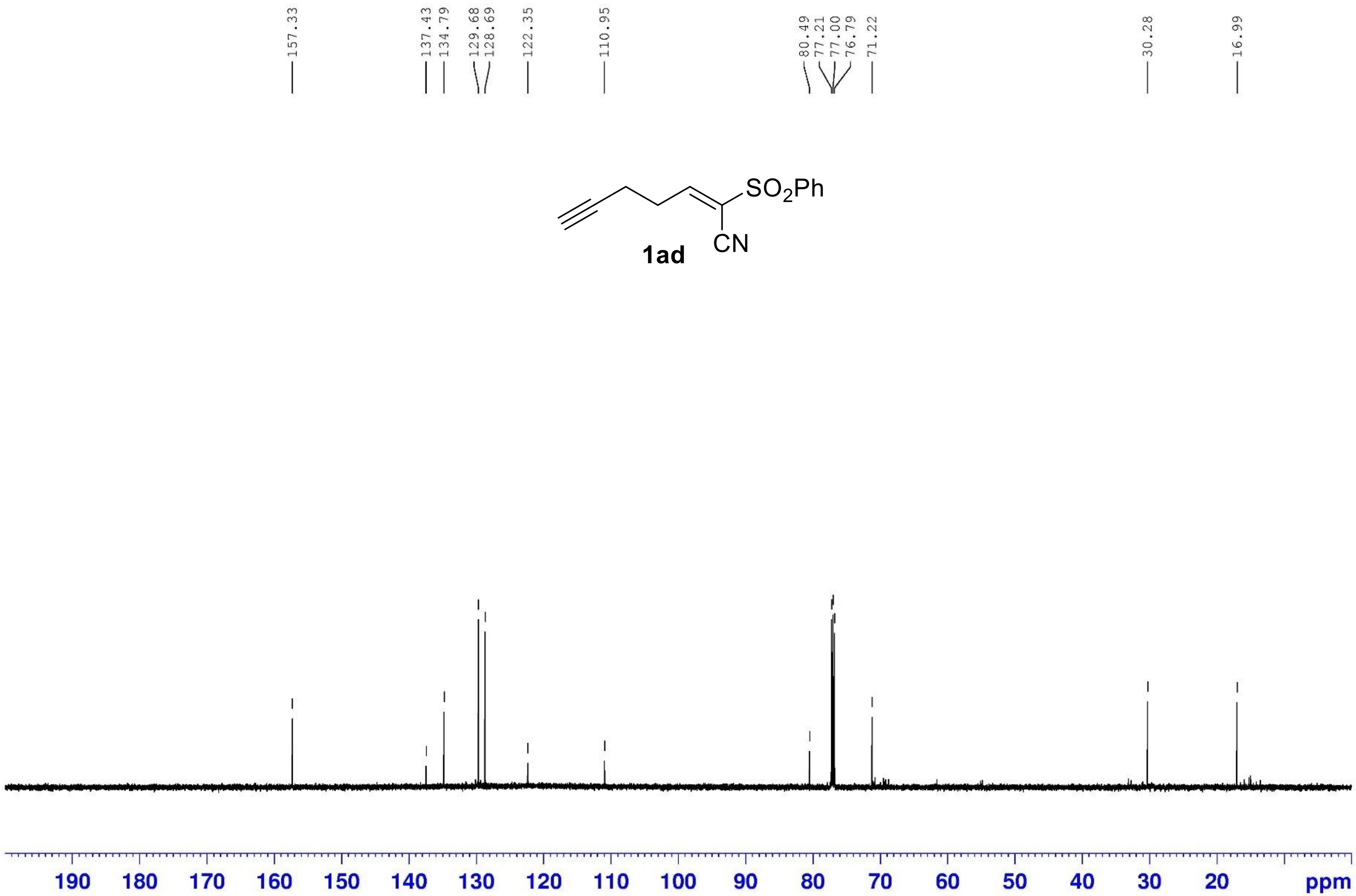

^{1}H NMR in $CDCl_3$ (300 MHz)

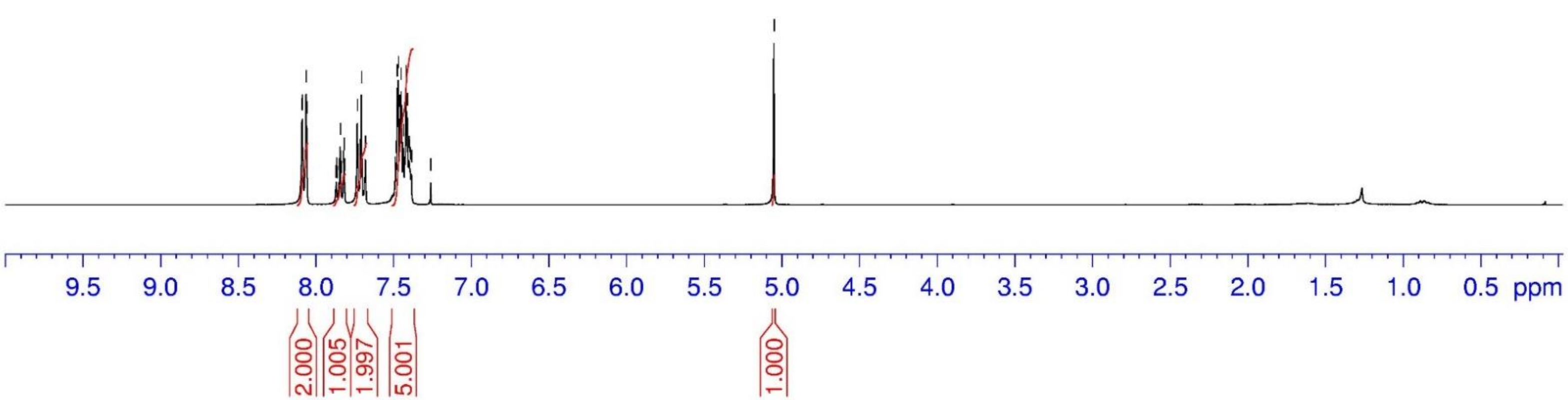

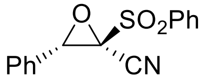

2a

^{13}C NMR in $CDCl_3$ (75 MHz)

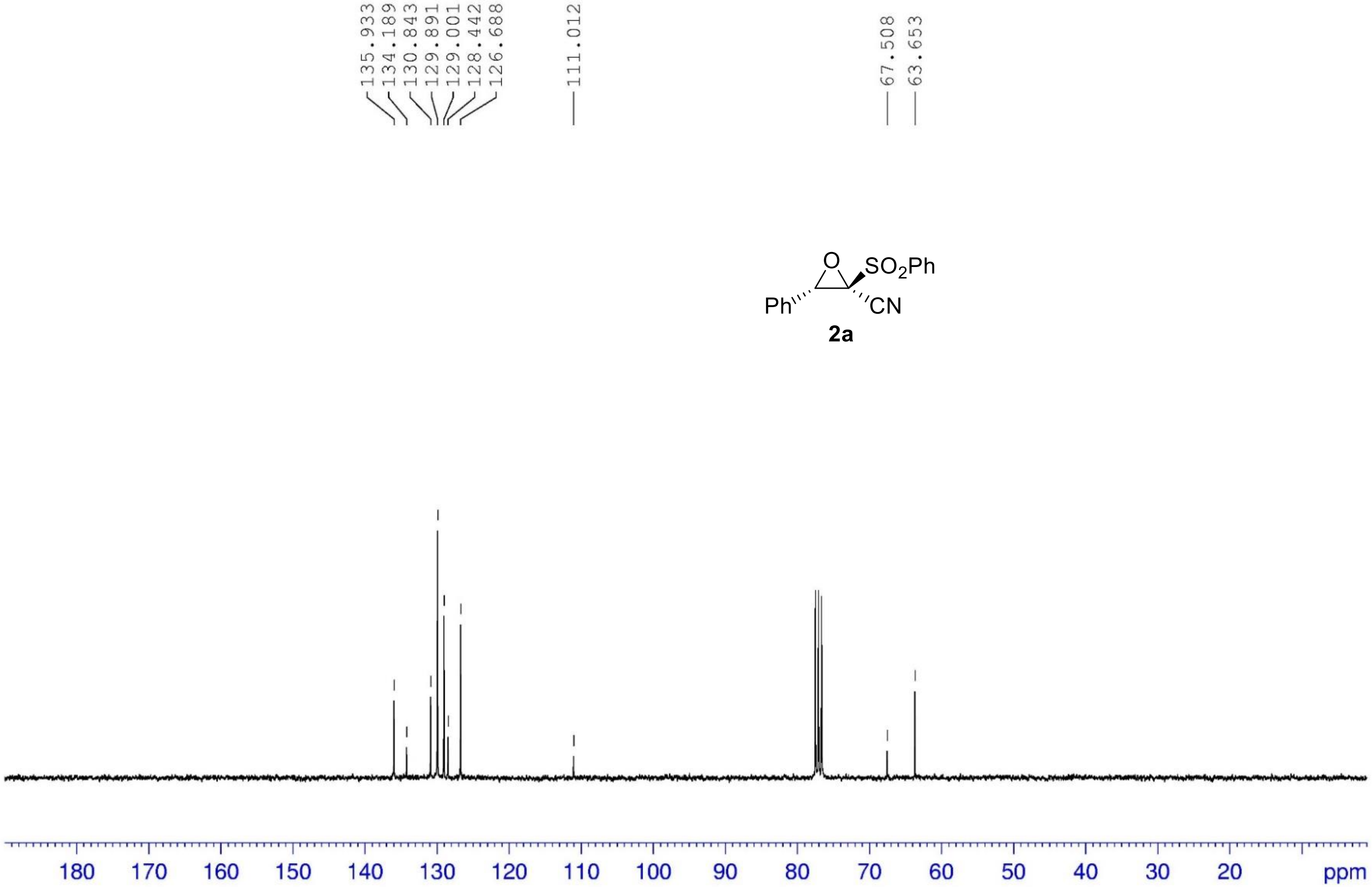

2a

^{1}H NMR in $CDCl_3$ (400 MHz)

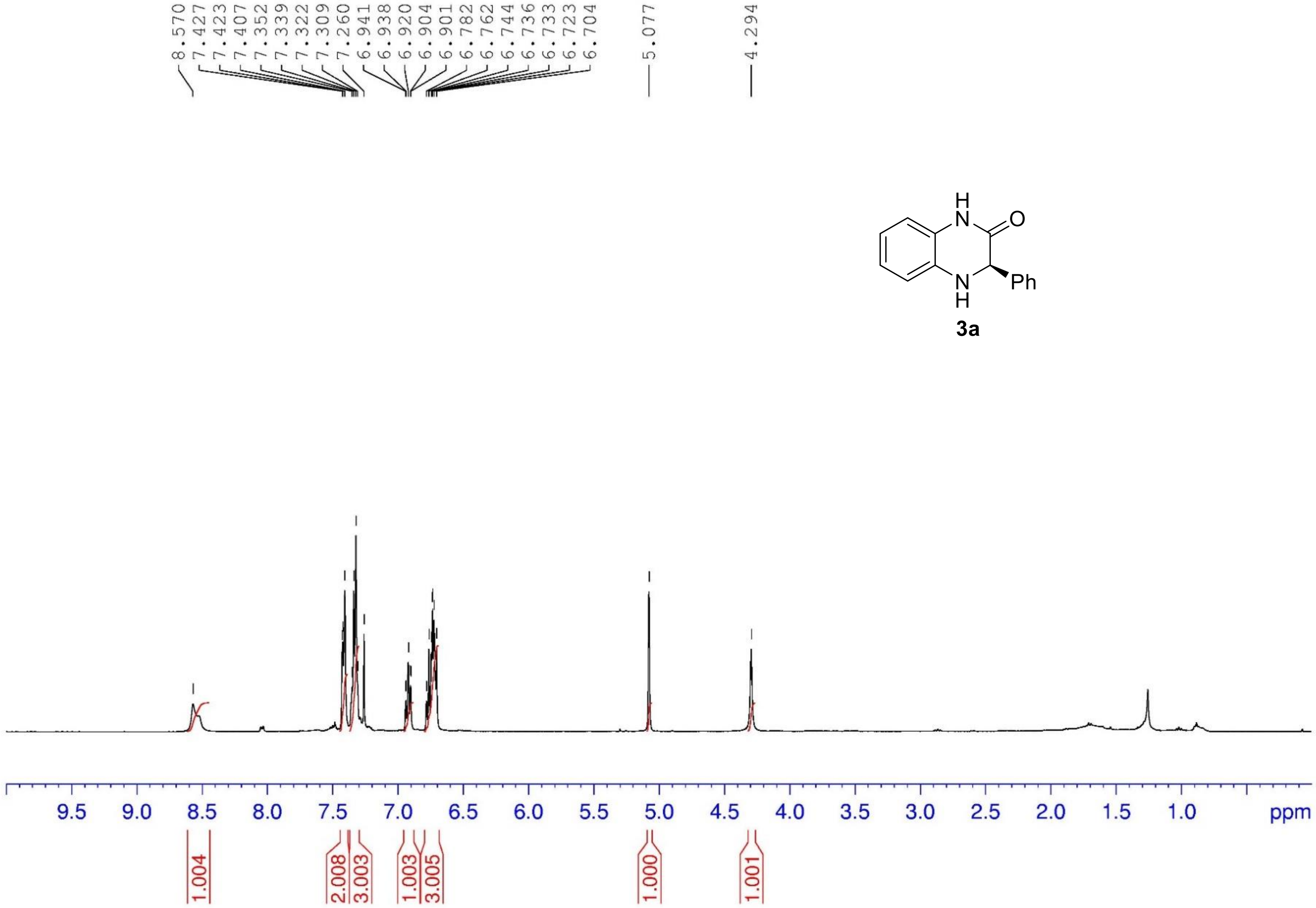

3a

^{13}C NMR in $CDCl_3$ (100 MHz)

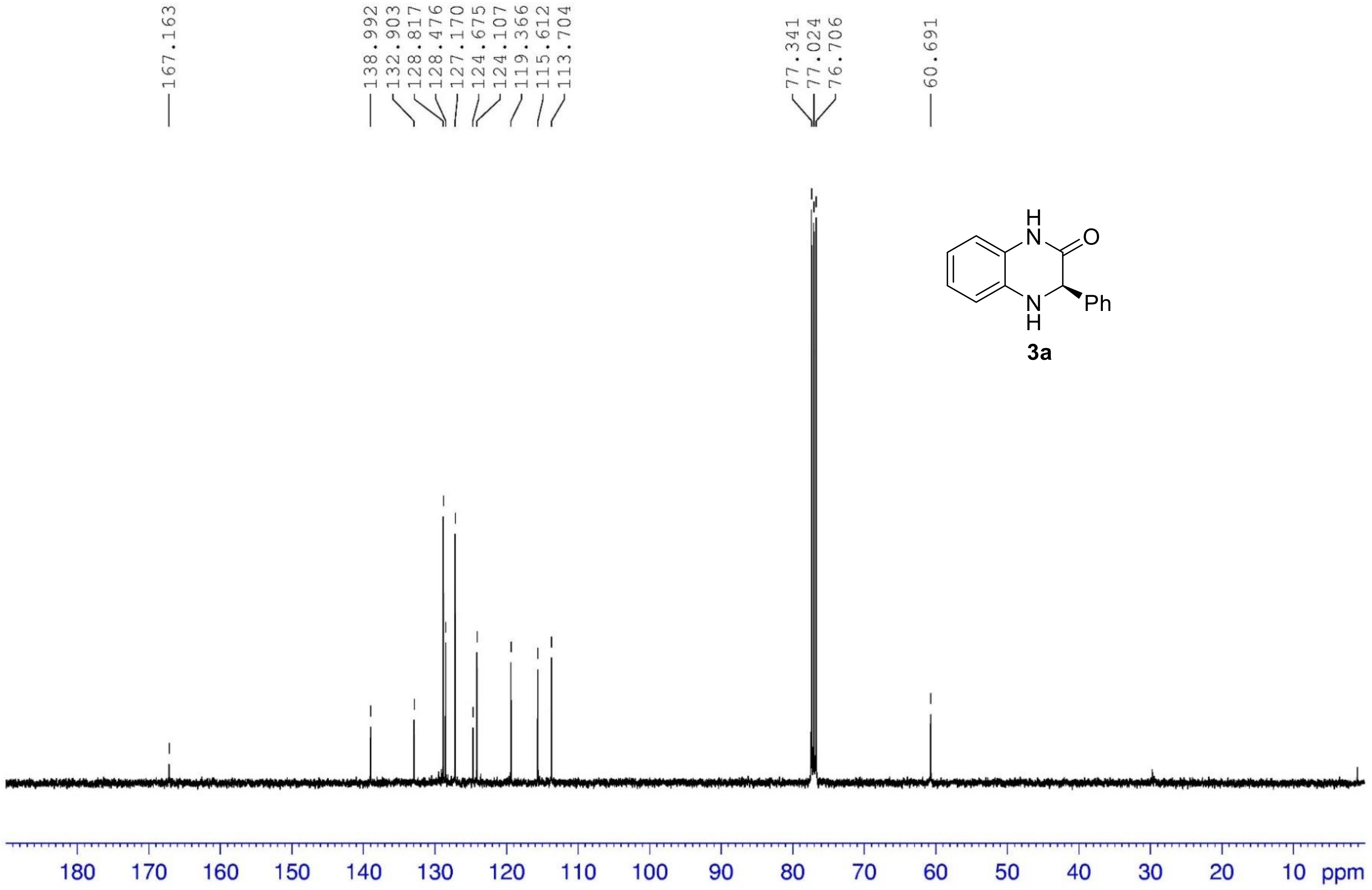

3a

^{1}H NMR in $CDCl_3$ (400 MHz)

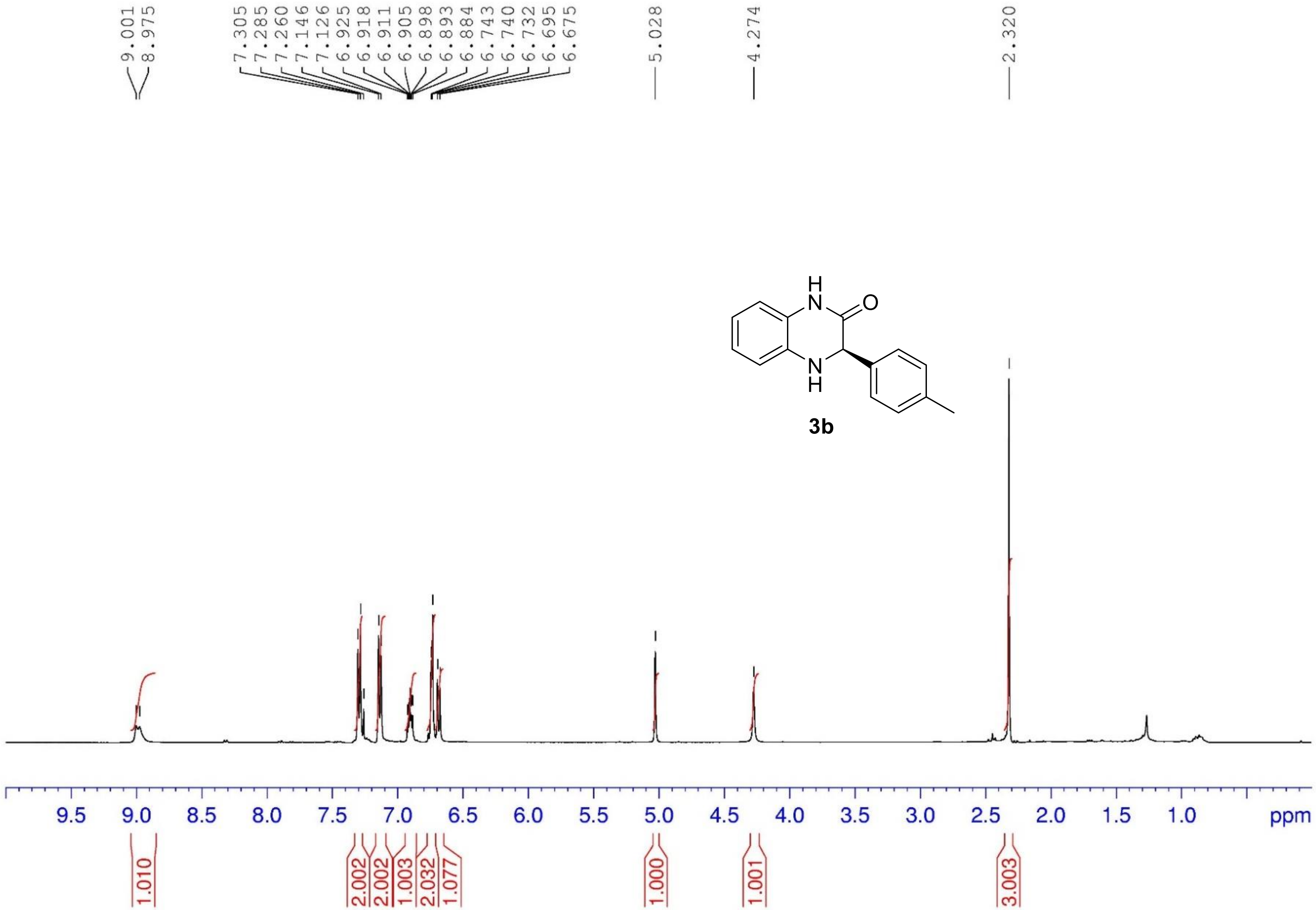

^{13}C NMR in $CDCl_3$ (100 MHz)

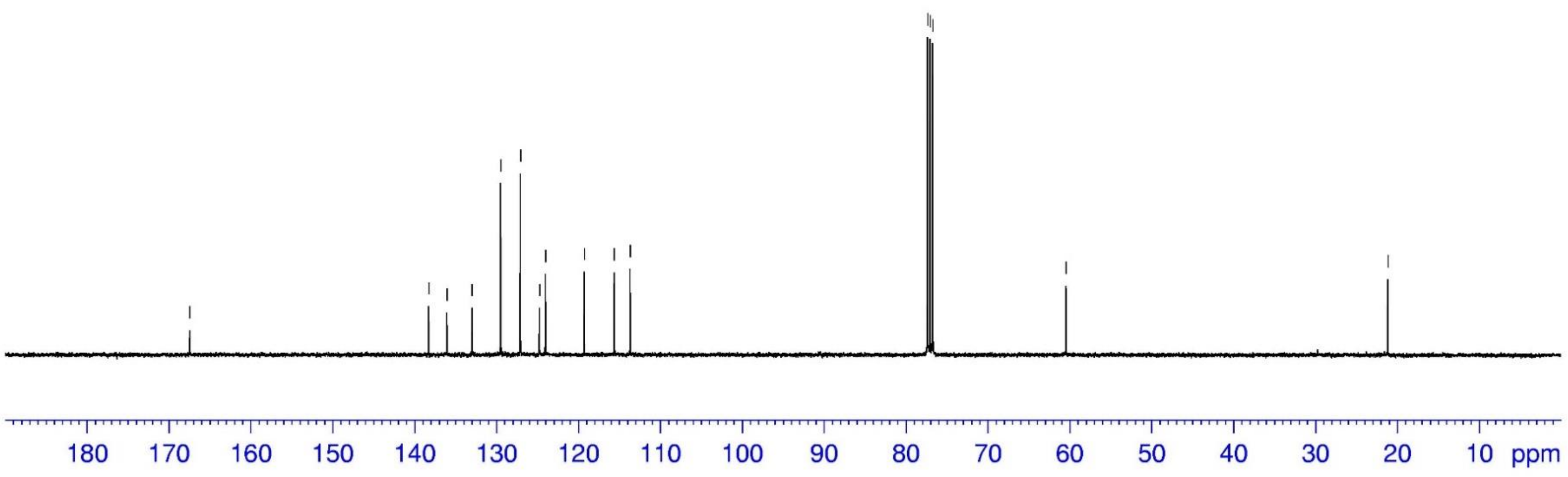

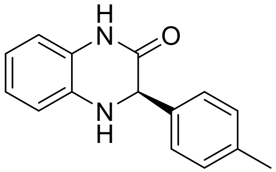

3b

^{1}H NMR in MeOD (400 MHz)

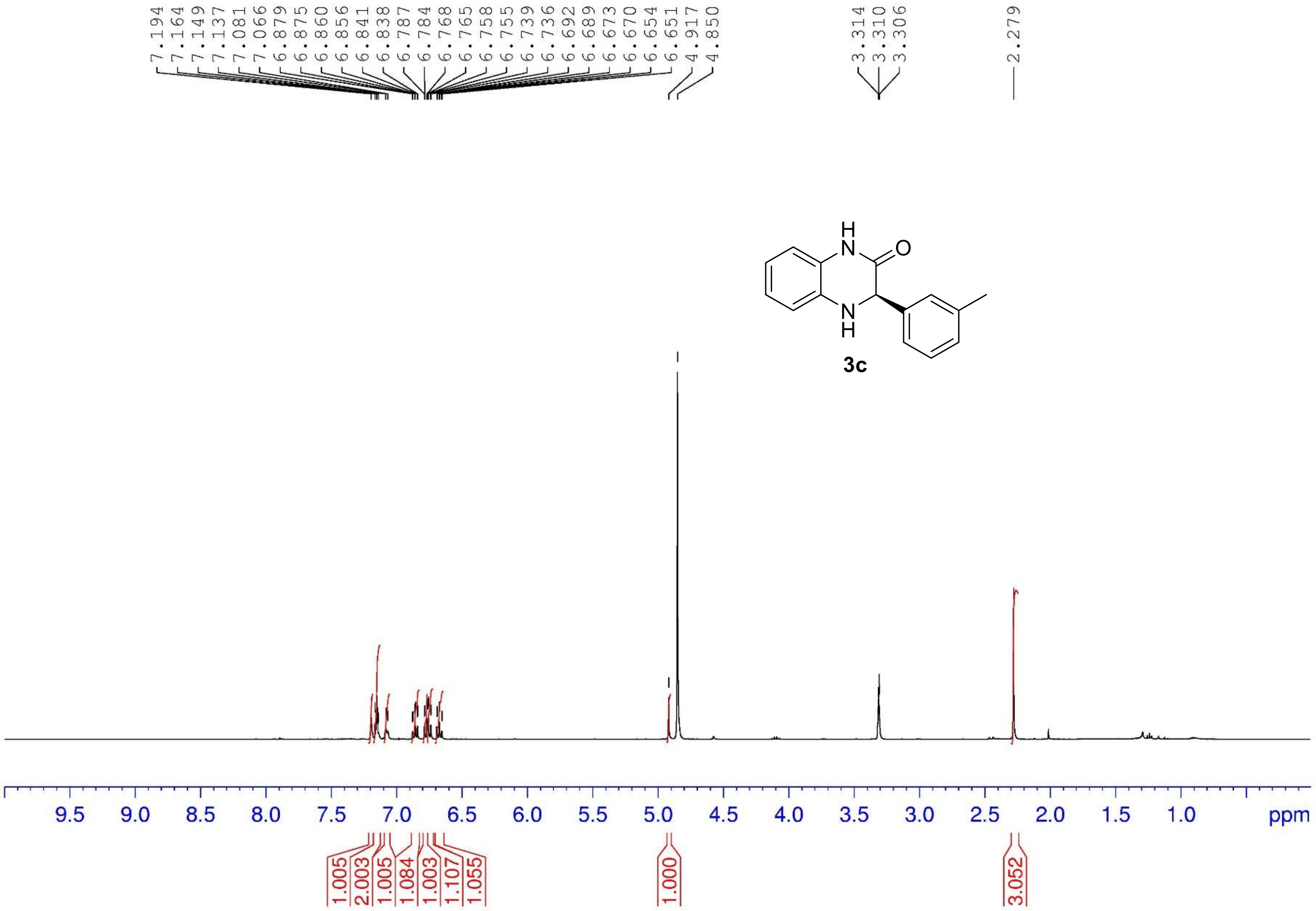

^{13}C NMR in MeOD (150 MHz)

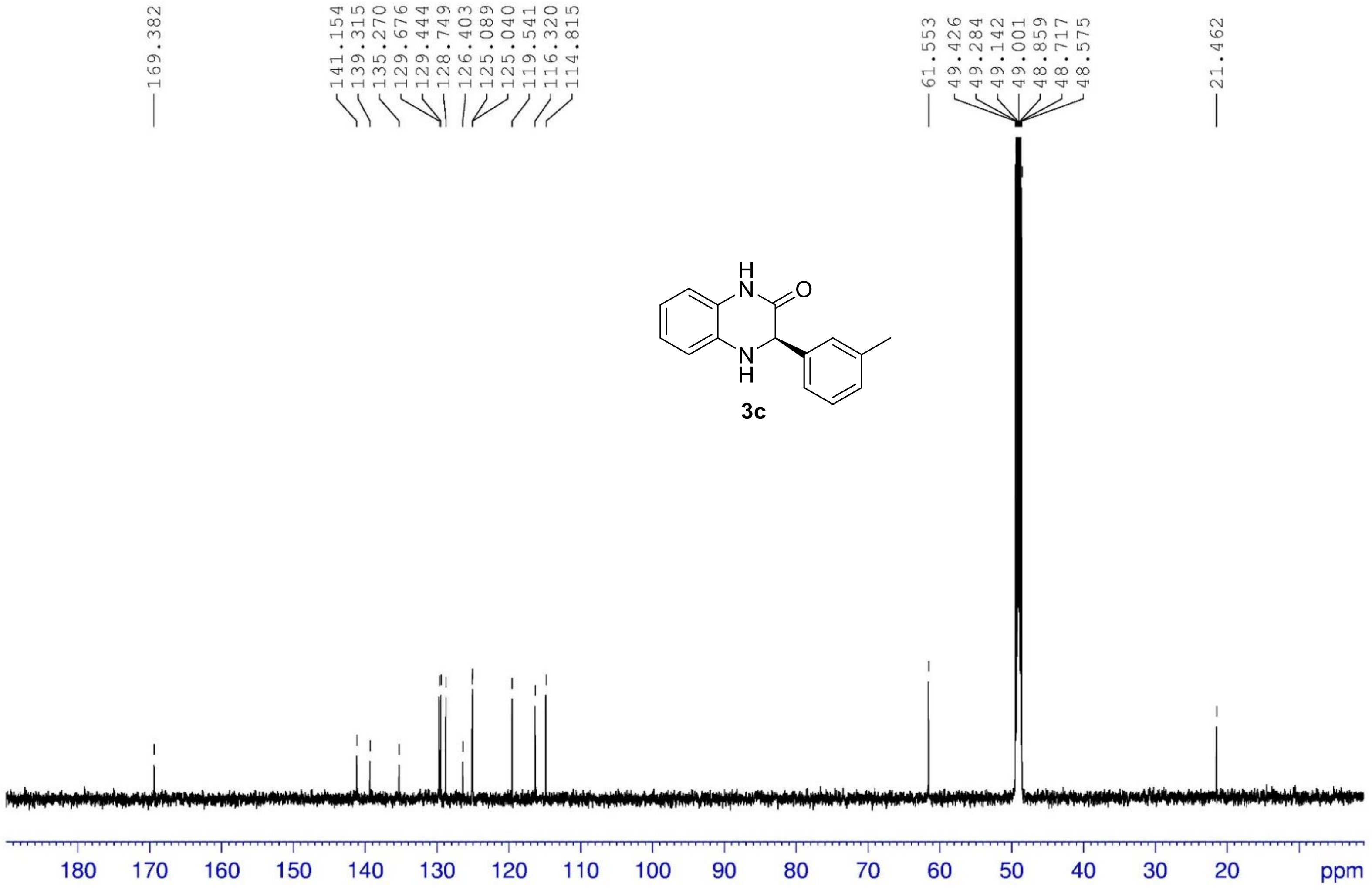

^{1}H NMR in $CDCl_3$ (400 MHz)

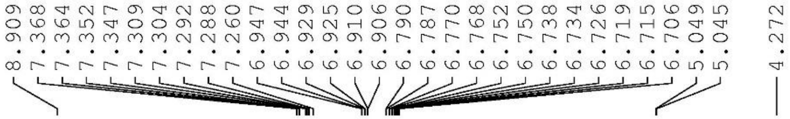

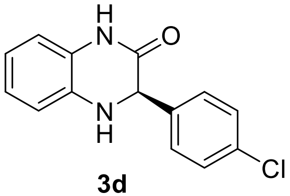

3d

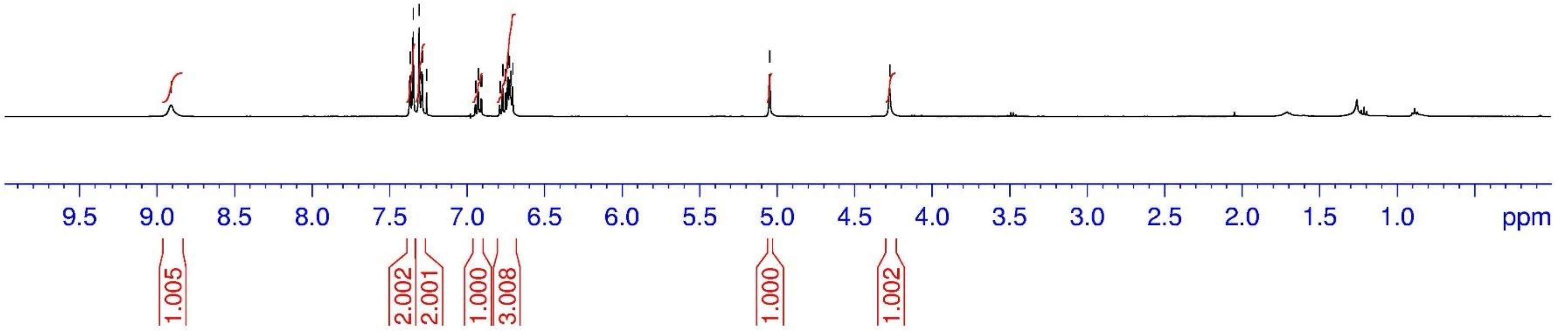

^{13}C NMR in $CDCl_3$ (100 MHz)

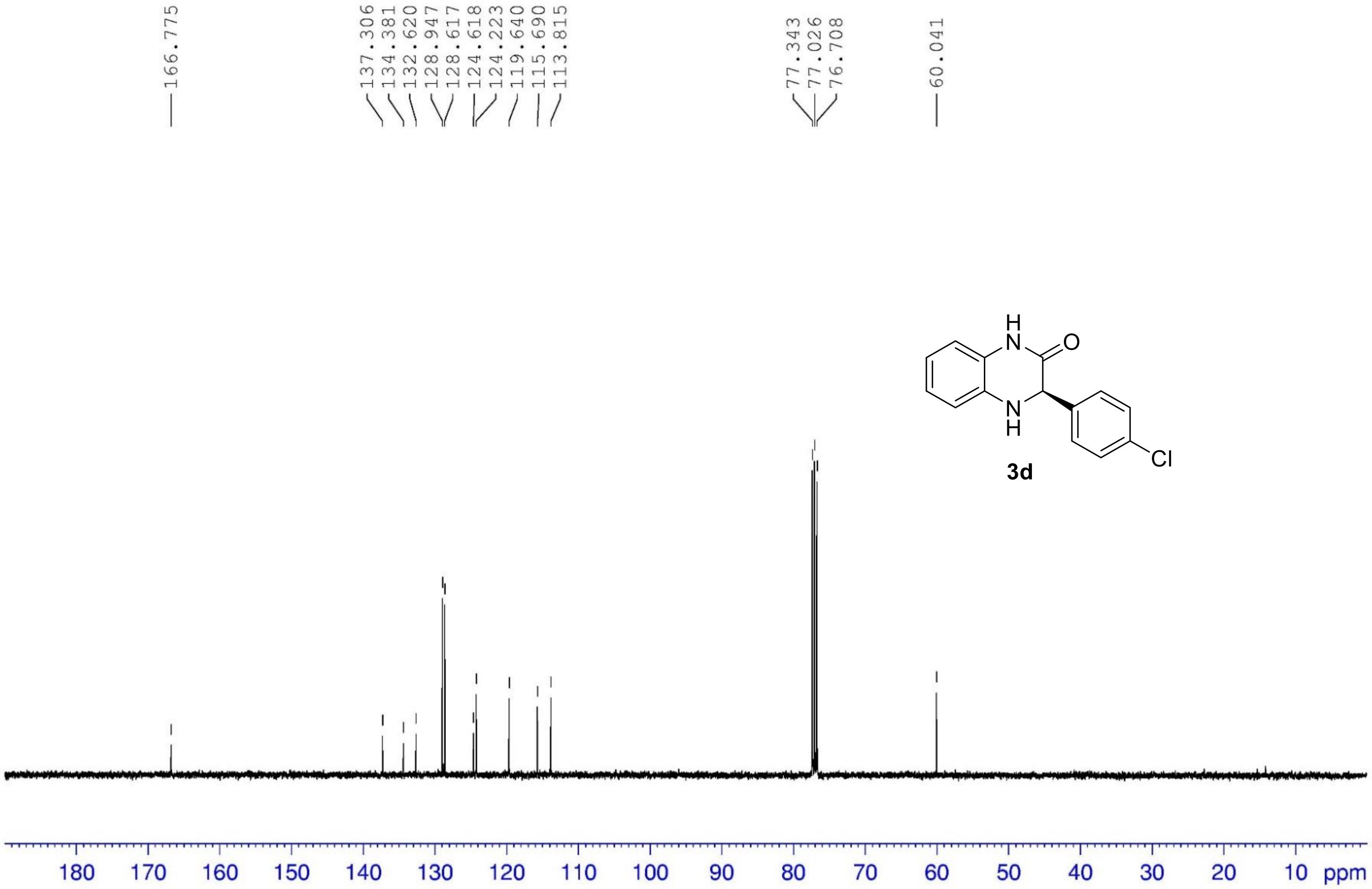

^{1}H NMR in $CDCl_3$ (400 MHz)

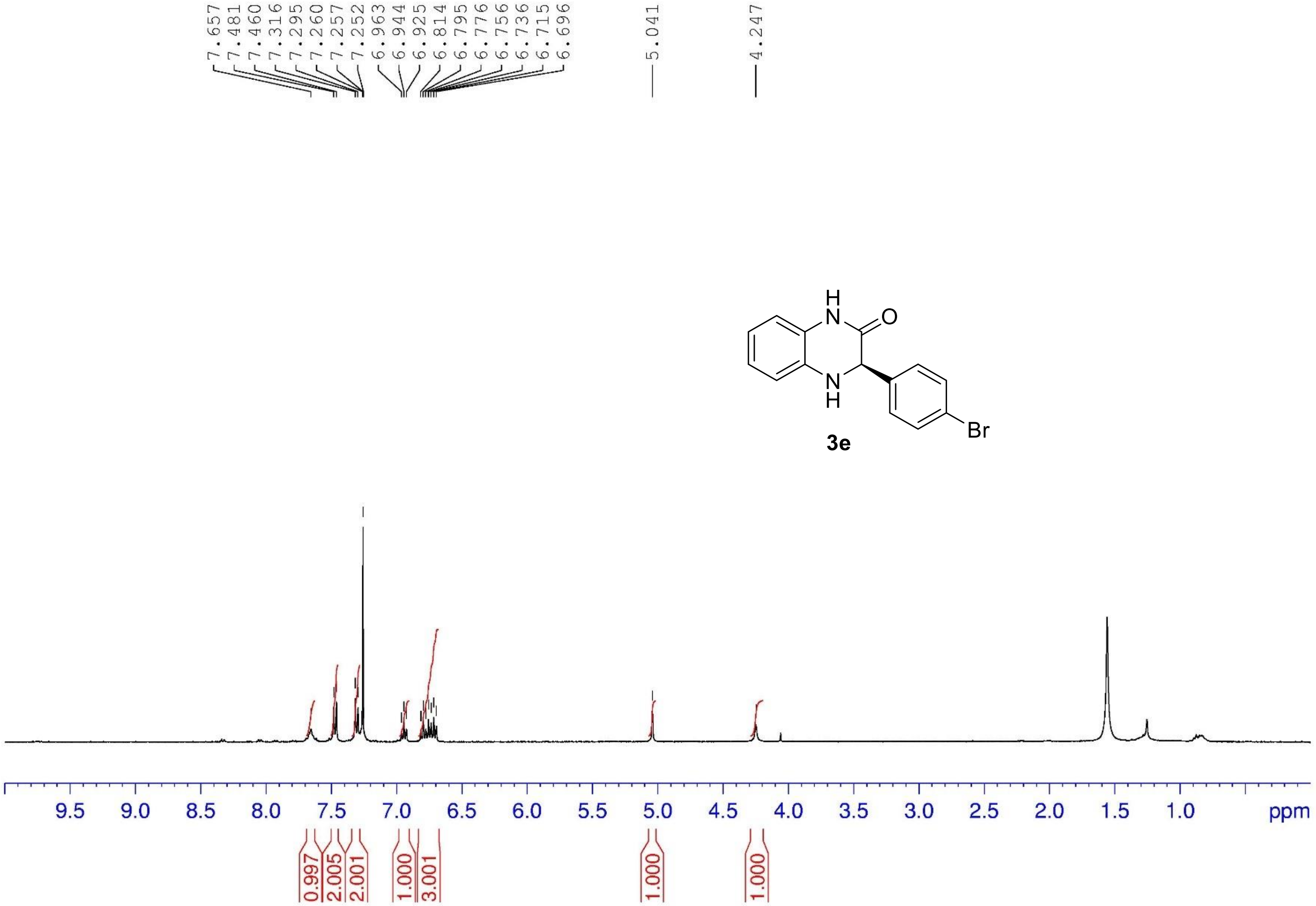

3e

^{13}C NMR in $CDCl_3$ (100 MHz)

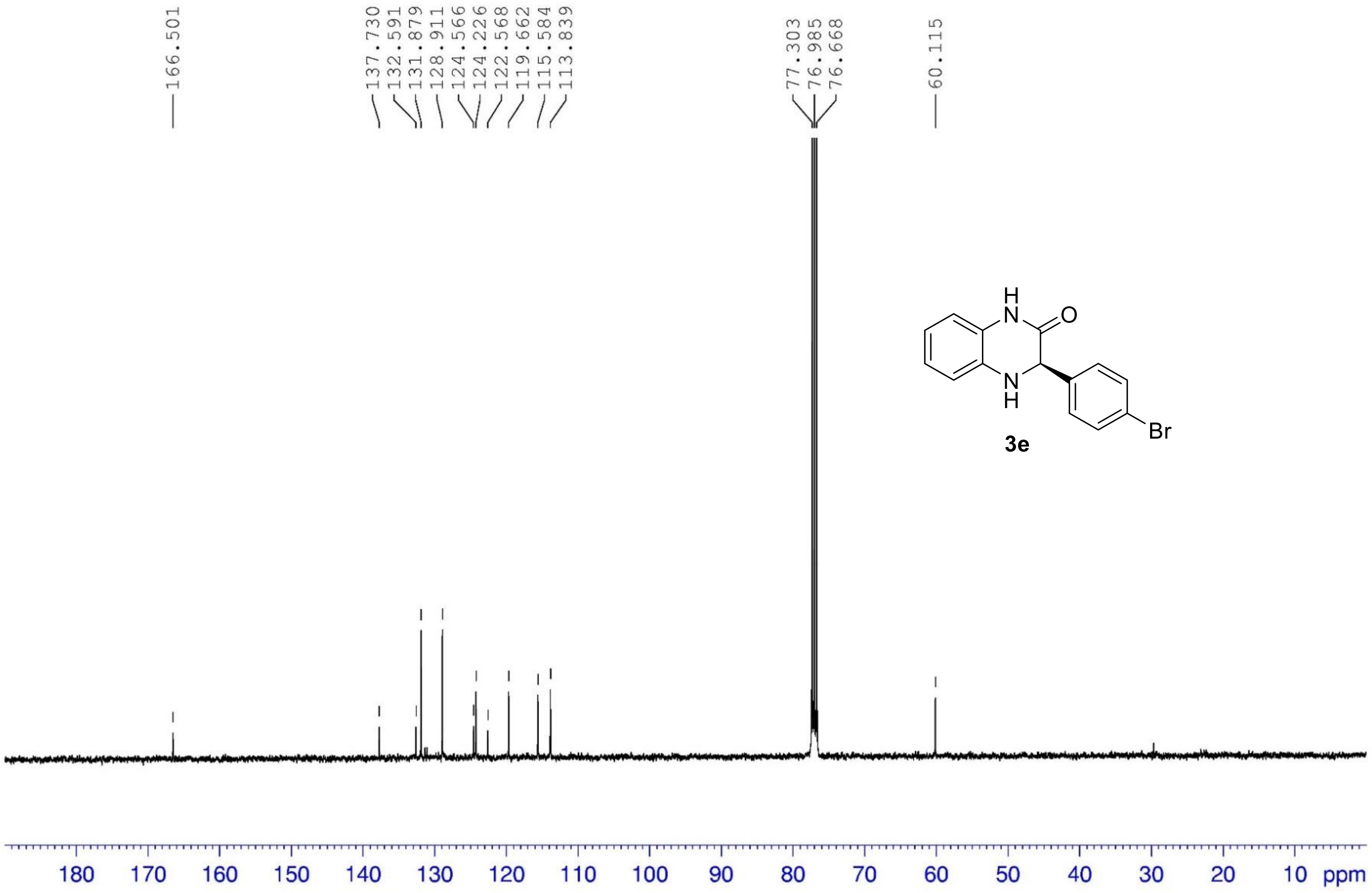

^{1}H NMR in $CDCl_3$ (400 MHz)

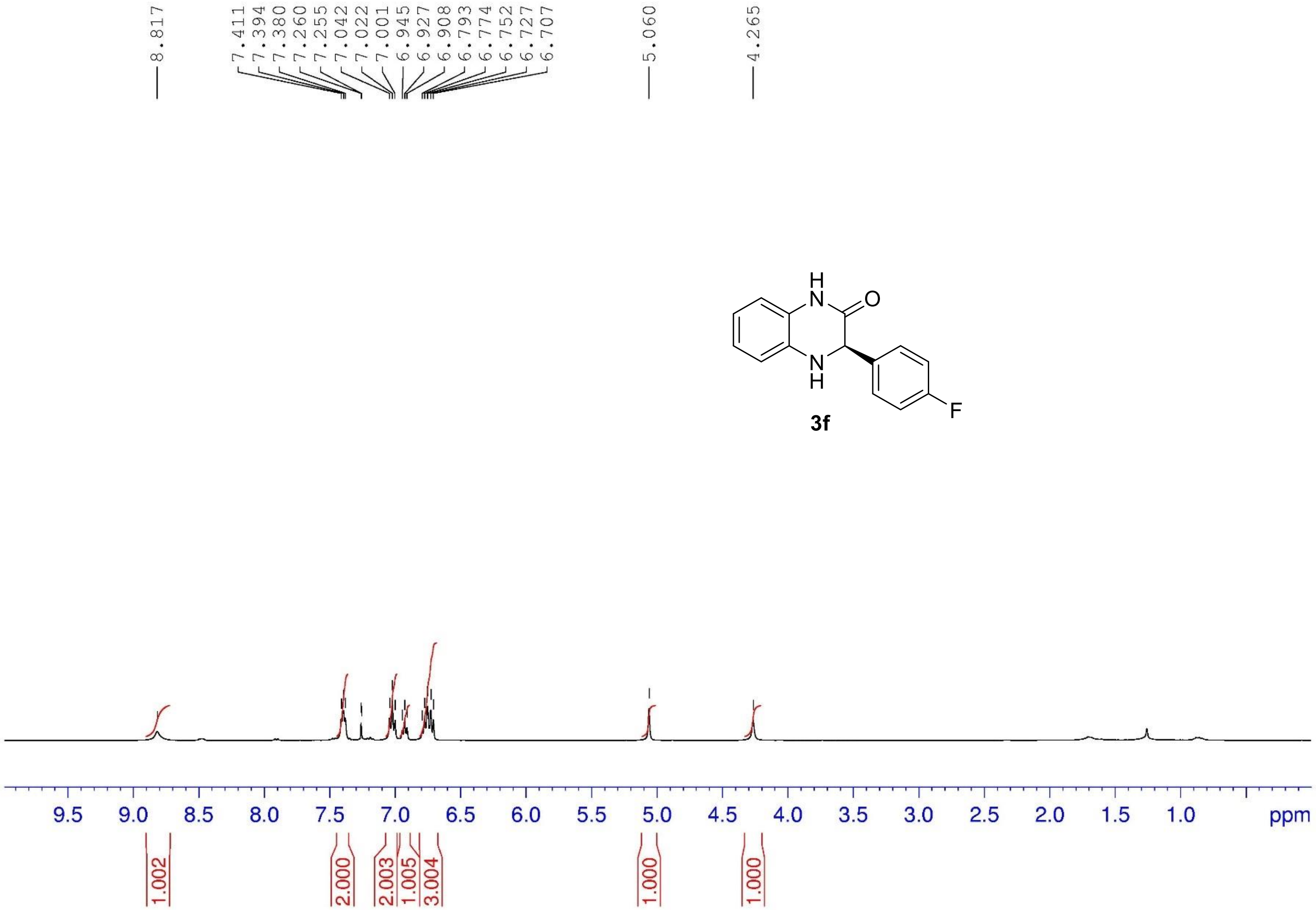

^{1}H NMR in $CDCl_3$ (400 MHz)

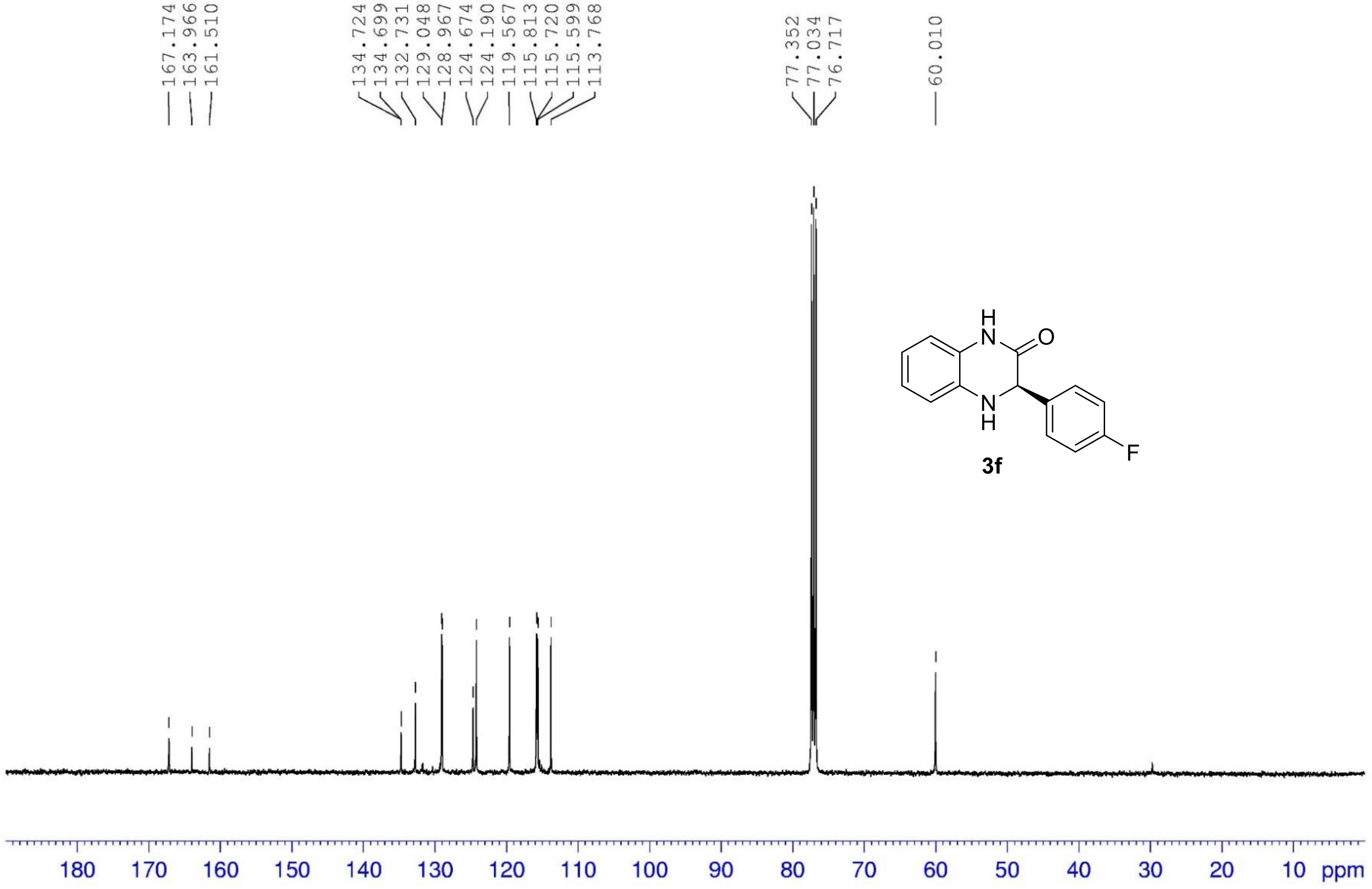

3f

^{1}H NMR in $CDCl_3$ (600 MHz)

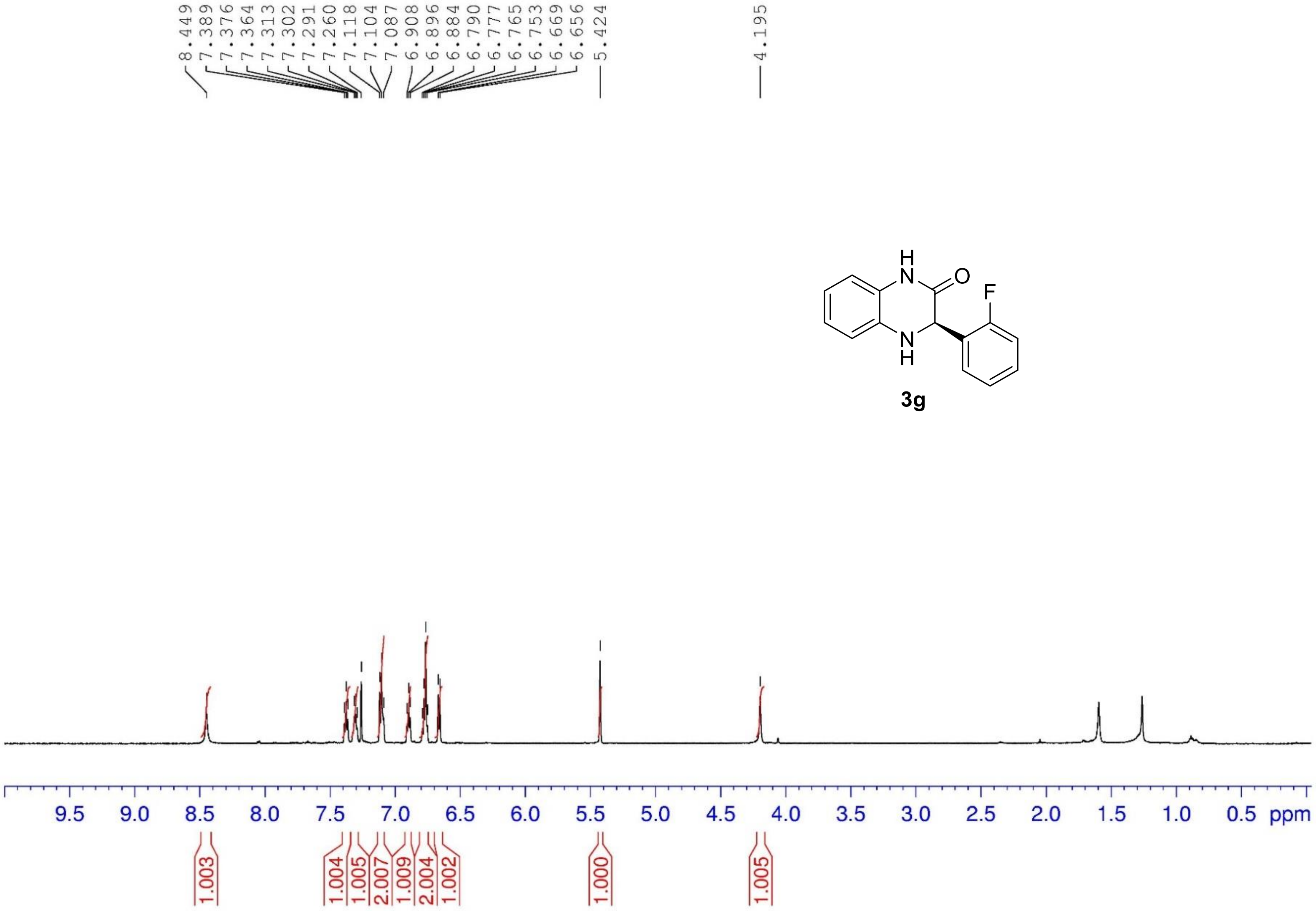

3g

^{13}C NMR in $CDCl_3$ (150 MHz)

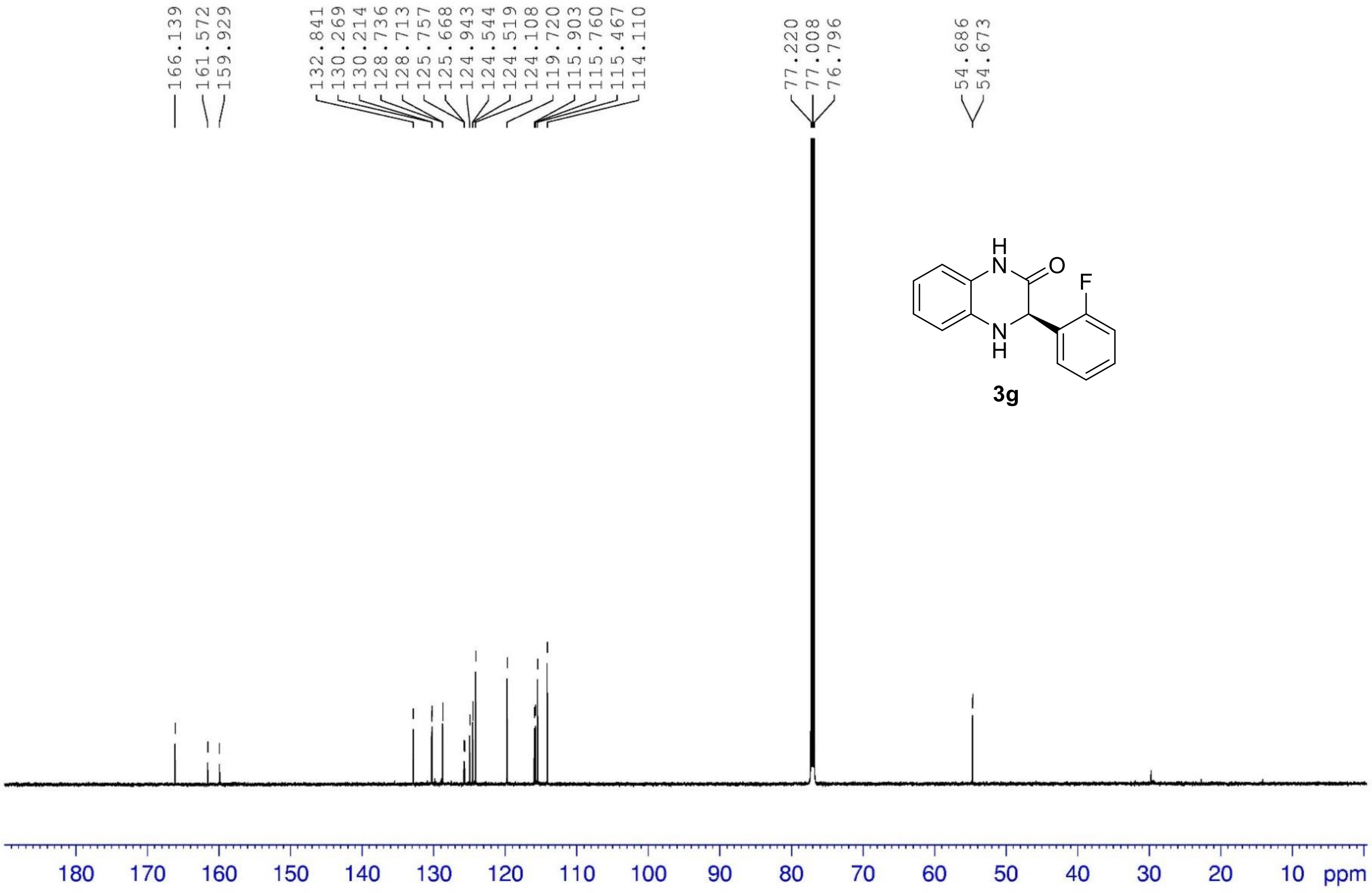

^{1}H NMR in $CDCl_3$ (400 MHz)

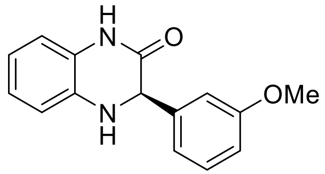

3h

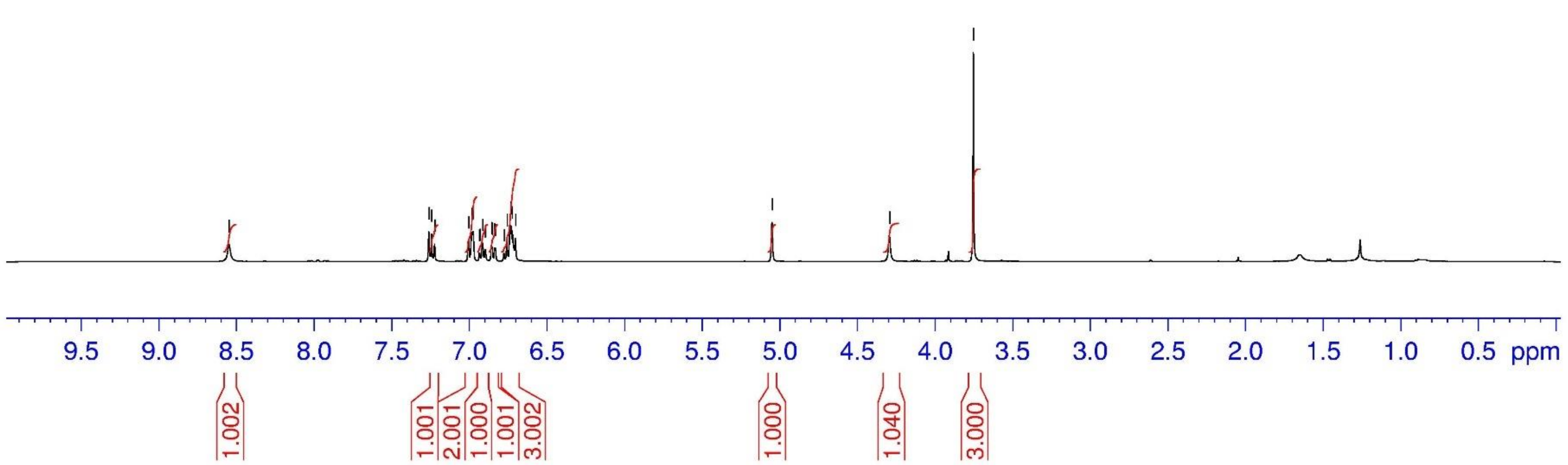

^{1}H NMR in $CDCl_3$ (100 MHz)

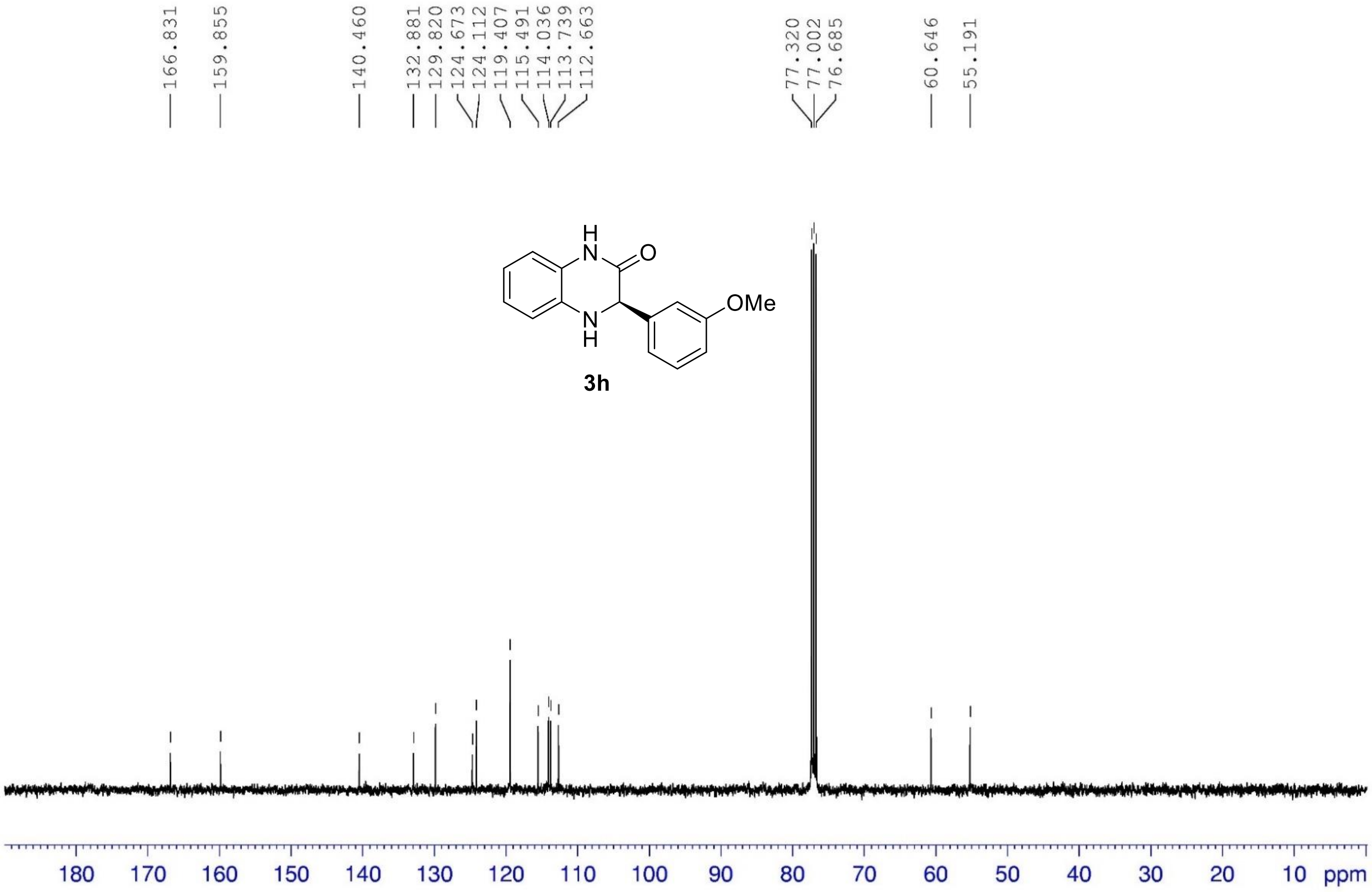

3h

^1H NMR in $CDCl_3$ (400 MHz)

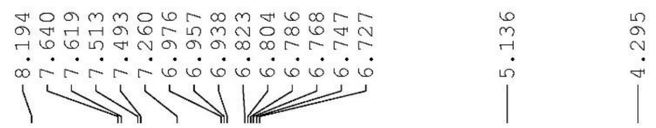

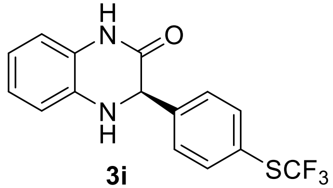

3i

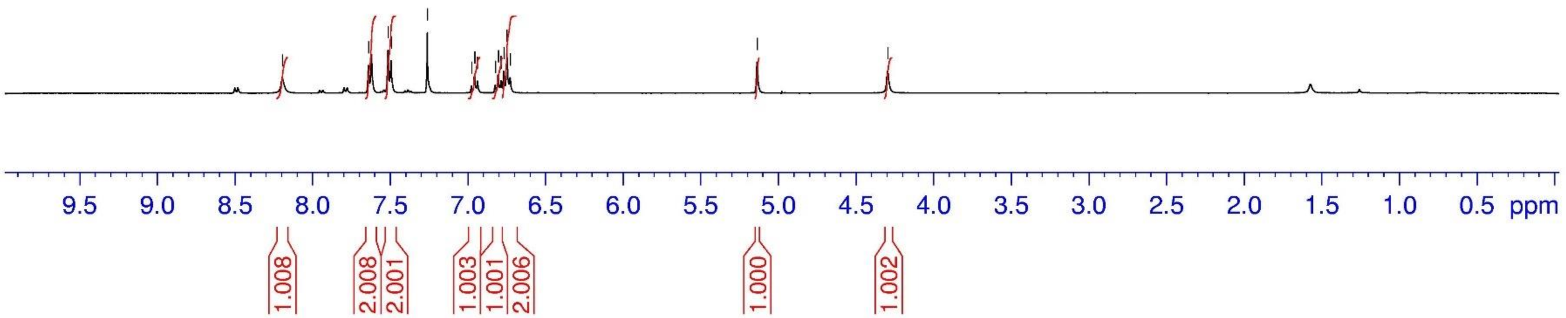

^{13}C NMR in $CDCl_3$ (150 MHz)

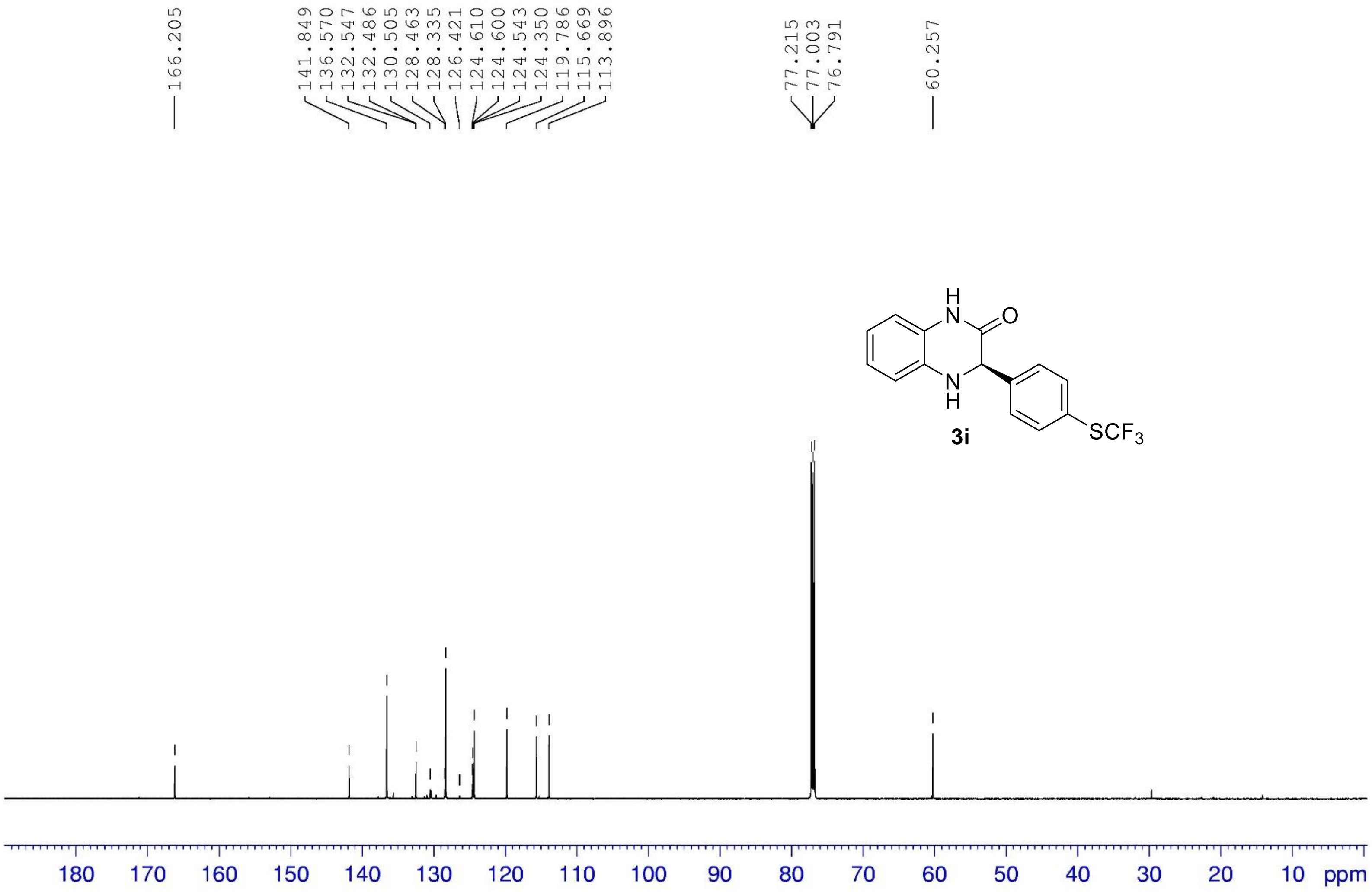

^{1}H NMR in $CDCl_3$ (400 MHz)

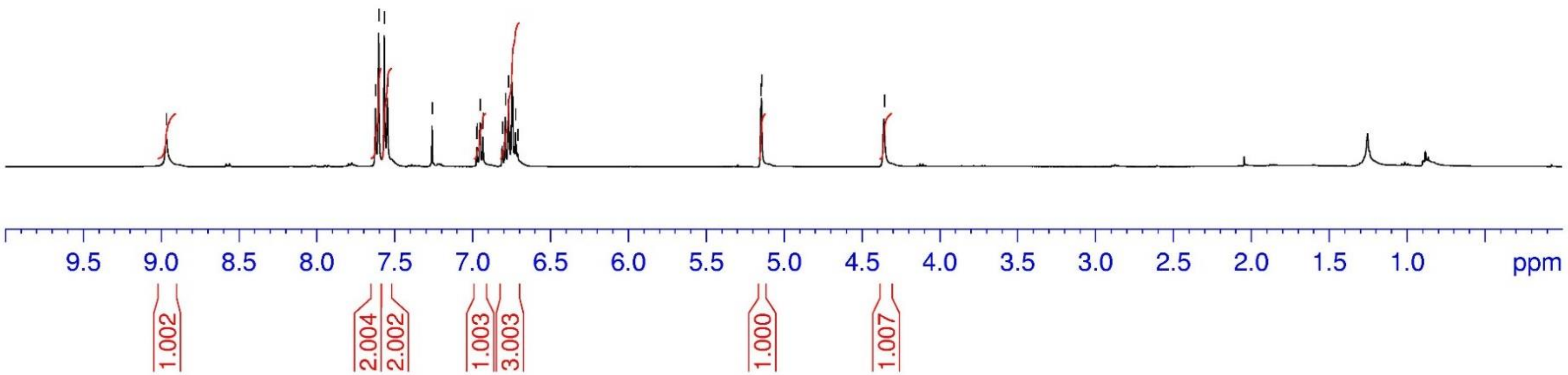

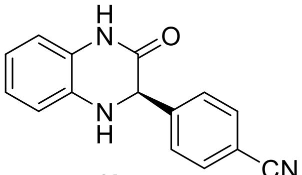

3j

^{13}C NMR in $CDCl_3$ (100 MHz)

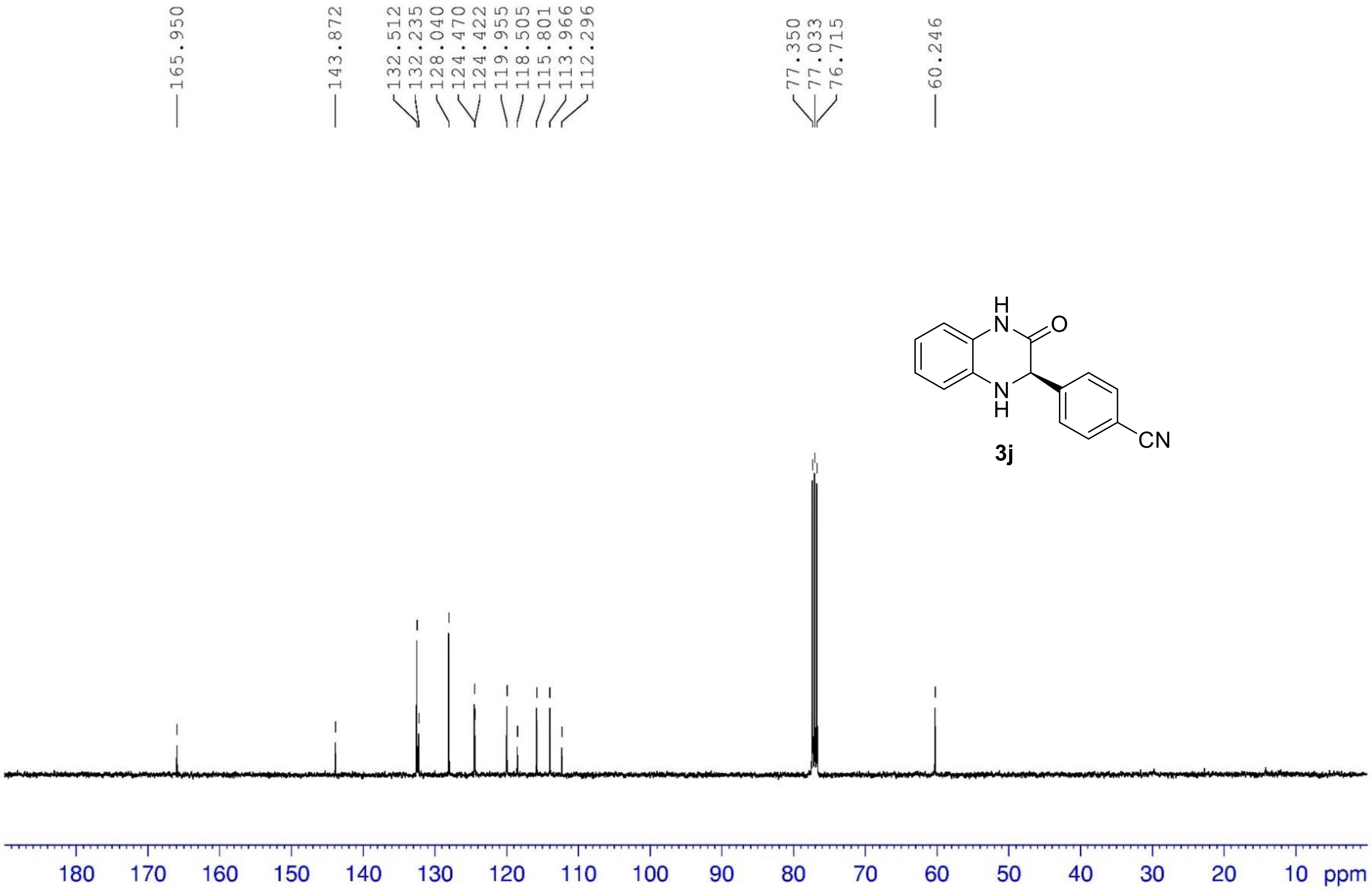

3j

^{1}H NMR in $CDCl_3$ (400 MHz)

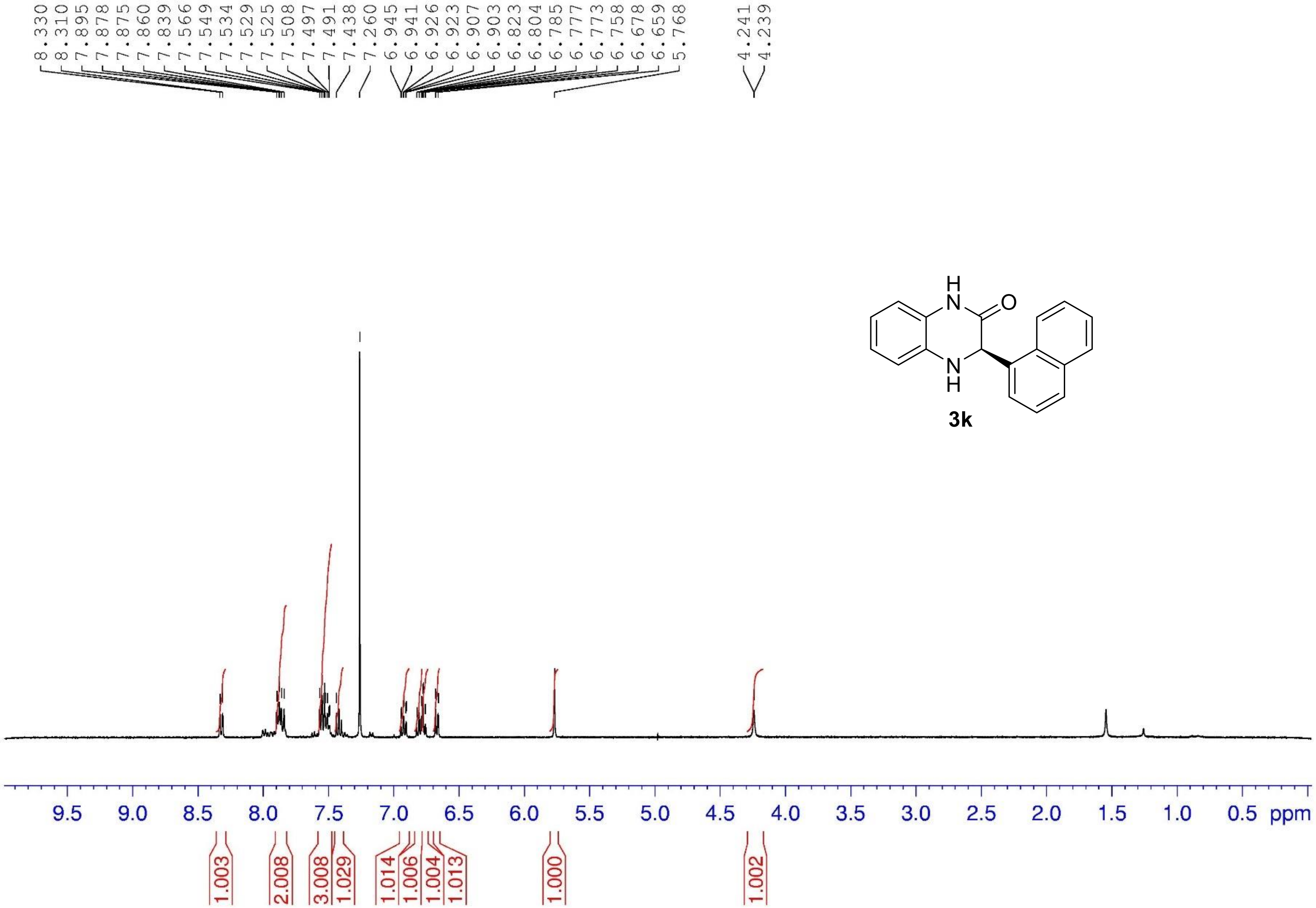

3k

^{13}C NMR in CDCl3 (150 MHz)

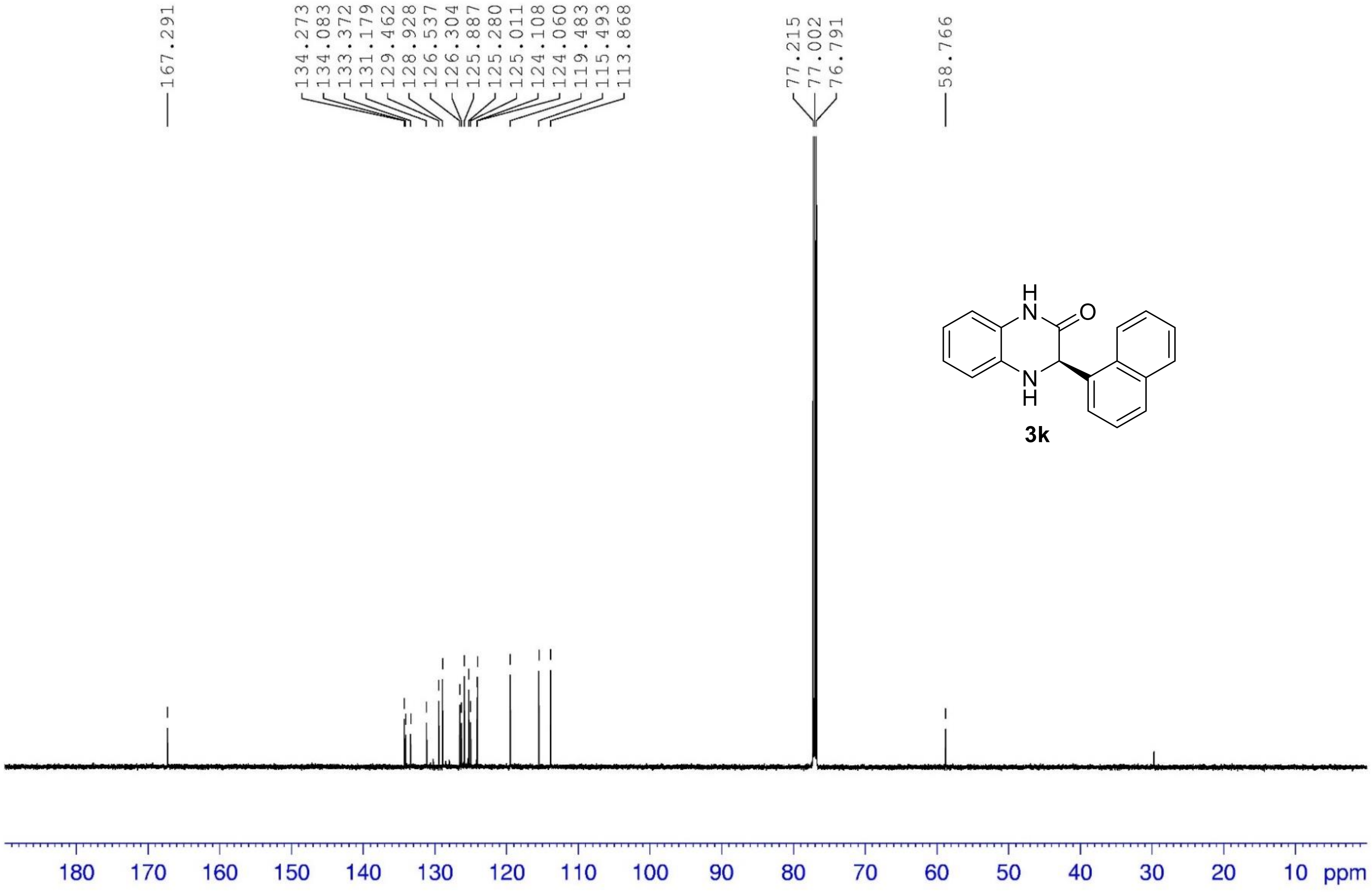

^{1}H NMR in MeOD (600 MHz)

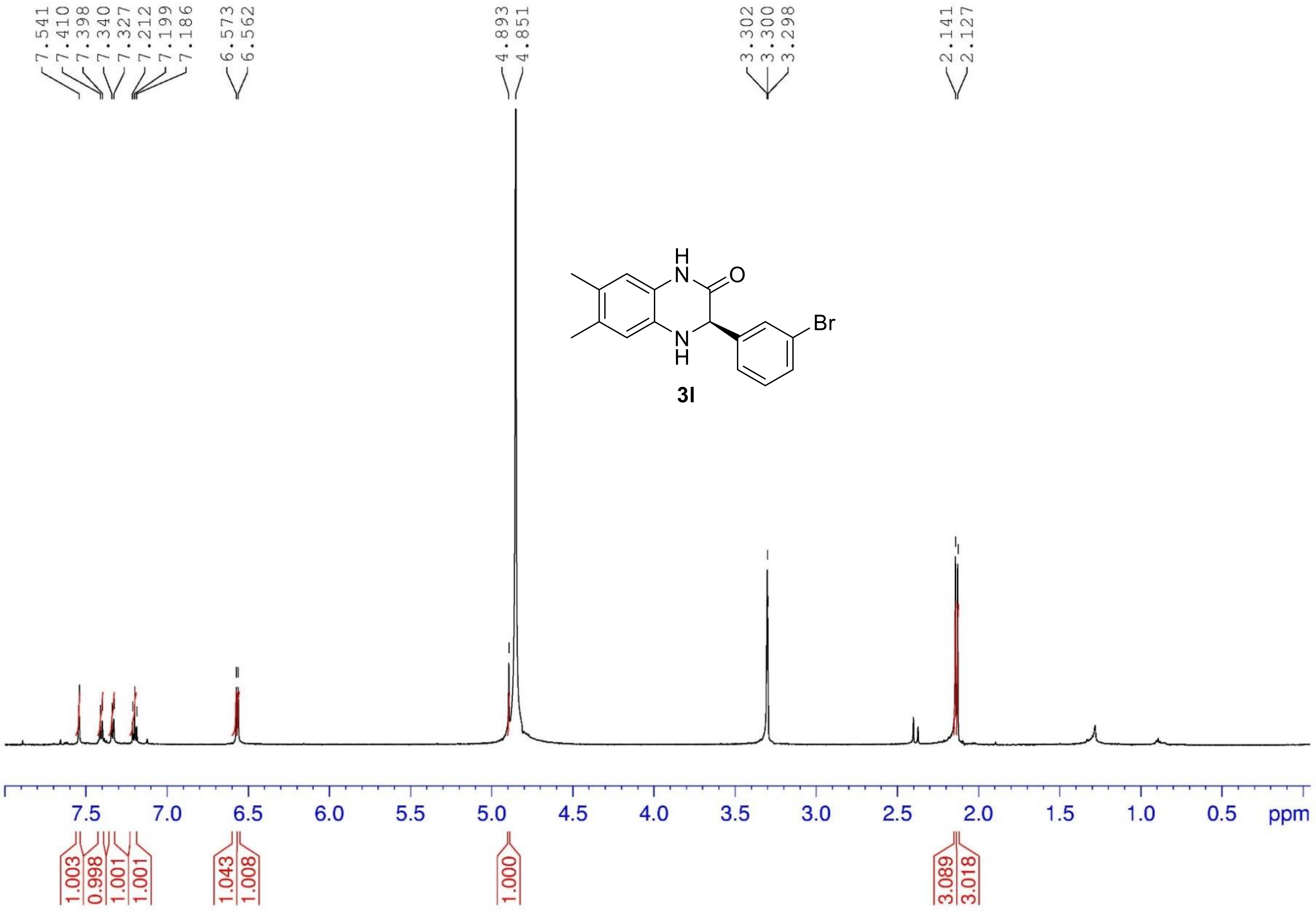

^{13}C NMR in MeOD (150 MHz)

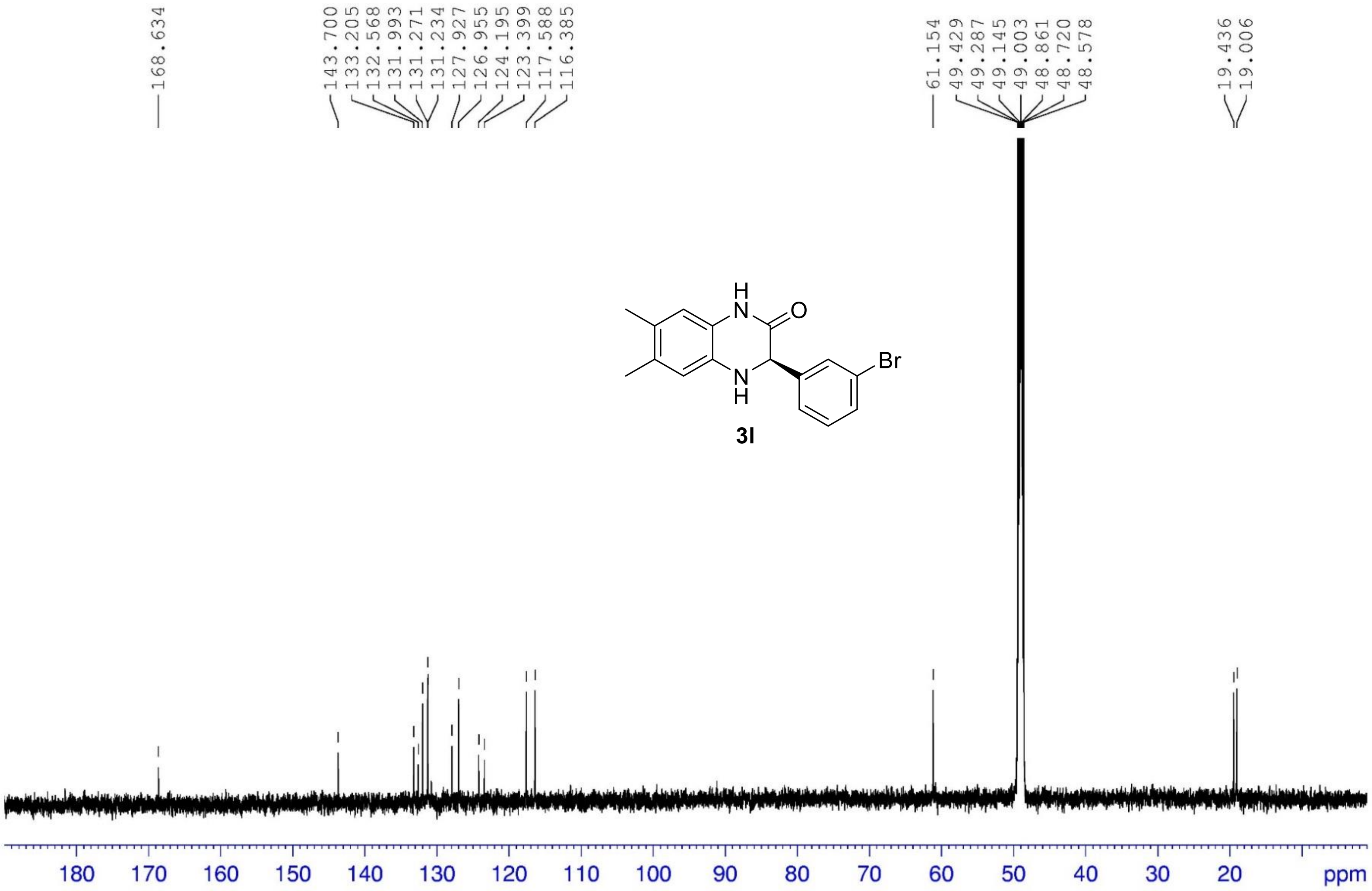

^{1}H NMR in MeOD (600 MHz)

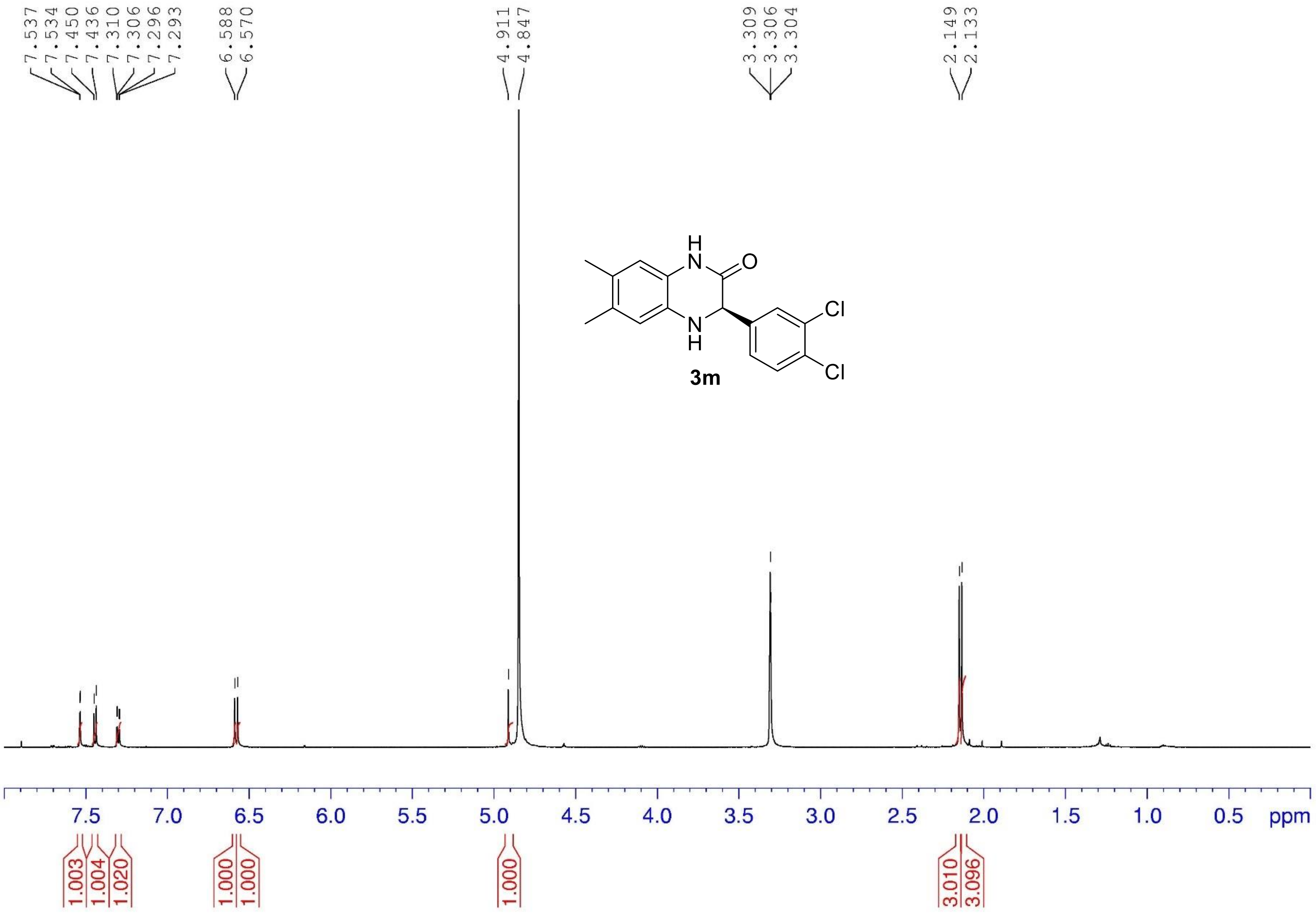

^{13}C NMR in MeOD (150 MHz)

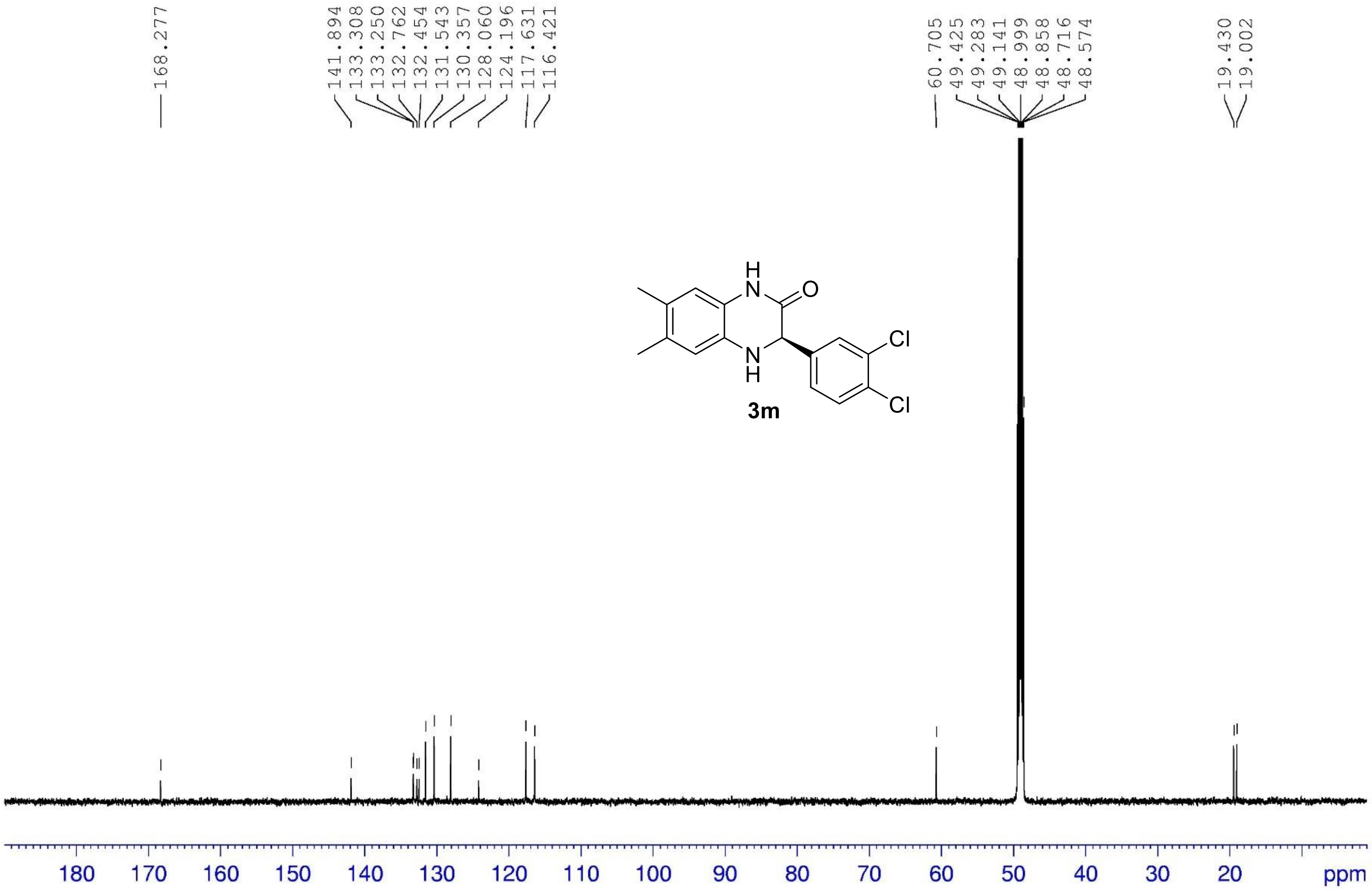

^{1}H NMR in MeOD (400 MHz)

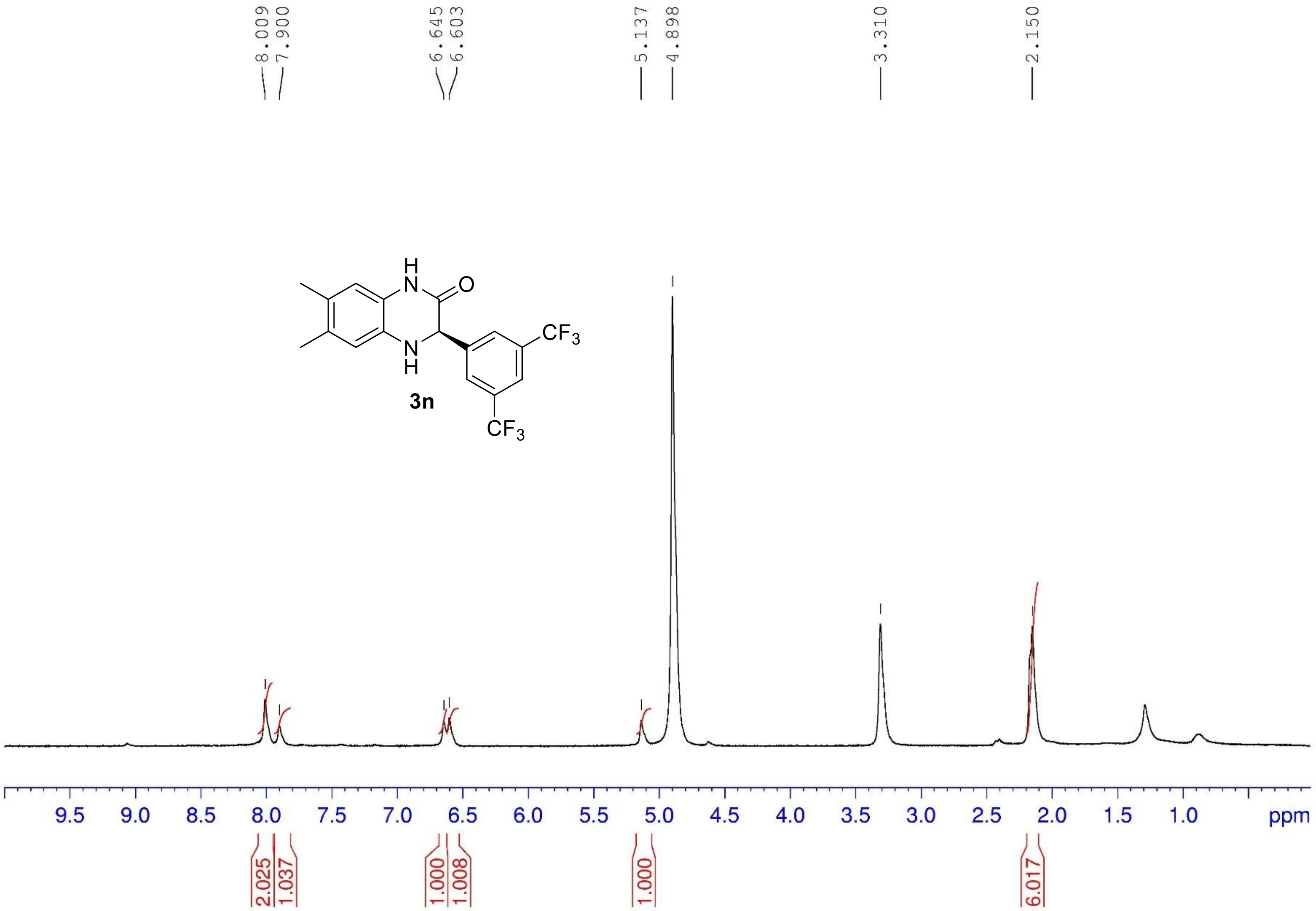

^{13}C NMR in MeOD (100 MHz)

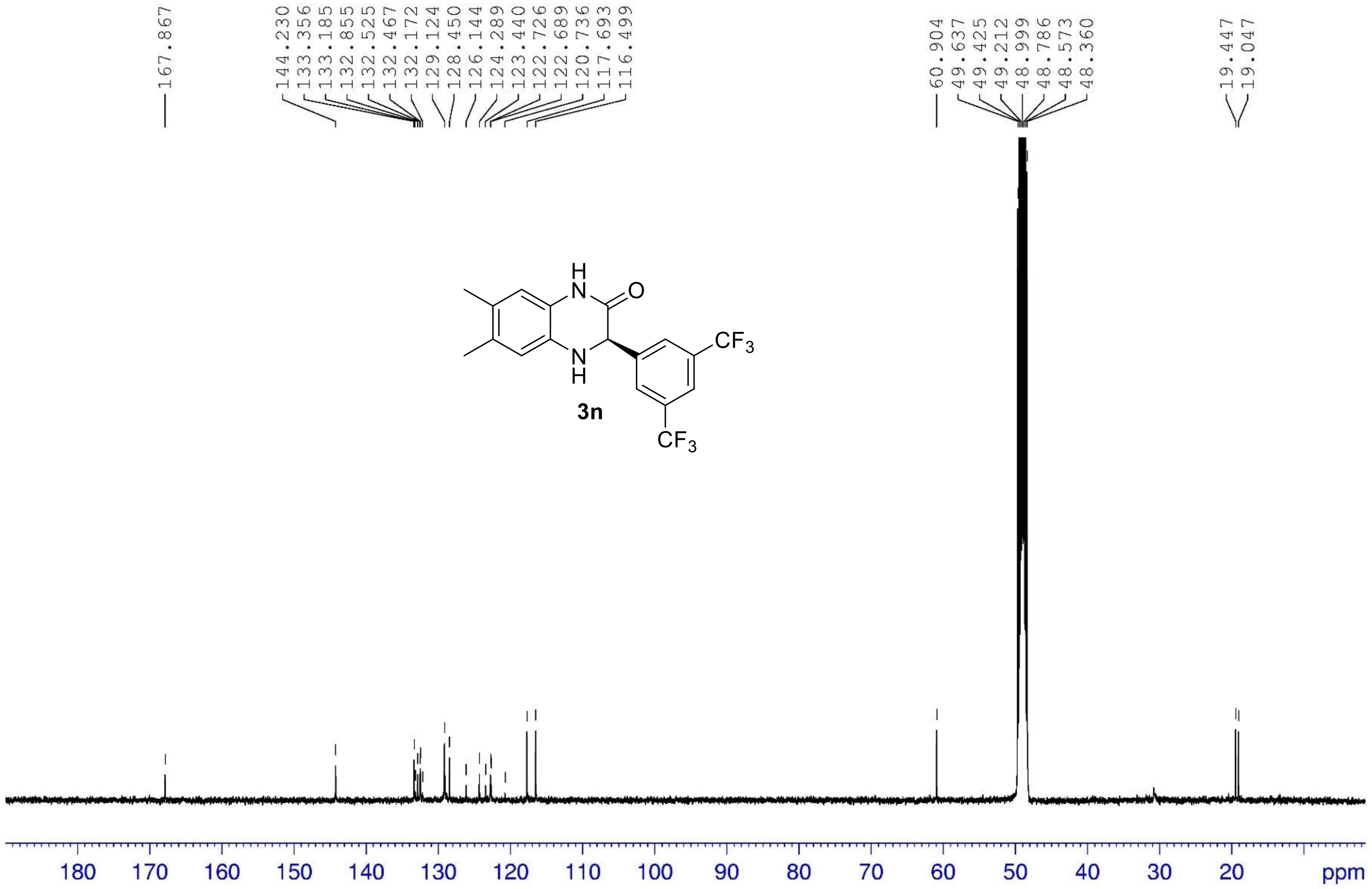

^{1}H NMR in $CDCl_3$ (400 MHz)

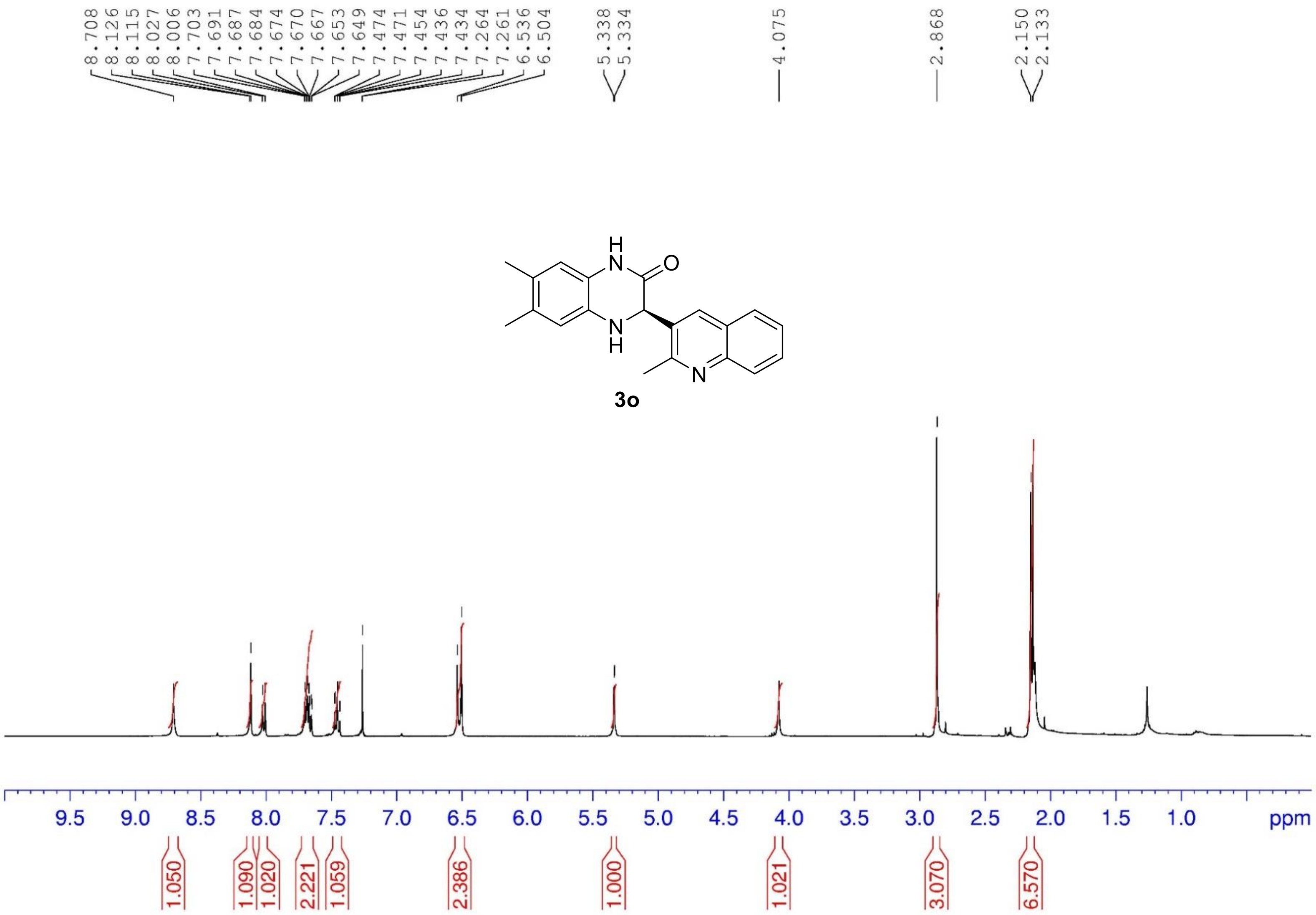

^{13}C NMR in $CDCl_3$ (75 MHz)

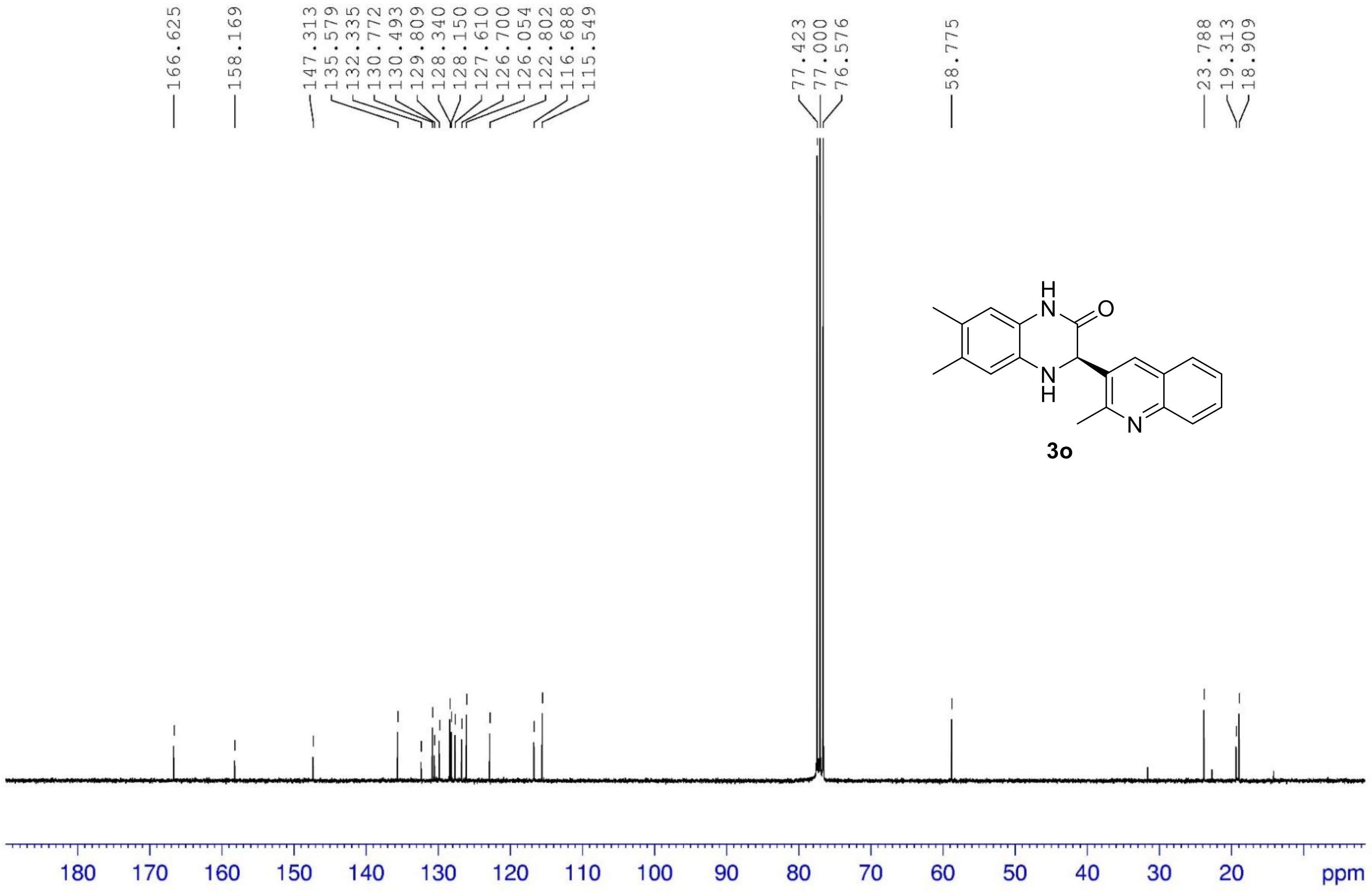

^{1}H NMR in MeOD (400 MHz)

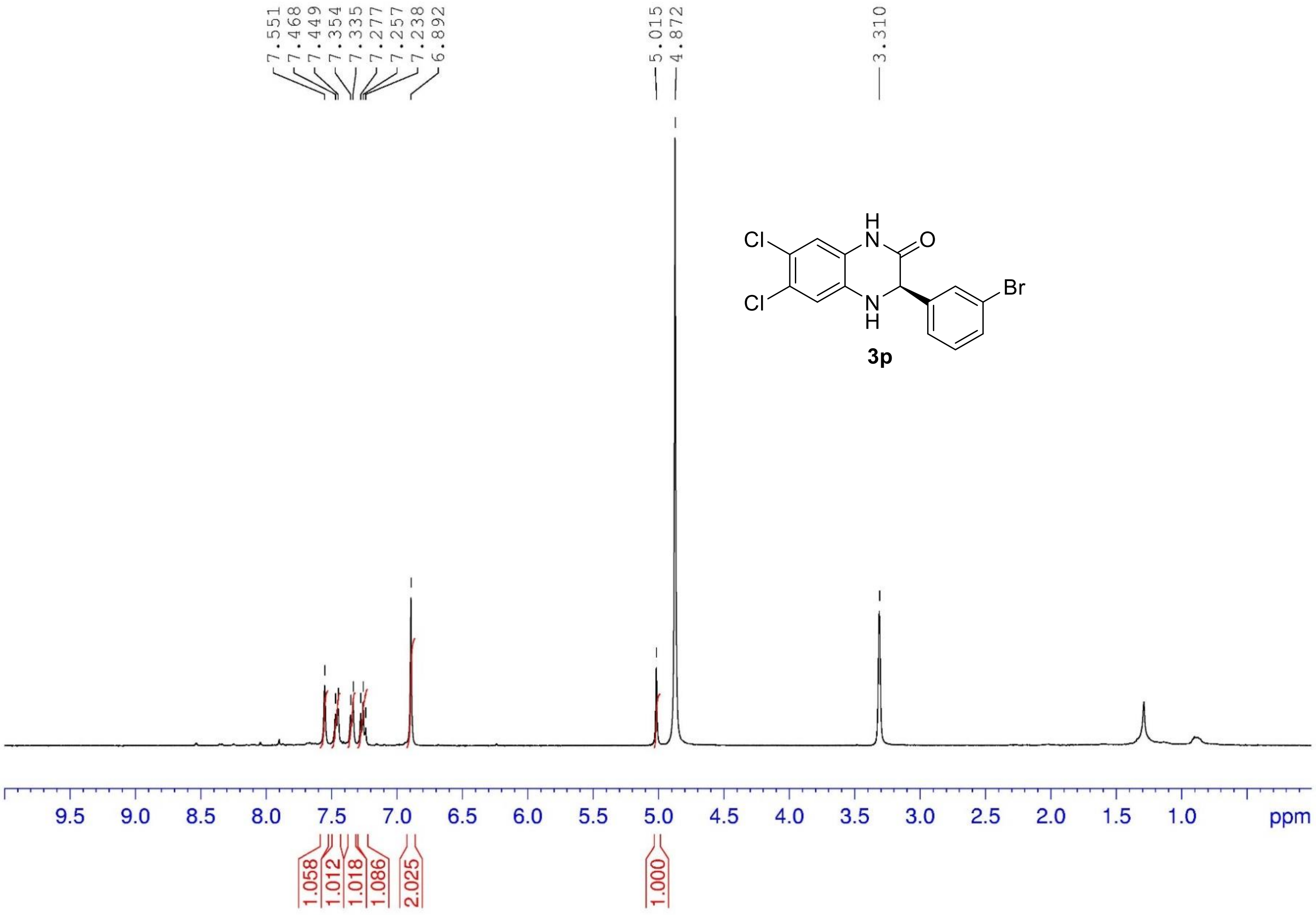

^{13}C NMR in MeOD (100 MHz)

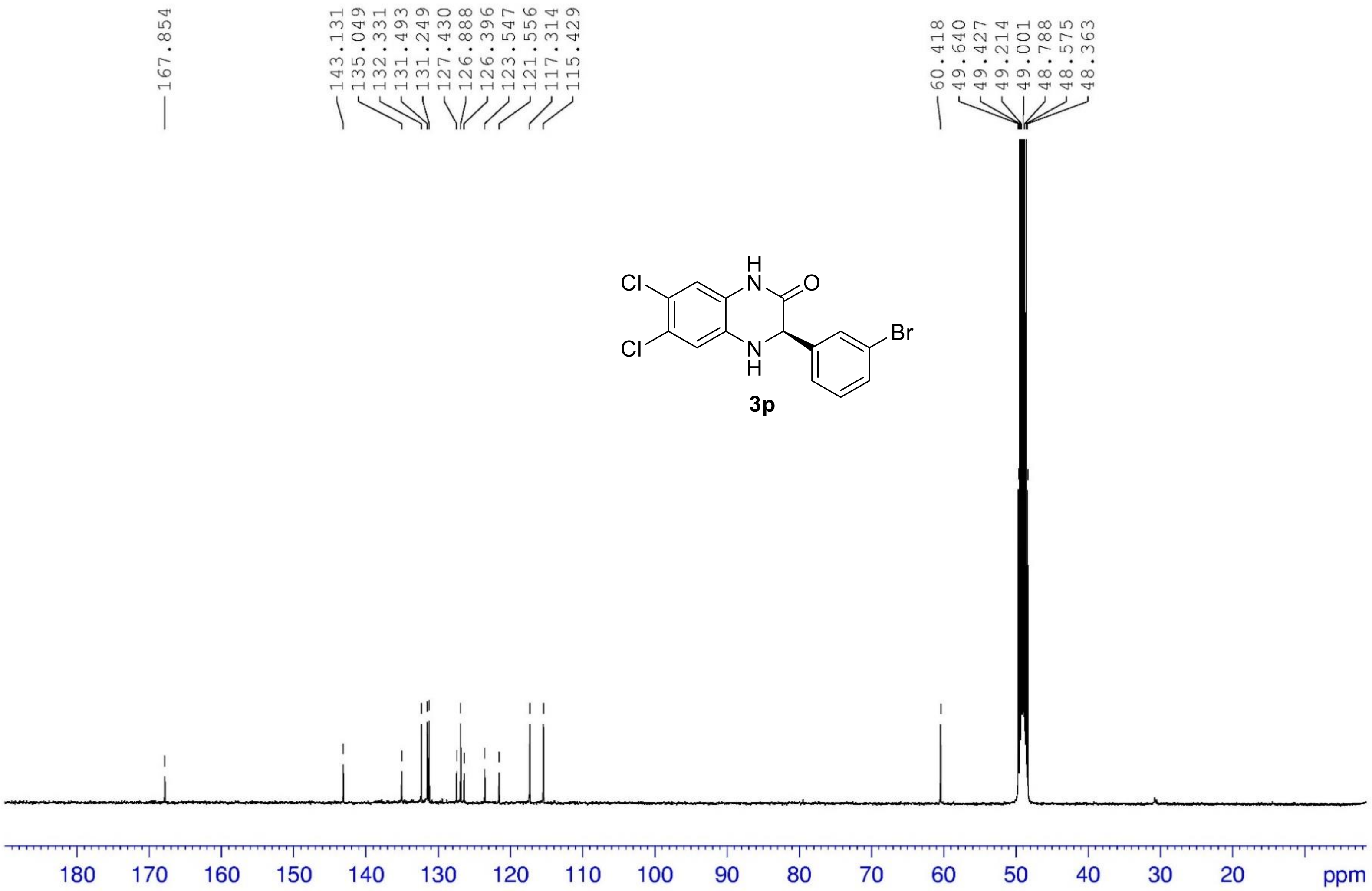

^{1}H NMR in MeOD (300 MHz)

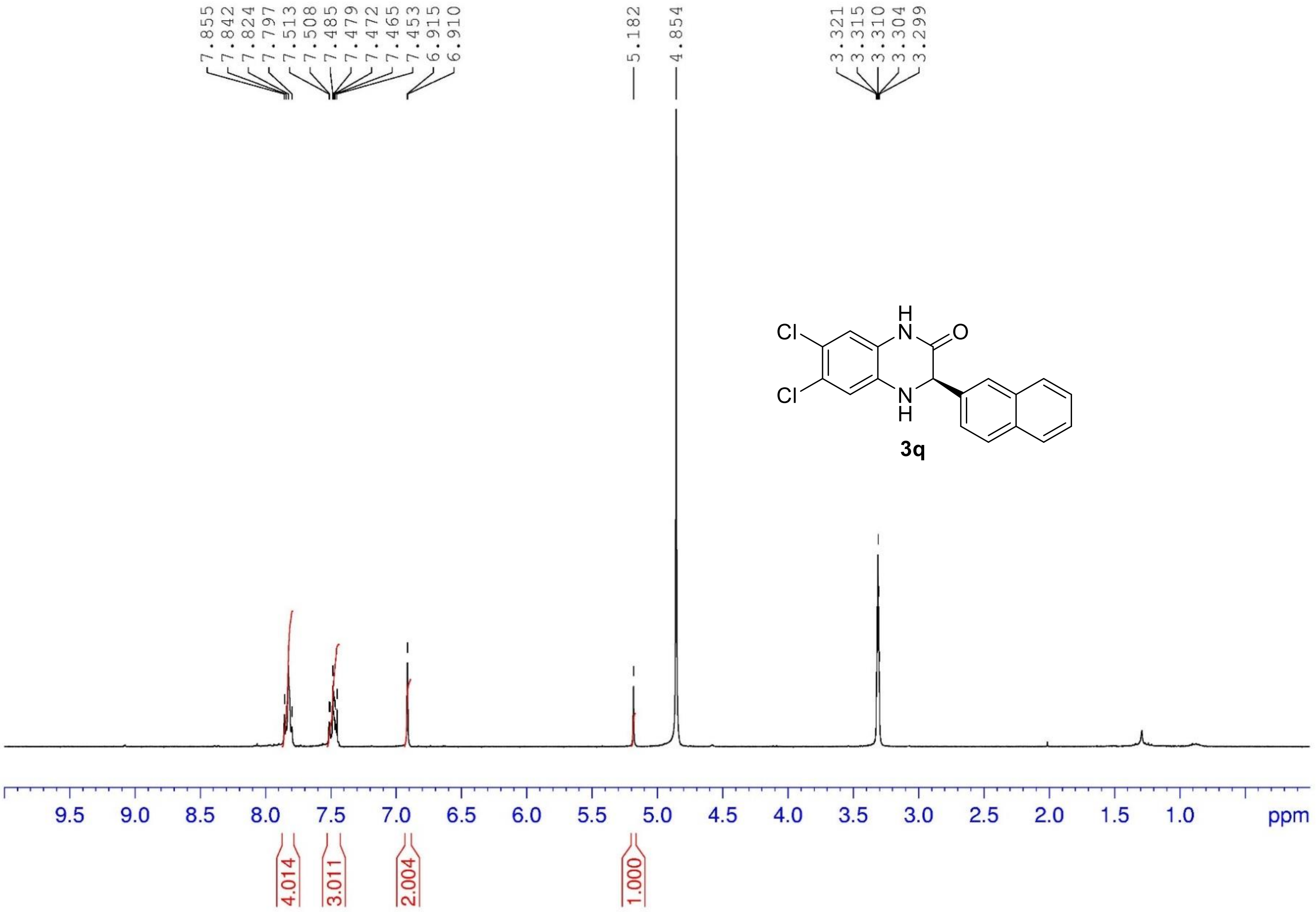

3q

^{13}C NMR in MeOD (150 MHz)

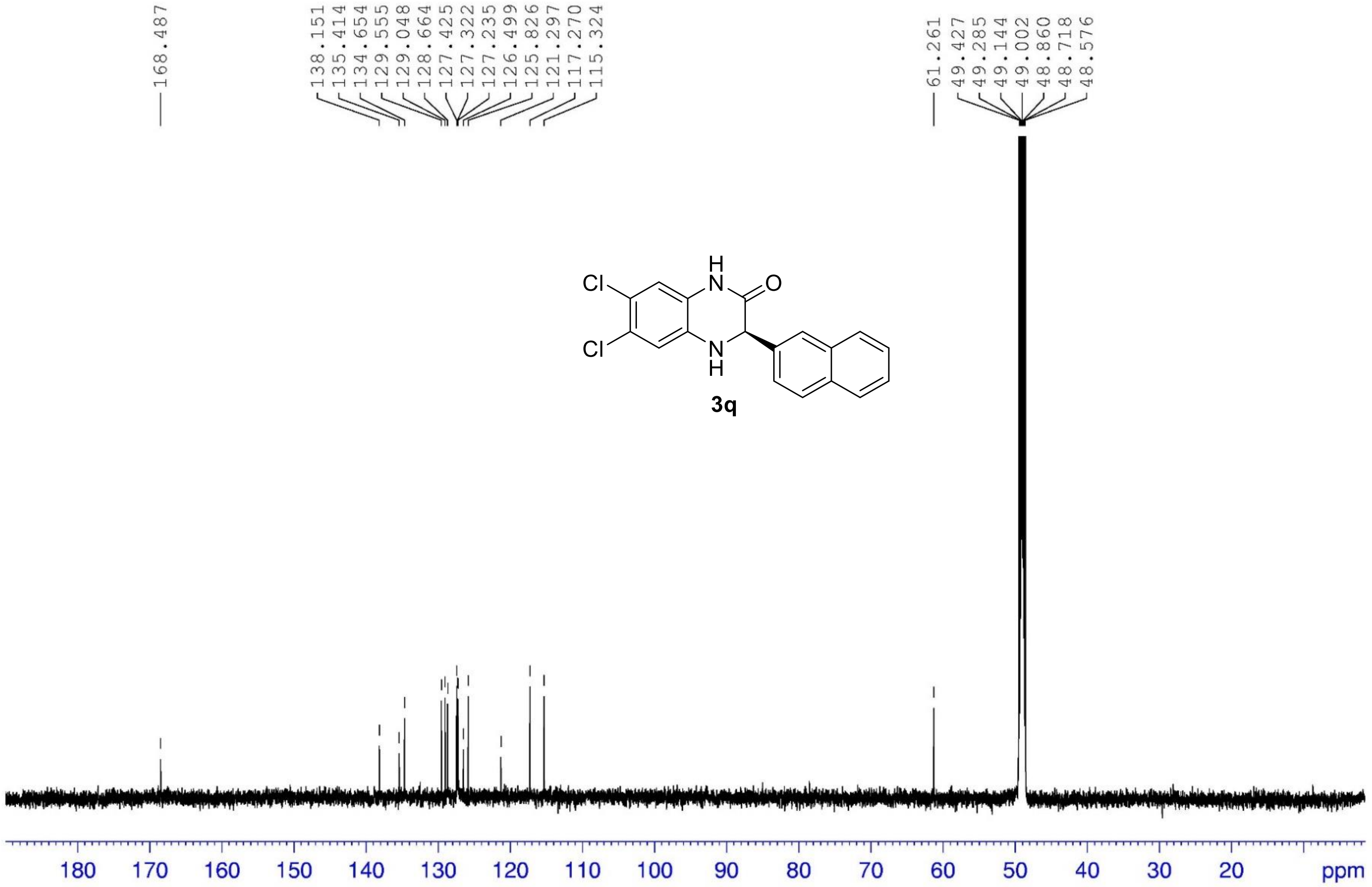

^{1}H NMR in $CDCl_3$ (400 MHz)

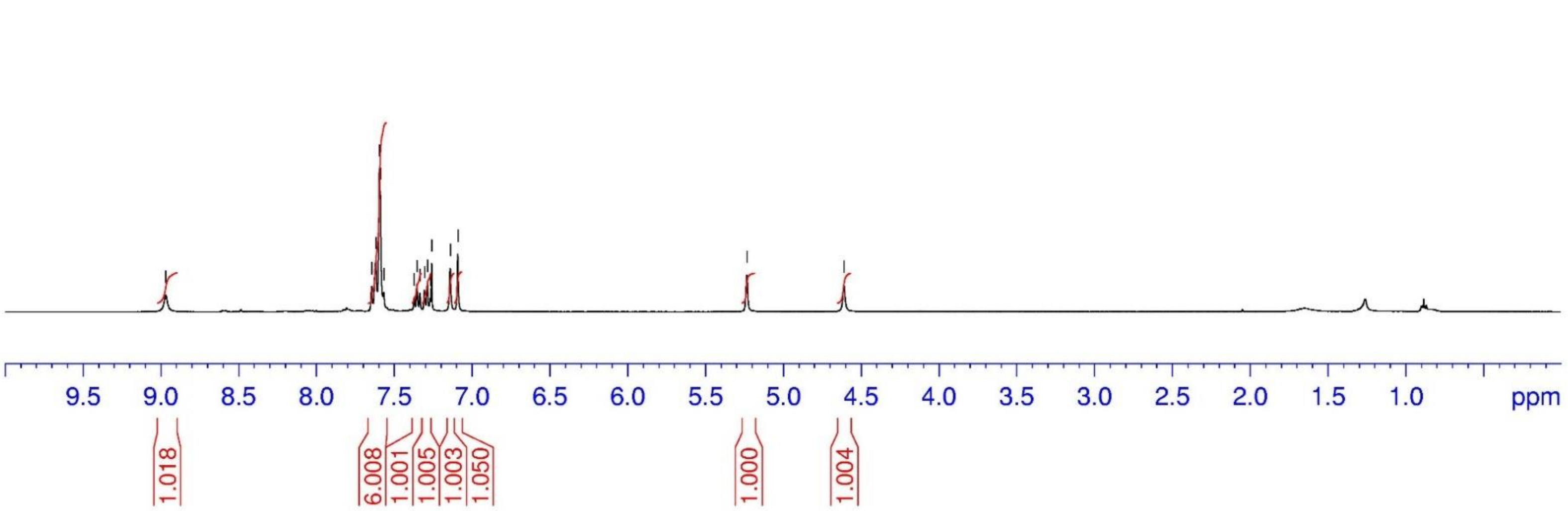

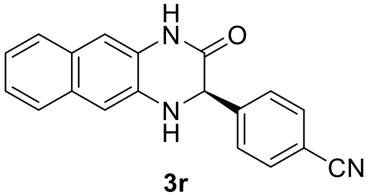

3r

^{13}C NMR in $CDCl_3$ (100 MHz)

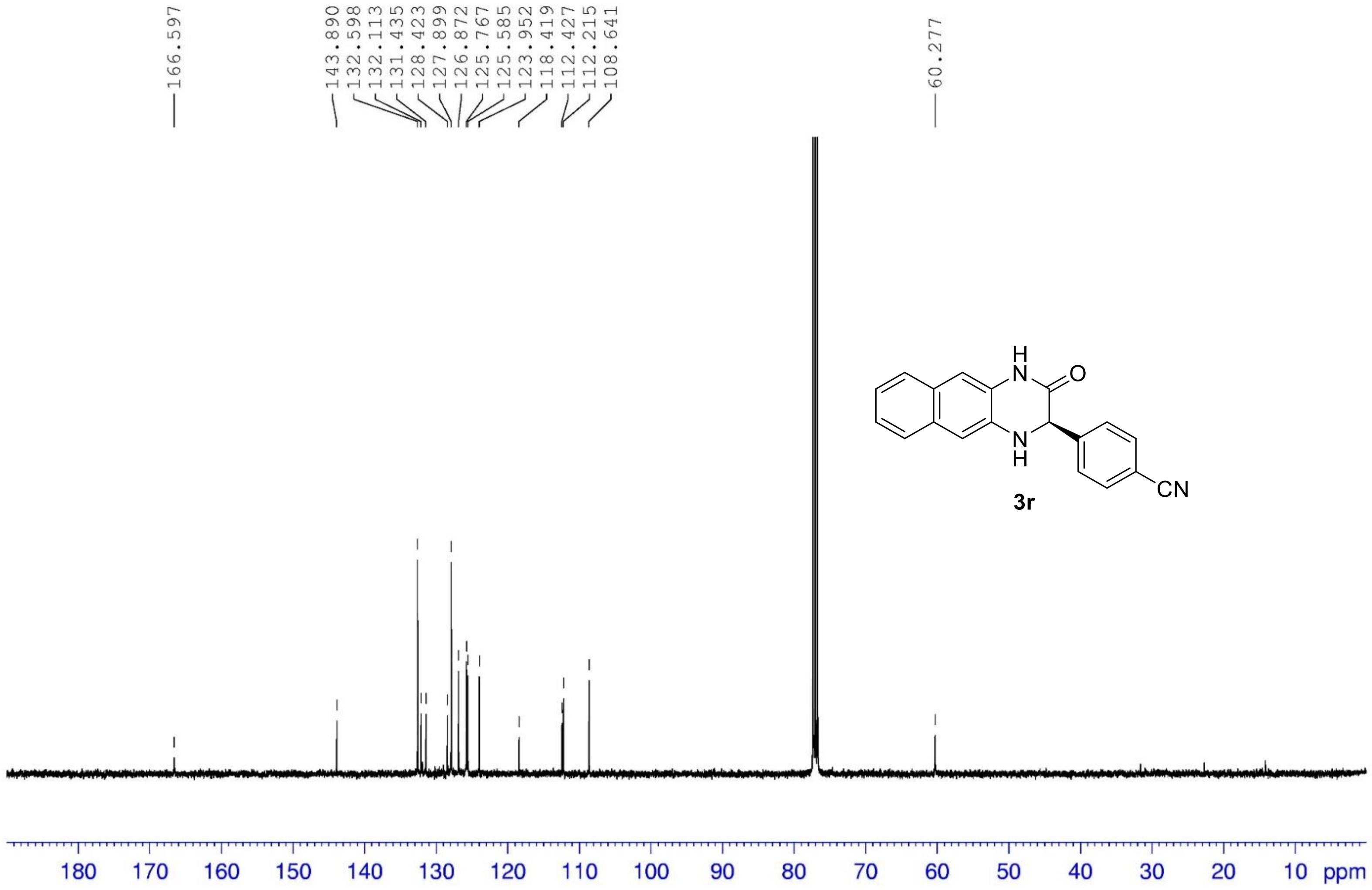

^{1}H NMR in $CDCl_3$ (400 MHz)

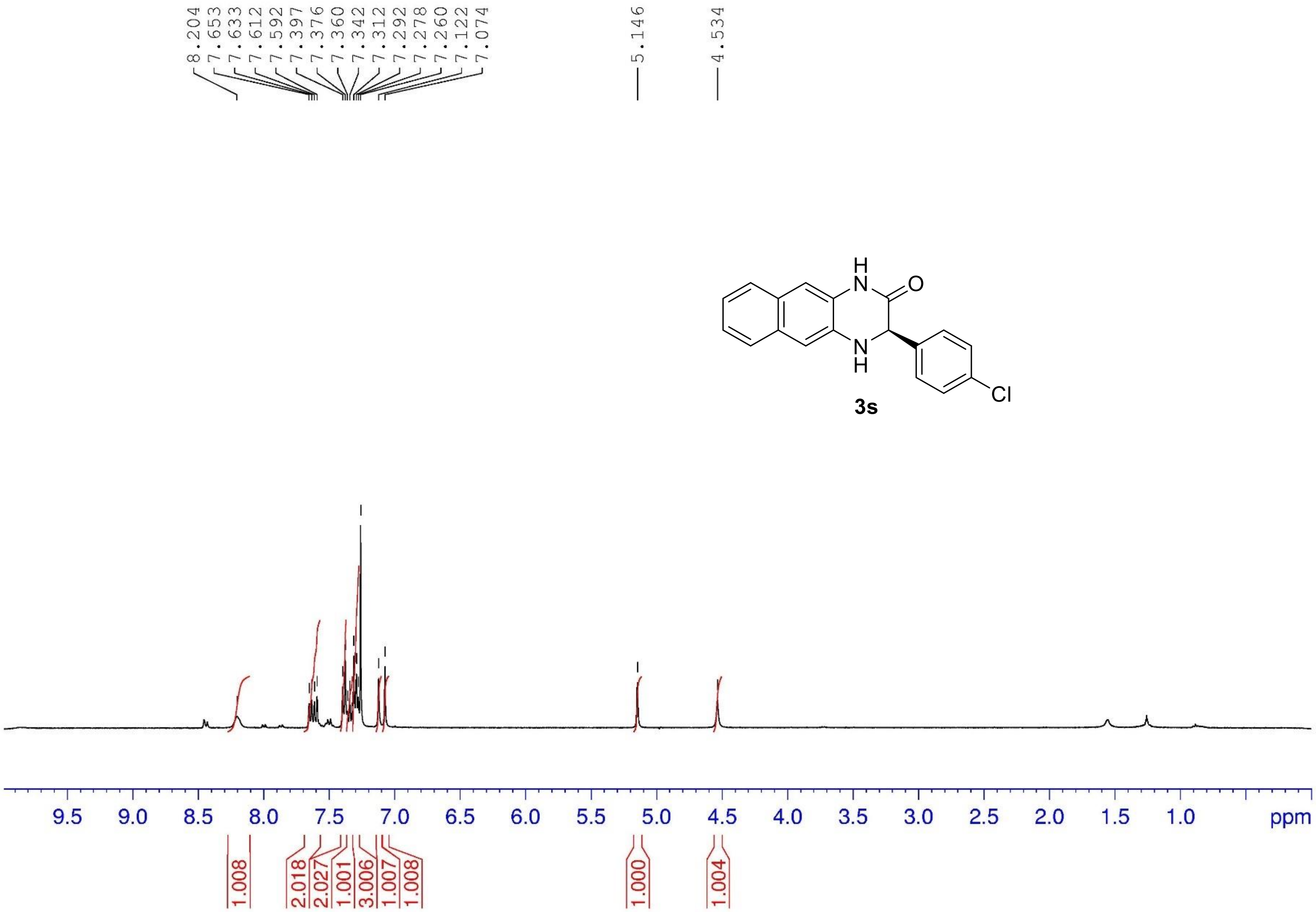

3s

^{13}C NMR in $CDCl_3$ (150 MHz)

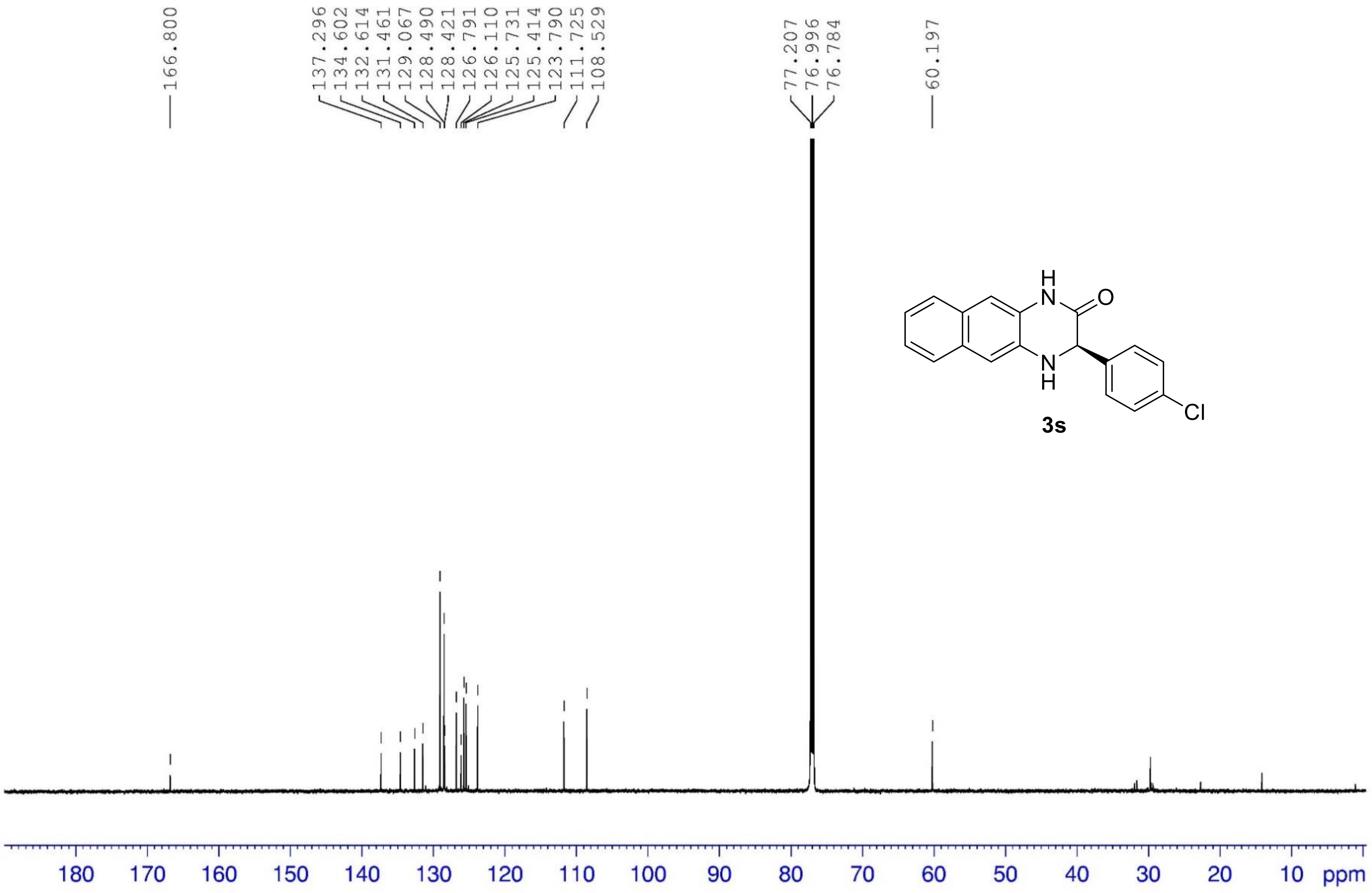

^{1}H NMR in $CDCl_3$ (300 MHz)

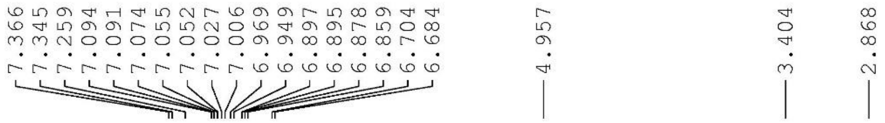

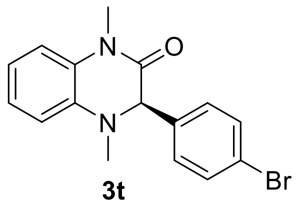

3t

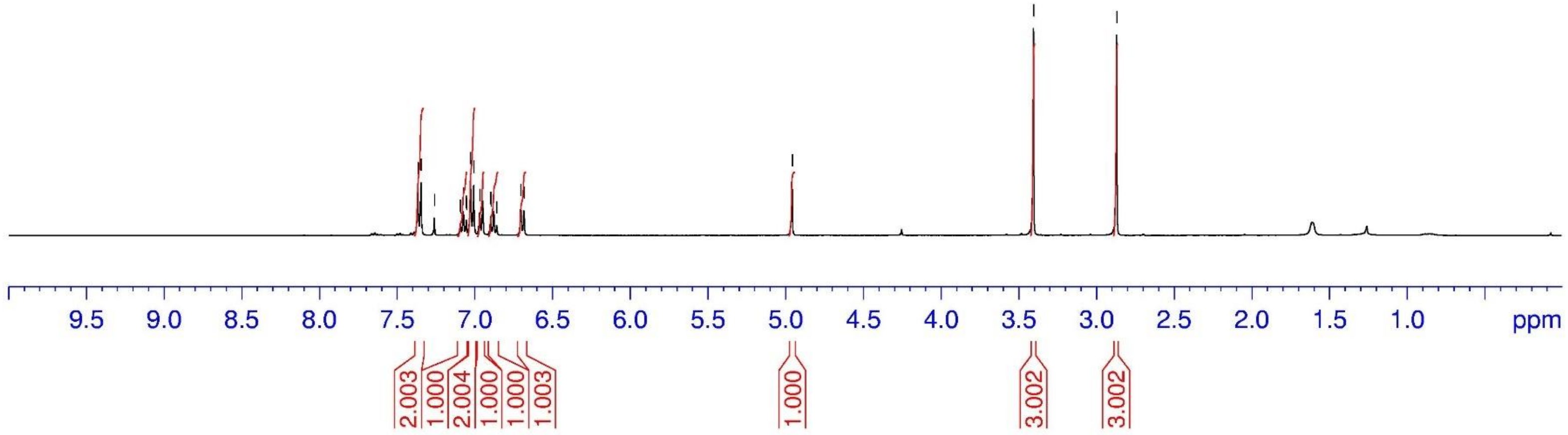

^{13}C NMR in $CDCl_3$ (100 MHz)

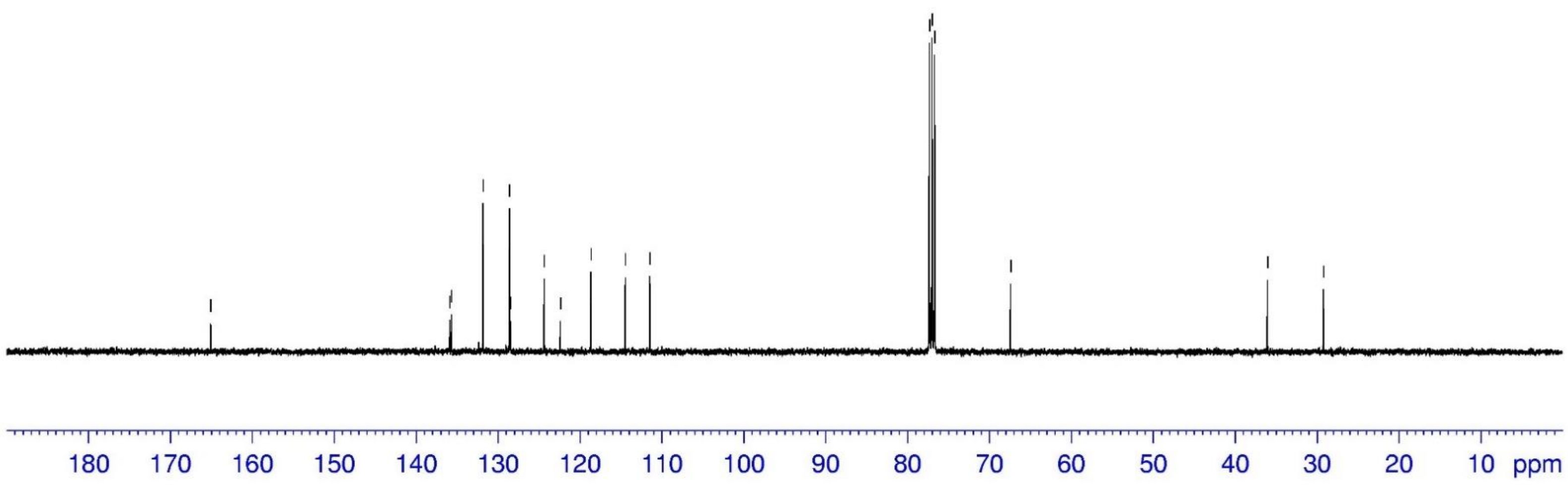

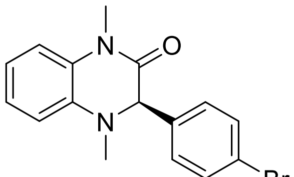

3t

^{1}H NMR in $CDCl_3$ (400 MHz)

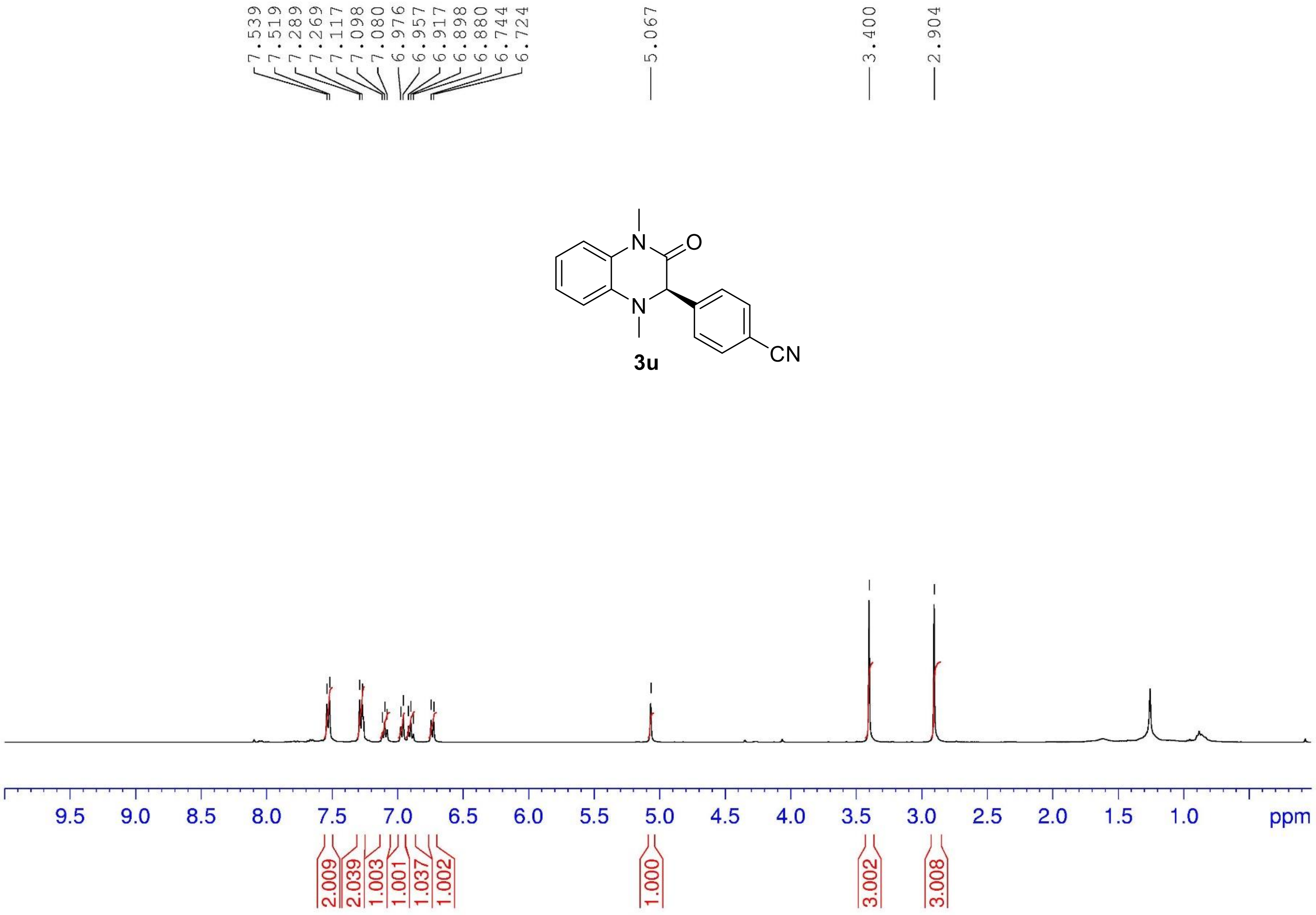

^{13}C NMR in $CDCl_3$ (100 MHz)

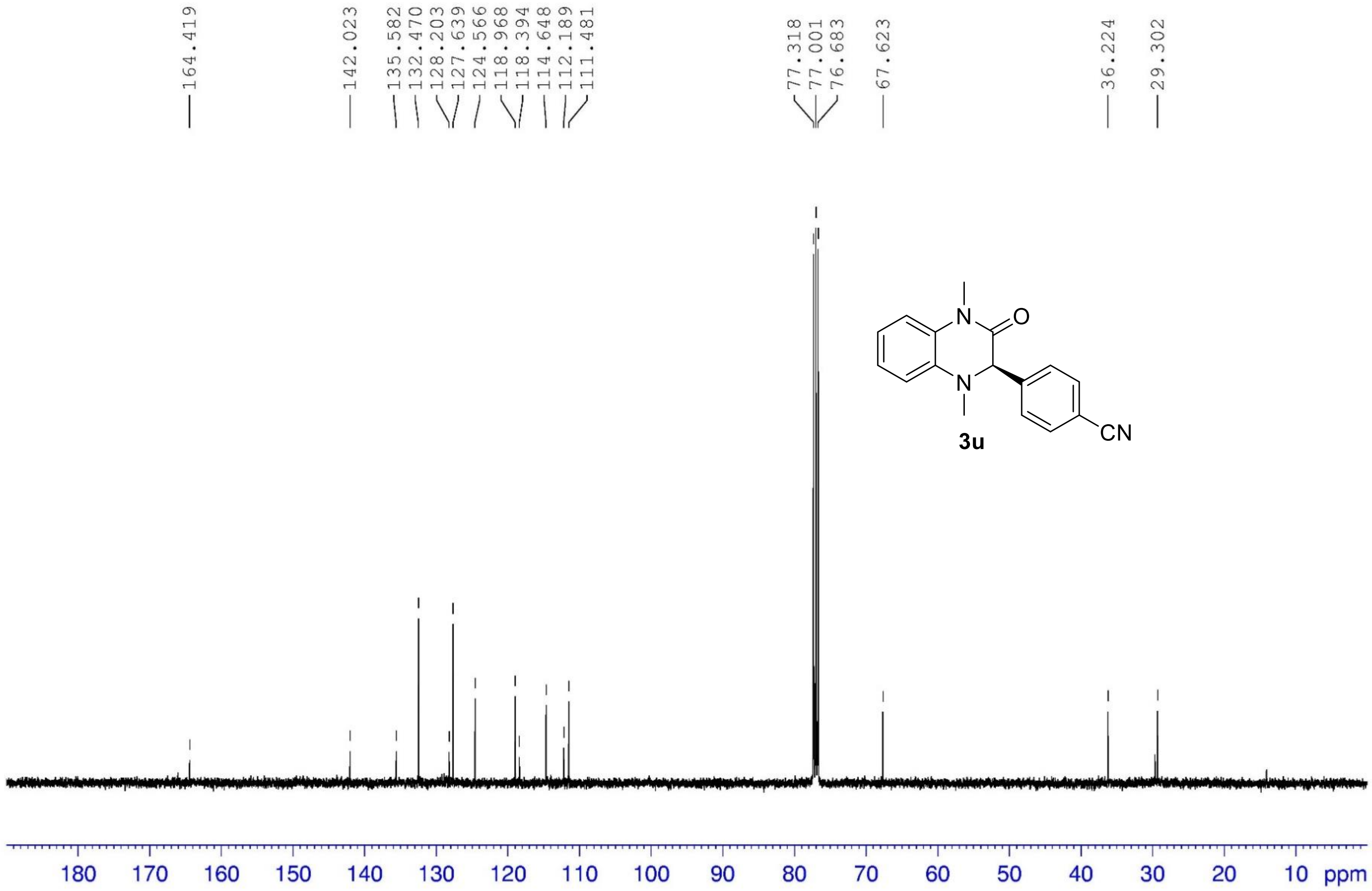

^{1}H NMR in $CDCl_3$ (600 MHz)

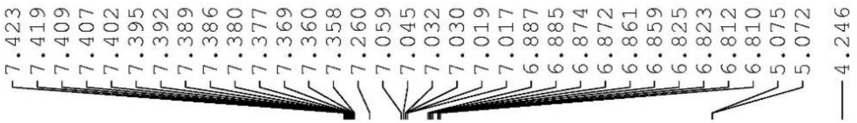

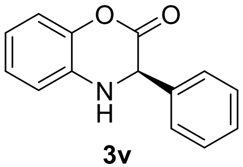

3v

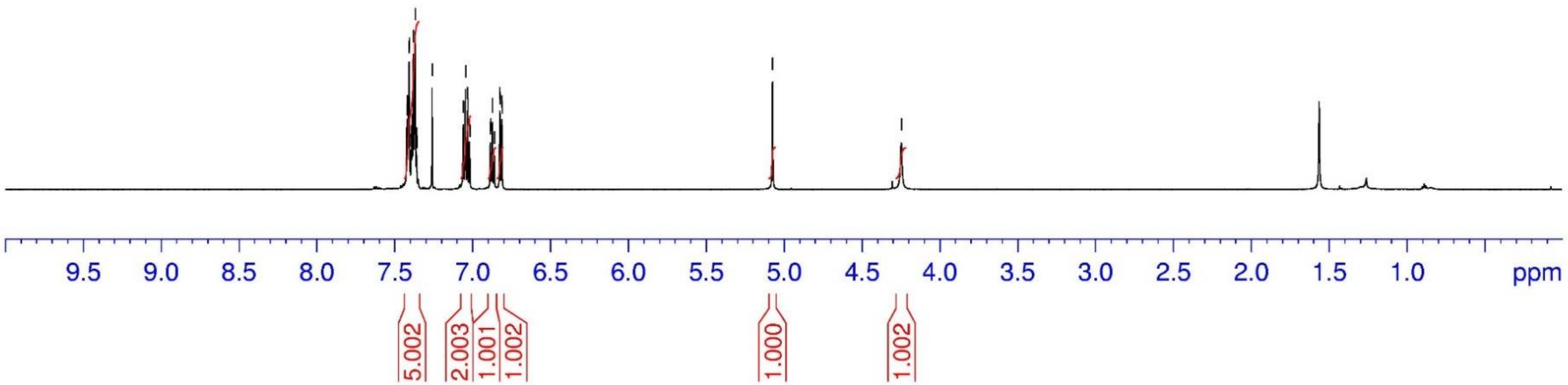

^{13}C NMR in $CDCl_3$ (150 MHz)

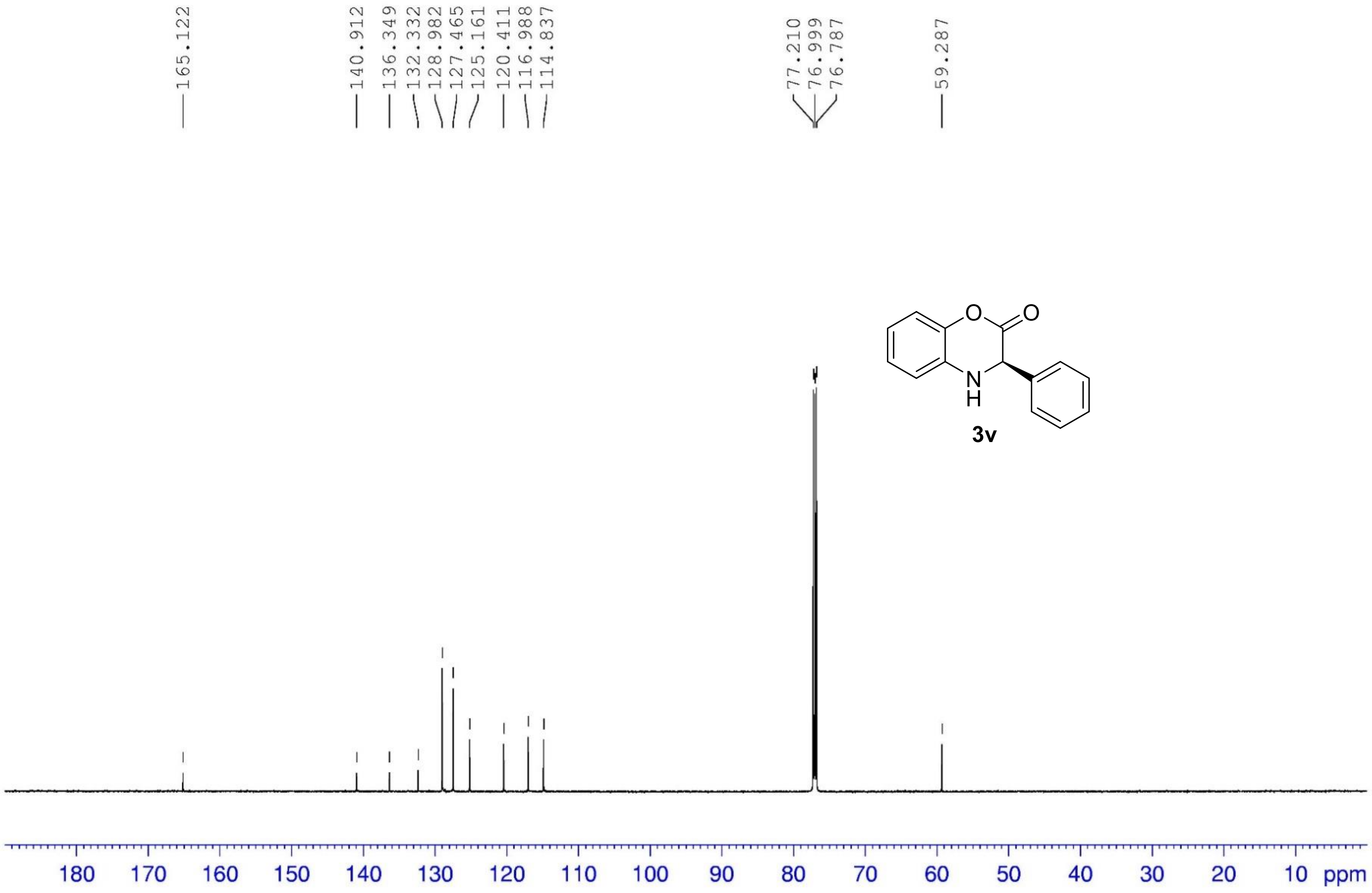

^{1}H NMR in $CDCl_3$ (400 MHz)

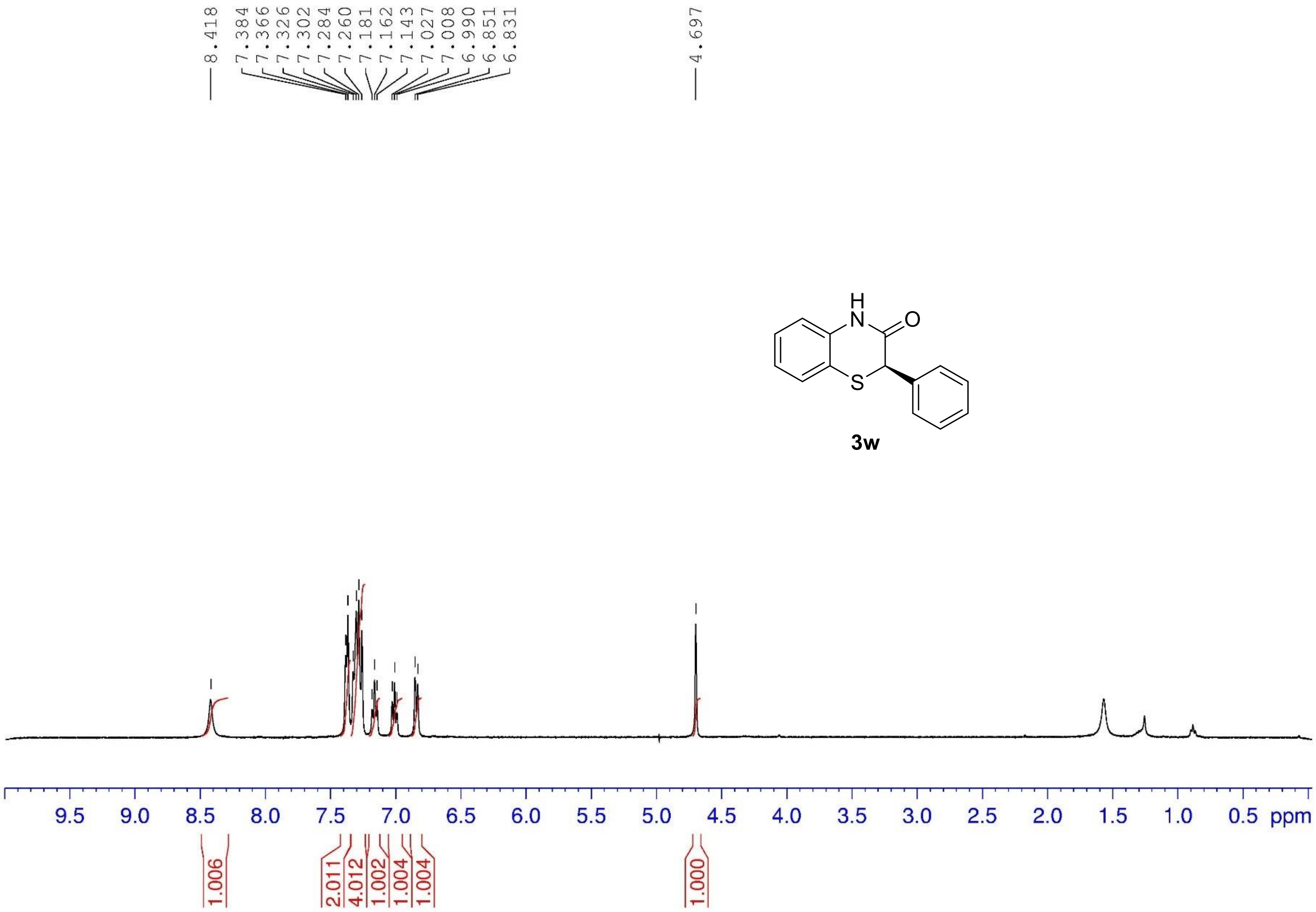

3w

^{13}C NMR in $CDCl_3$ (100 MHz)

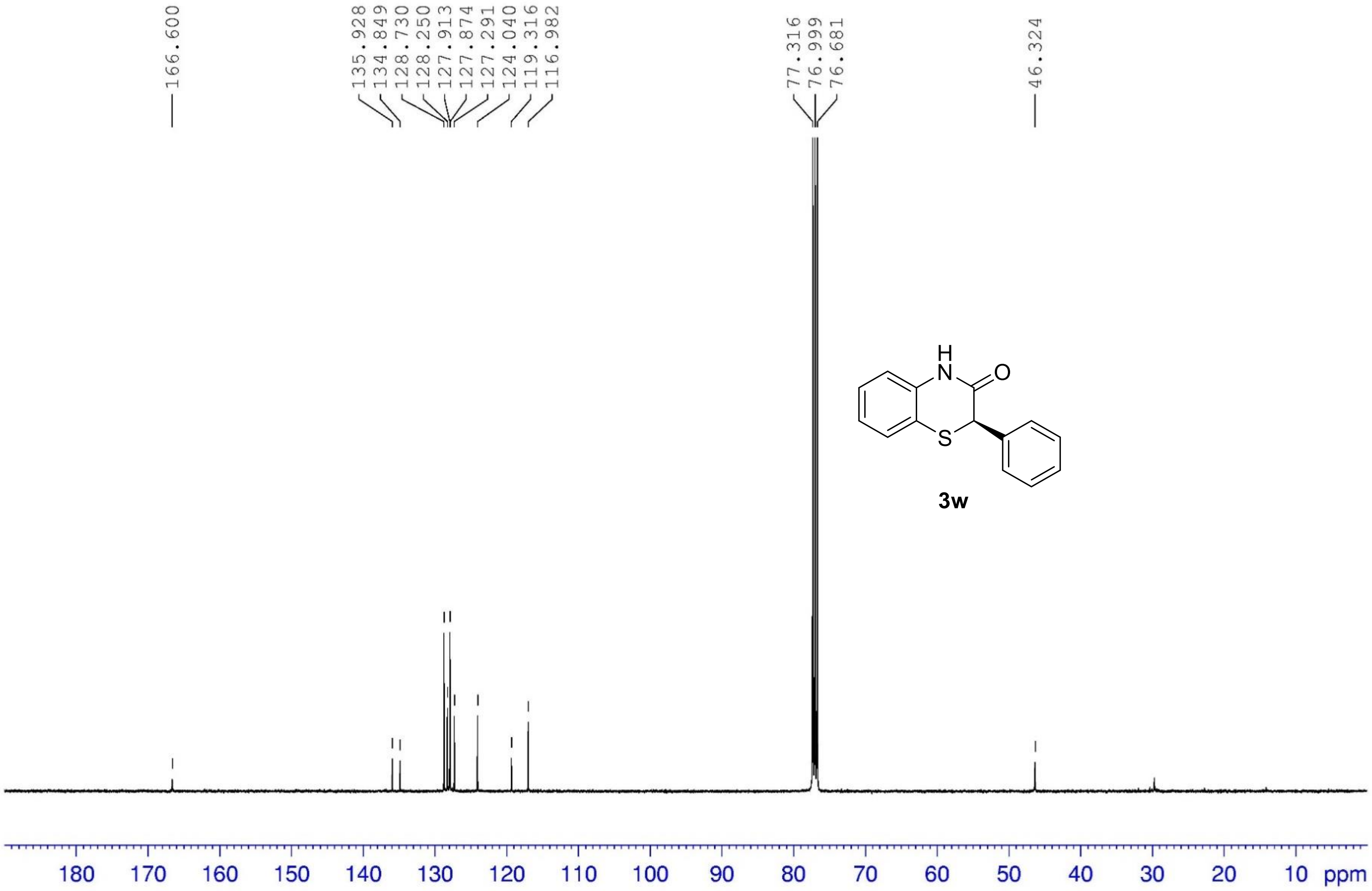

^{1}H NMR in $CDCl_3$ (400 MHz)

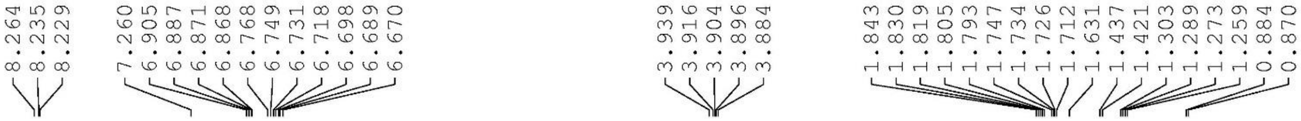

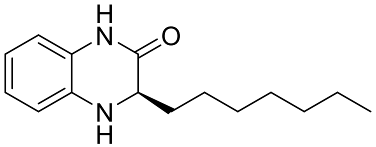

3x

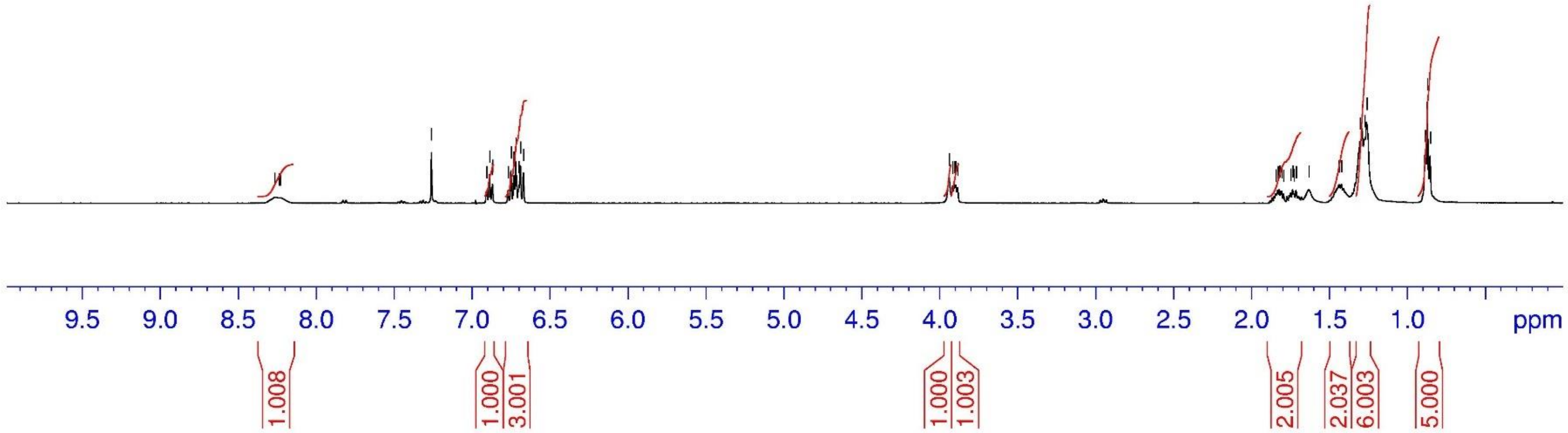

^{13}C NMR in $CDCl_3$ (100 MHz)

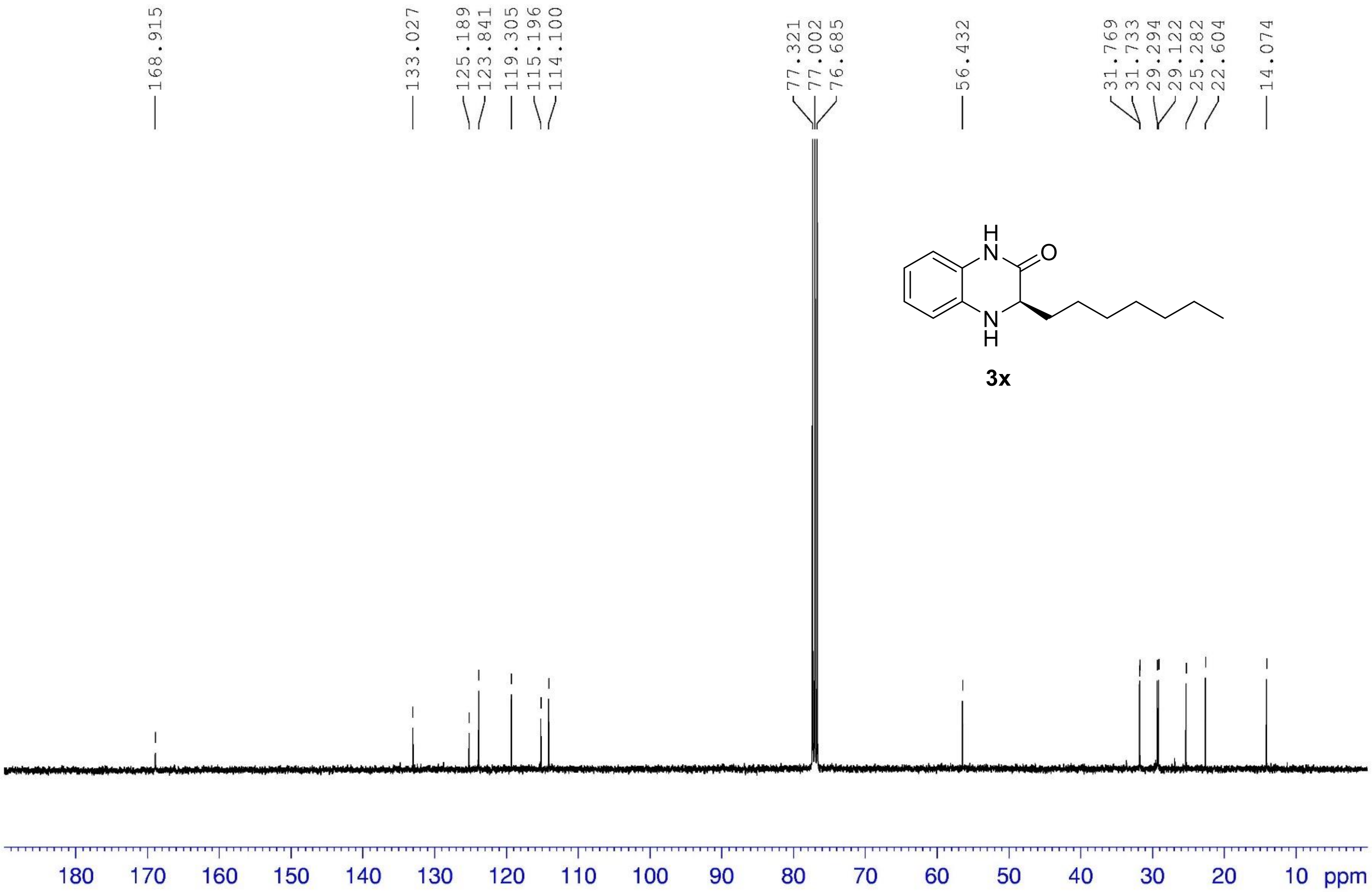

3x

^{1}H NMR in $CDCl_3$ (400 MHz)

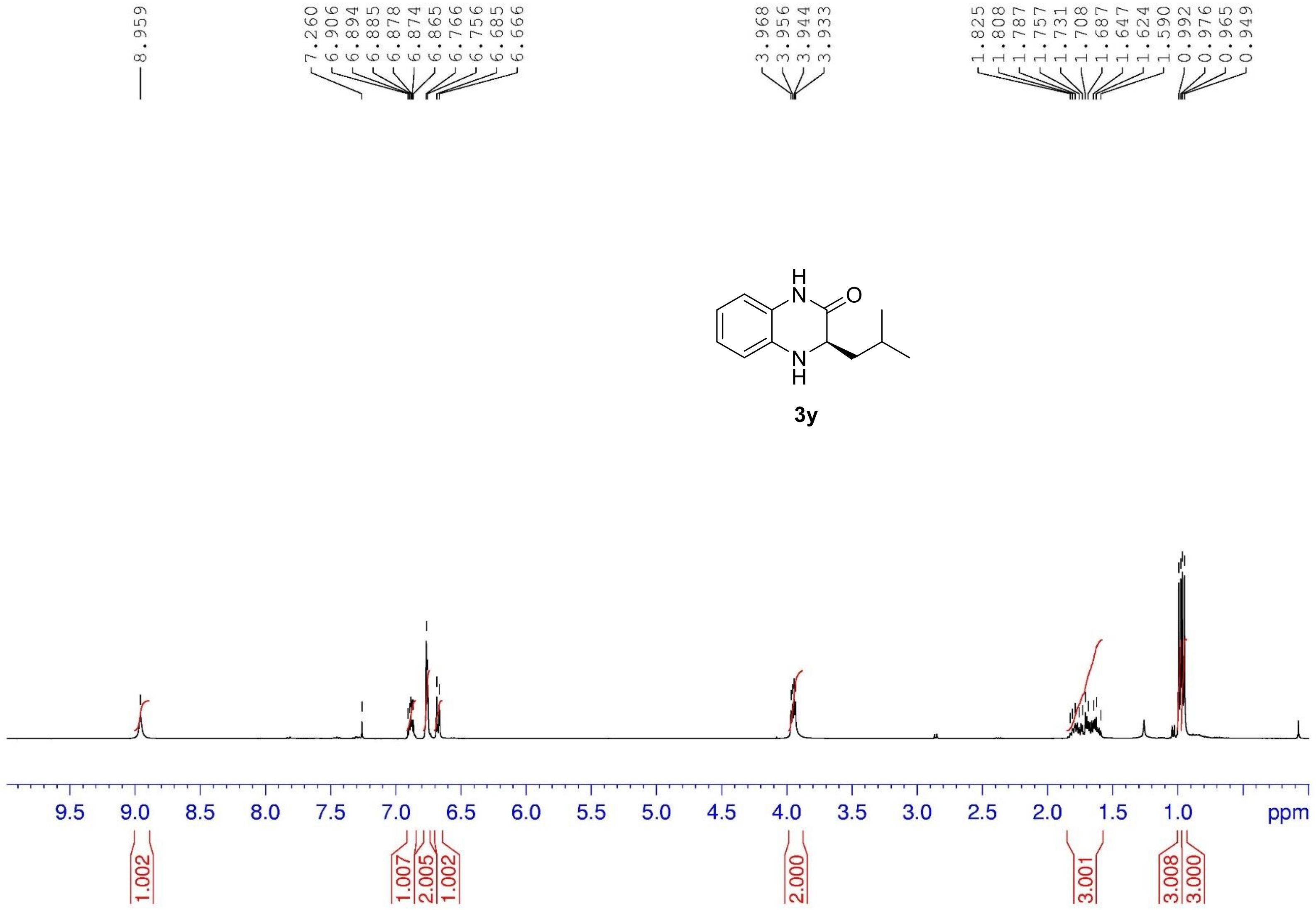

^{13}C NMR in $CDCl_3$ (100 MHz)

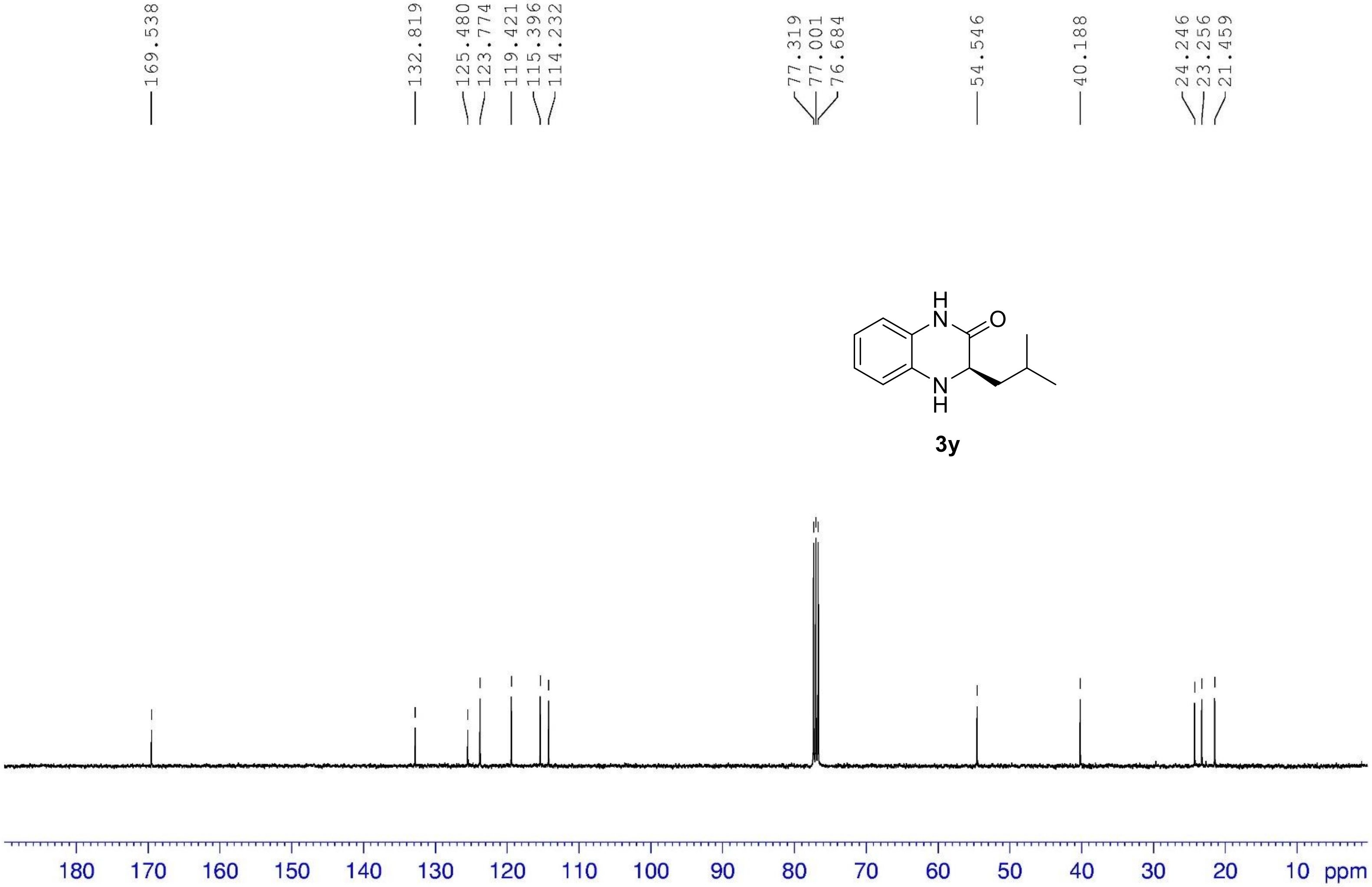

^{1}H NMR in $CDCl_3$ (400 MHz)

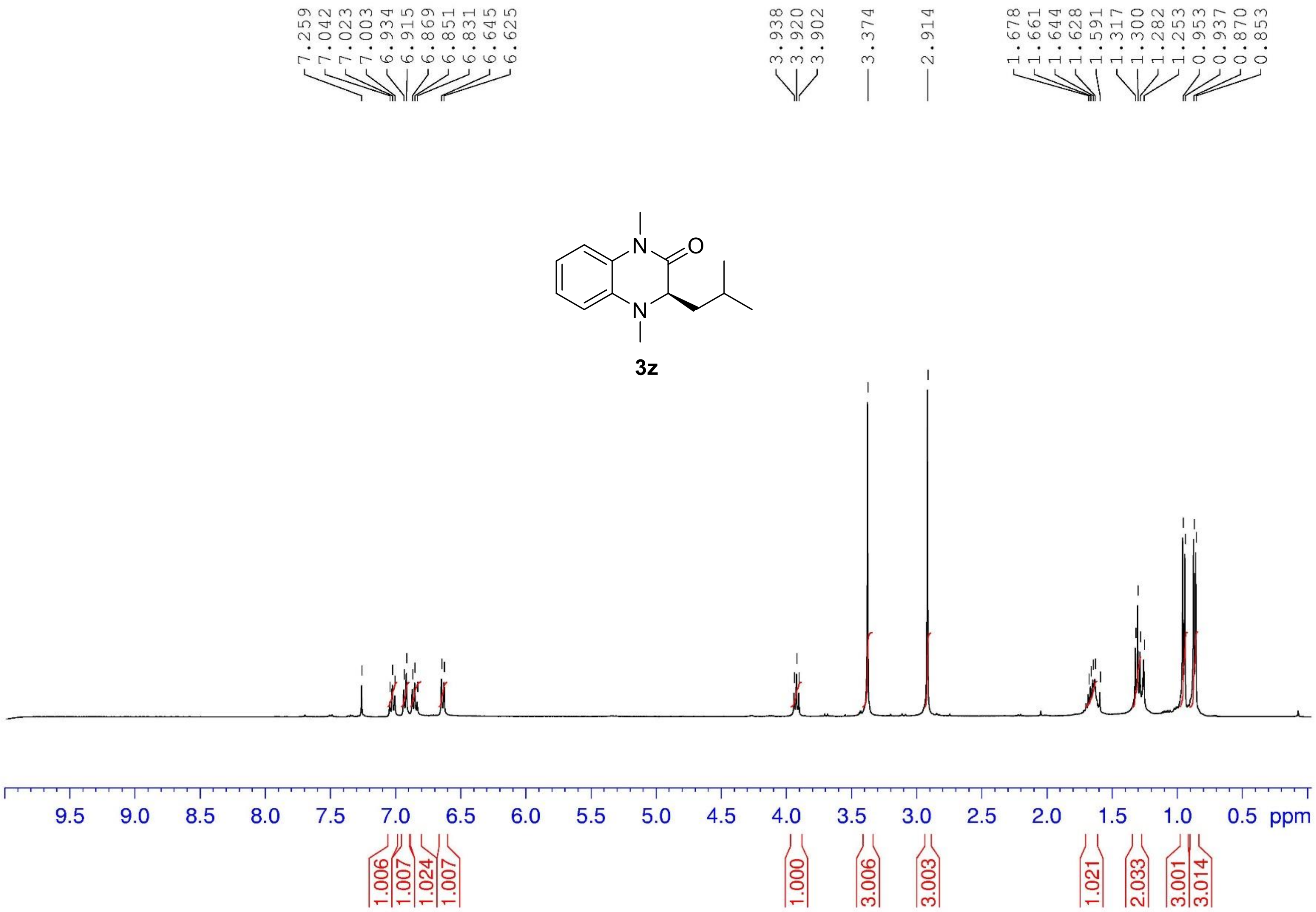

^{13}C NMR in $CDCl_3$ (100 MHz)

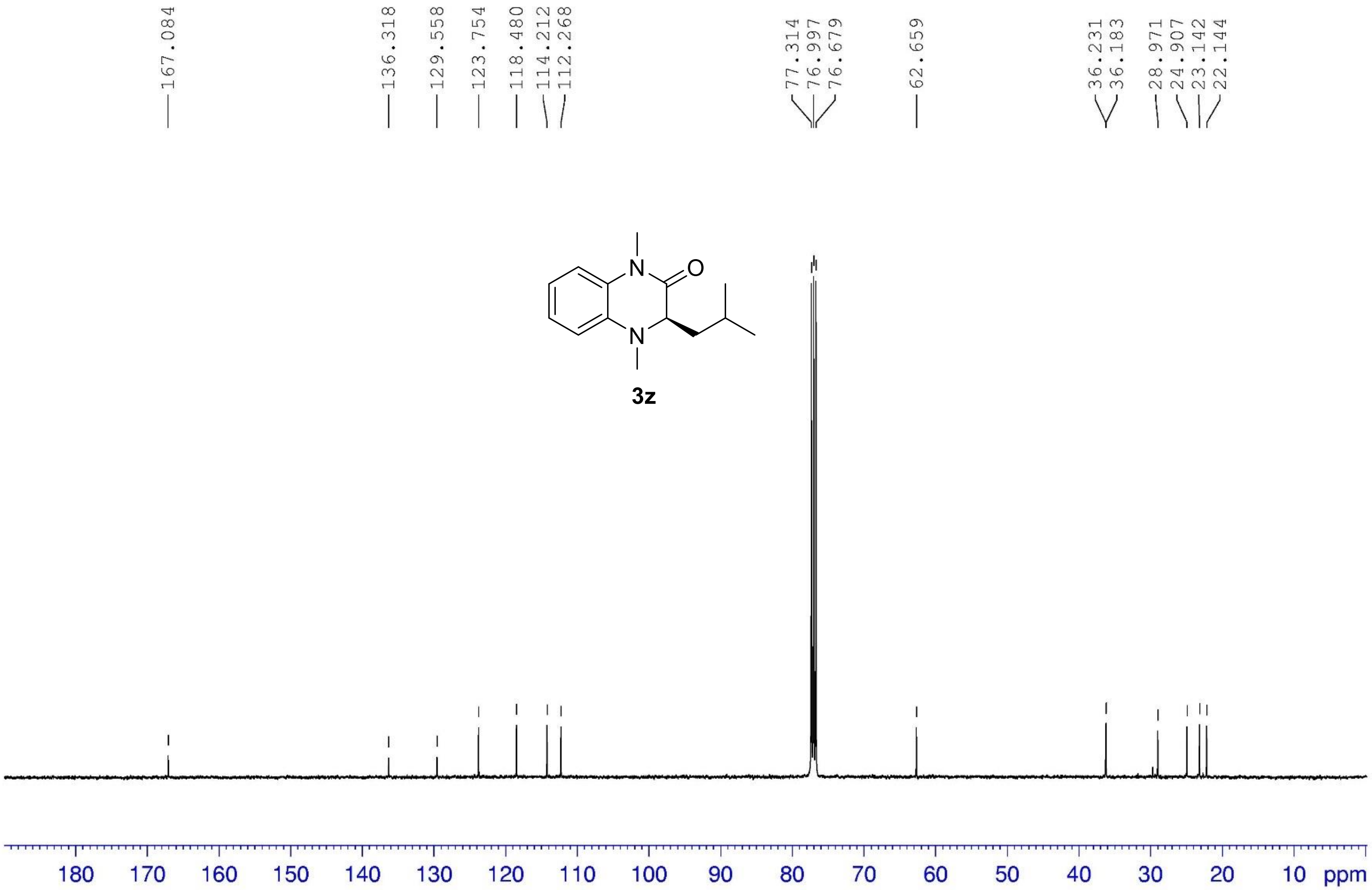

^{1}H NMR in $CDCl_3$ (300 MHz)

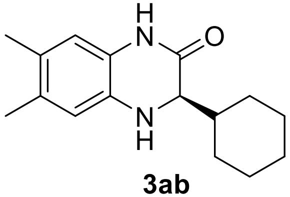

3ab

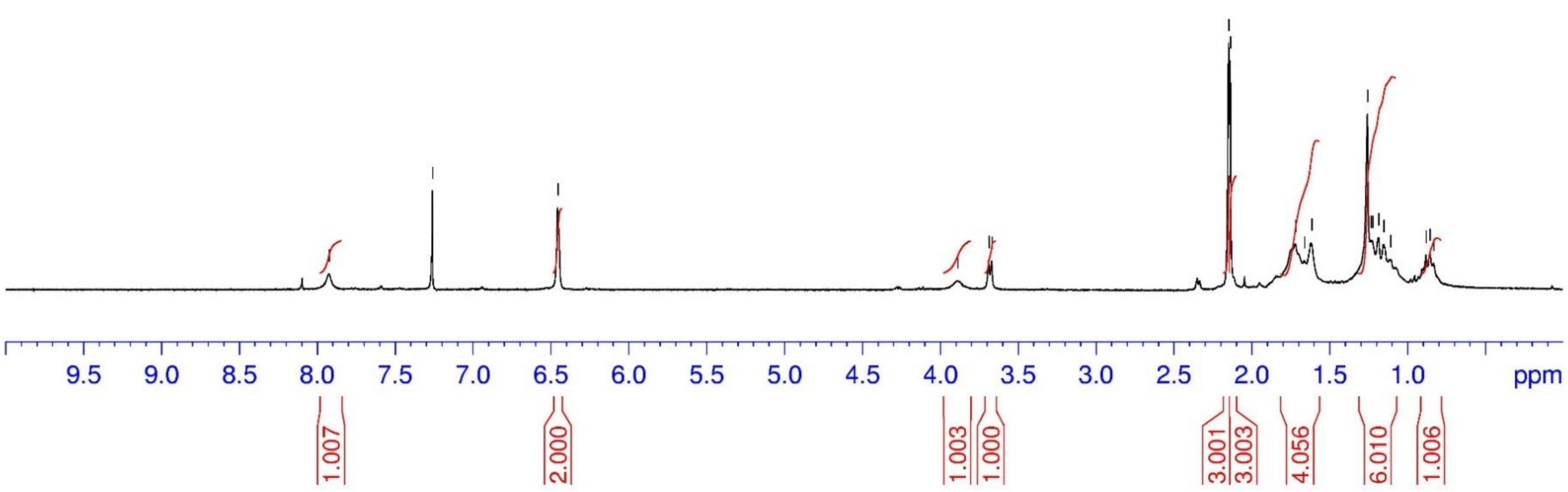

^{13}C NMR in $CDCl_3$ (75 MHz)

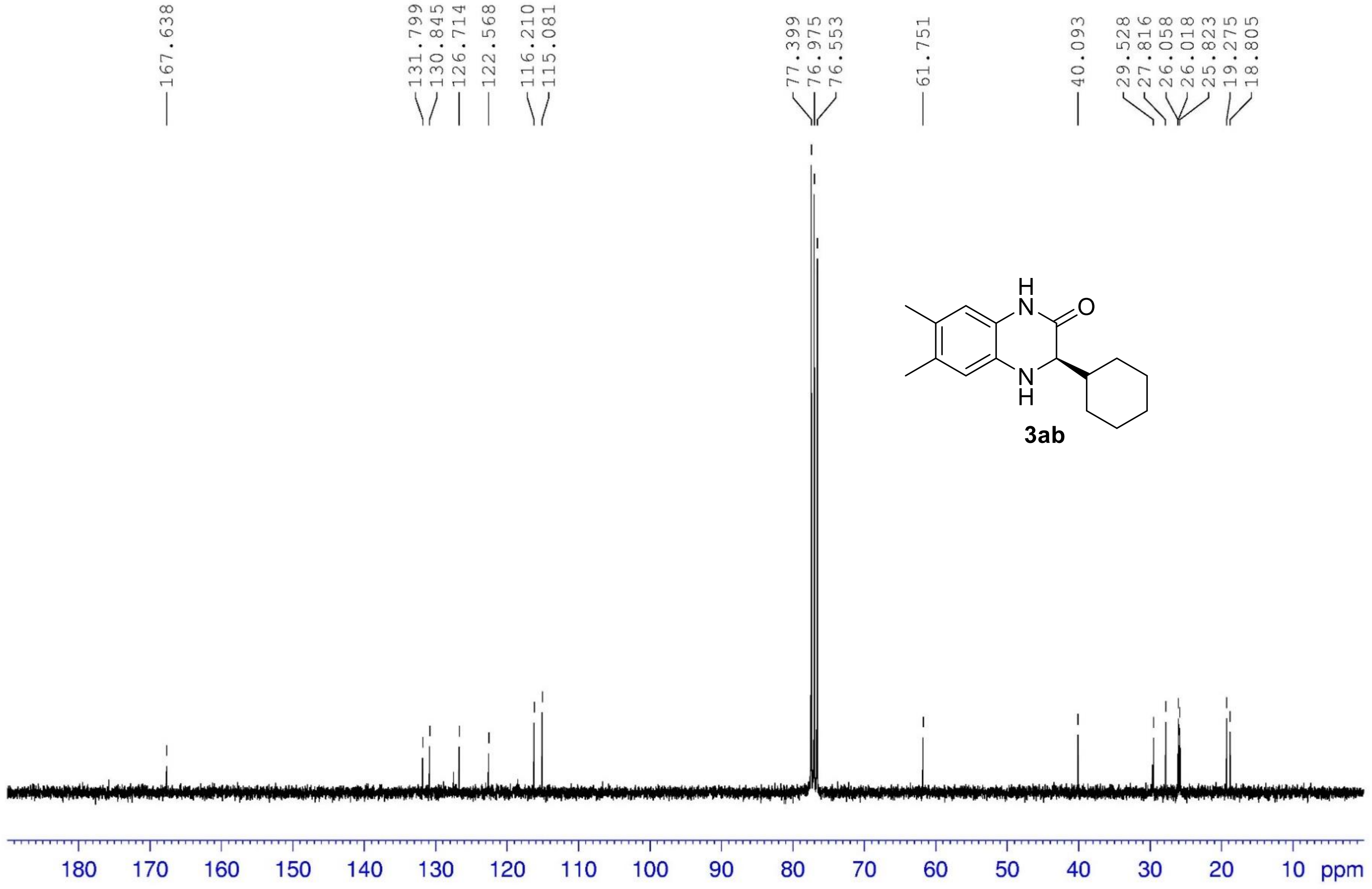

^{1}H NMR in $CDCl_3$ (250 MHz)

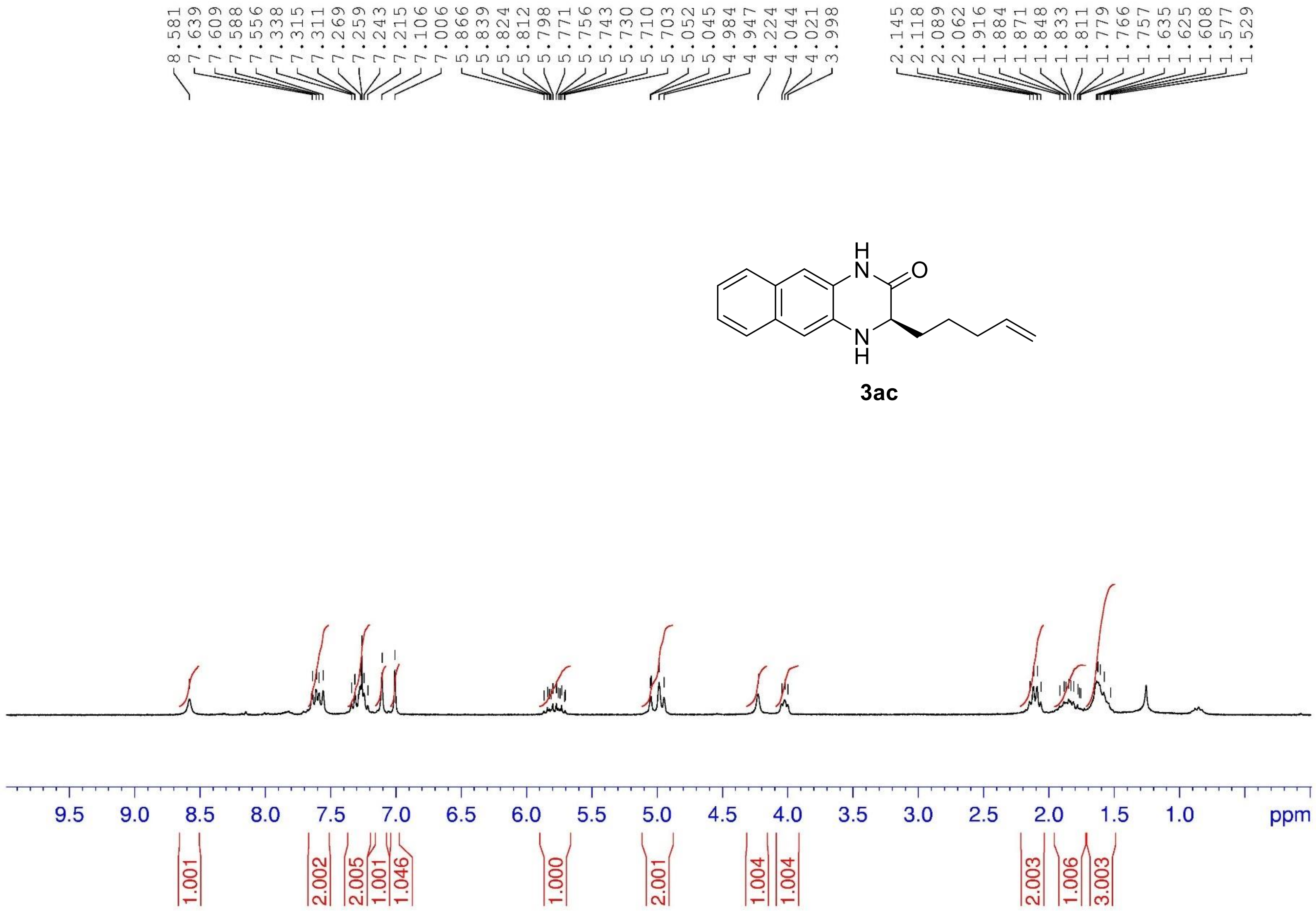

^{13}C NMR in $CDCl_3$ (62.5 MHz)

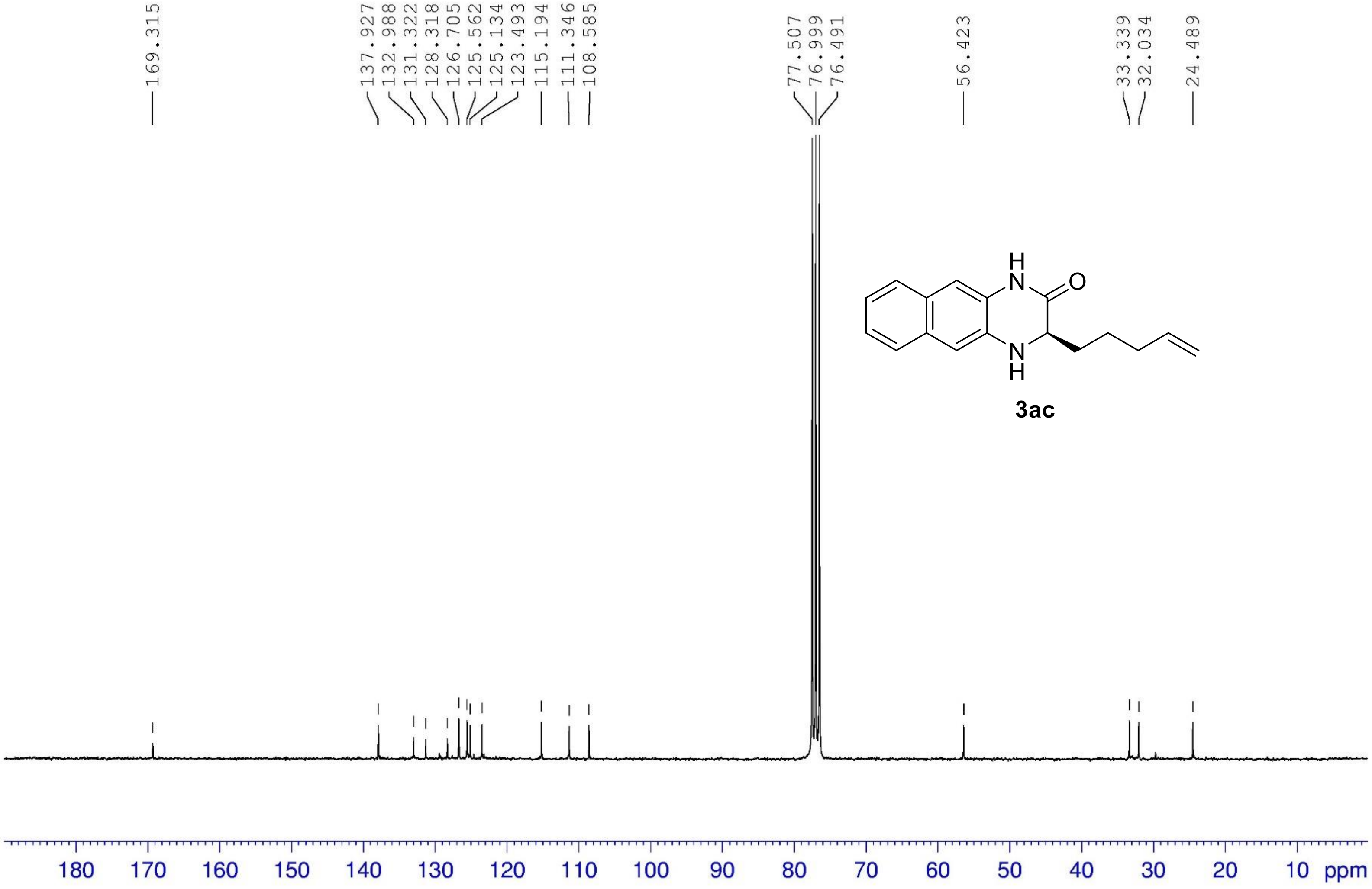

3ac

^{1}H NMR in $CDCl_3$ (300 MHz)

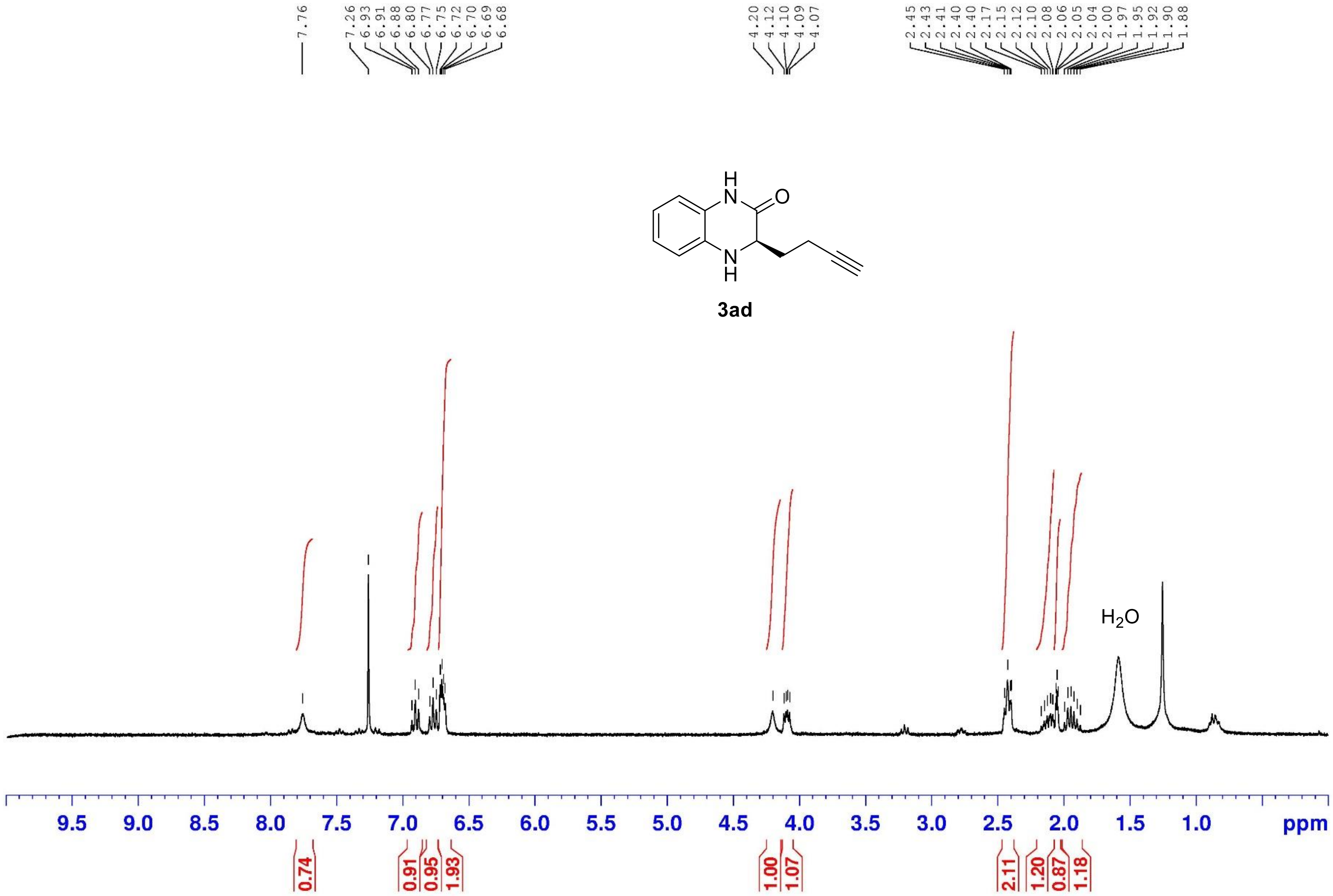

^{13}C NMR in $CDCl_3$ (100 MHz)

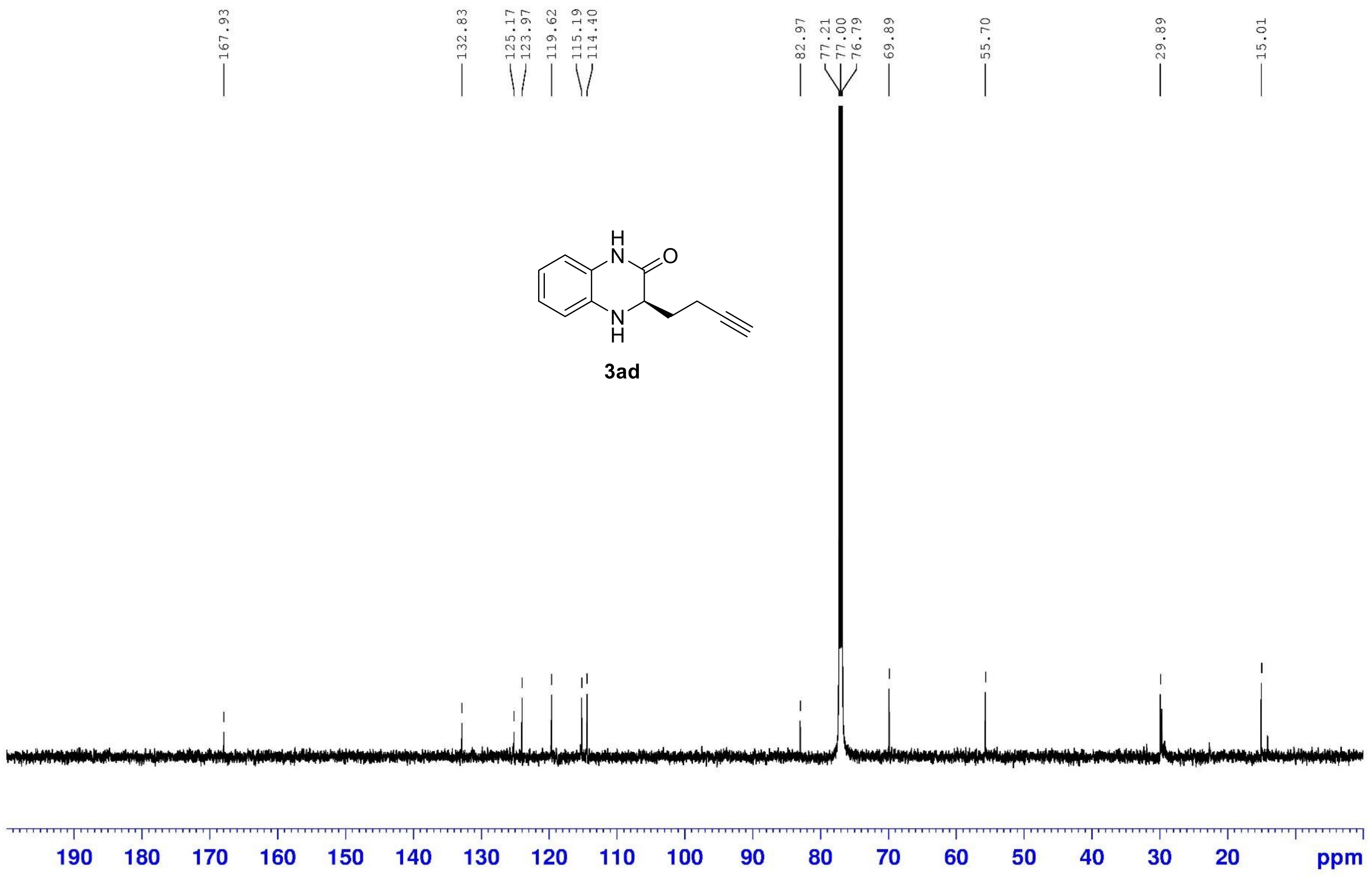

^{1}H NMR in $CDCl_3$ (400 MHz)

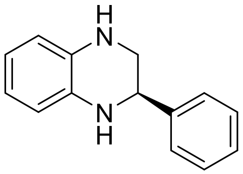

4a

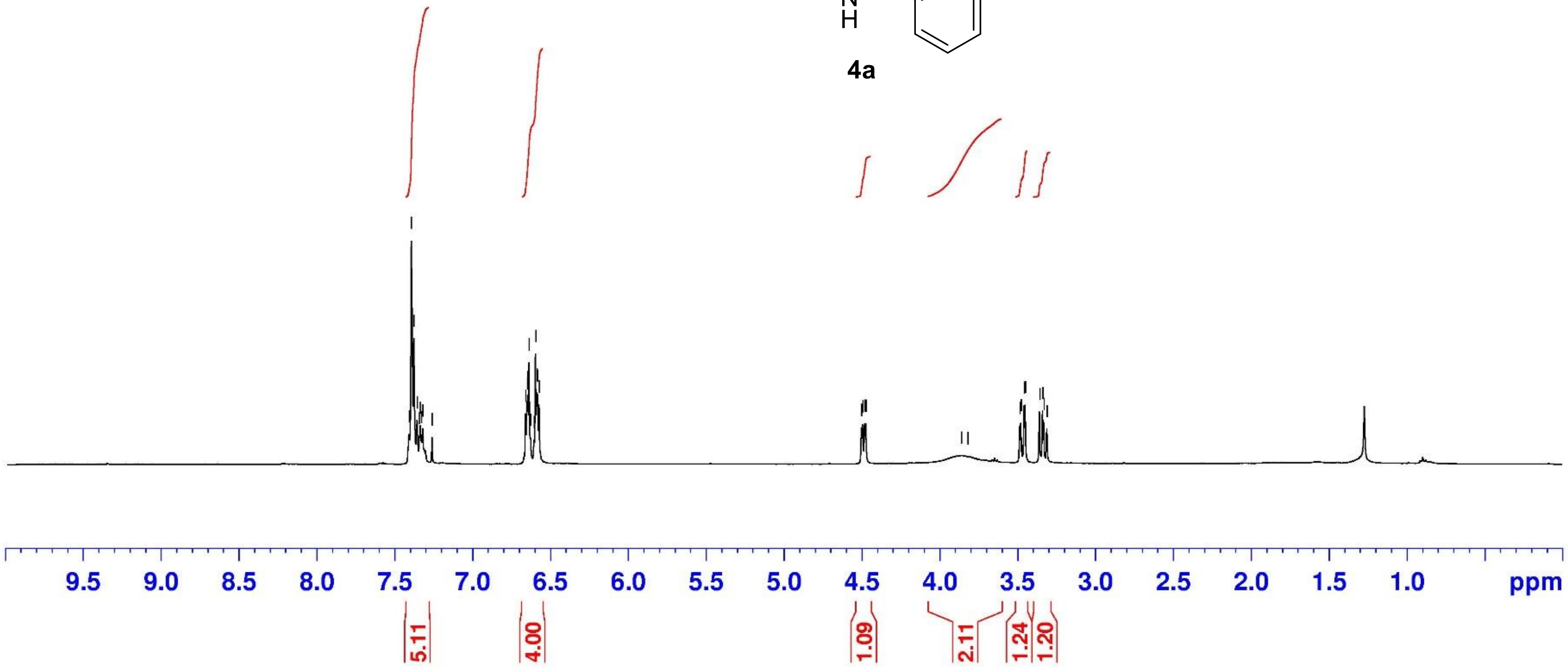

^{13}C NMR in $CDCl_3$ (75 MHz)

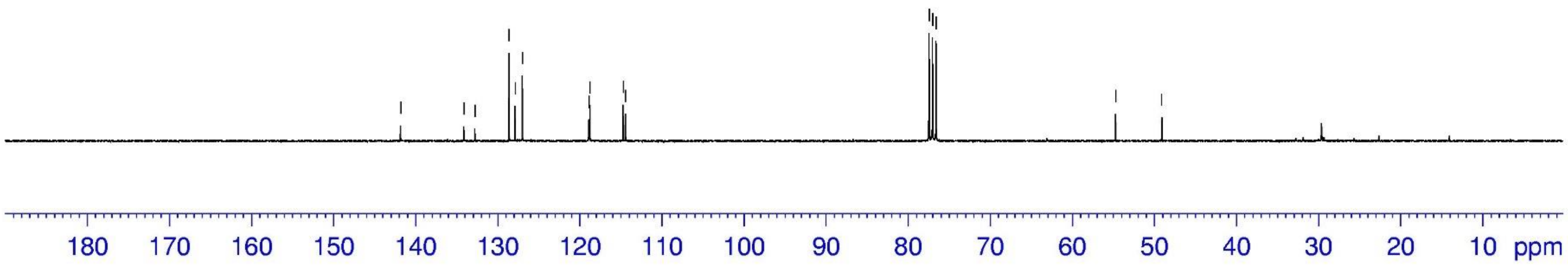

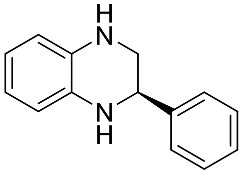

4a

^{1}H NMR in $CDCl_3$ (400 MHz)

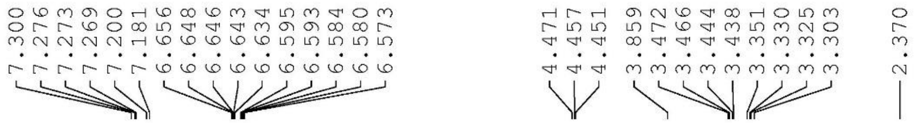

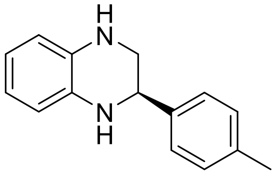

4b

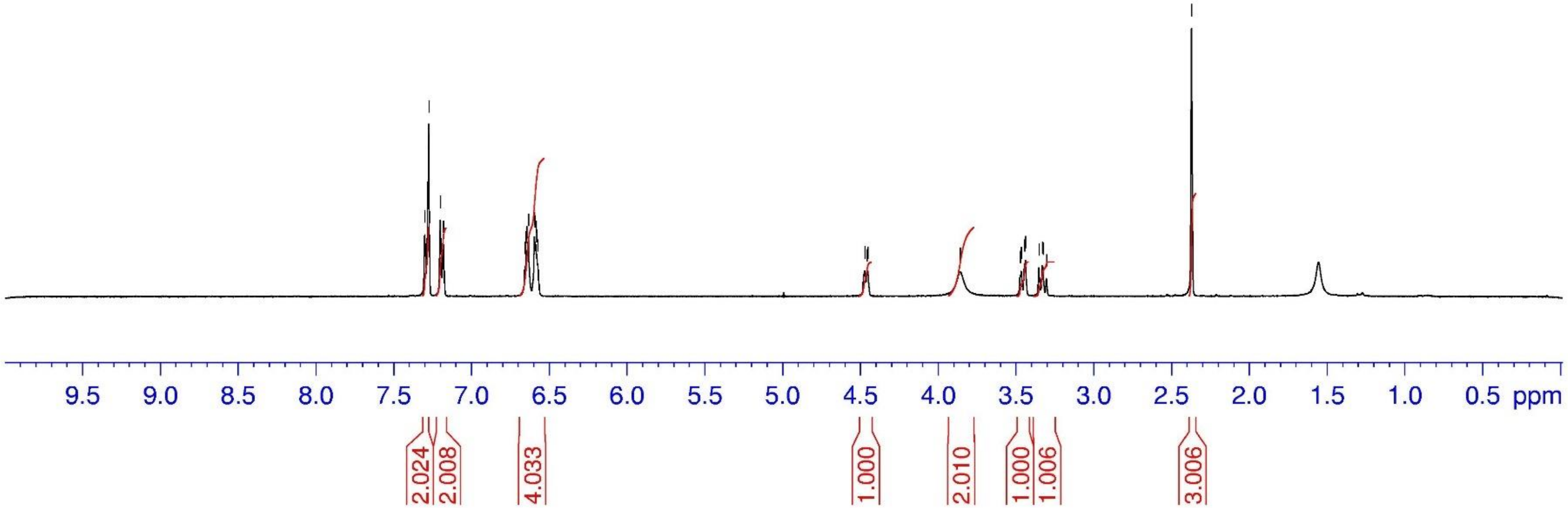

^{13}C NMR in $CDCl_3$ (100 MHz)

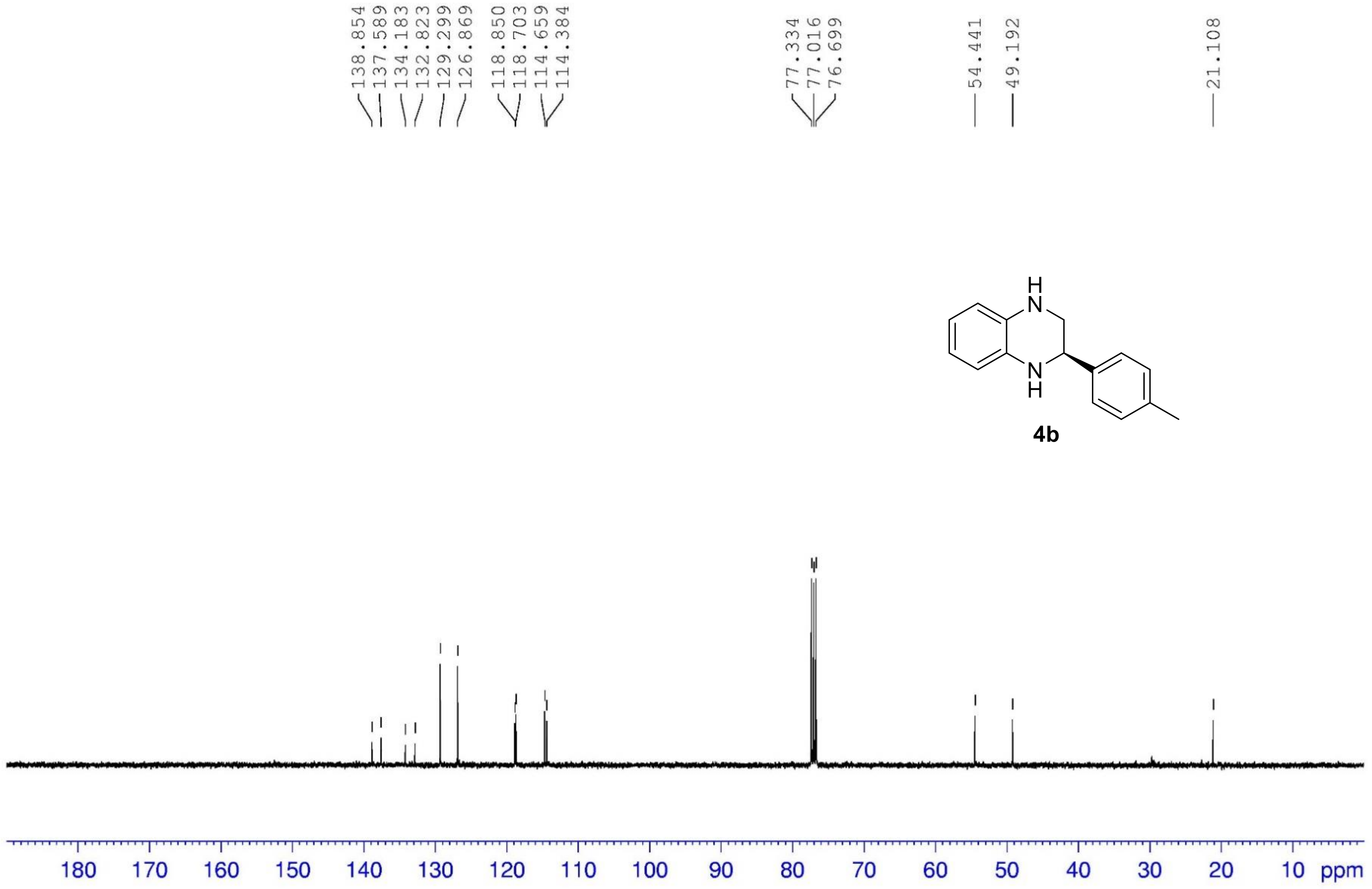

^{1}H NMR in CDCl$_3$ (400 MHz)

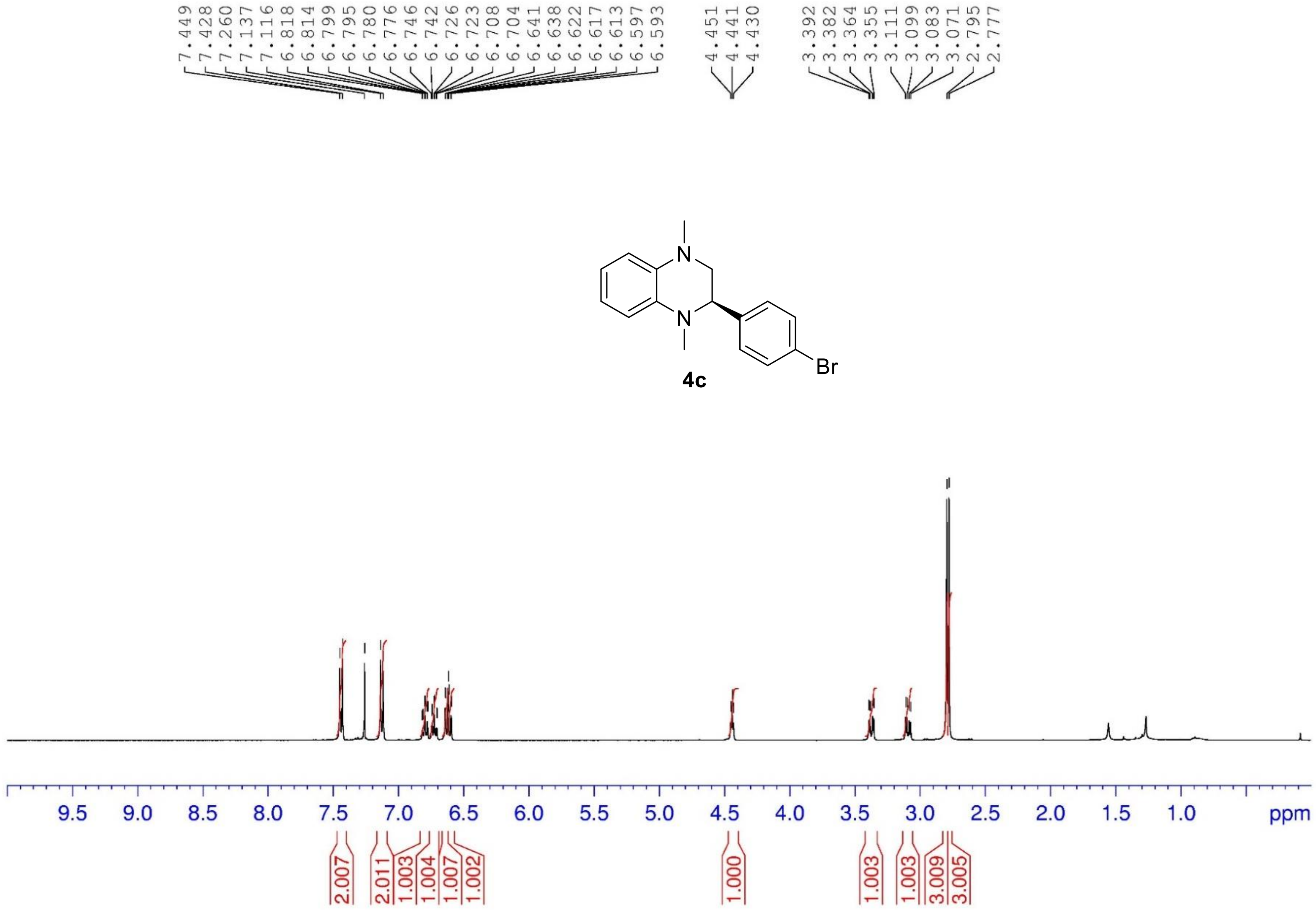

^{13}C NMR in $CDCl_3$ (100 MHz)

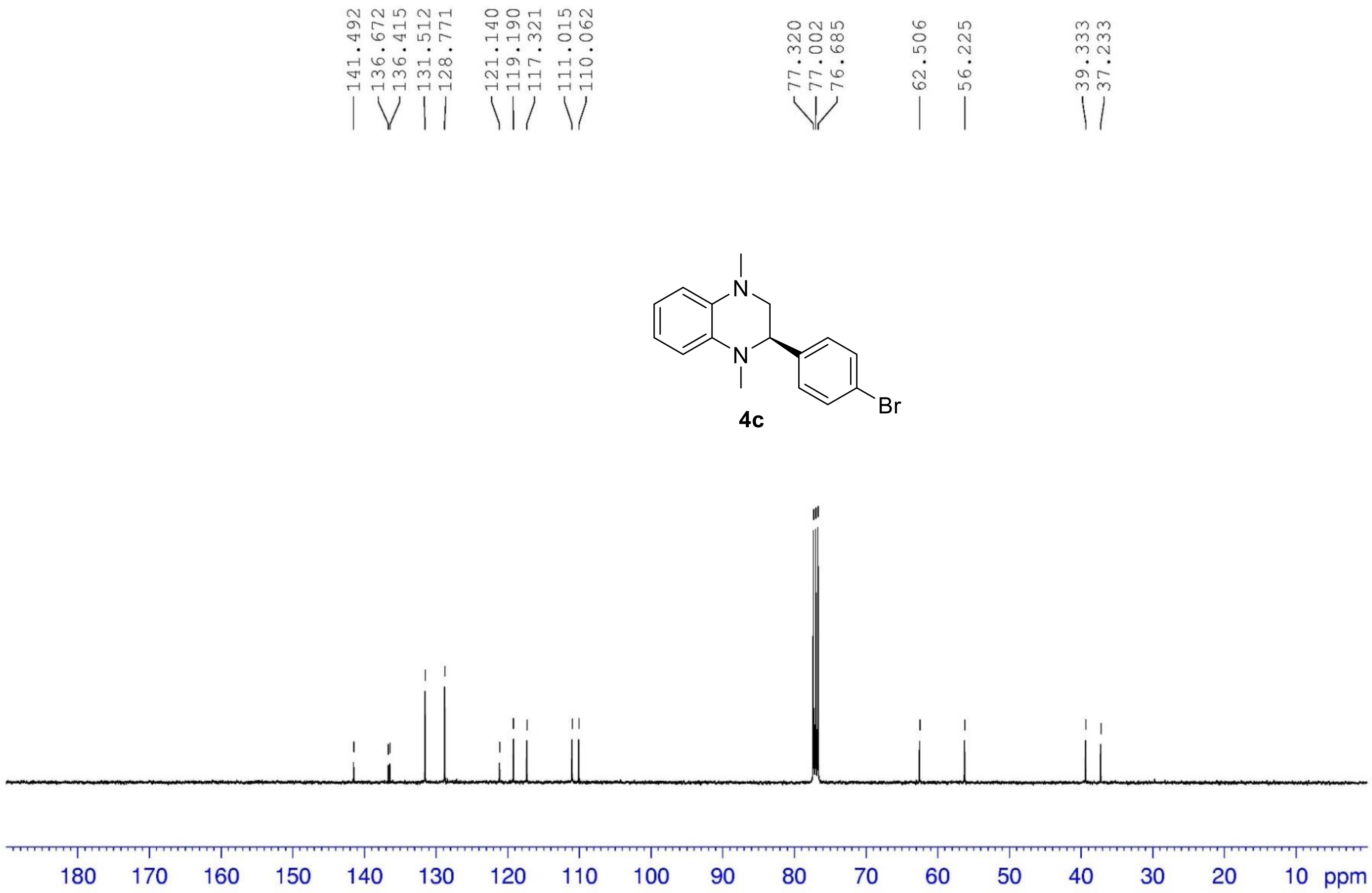

HPLC chromatograms

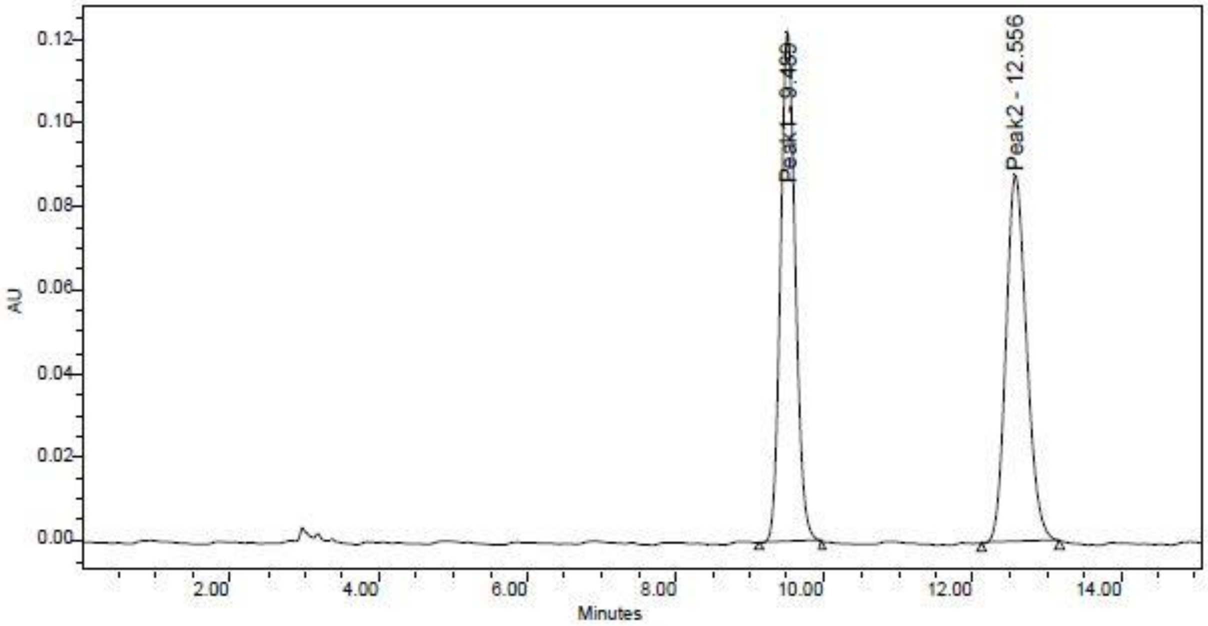

	Peak Name	RT (min)	Area (□V*sec)	% Area	Height (□V)	% Height
1	Peak1	9.489	1702046	49.98	122216	58.16
2	Peak2	12.556	1703269	50.02	87929	41.84

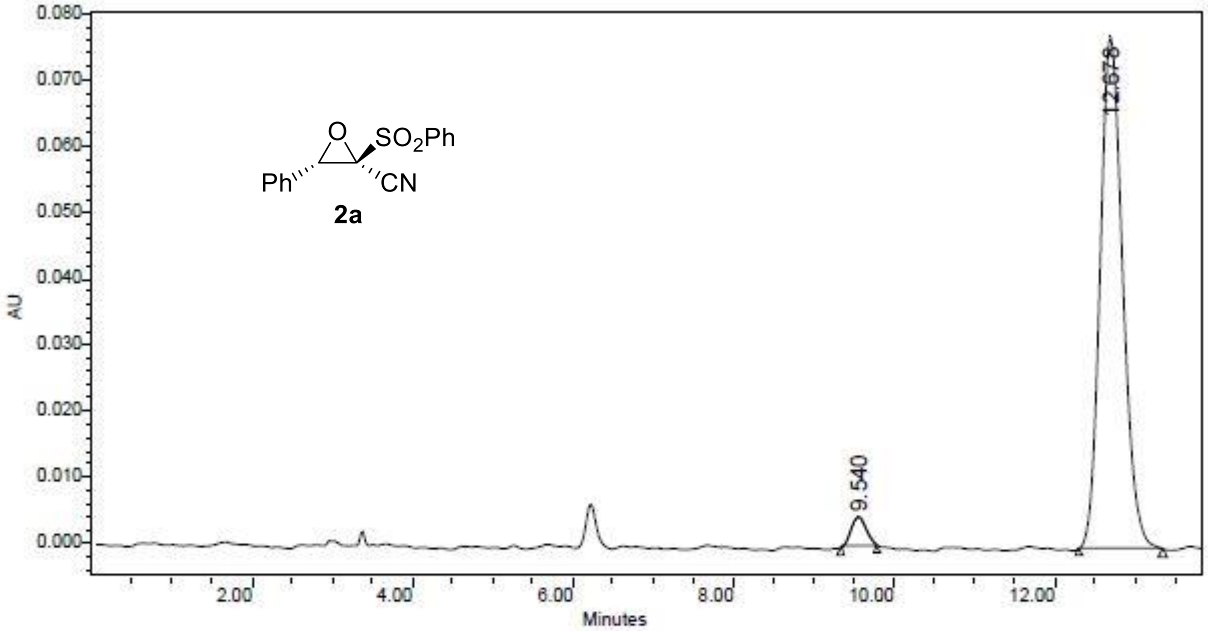

	RT (min)	Area (□V*sec)	% Area	Height (□V)	% Height
1	9.540	59219	3.78	4452	5.46
2	12.678	1509167	96.22	77118	94.54

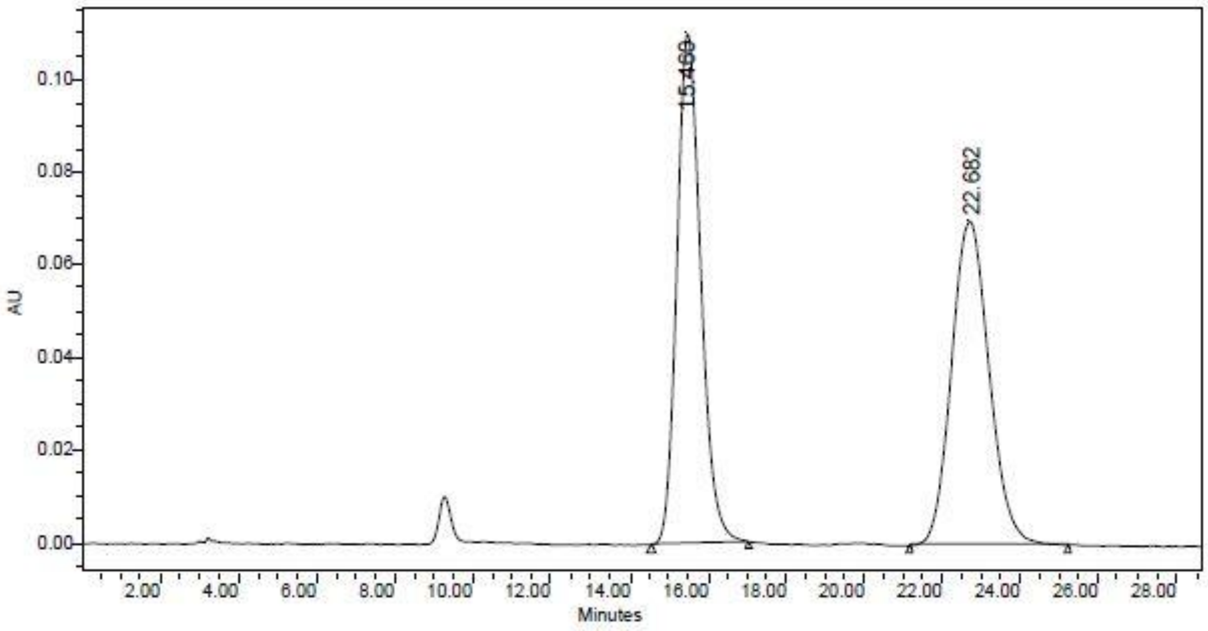

	RT (min)	Area (□V*sec)	% Area	Height (□V)	% Height
1	15.460	4685381	49.99	110074	61.14
2	22.682	4686475	50.01	69951	38.86

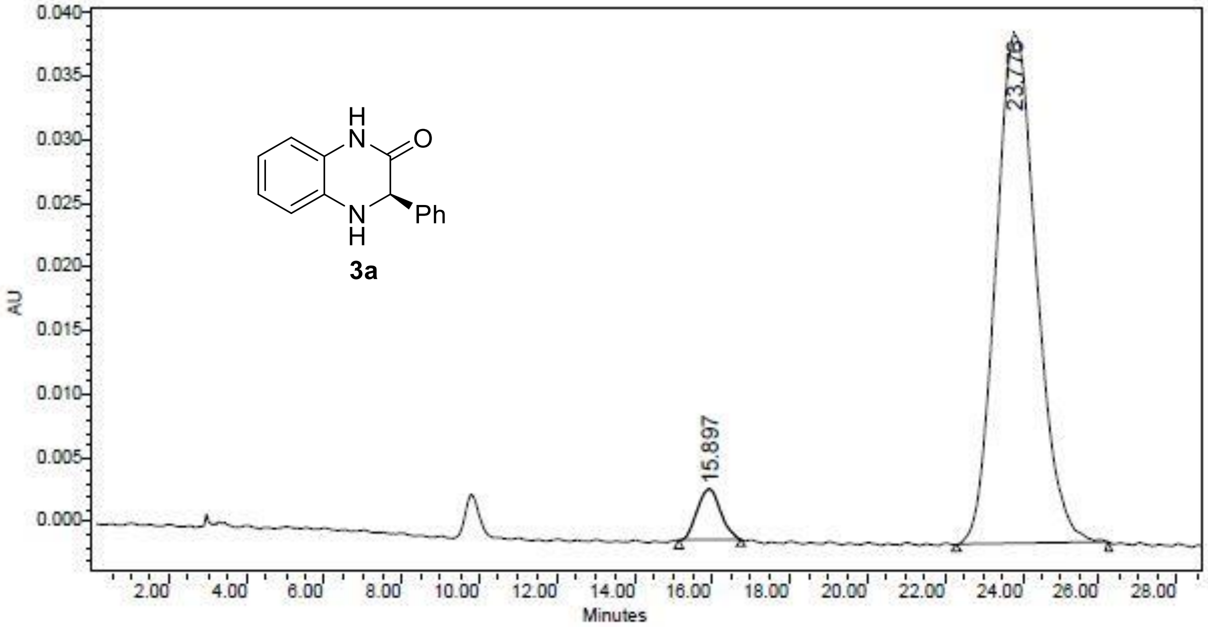

	RT (min)	Area (□V*sec)	% Area	Height (□V)	% Height
1	15.897	171109	5.51	4025	9.13
2	23.776	2936750	94.49	40050	90.87

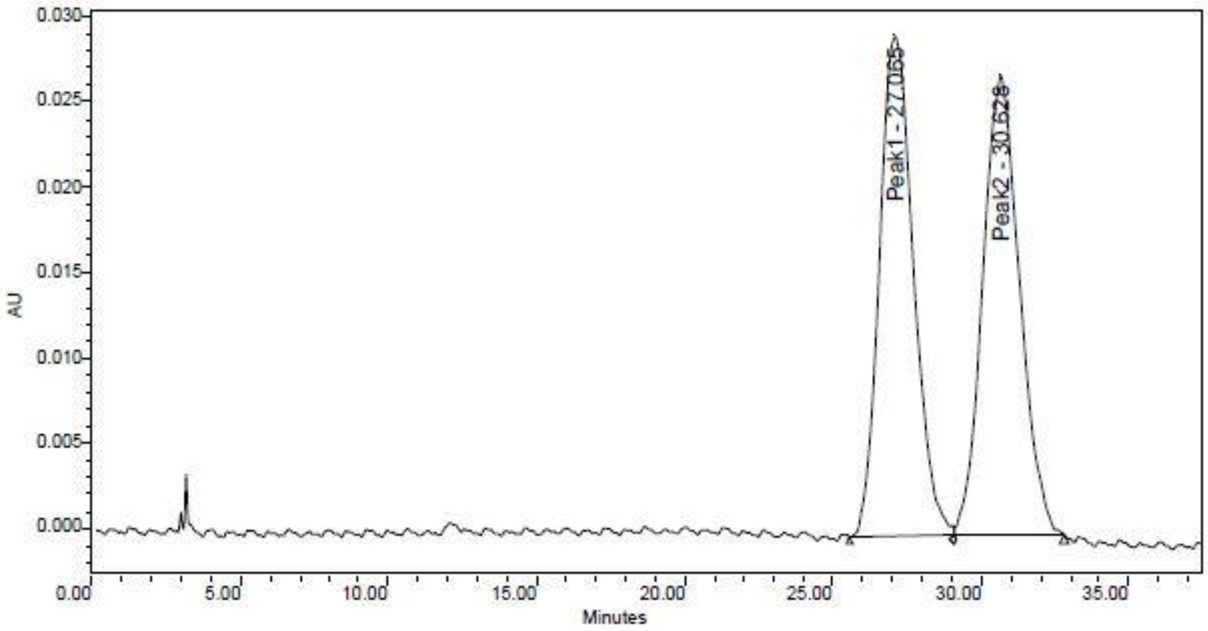

	Peak Name	RT (min)	Area (□V*sec)	% Area	Height (□V)	% Height
1	Peak1	27.065	2355899	50.07	29346	52.05
2	Peak2	30.628	2349467	49.93	27035	47.95

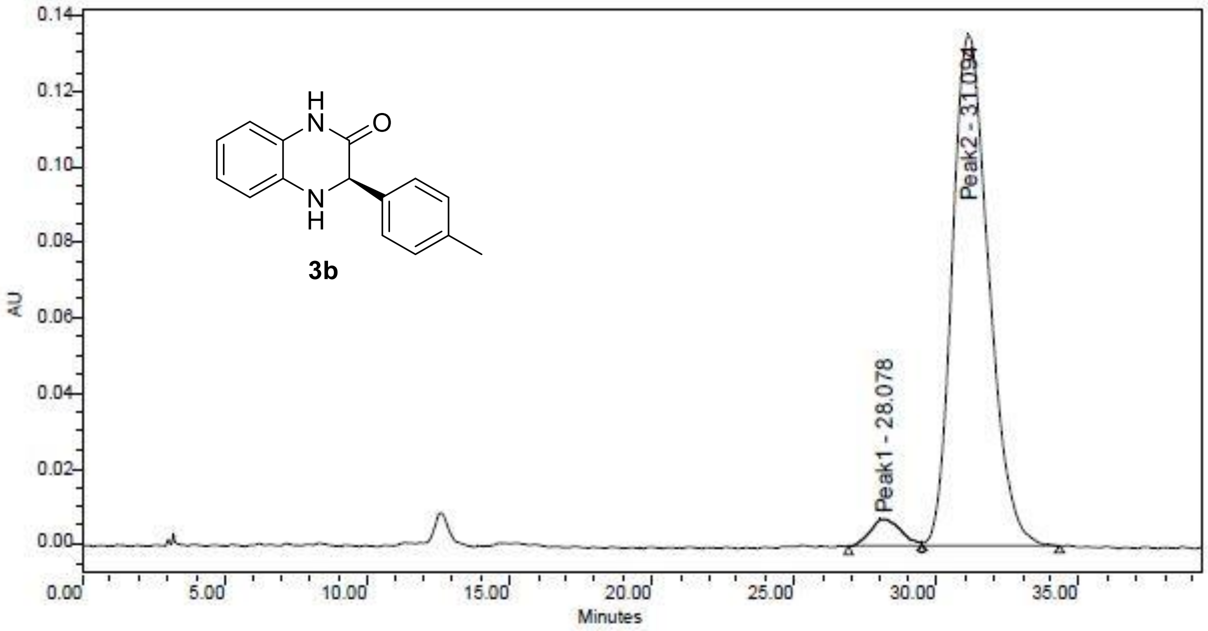

	Peak Name	RT (min)	Area (□V*sec)	% Area	Height (□V)	% Height
1	Peak1	28.078	552703	4.38	7170	5.04
2	Peak2	31.094	12058681	95.62	135196	94.96

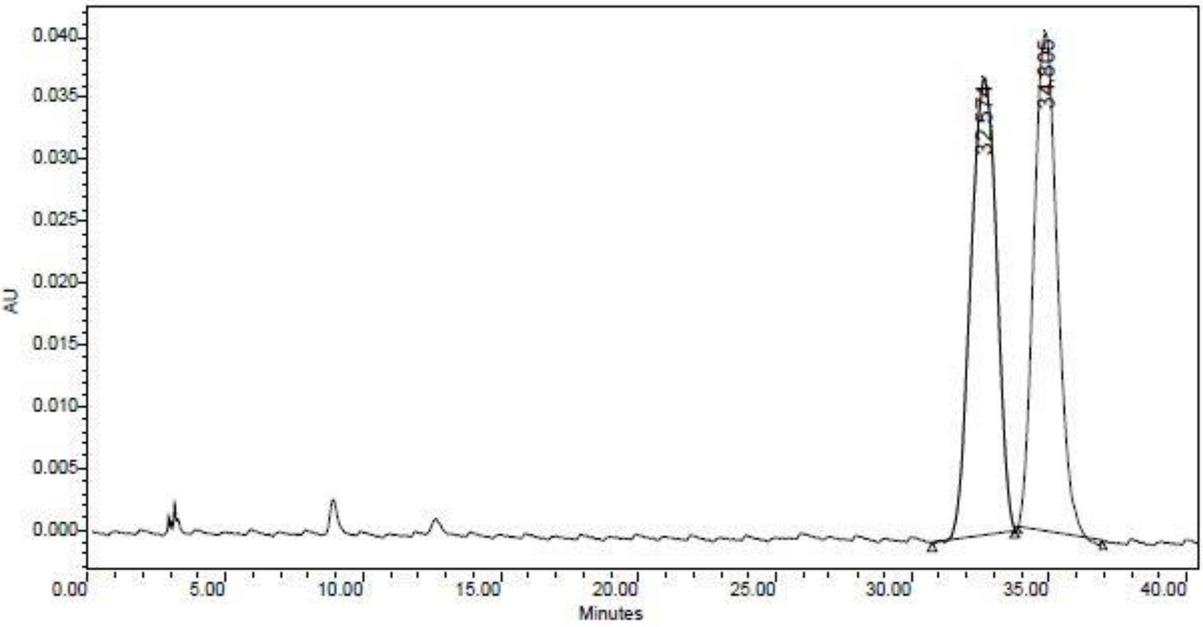

	RT (min)	Area (µV*sec)	% Area	Height (µV)	% Height
1	32.574	2438147	50.40	37148	47.88
2	34.805	2399389	49.60	40432	52.12

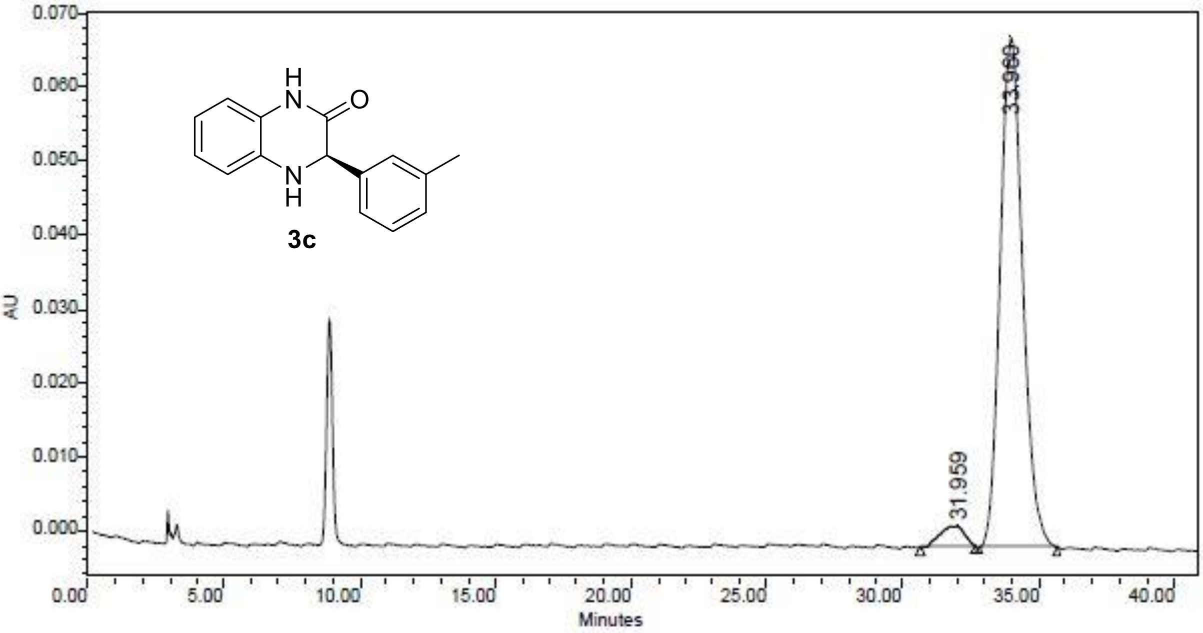

	RT (min)	Area (µV*sec)	% Area	Height (µV)	% Height
1	31.959	177357	4.29	2786	3.89
2	33.960	3959913	95.71	68816	96.11

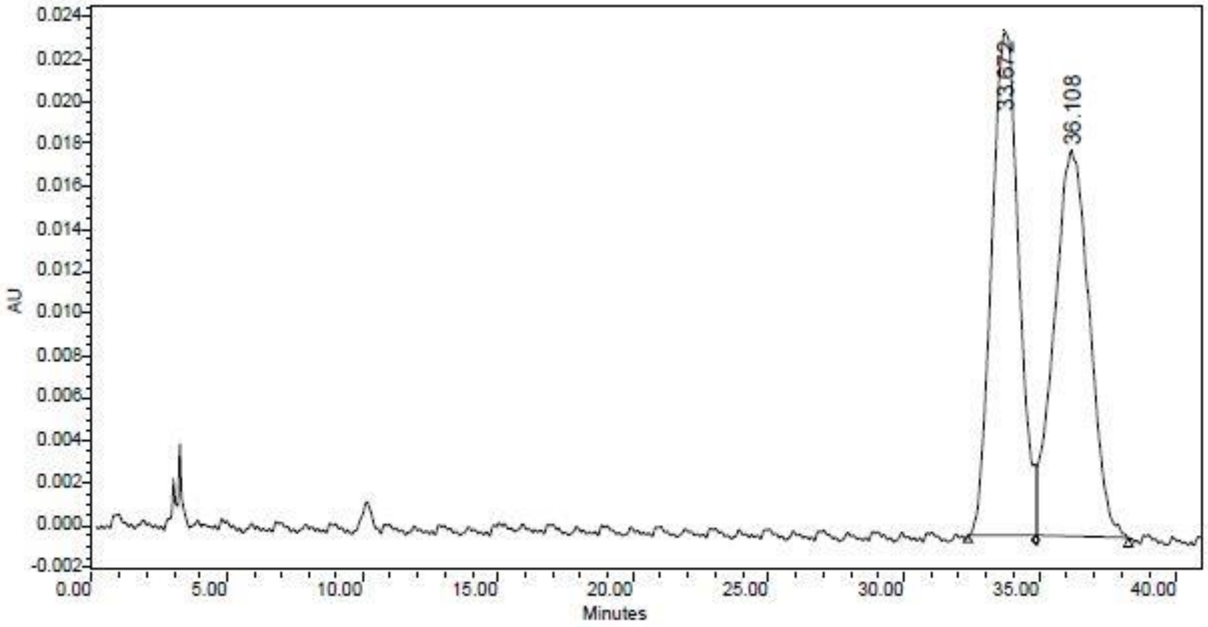

	RT (min)	Area (μV*sec)	% Area	Height (μV)	% Height
1	33.672	1660582	49.96	23775	56.59
2	36.108	1663296	50.04	18236	43.41

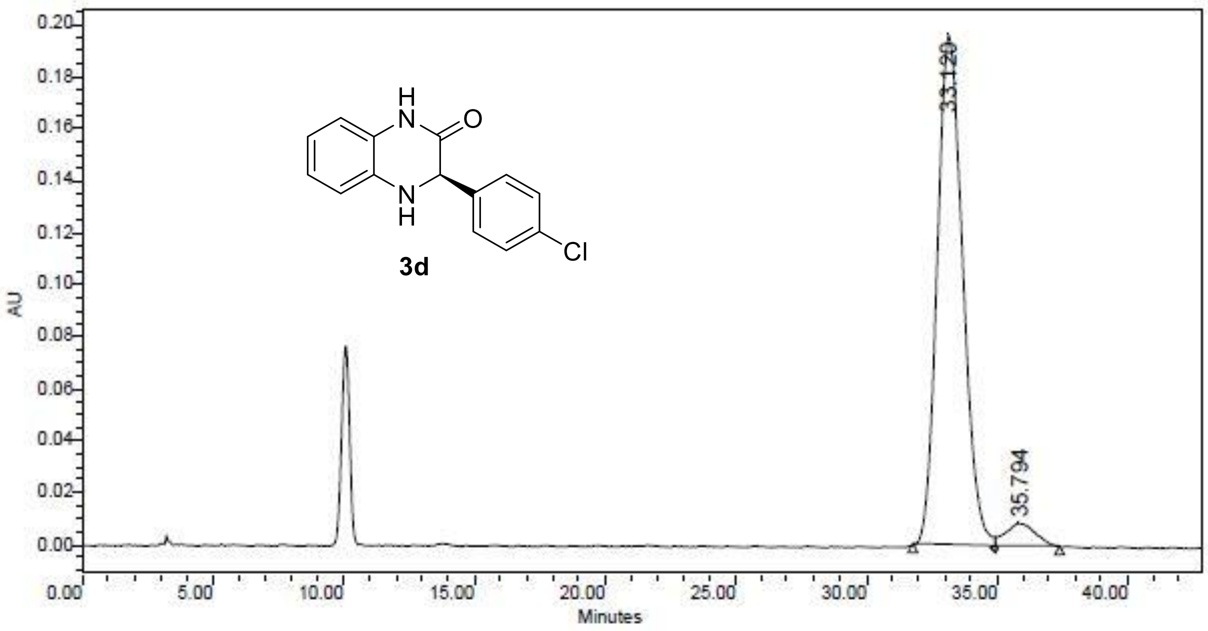

	RT (min)	Area (μV*sec)	% Area	Height (μV)	% Height
1	33.120	13338147	95.00	196197	95.72
2	35.794	702310	5.00	8769	4.28

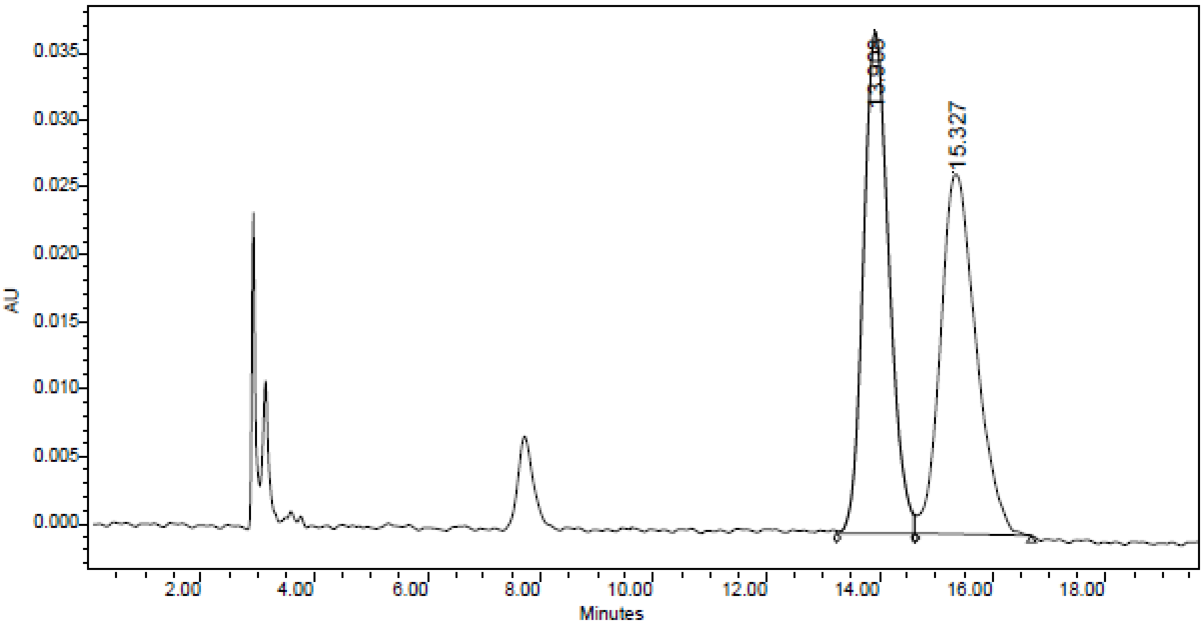

	RT (min)	Area (µV*sec)	% Area	Height (µV)	% Height
1	13.908	1182008	50.07	37399	58.21
2	15.327	1178921	49.93	26845	41.79

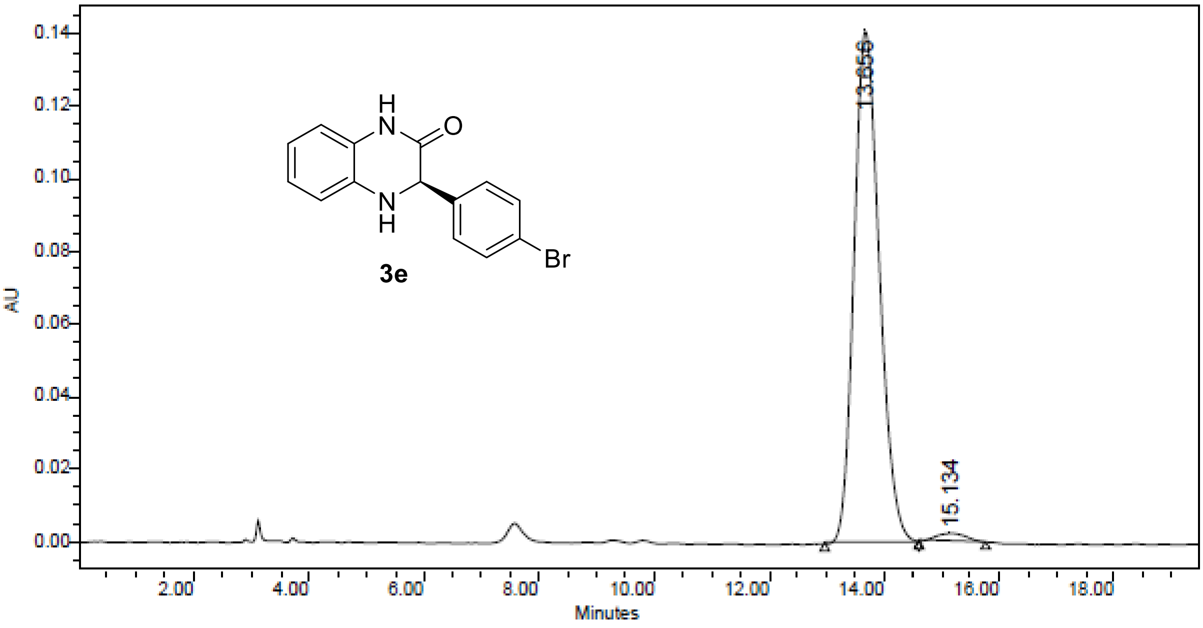

	RT (min)	Area (µV*sec)	% Area	Height (µV)	% Height
1	13.656	4361737	98.15	141525	98.43
2	15.134	82428	1.85	2252	1.57

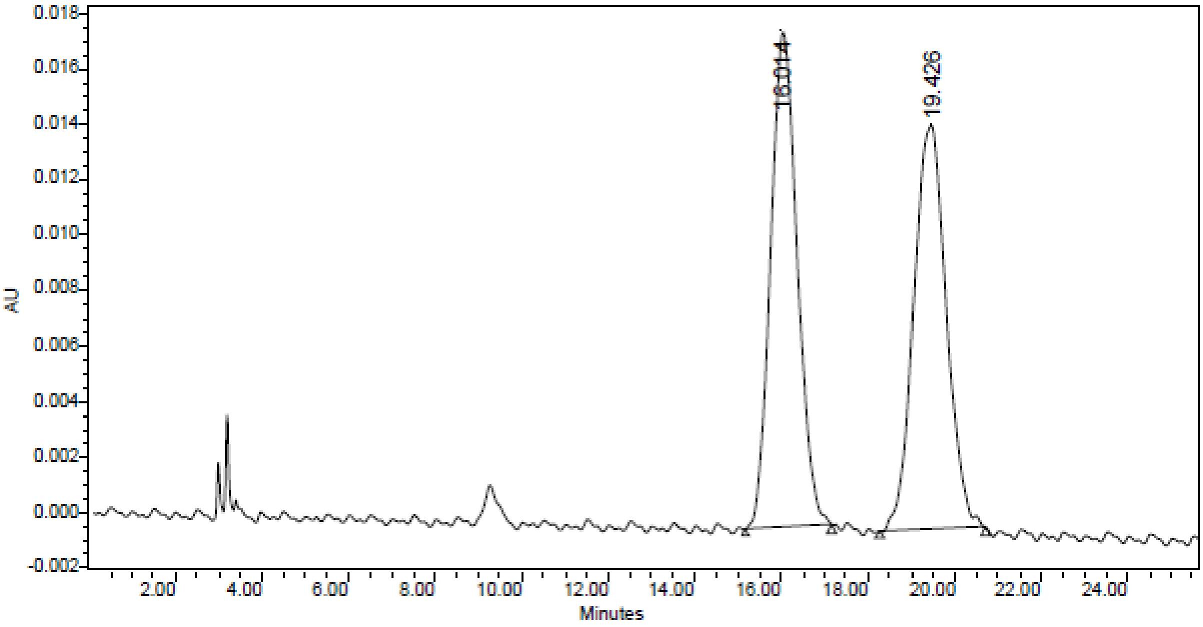

	RT (min)	Area (□V*sec)	% Area	Height (□V)	% Height
1	16.014	767341	50.06	17893	55.05
2	19.426	765358	49.94	14612	44.95

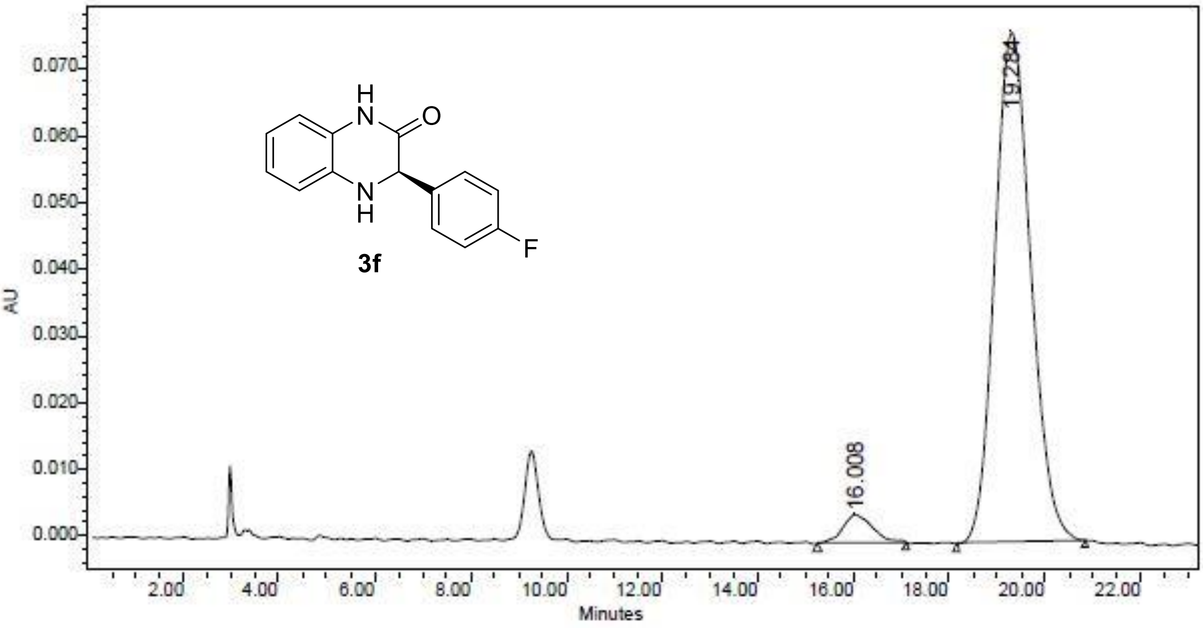

	RT (min)	Area (□V*sec)	% Area	Height (□V)	% Height
1	16.008	188340	4.50	4270	5.30
2	19.284	3995208	95.50	76249	94.70

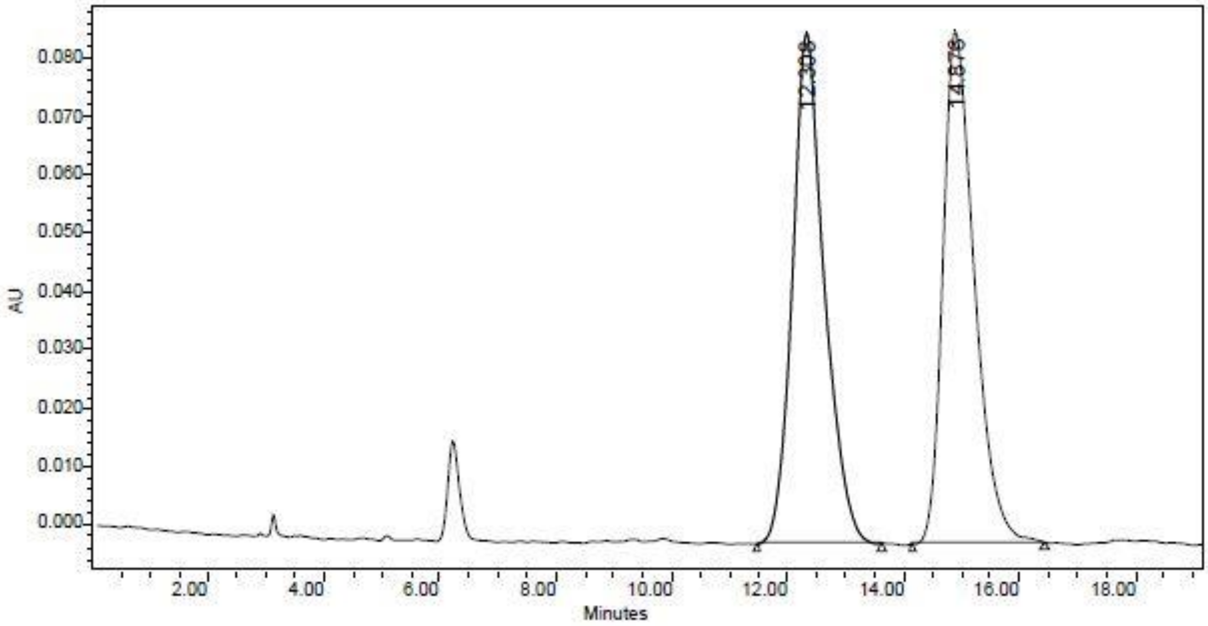

	RT (min)	Area (μV*sec)	% Area	Height (μV)	% Height
1	12.308	3348718	50.24	87460	49.91
2	14.876	3317013	49.76	87776	50.09

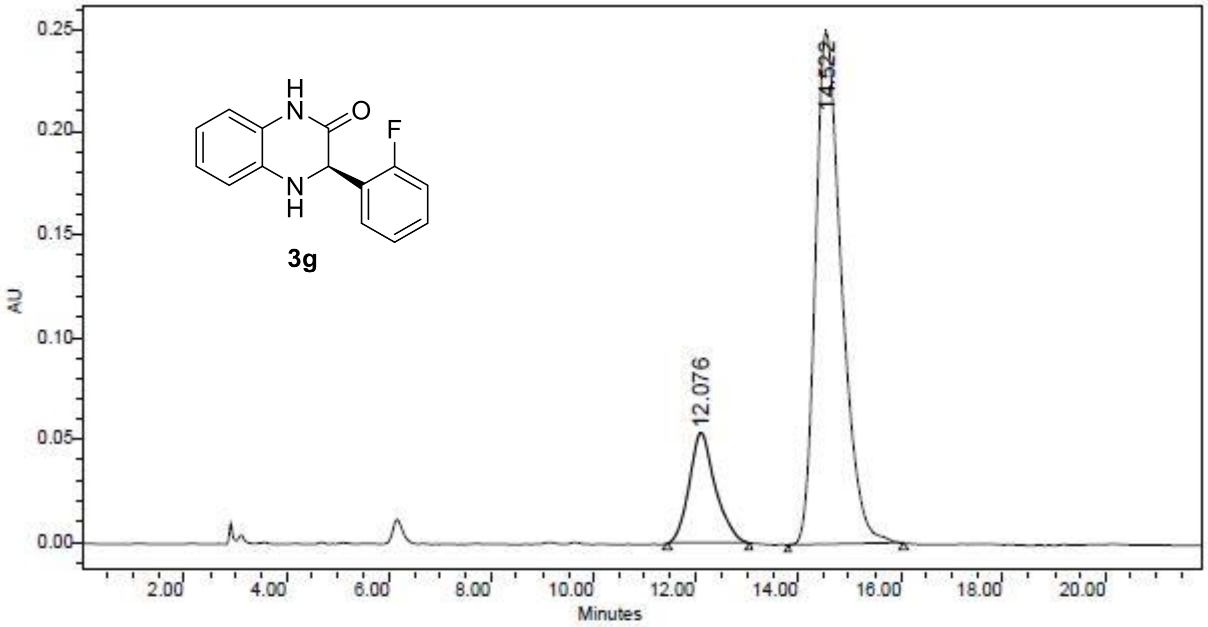

	RT (min)	Area (μV*sec)	% Area	Height (μV)	% Height
1	12.076	1917336	17.74	53944	17.76
2	14.522	8891666	82.26	249791	82.24

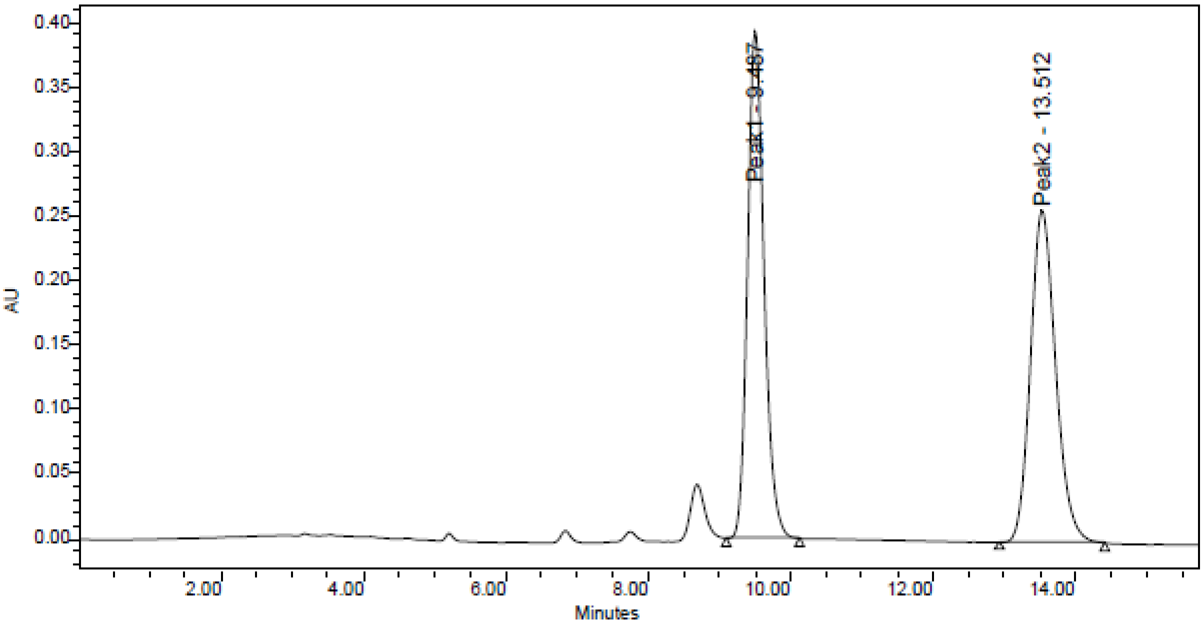

	Peak Name	RT (min)	Area (□V*sec)	% Area	Height (□V)	% Height
1	Peak1	9.487	6363236	49.99	393154	60.42
2	Peak2	13.512	6365740	50.01	257566	39.58

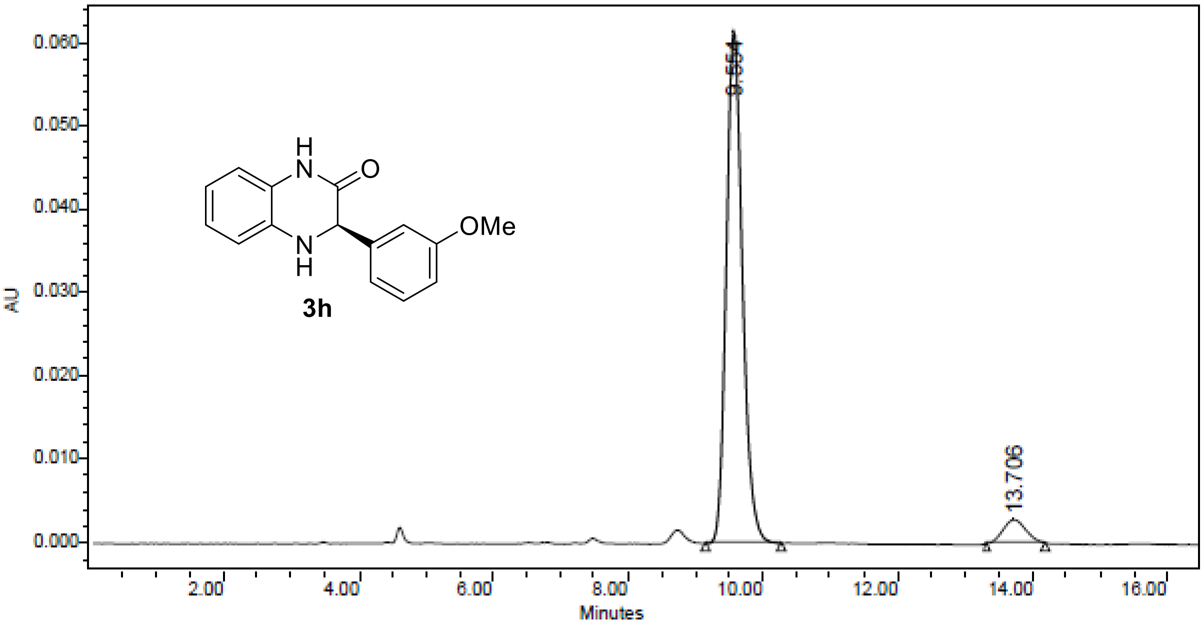

	RT (min)	Area (□V*sec)	% Area	Height (□V)	% Height
1	9.554	1025139	93.85	61495	95.60
2	13.706	67179	6.15	2831	4.40

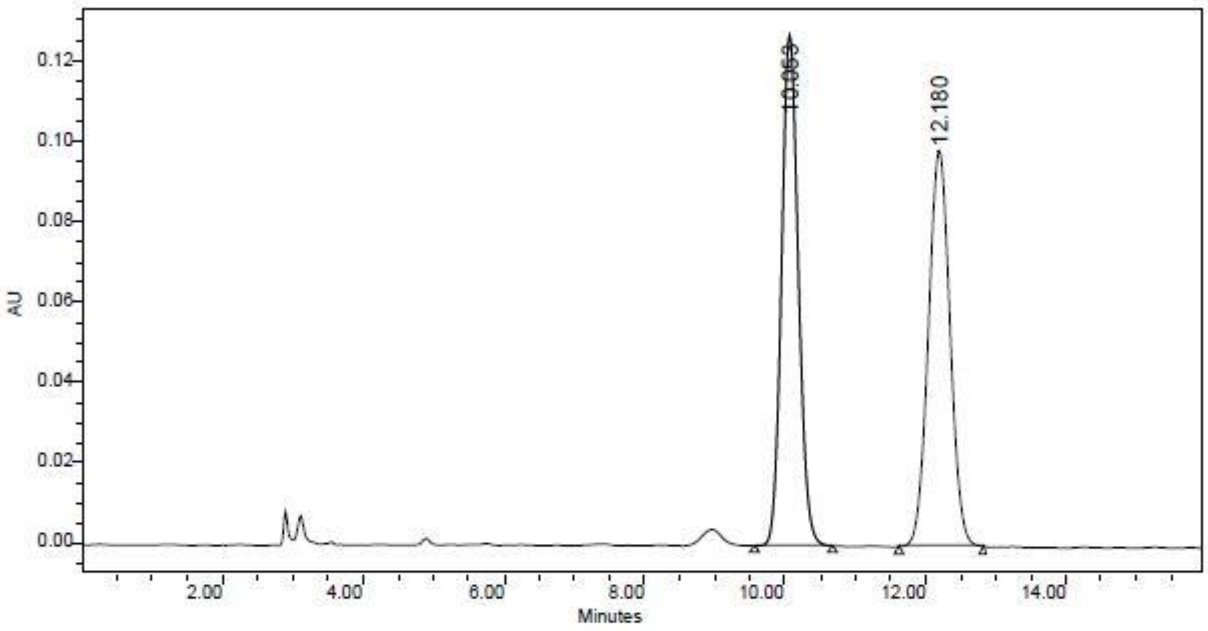

	RT (min)	Area (□V*sec)	% Area	Height (□V)	% Height
1	10.053	2104842	50.01	126846	56.34
2	12.180	2103768	49.99	98288	43.66

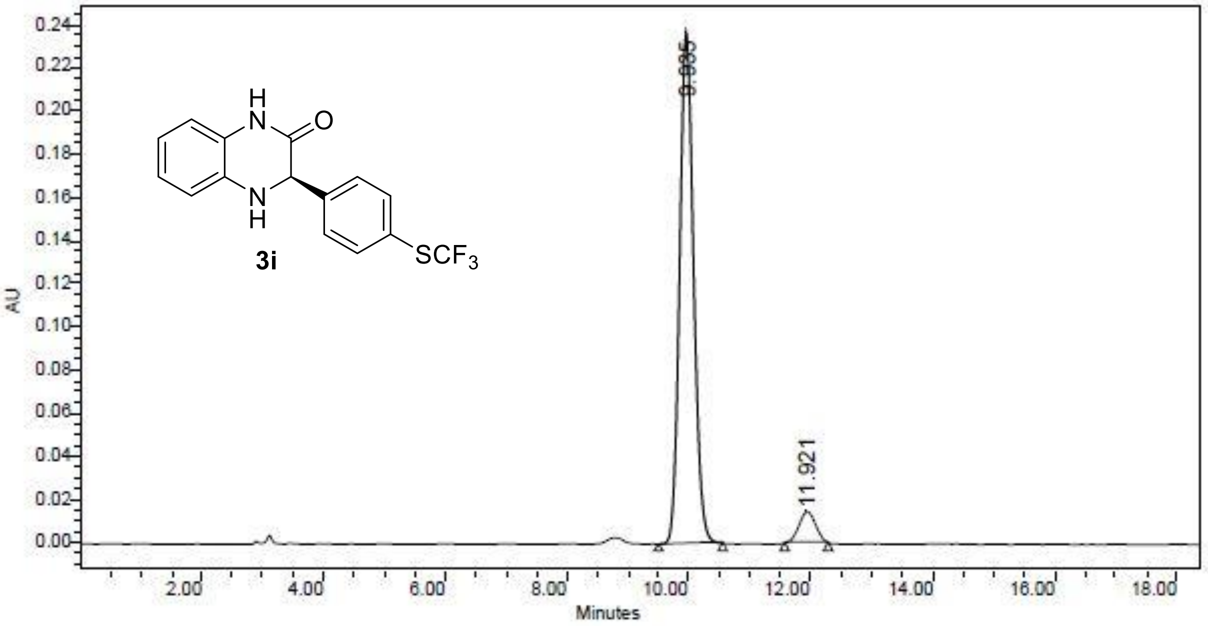

	RT (min)	Area (□V*sec)	% Area	Height (□V)	% Height
1	9.935	3762319	93.06	237122	94.19
2	11.921	280604	6.94	14614	5.81

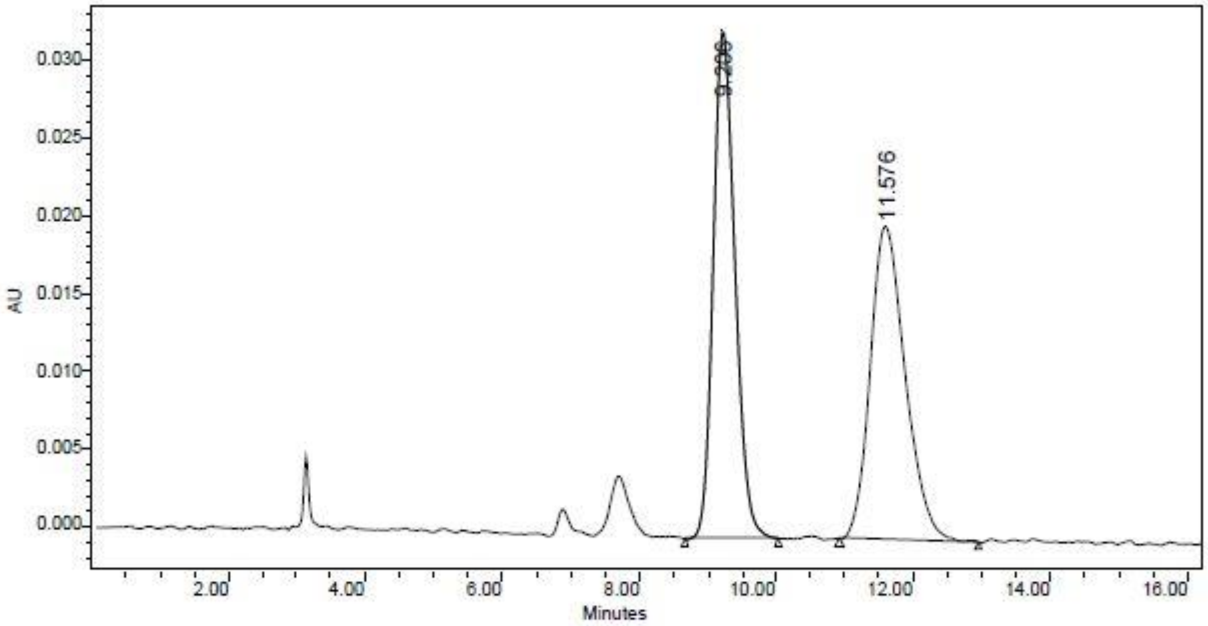

	RT (min)	Area (µV*sec)	% Area	Height (µV)	% Height
1	9.206	720963	50.00	32678	61.80
2	11.576	720921	50.00	20202	38.20

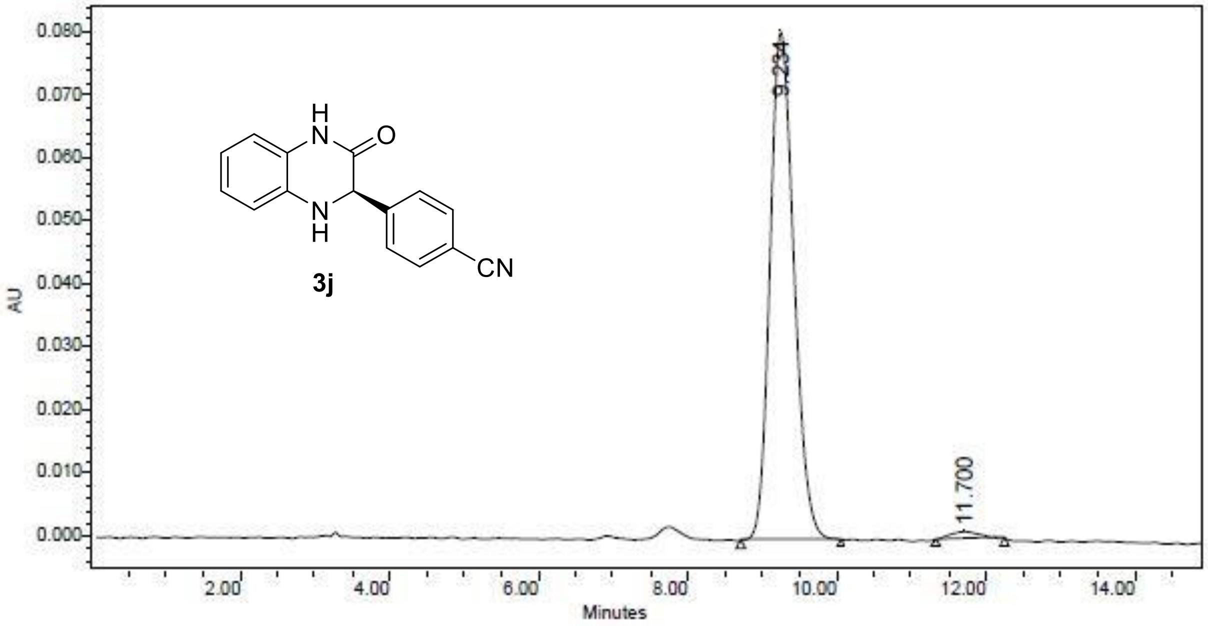

	RT (min)	Area (µV*sec)	% Area	Height (µV)	% Height
1	9.234	1800957	98.14	80644	98.53
2	11.700	34221	1.86	1206	1.47

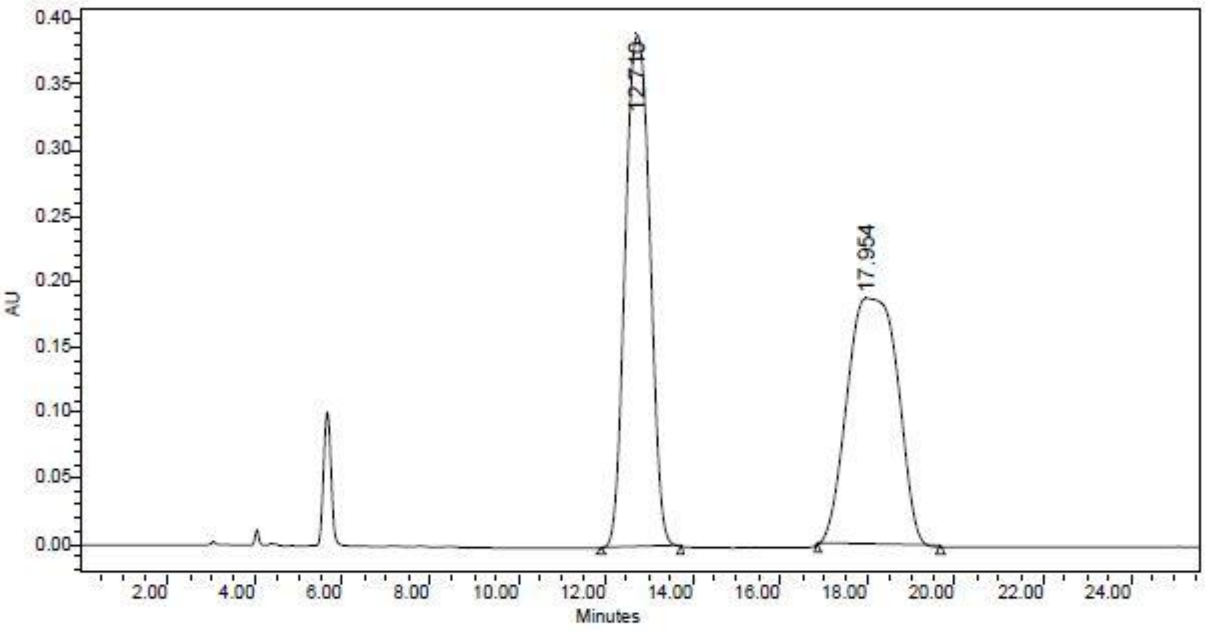

	RT (min)	Area (μV*sec)	% Area	Height (μV)	% Height
1	12.710	15317330	50.22	390527	67.45
2	17.954	15185487	49.78	188492	32.55

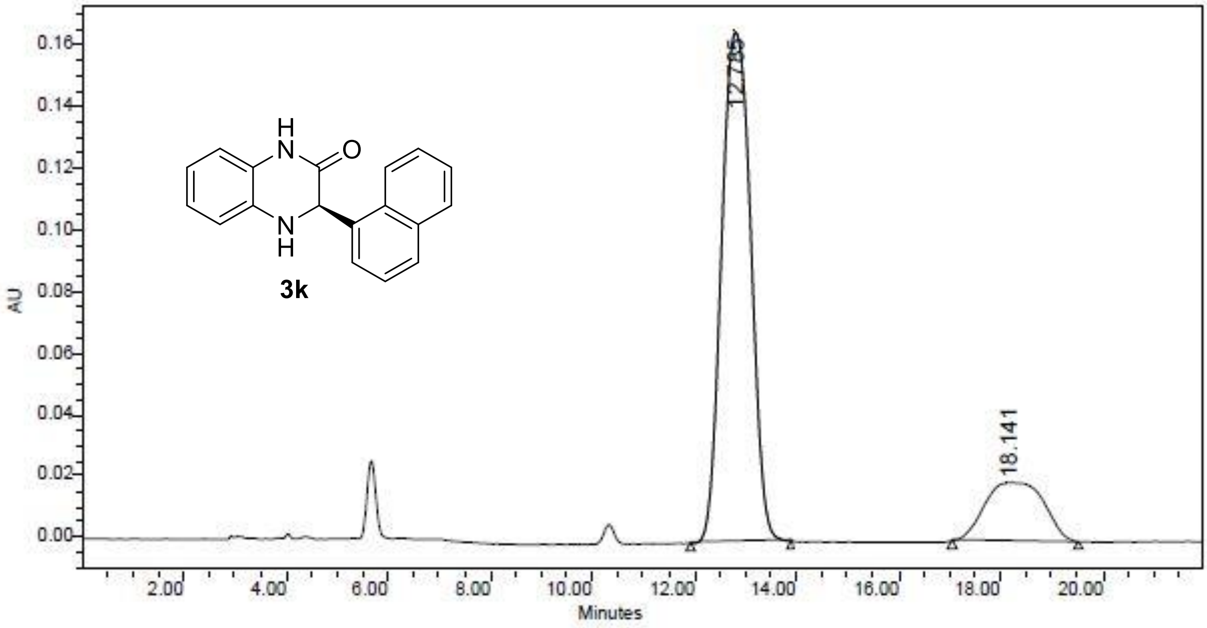

	RT (min)	Area (μV*sec)	% Area	Height (μV)	% Height
1	12.785	6585032	81.17	165773	89.75
2	18.141	1528079	18.83	18924	10.25

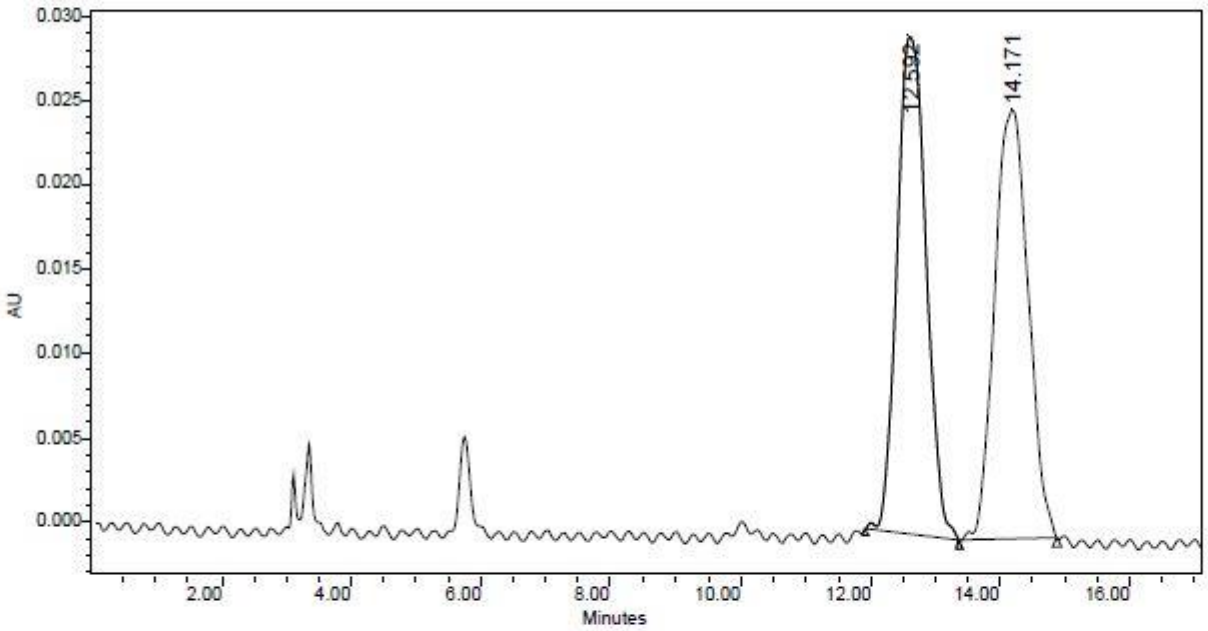

	RT (min)	Area (□V*sec)	% Area	Height (□V)	% Height
1	12.592	922879	49.93	29511	53.66
2	14.171	925510	50.07	25489	46.34

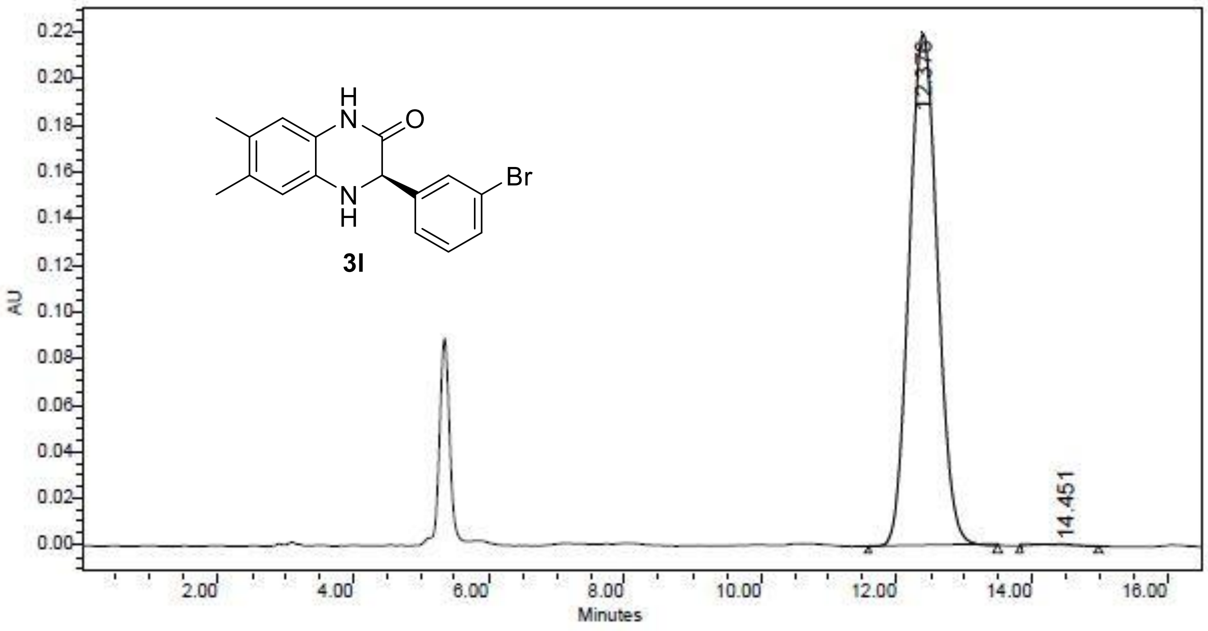

	RT (min)	Area (□V*sec)	% Area	Height (□V)	% Height
1	12.376	6370725	99.54	219916	99.60
2	14.451	29385	0.46	873	0.40

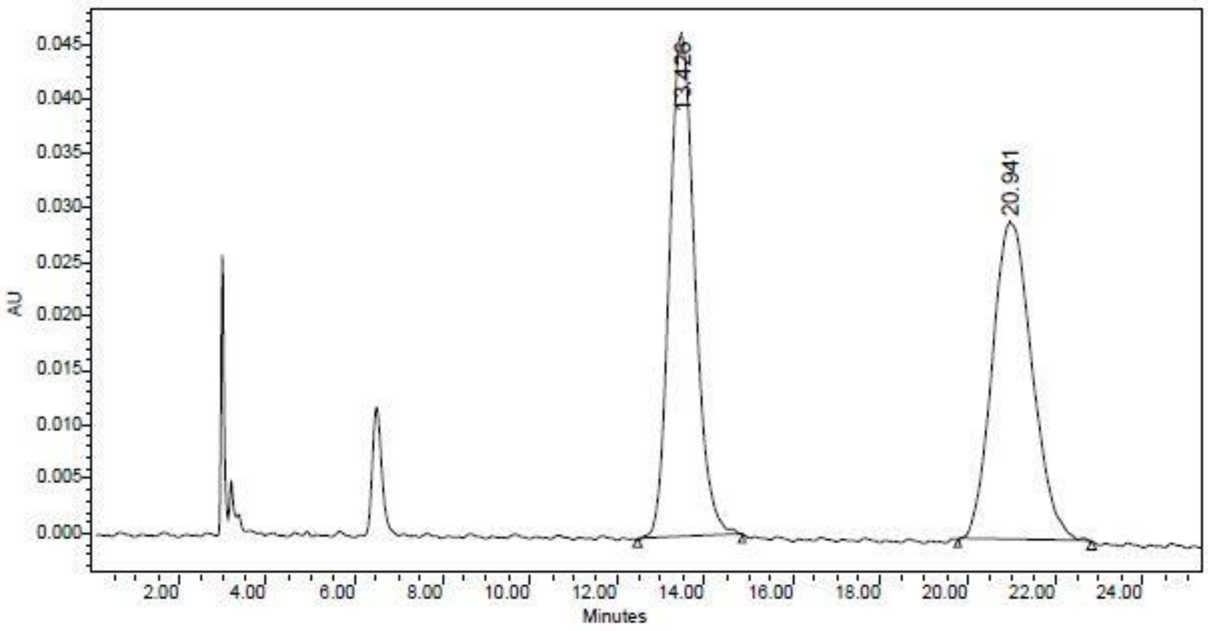

	RT (min)	Area (▯V*sec)	% Area	Height (▯V)	% Height
1	13.426	1878517	50.17	46276	61.32
2	20.941	1865623	49.83	29196	38.68

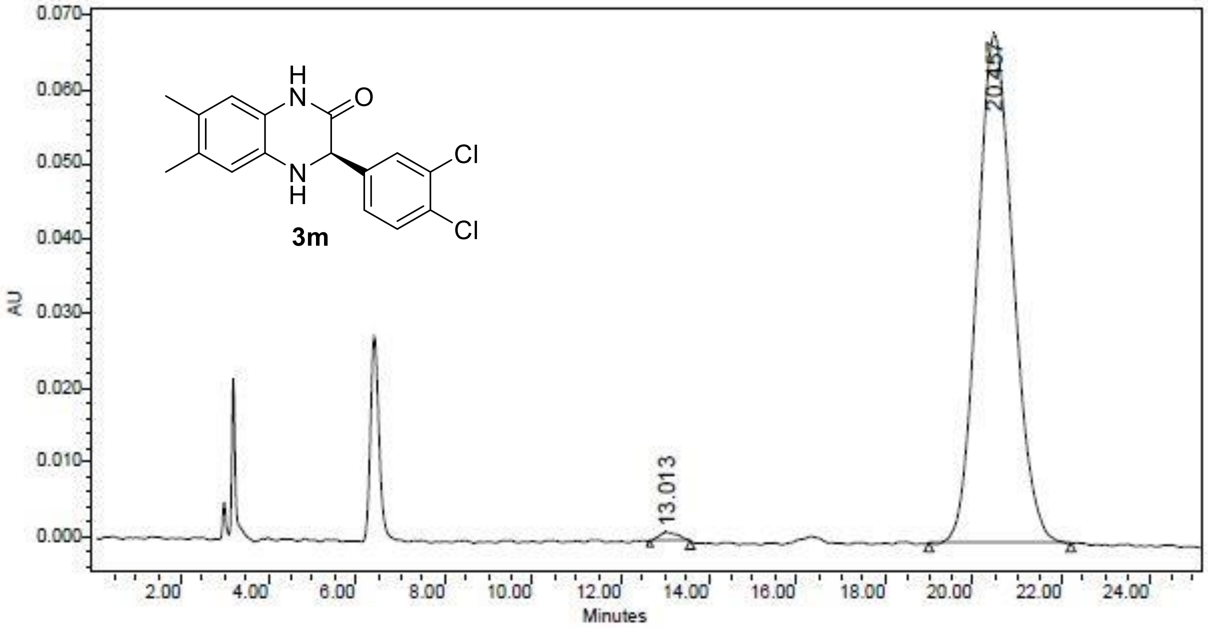

	RT (min)	Area (▯V*sec)	% Area	Height (▯V)	% Height
1	13.013	39080	1.00	1224	1.76
2	20.457	3857706	99.00	68145	98.24

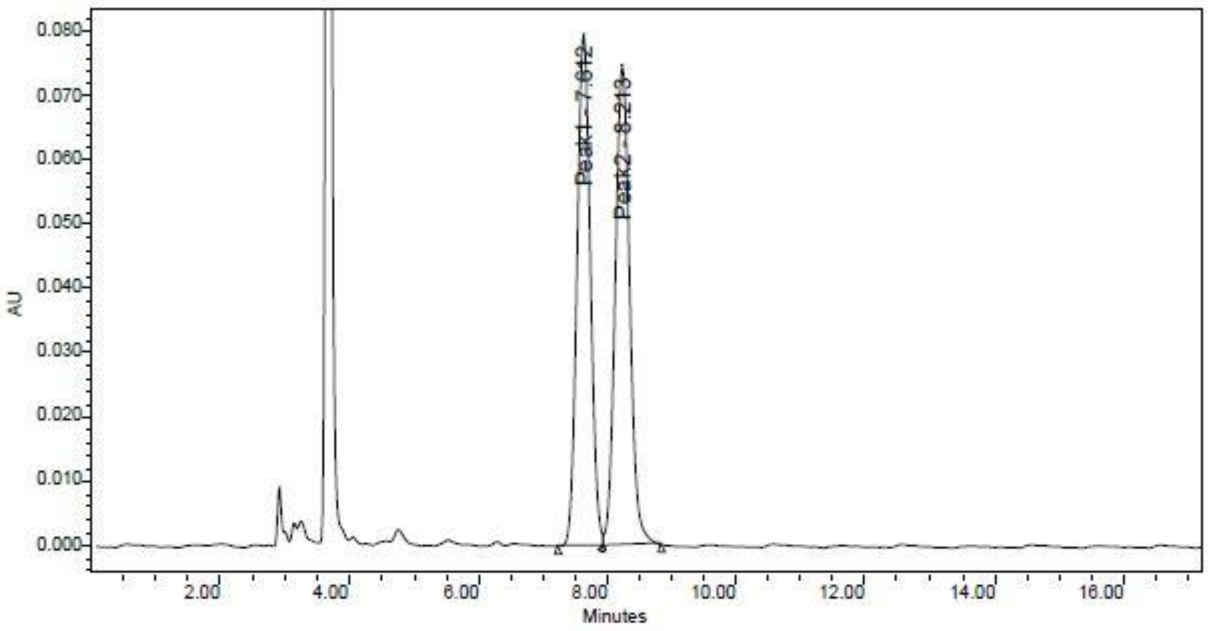

	Peak Name	RT (min)	Area (□V*sec)	% Area	Height (□V)	% Height
1	Peak1	7.612	1142978	49.56	79611	51.61
2	Peak2	8.213	1163164	50.44	74633	48.39

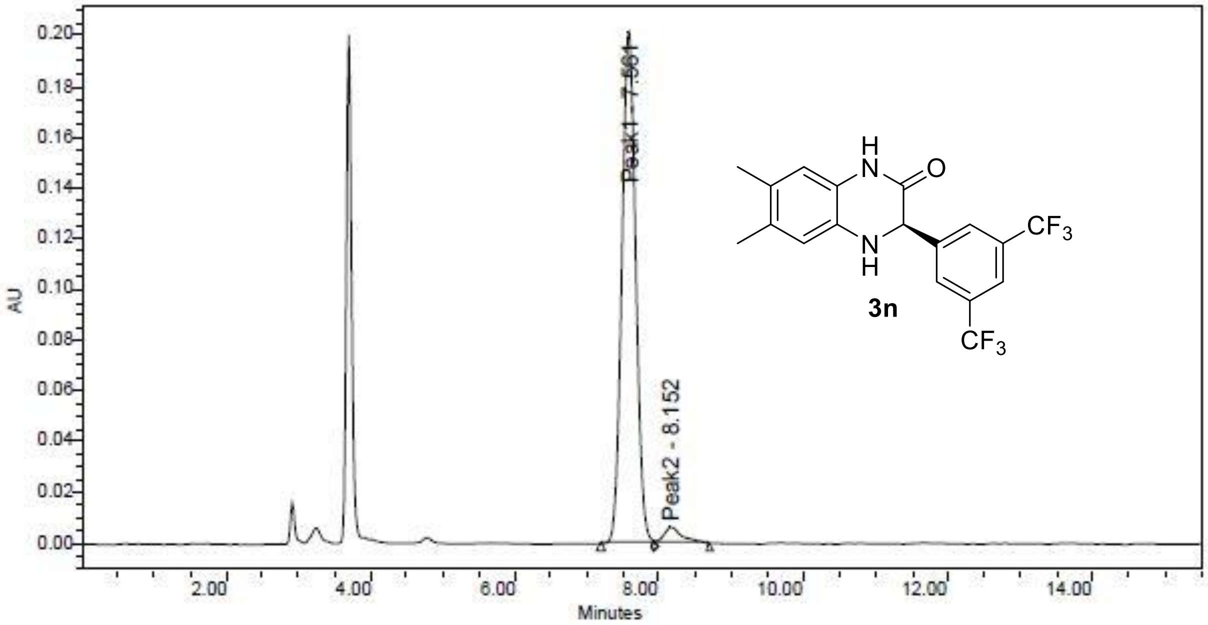

	Peak Name	RT (min)	Area (□V*sec)	% Area	Height (□V)	% Height
1	Peak1	7.561	2699839	95.88	201962	96.94
2	Peak2	8.152	116159	4.12	6366	3.06

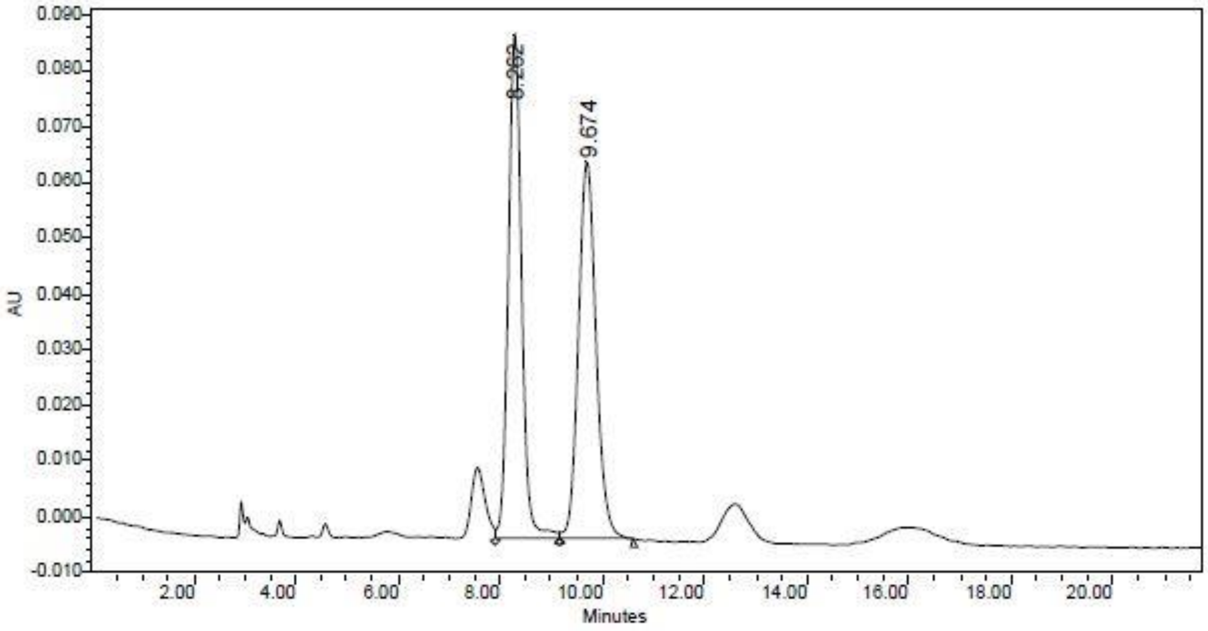

	RT (min)	Area (µV*sec)	% Area	Height (µV)	% Height
1	8.262	1640320	50.22	90405	57.19
2	9.674	1626261	49.78	67685	42.81

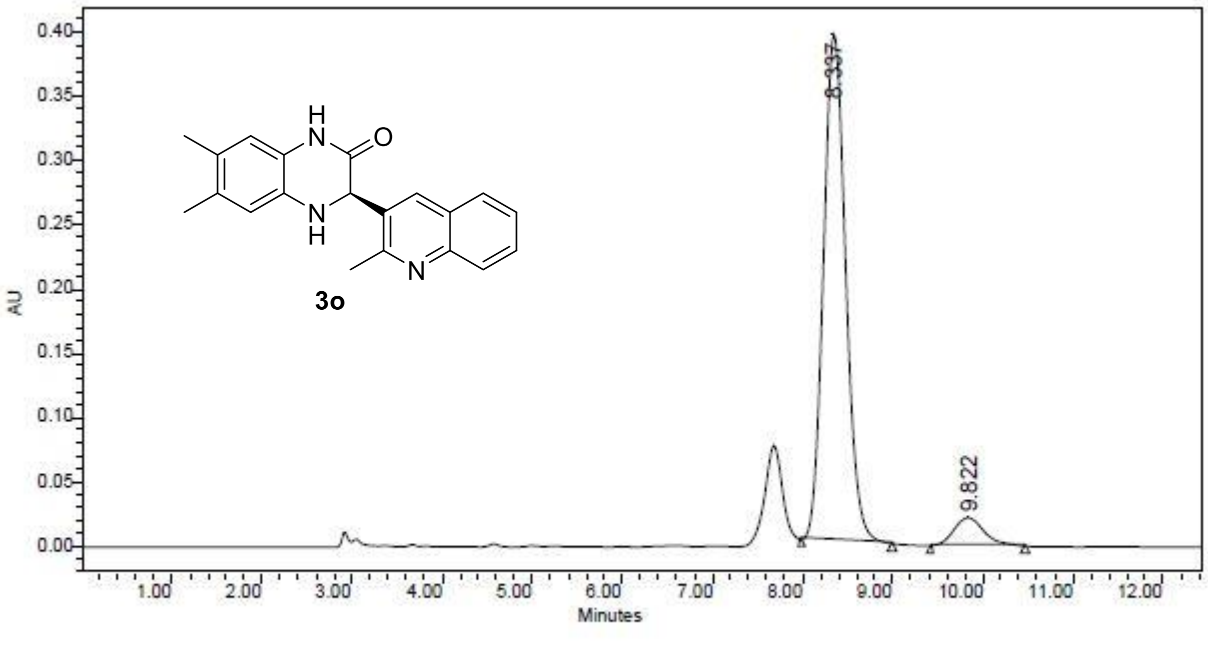

	RT (min)	Area (µV*sec)	% Area	Height (µV)	% Height
1	8.337	6743965	92.92	394580	94.74
2	9.822	513891	7.08	21908	5.26

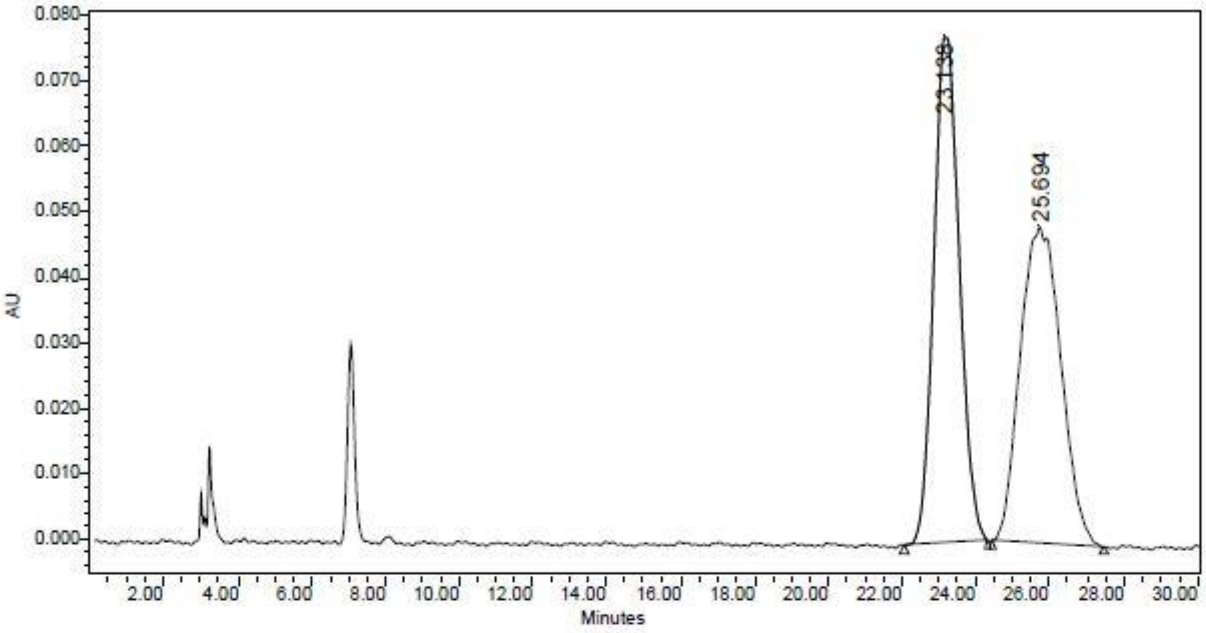

	RT (min)	Area (□V*sec)	% Area	Height (□V)	% Height
1	23.138	3728875	50.09	77349	61.55
2	25.694	3715950	49.91	48327	38.45

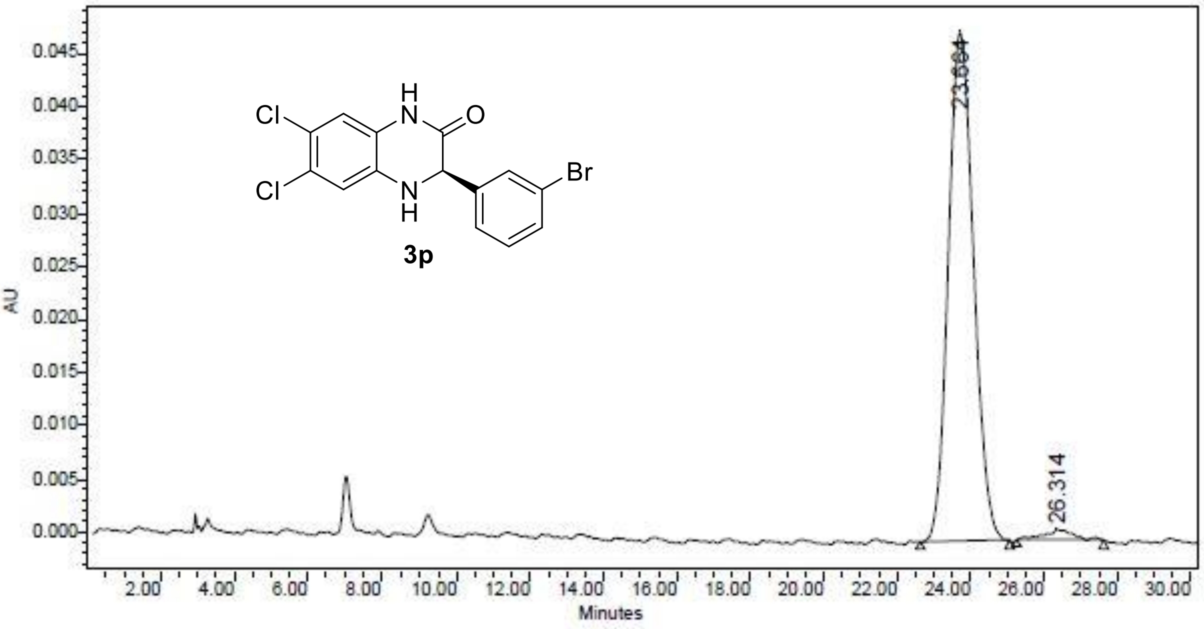

	RT (min)	Area (□V*sec)	% Area	Height (□V)	% Height
1	23.664	2291564	97.44	47920	97.86
2	26.314	60207	2.56	1046	2.14

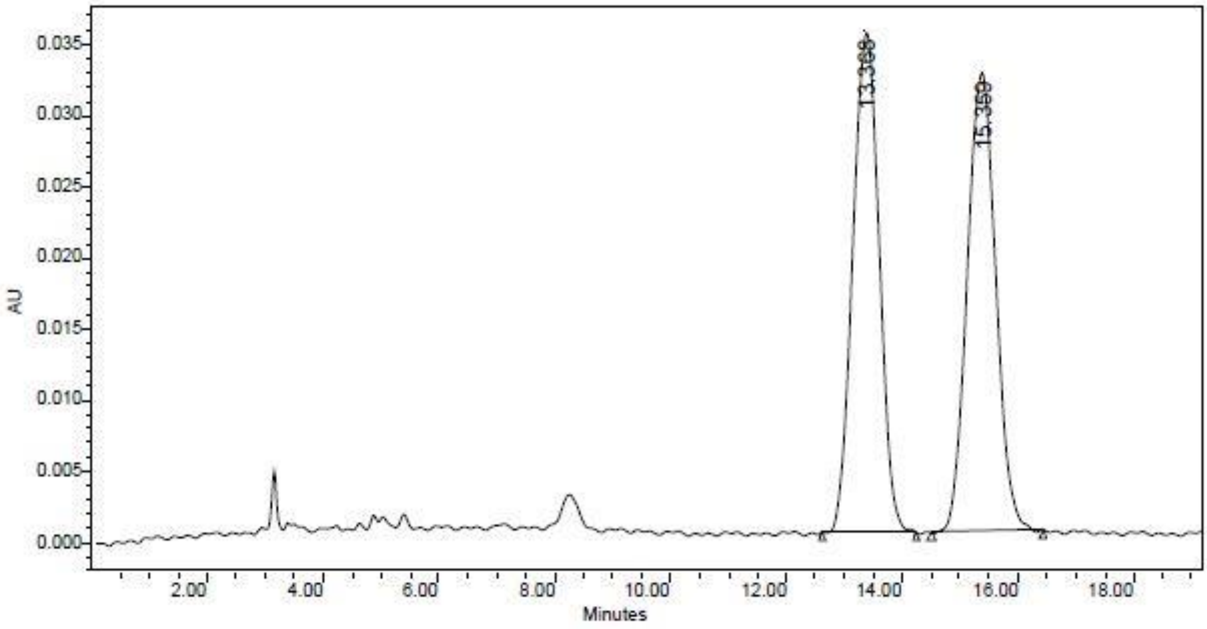

	RT (min)	Area (µV*sec)	% Area	Height (µV)	% Height
1	13.368	1118644	50.19	35161	52.14
2	15.359	1110296	49.81	32278	47.86

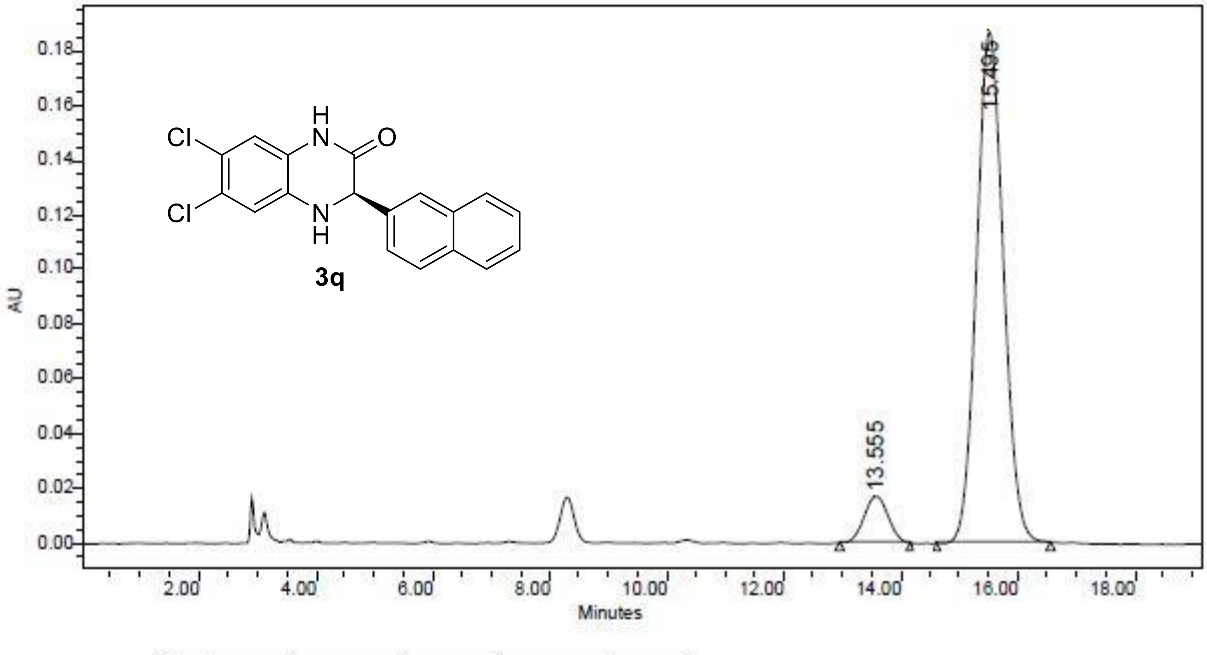

	RT (min)	Area (µV*sec)	% Area	Height (µV)	% Height
1	13.555	486990	7.55	17015	8.33
2	15.495	5966414	92.45	187203	91.67

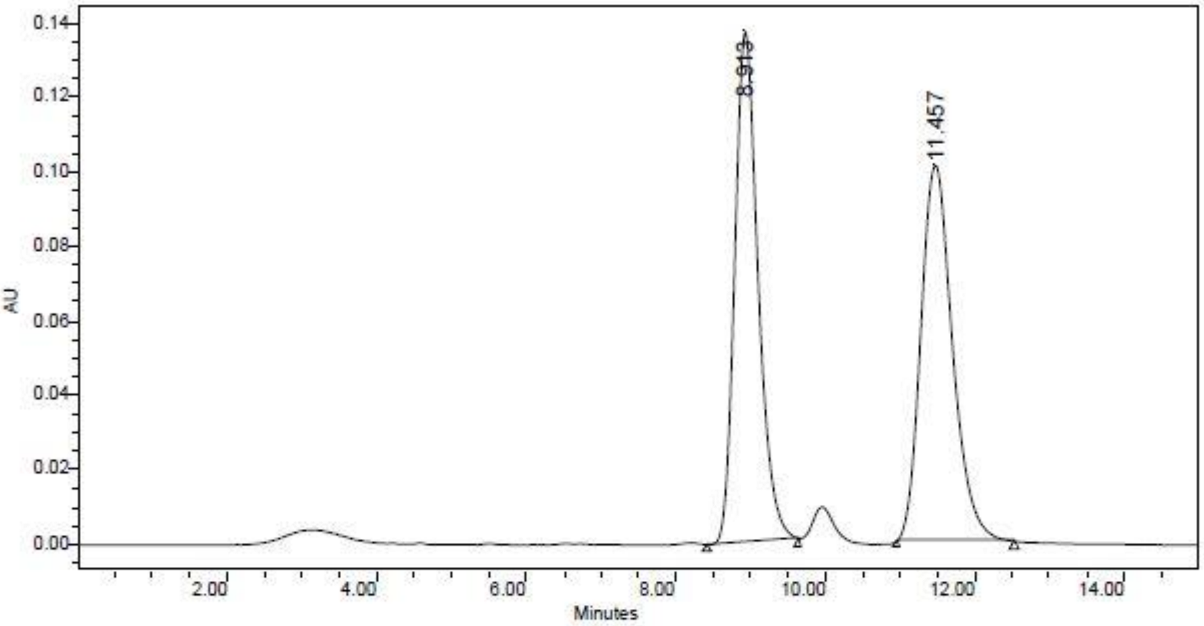

	RT (min)	Area (□V*sec)	% Area	Height (□V)	% Height
1	8.913	2967404	49.97	137140	57.61
2	11.457	2971465	50.03	100919	42.39

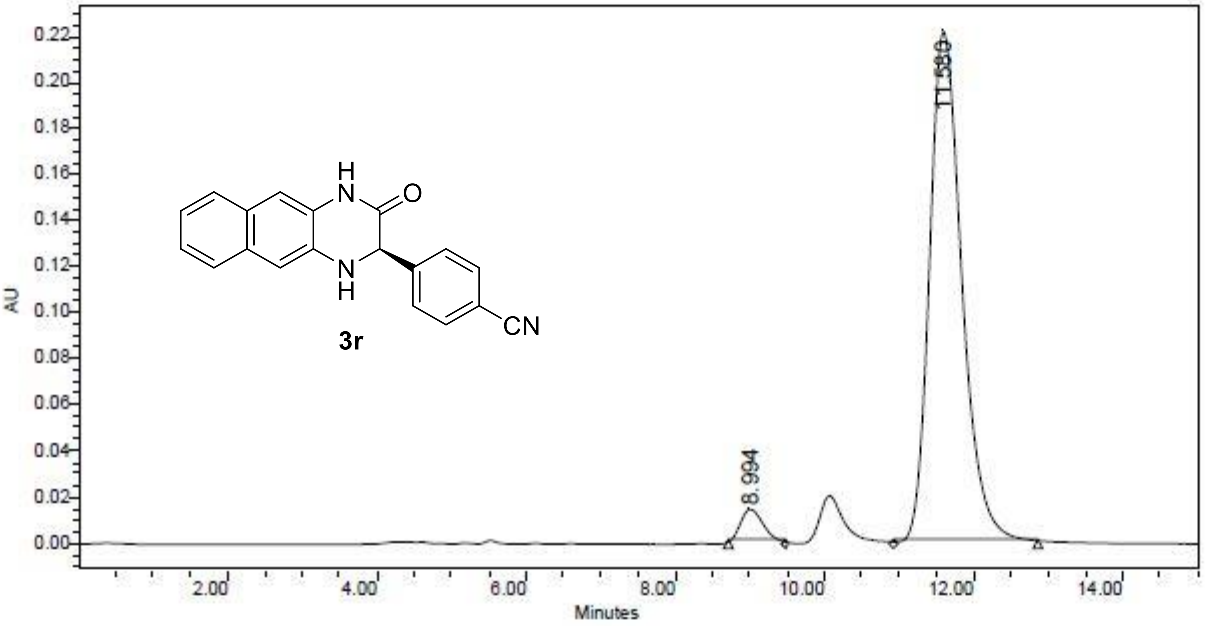

	RT (min)	Area (□V*sec)	% Area	Height (□V)	% Height
1	8.994	279055	4.04	13819	5.88
2	11.580	6634755	95.96	221165	94.12

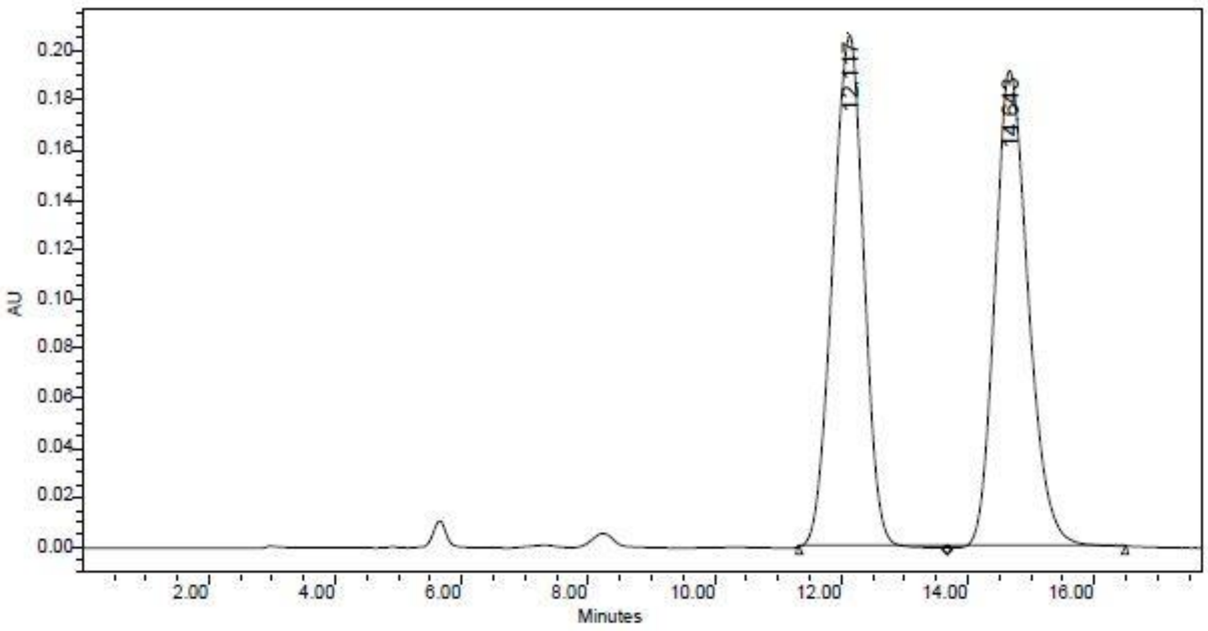

	RT (min)	Area (□V*sec)	% Area	Height (□V)	% Height
1	12.117	7060906	50.09	206178	51.79
2	14.643	7034198	49.91	191954	48.21

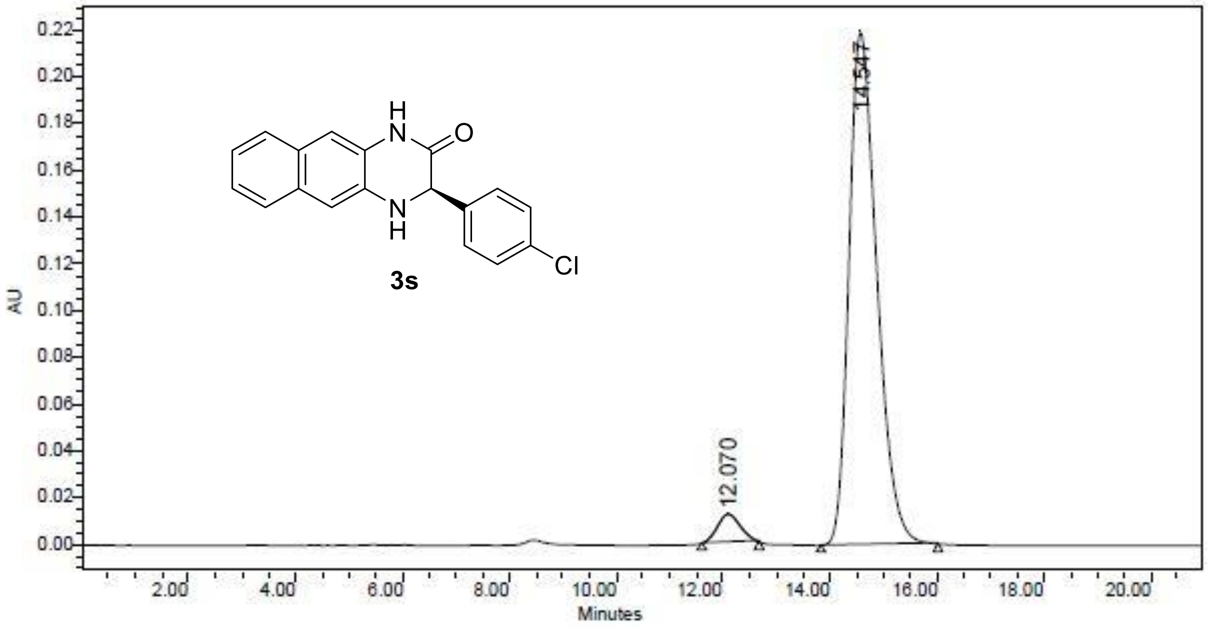

	RT (min)	Area (□V*sec)	% Area	Height (□V)	% Height
1	12.070	367977	4.43	12192	5.27
2	14.547	7942237	95.57	219249	94.73

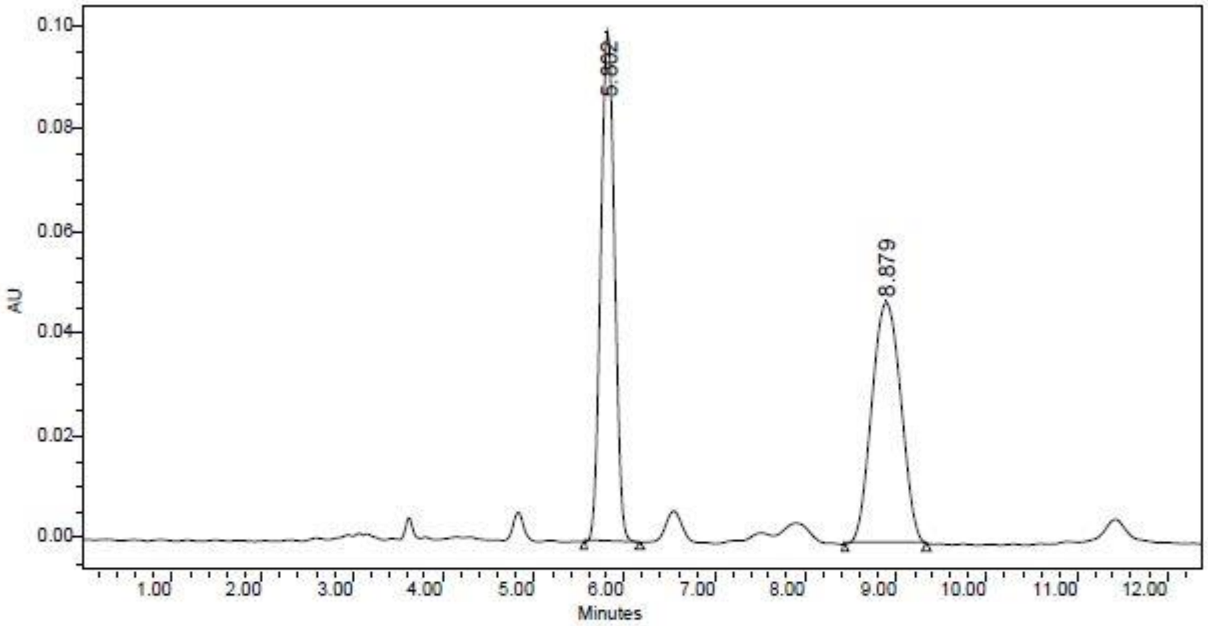

	RT (min)	Area (µV*sec)	% Area	Height (µV)	% Height
1	5.802	1061815	50.00	99555	67.83
2	8.879	1061664	50.00	47209	32.17

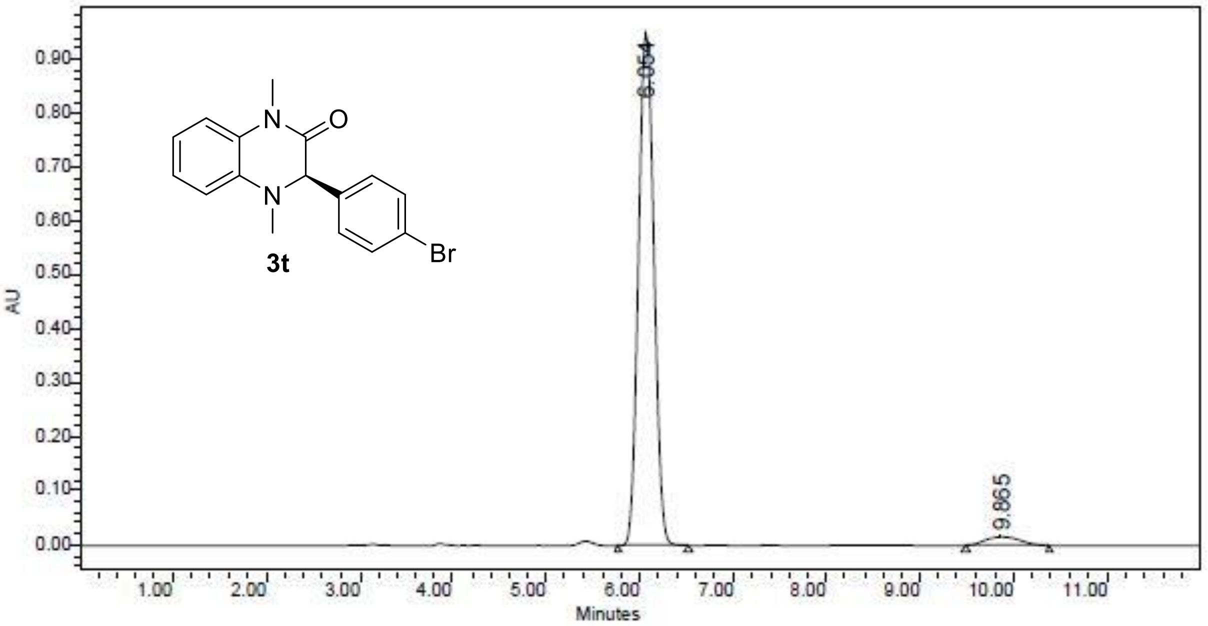

	RT (min)	Area (µV*sec)	% Area	Height (µV)	% Height
1	6.054	10961961	96.27	950608	98.35
2	9.865	425076	3.73	15978	1.65

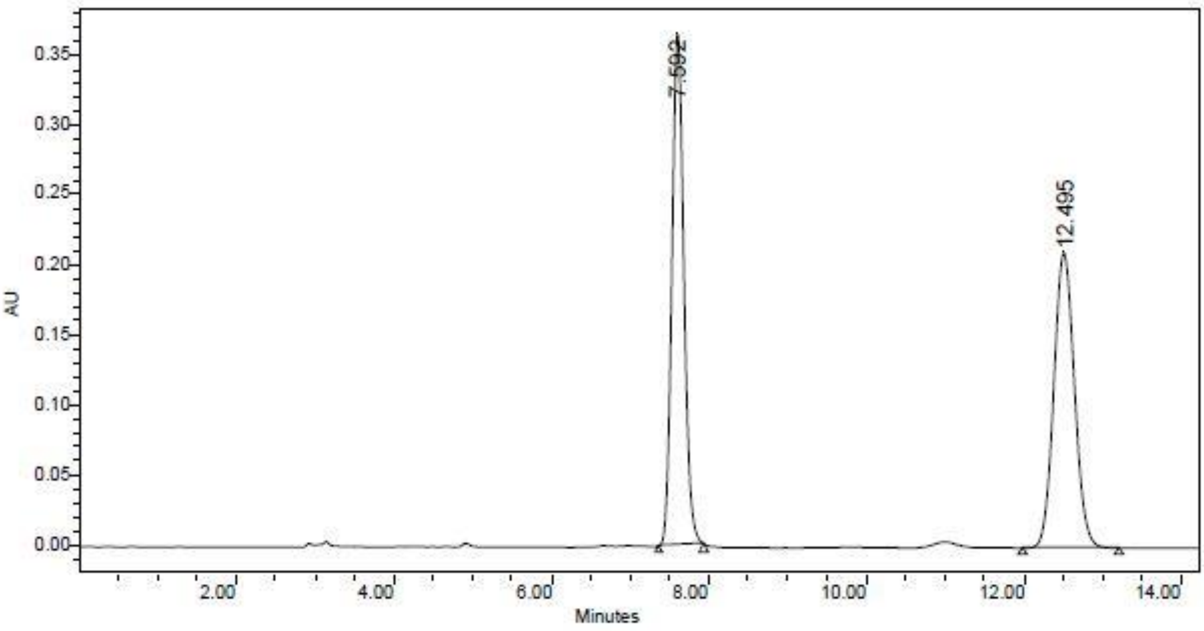

	RT (min)	Area (µV*sec)	% Area	Height (µV)	% Height
1	7.592	3834166	50.05	366375	63.48
2	12.495	3826021	49.95	210757	36.52

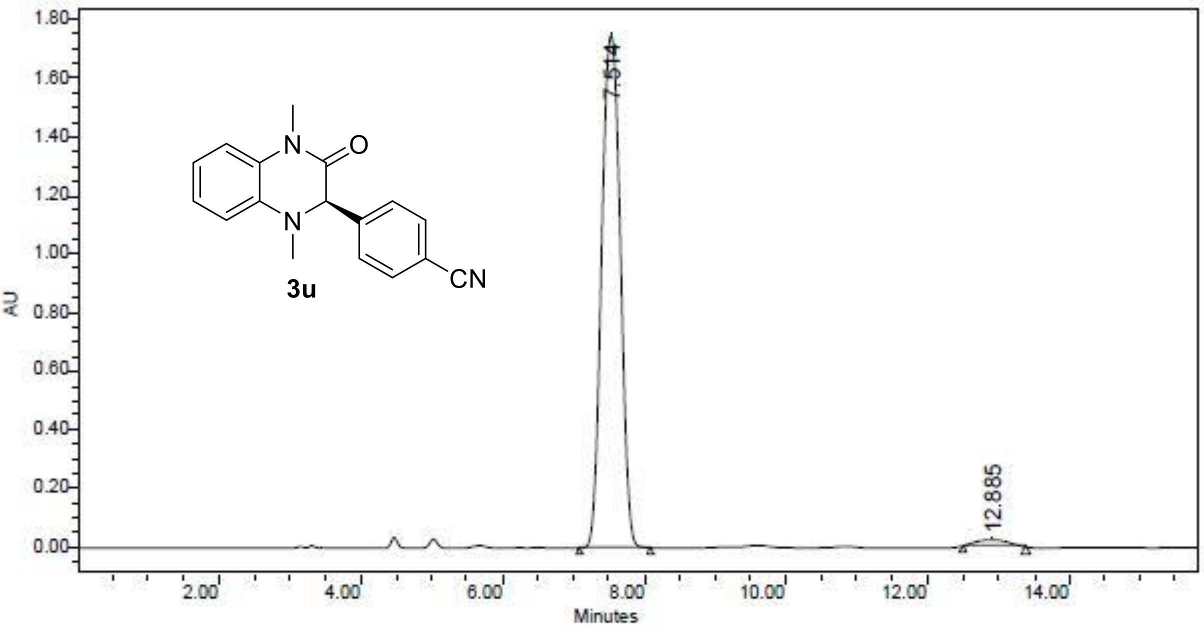

	RT (min)	Area (µV*sec)	% Area	Height (µV)	% Height
1	7.514	31962091	97.85	1749366	98.78
2	12.885	702389	2.15	21625	1.22

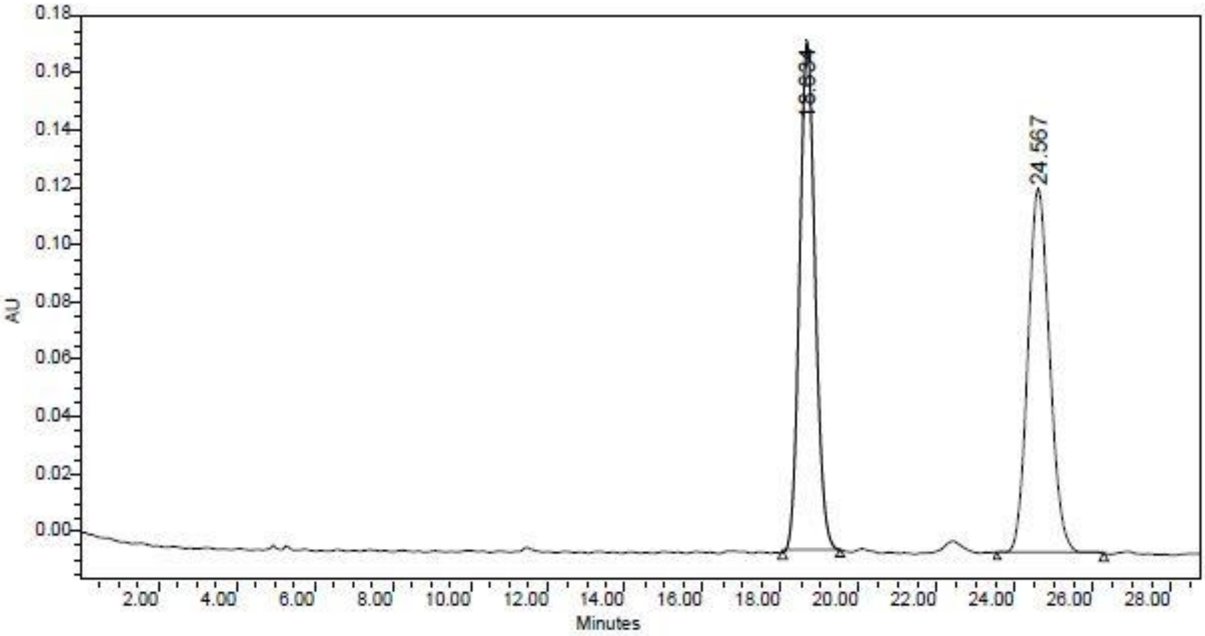

	RT (min)	Area (µV*sec)	% Area	Height (µV)	% Height
1	18.634	4939144	49.99	177655	58.34
2	24.567	4941430	50.01	126863	41.66

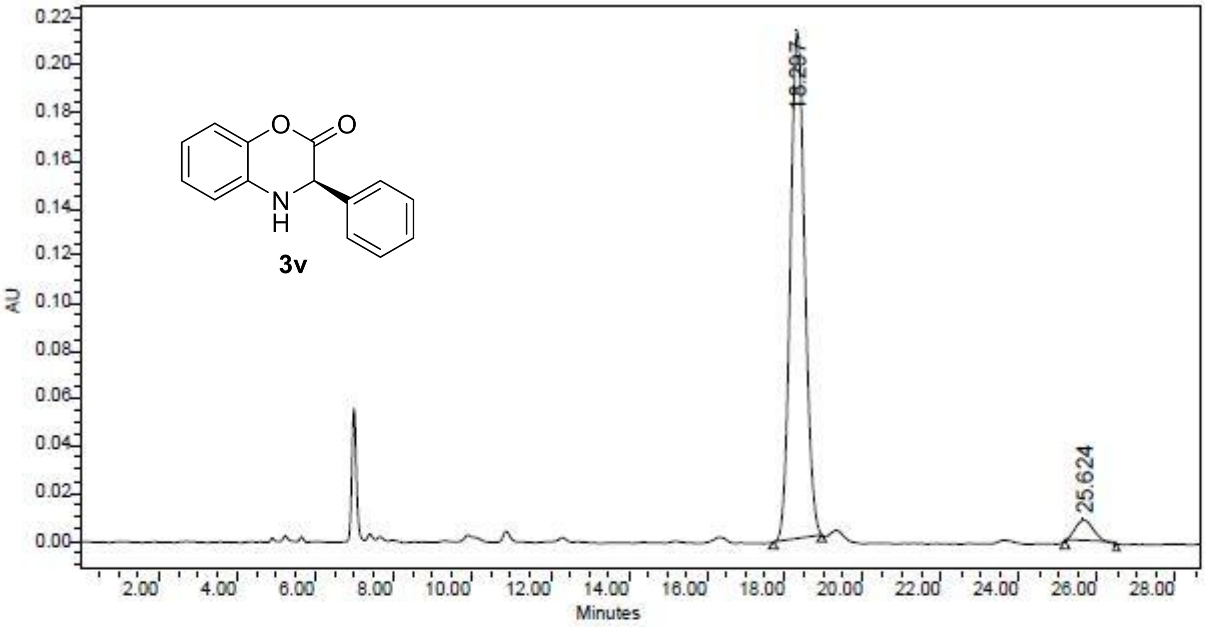

	RT (min)	Area (µV*sec)	% Area	Height (µV)	% Height
1	18.297	5474892	94.55	213403	95.79
2	25.624	315354	5.45	9371	4.21

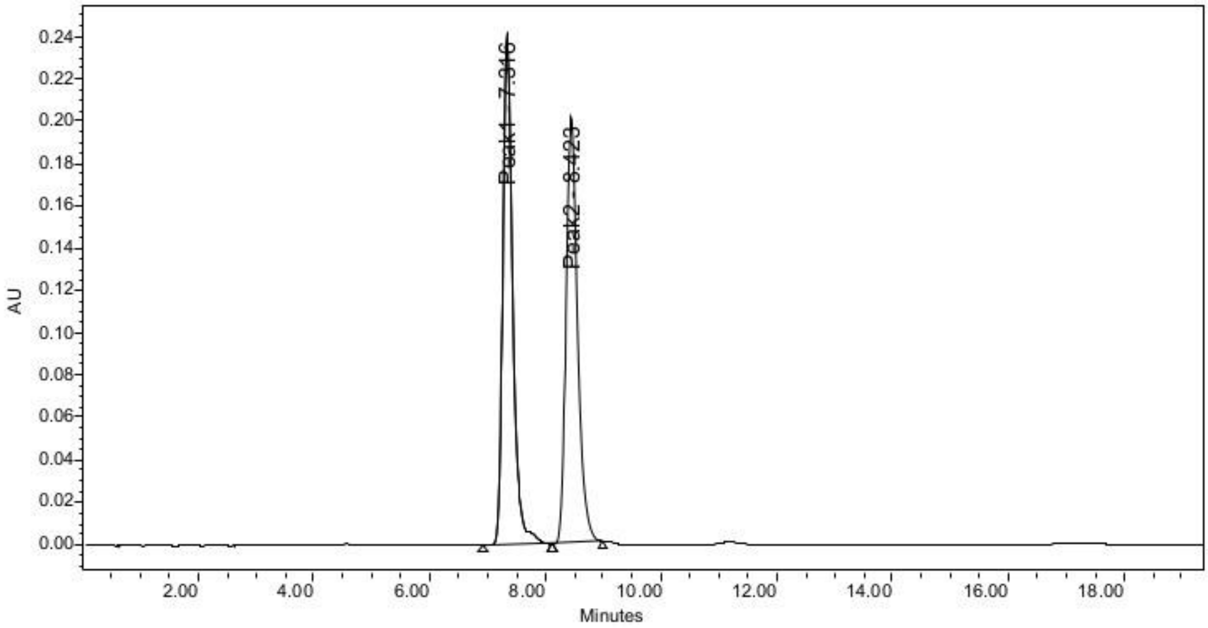

	Peak Name	RT (min)	Area (V*sec)	% Area	Height (V)	% Height
1	Peak1	7.316	2898272	50.92	241897	54.52
2	Peak2	8.423	2793286	49.08	201808	45.48

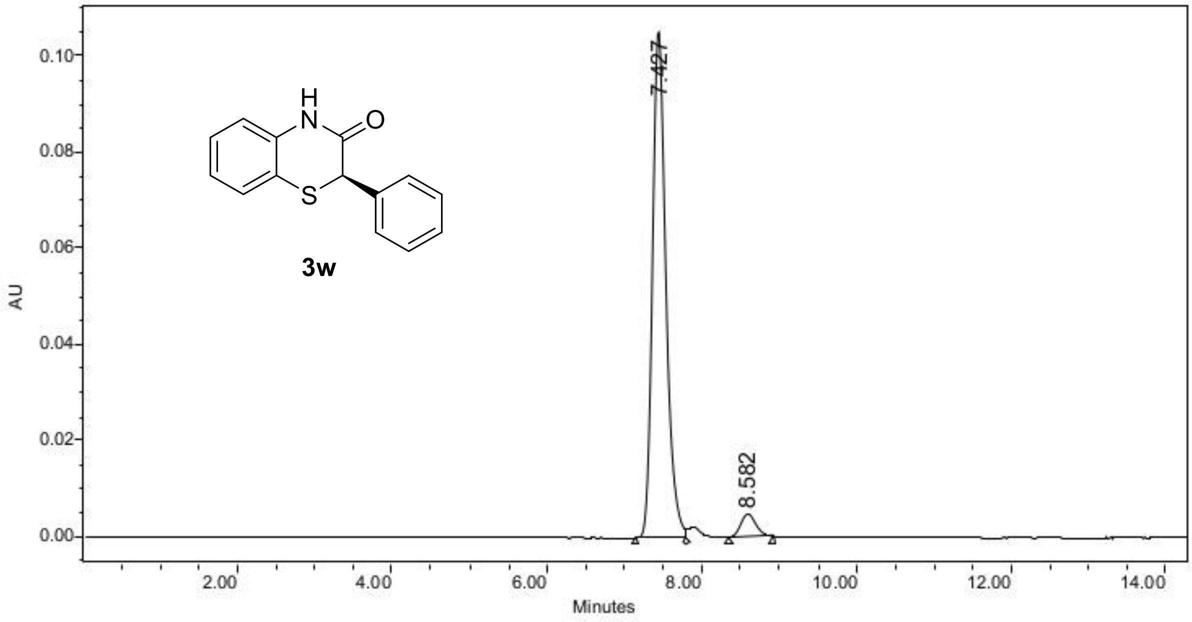

	RT (min)	Area (V*sec)	% Area	Height (V)	% Height
1	7.427	1267720	95.04	105589	95.61
2	8.582	66164	4.96	4845	4.39

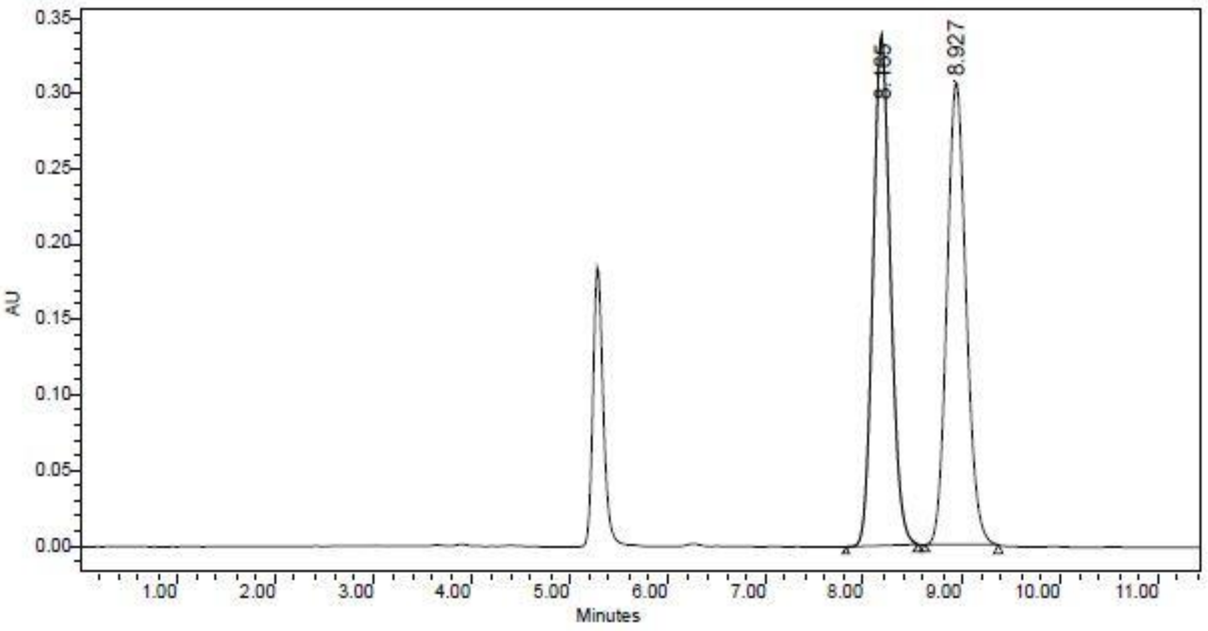

	RT (min)	Area (□V*sec)	% Area	Height (□V)	% Height
1	8.165	4179649	50.00	339135	52.42
2	8.927	4180358	50.00	307868	47.58

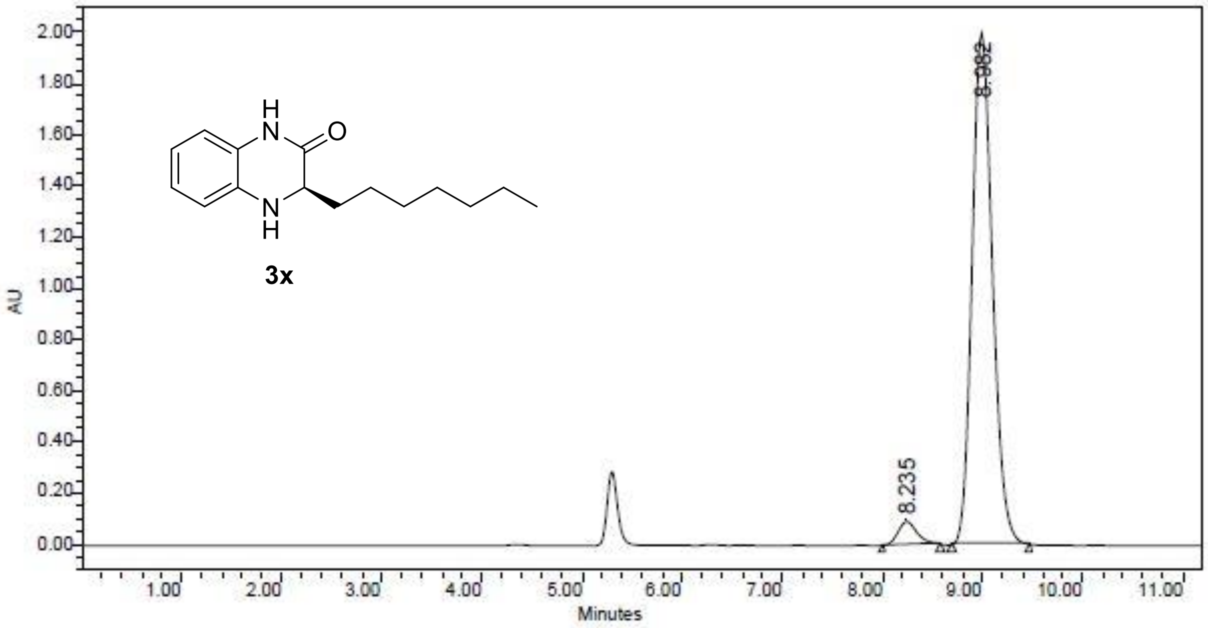

	RT (min)	Area (□V*sec)	% Area	Height (□V)	% Height
1	8.235	1229115	4.18	91447	4.38
2	8.982	28183246	95.82	1996000	95.62

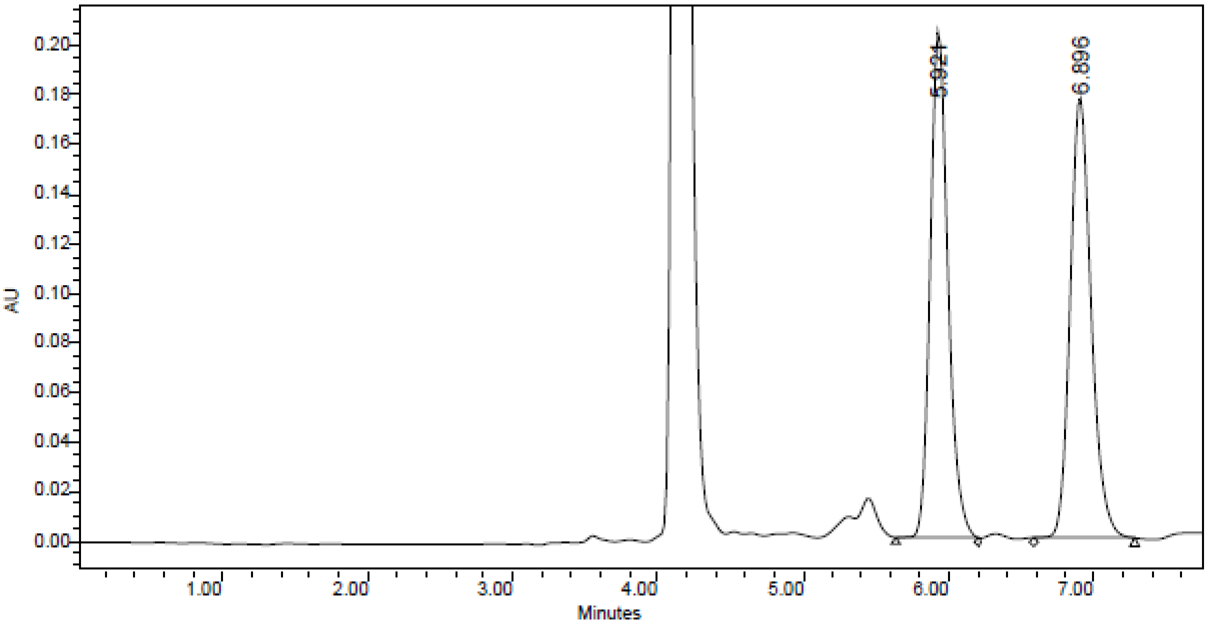

	RT (min)	Area (□V*sec)	% Area	Height (□V)	% Height
1	5.921	1747495	49.23	204504	53.49
2	6.896	1802140	50.77	177797	46.51

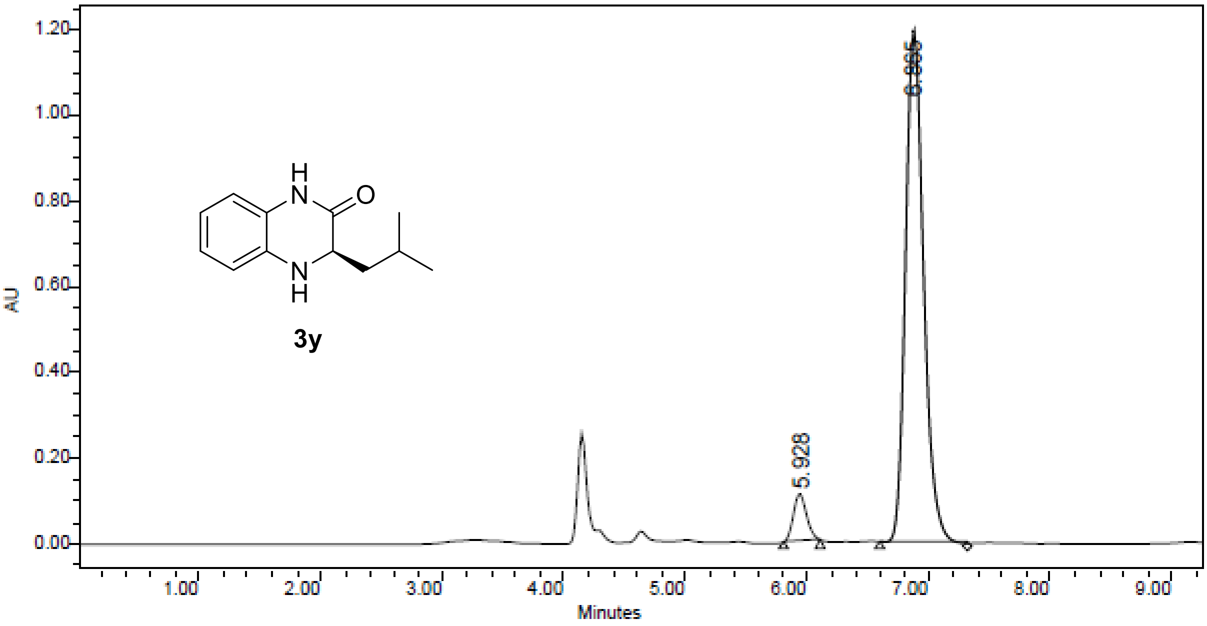

	RT (min)	Area (□V*sec)	% Area	Height (□V)	% Height
1	5.928	838379	6.49	110209	8.46
2	6.865	12070859	93.51	1191804	91.54

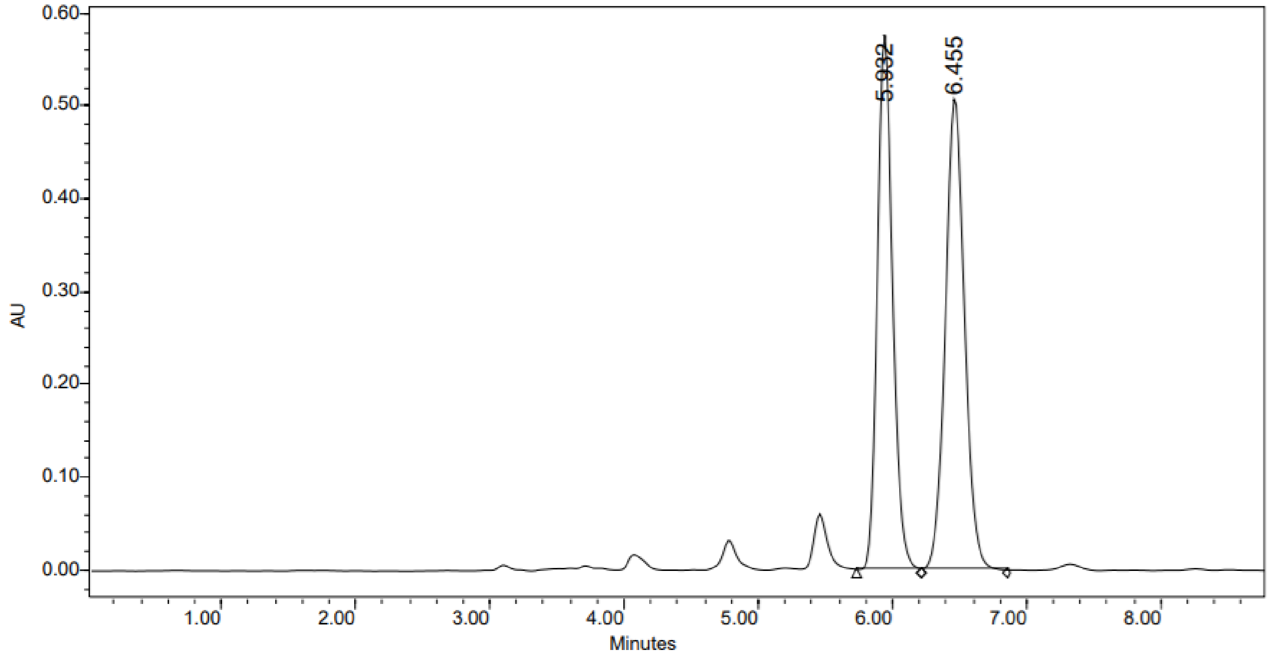

	RT (min)	Area (µV*sec)	% Area	Height (µV)	% Height
1	5.932	4566663	47.44	574842	53.05
2	6.455	5059211	52.56	508807	46.95

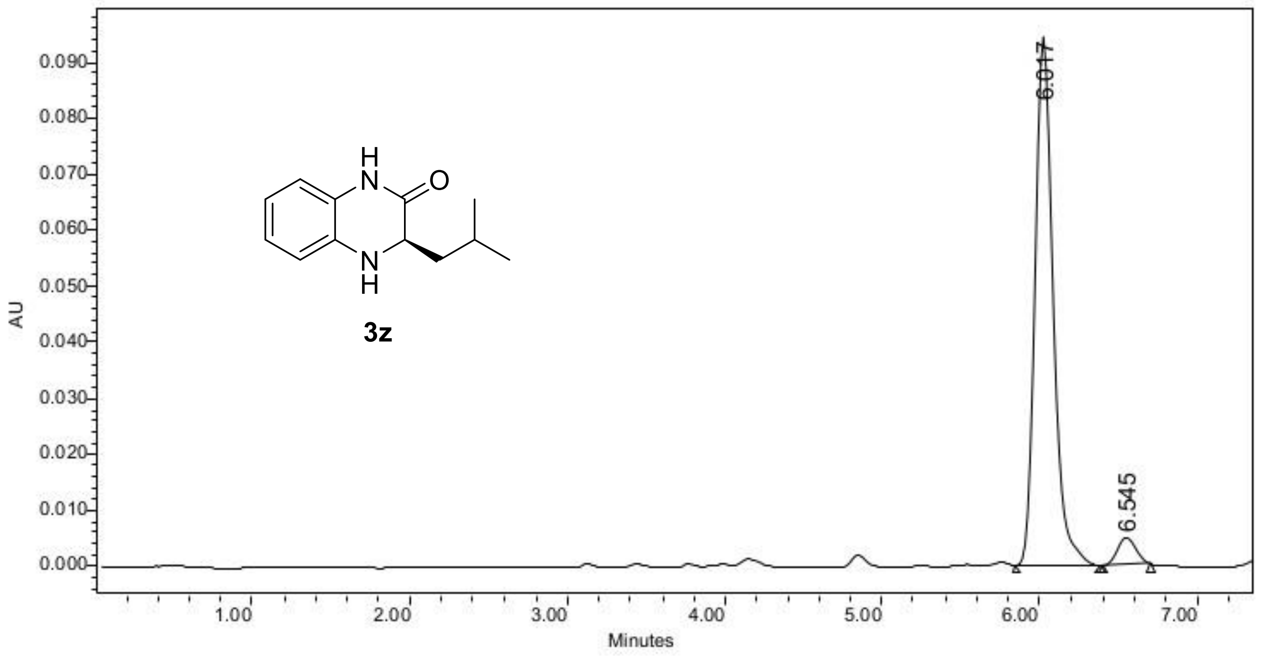

	RT (min)	Area (V*sec)	% Area	Height (V)	% Height
1	6.017	753539	94.94	94169	95.09
2	6.545	40166	5.06	4865	4.91

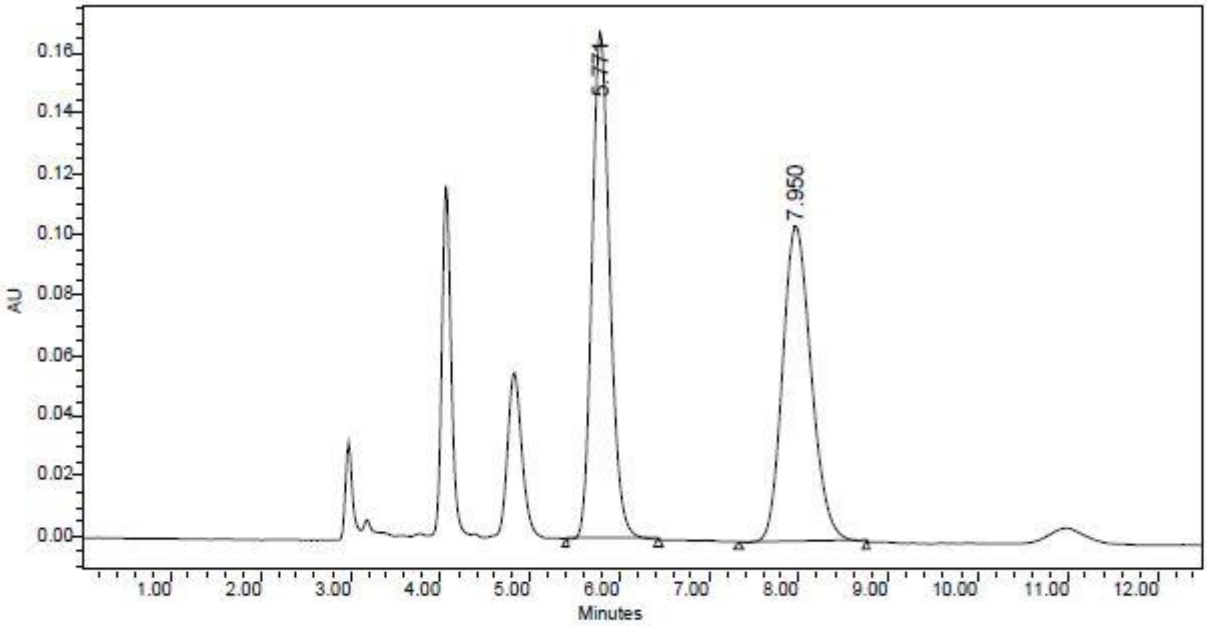

	RT (min)	Area (µV*sec)	% Area	Height (µV)	% Height
1	5.771	2324025	50.01	167945	61.64
2	7.950	2322904	49.99	104531	38.36

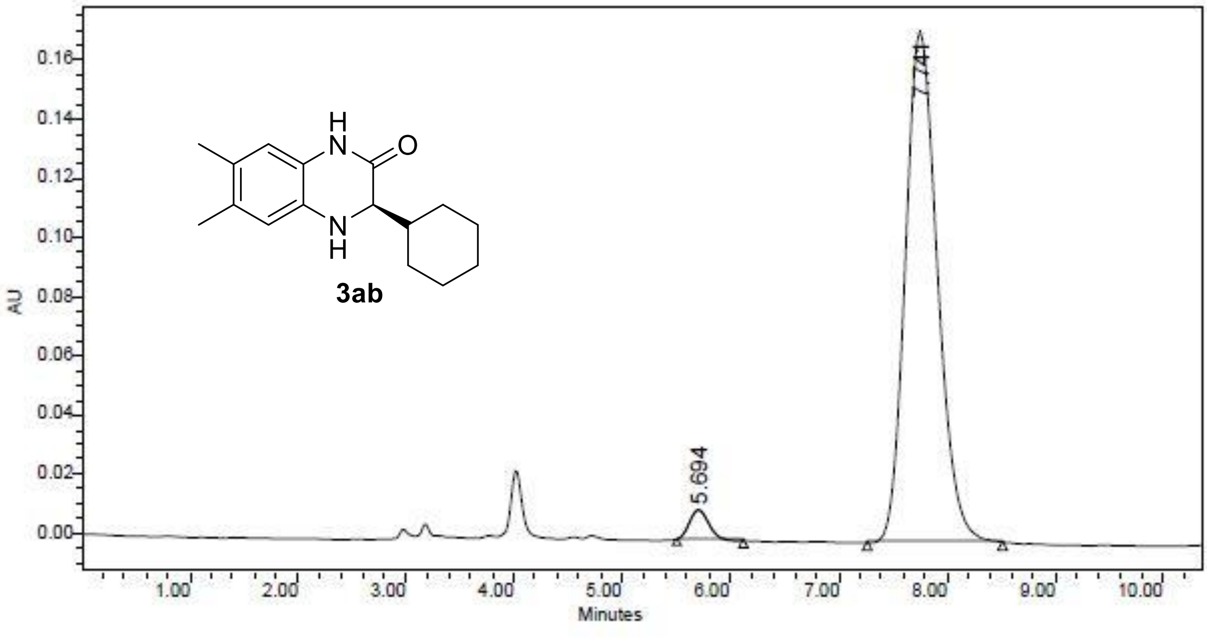

	RT (min)	Area (µV*sec)	% Area	Height (µV)	% Height
1	5.694	123051	3.34	10035	5.50
2	7.741	3565088	96.66	172468	94.50

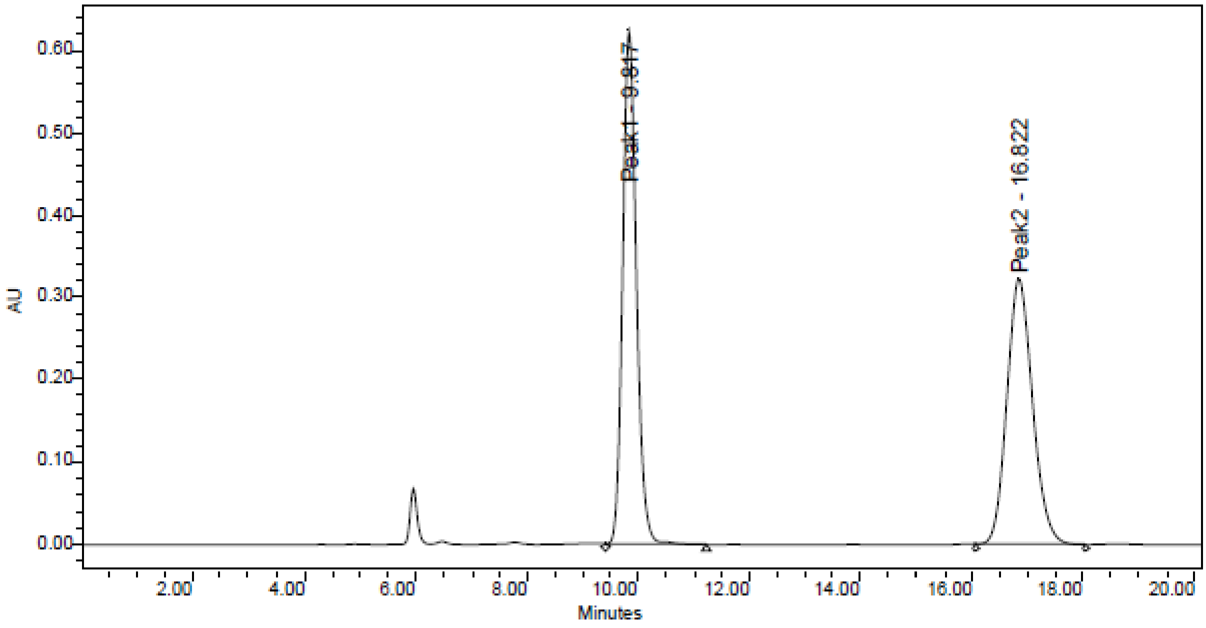

	Peak Name	RT (min)	Area (µV*sec)	% Area	Height (µV)	% Height
1	Peak1	9.817	10717871	50.60	623672	65.79
2	Peak2	16.822	10463179	49.40	324239	34.21

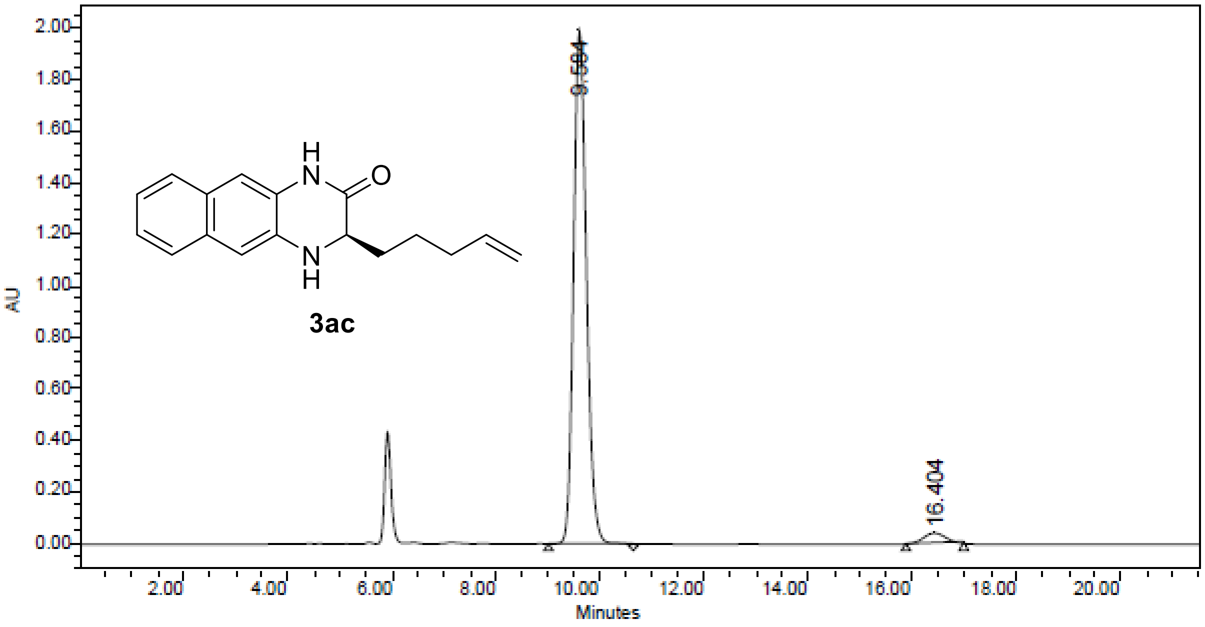

	RT (min)	Area (µV*sec)	% Area	Height (µV)	% Height
1	9.584	34270899	96.49	1987710	97.95
2	16.404	1245408	3.51	41591	2.05

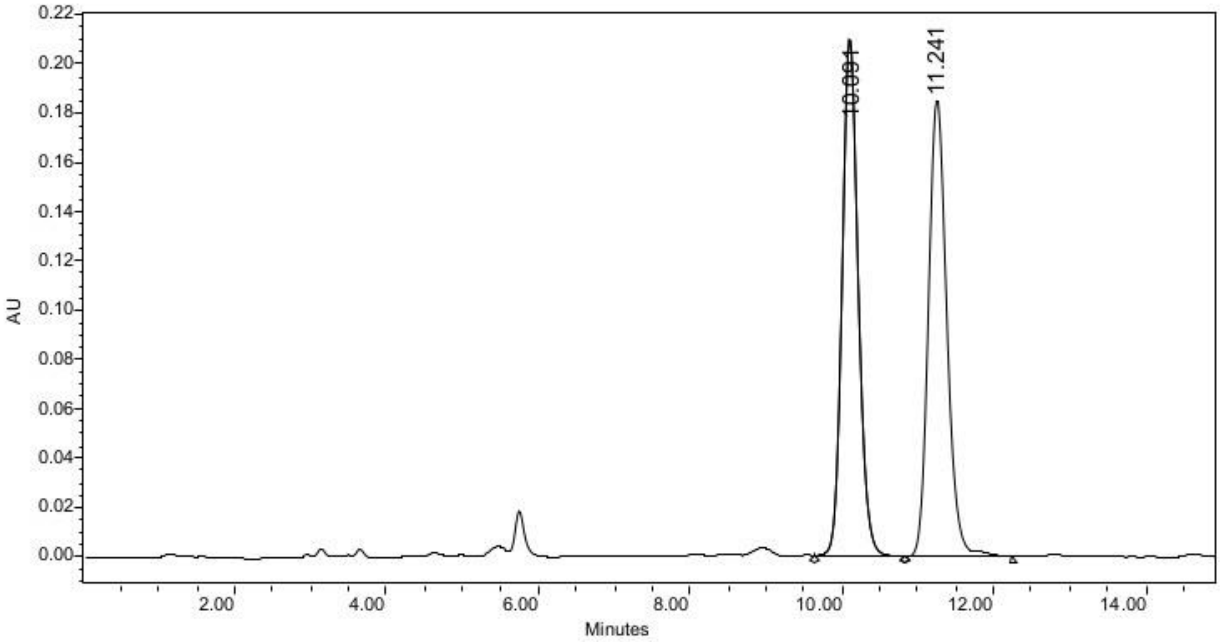

	RT (min)	Area (V*sec)	% Area	Height (V)	% Height
1	10.091	3102609	49.89	210448	53.14
2	11.241	3116440	50.11	185553	46.86

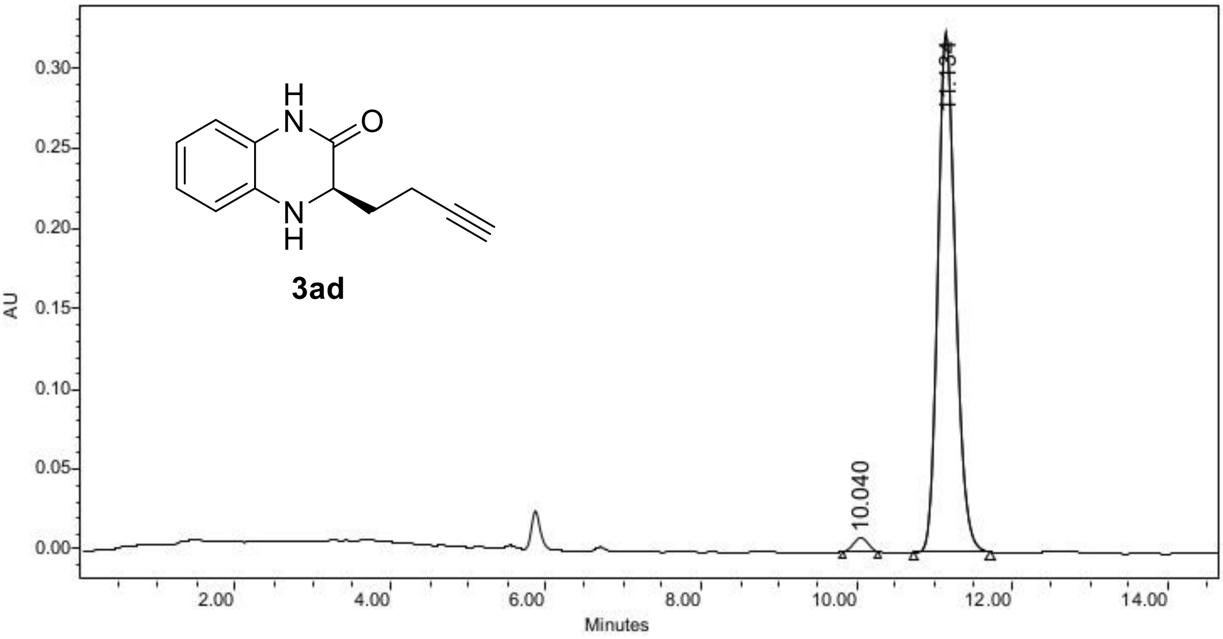

	RT (min)	Area (V*sec)	% Area	Height (V)	% Height
1	10.040	112098	2.14	8770	2.64
2	11.134	5125403	97.86	323815	97.36

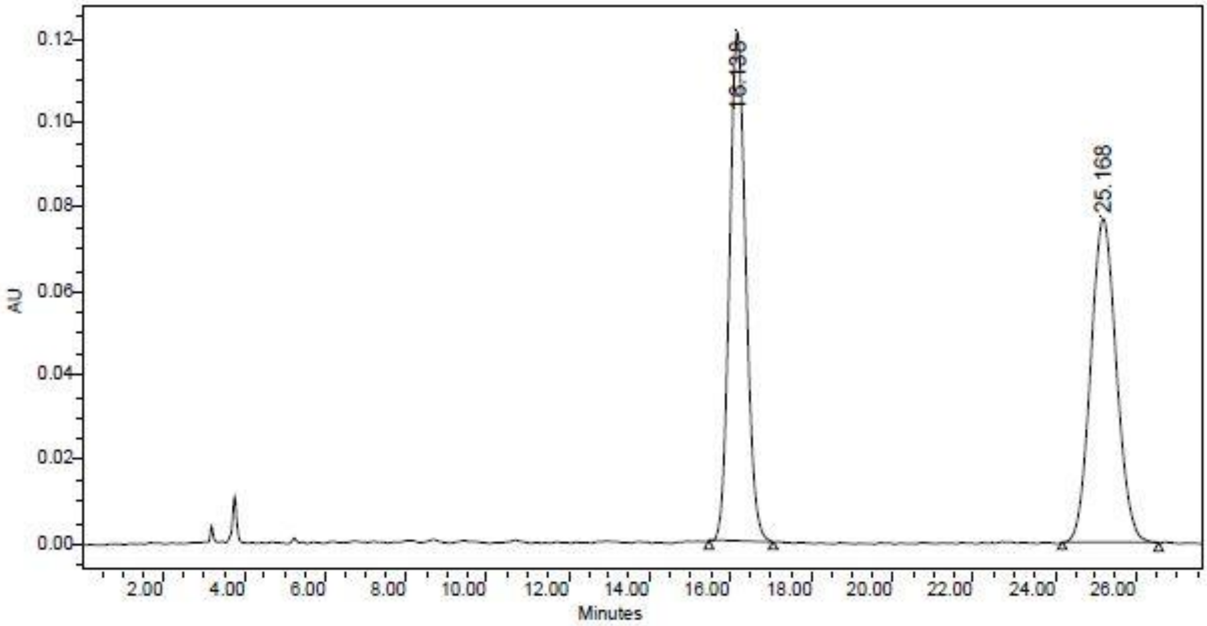

	RT (min)	Area (µV*sec)	% Area	Height (µV)	% Height
1	16.138	3394770	50.00	121119	61.12
2	25.168	3394386	50.00	77037	38.88

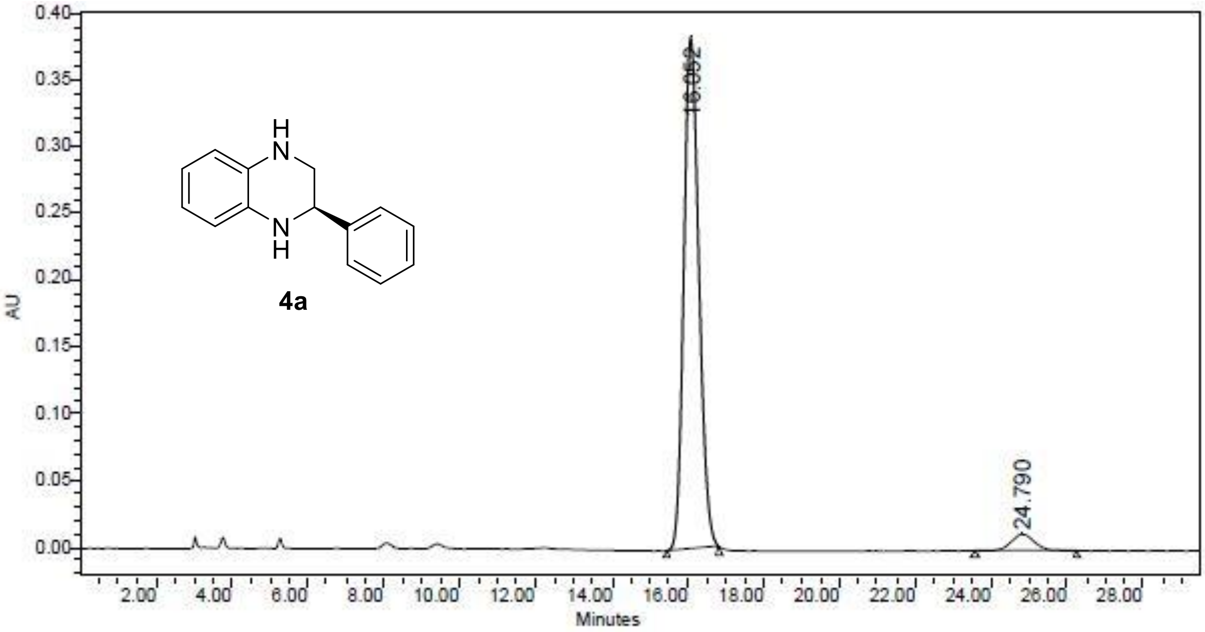

	RT (min)	Area (µV*sec)	% Area	Height (µV)	% Height
1	16.052	10568145	95.15	382702	96.86
2	24.790	538189	4.85	12389	3.14

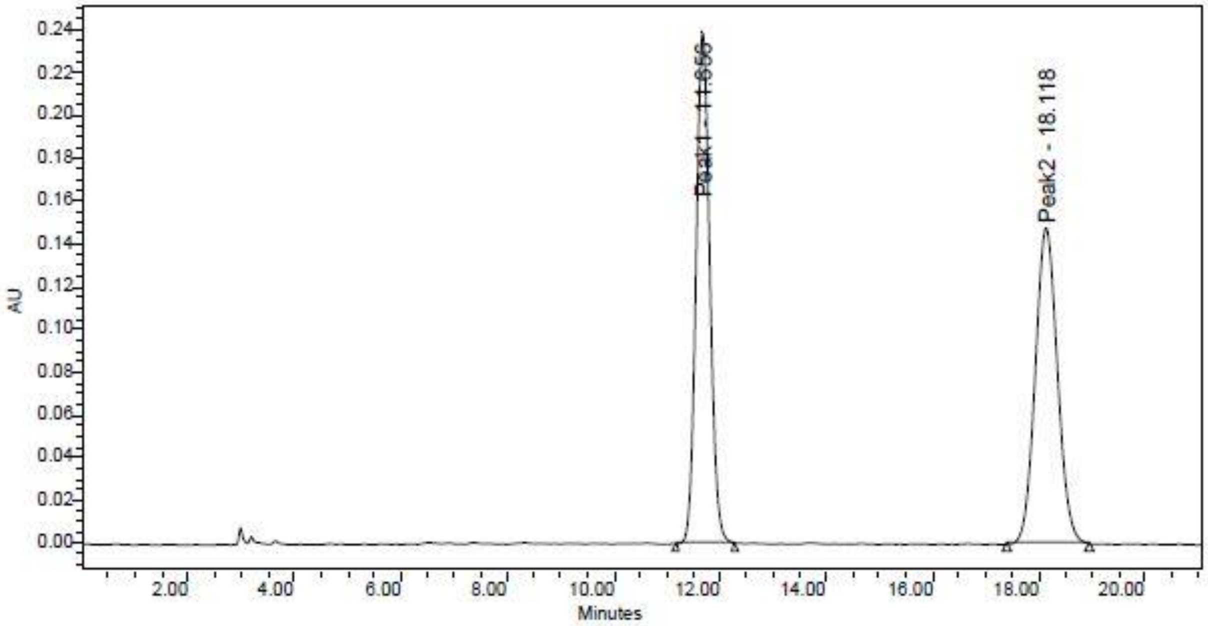

	Peak Name	RT (min)	Area (□V*sec)	% Area	Height (□V)	% Height
1	Peak1	11.656	4289897	49.98	239322	61.82
2	Peak2	18.118	4293616	50.02	147809	38.18

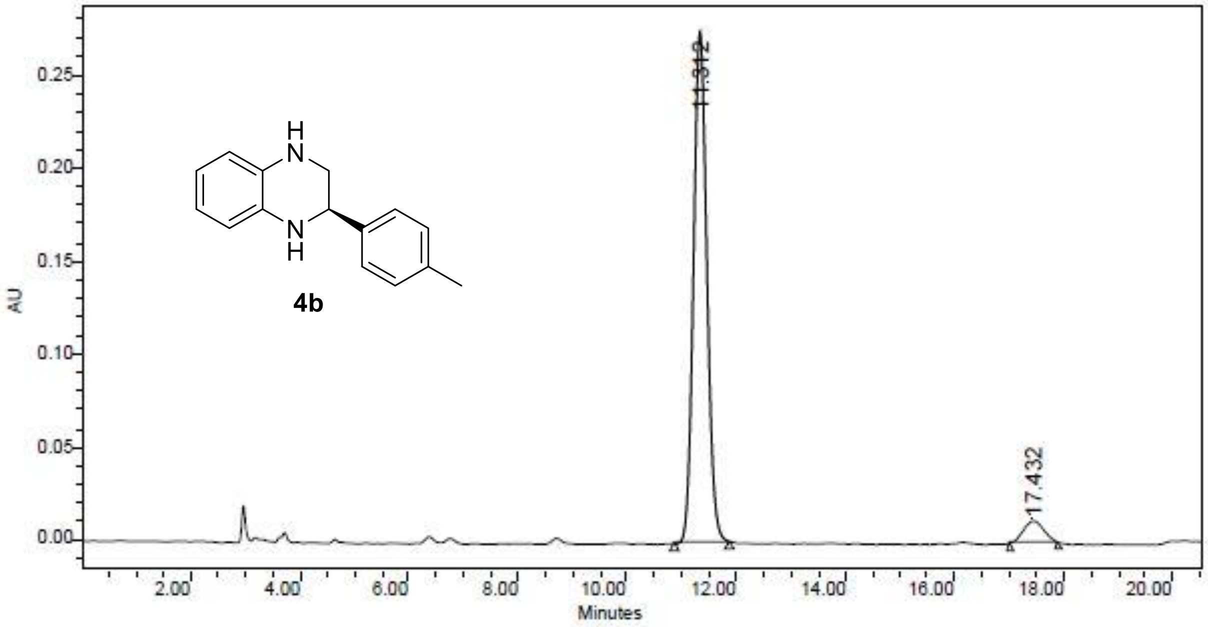

	RT (min)	Area (□V*sec)	% Area	Height (□V)	% Height
1	11.312	4681382	93.91	274932	95.92
2	17.432	303751	6.09	11703	4.08

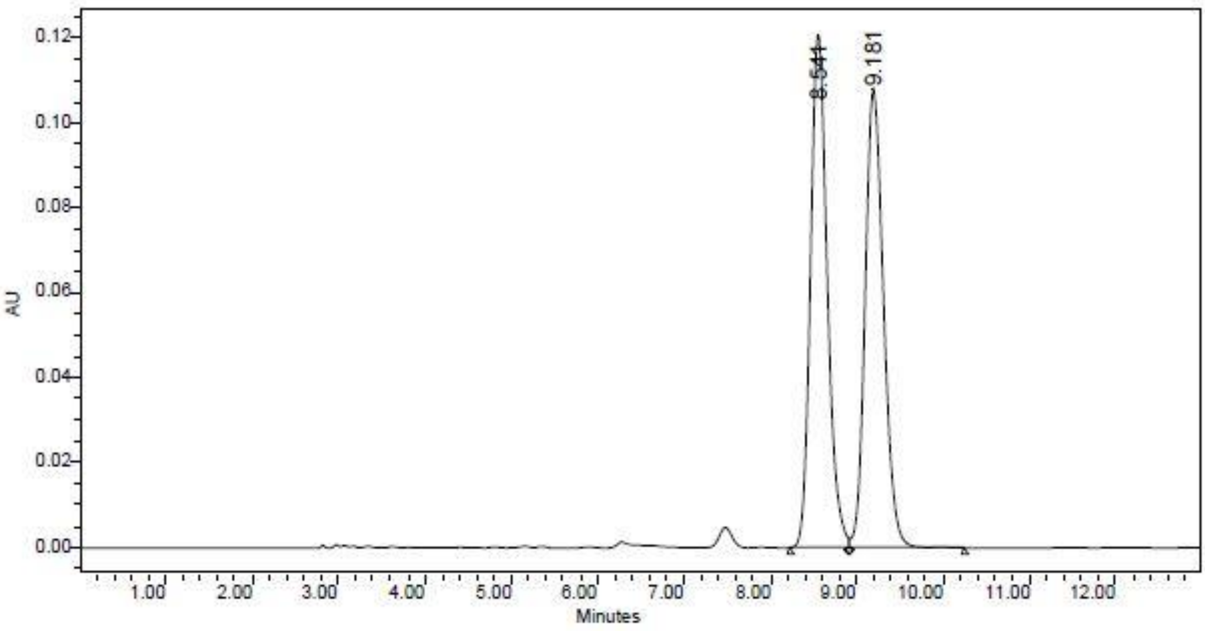

	RT (min)	Area (µV*sec)	% Area	Height (µV)	% Height
1	8.541	1637751	50.59	121374	52.91
2	9.181	1599750	49.41	108038	47.09

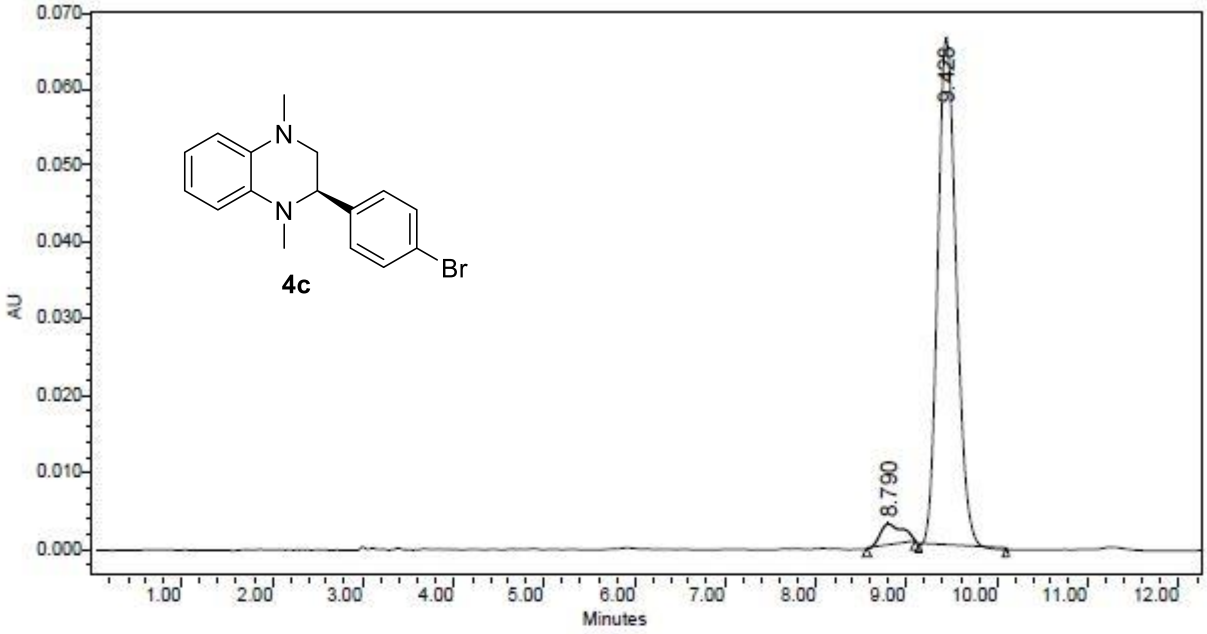

	RT (min)	Area (µV*sec)	% Area	Height (µV)	% Height
1	8.790	50915	5.12	2875	4.16
2	9.428	943576	94.88	66312	95.84

DFT calculations

Ground state optimizations and transition states were obtained by DFT calculations performed by the Gaussian16 software suite,[14] using standard parameters. Full optimization and frequency analysis for ground states and transition states employed the B3LYP and the 6-31G(d) basis set. The IEFPCM approach was used to account for the solvent contribution (toluene). The analysis of the vibrational frequencies showed the absence of imaginary frequencies for the ground states, and the presence of one imaginary frequency for each transition state. Visual inspection of the corresponding normal mode validated the identification of the transition states. The frequencies were scaled by 0.977[15] and the RRHO approximation[16] was used to moderate the effect of the low-energy vibrators on the evaluation of the entropic correction. Single point energies were calculated at the B3LYP-GD3(BJ)/6-311+G(2d,p) level.[17] The thermal corrections obtained with B3LYP at the lower level were used to derive the Gibbs free energies reported in Table S4 ("Corr. G°" column).

[14] Gaussian 16, Revision A.03, Frisch, M. J.; Trucks, G. W.; Schlegel, H. B.; Scuseria, G. E.; Robb, M. A.; Cheeseman, J. R.; Scalmani, G.; Barone, V.; Petersson, G. A.; Nakatsuji, H.; Li, X.; Caricato, M.; Marenich, A. V.; Bloino, J.; Janesko, B. G.; Gomperts, R.; Mennucci, B.; Hratchian, H. P.; Ortiz, J. V.; Izmaylov, A. F.; Sonnenberg, J. L.; Williams-Young, D.; Ding, F.; Lipparini, F.; Egidi, F.; Goings, J.; Peng, B.; Petrone, A.; Henderson, T.; Ranasinghe, D.; Zakrzewski, V. G.; Gao, J.; Rega, N.; Zheng, G.; Liang, W.; Hada, M.; Ehara, M.; Toyota, K.; Fukuda, R.; Hasegawa, J.; Ishida, M.; Nakajima, T.; Honda, Y.; Kitao, O.; Nakai, H.; Vreven, T.; Throssell, K.; Montgomery Jr., J. A.; Peralta, J. E.; Ogliaro, F.; Bearpark, M. J.; Heyd, J. J.; Brothers, E. N.; Kudin, K. N.; Staroverov, V. N.; Keith, T. A.; Kobayashi, R.; Normand, J.; Raghavachari, K.; Rendell, A. P.; Burant, J. C.; Iyengar, S. S.; Tomasi, J.; Cossi, M.; Millam, J. M.; Klene, M.; Adamo, C.; Cammi, R.; Ochterski, J. W.; Martin, R. L.; Morokuma, K.; Farkas, O.; Foresman, J. B.; Fox, D. J. Gaussian, Inc., Wallingford CT, **2016**.

[15] Alecu, I. M.; Zheng, J.; Zhao, Y.; Truhlar, D. G. *J. Chem. Theory Comput*. **2010**, *6*, 2872.

[16] Grimme, S. *Chem. Eur. J.* **2012**, *18*, 9955.

[17] (a) Grimme, S.; Antony, J.; Ehrlich, S.; Krieg, H. *J. Chem. Phys.* **2010**, *132*, 154104; (b) Grimme, S.; Ehrlich, S.; Goerigk, L. *J. Comp. Chem*. **2011**, *32*, 1456.

Table S4. Summary of DFT calculated Energies

	EE	H	G°	H PCM-B3LYP 6-31G(d)	G° PCM-B3LYP 6-31G(d)	RRHO-corr. G°	**img.freq**	SP PCM B3LYP-GD3 6-311+G(2d,p)	Corr. G°	rel. ΔG°
TBHP	-308.804707	0.148364	0.108597	-308.656344	-308.696110	-308.699400		-308.935359	-308.830052	
catalyst	-2013.070408	0.543373	0.437577	-2012.527035	-2012.632831	-1181.341125		-2013.846193	-1182.11691	
Sulfone	-1181.517288	0.241703	0.178127	-1181.275585	-1181.339161	-2012.635238		-1181.870601	-2012.988551	
sum of reagents	-3503.392403	0.93344	0.724301	-3502.458964	-3502.668102	-3502.675763		-3504.652153	-3503.935513	**0**
**SS-Pathway**										
I-Si	-3194.605249	0.7876	0.637849	-3193.817649	-3193.9674	-3193.967305		-3195.741933	-3195.103989	-1.9
TS-Si	-3503.401956	0.937307	0.775307	-3502.464649	-3502.626649	-3502.630586	-197	-3504.693491	-3503.922121	8.4
II-S	-3503.425138	0.941857	0.776795	-3502.483281	-3502.648343	-3502.650431		-3504.711585	-3503.936878	-0.9
TS-SS	-3503.396412	0.937647	0.771976	-3502.458765	-3502.624436	-3502.626708	-204	-3504.683	-3503.913296	13.9
TS-Srot	-3503.401858	0.940275	0.779316	-3502.461583	-3502.622542	-3502.626236	-16	-3504.68604	-3503.910418	15.7
II-S'	-3503.417969	0.940247	0.781122	-3502.477722	-3502.636847	-3502.641447		-3504.706415	-3503.929893	3.5
TS-SR	-3503.395169	0.937966	0.774792	-3502.457203	-3502.620377	-3502.623673	-254	-3504.681961	-3503.910465	15.7
Epoxide SS	-3503.475308	0.938591	0.768401	-3502.536717	-3502.706907	-3502.707168		-3504.755851	-3503.987711	-32.8
Epoxide SR	-3503.466308	0.938393	0.76782	-3502.527915	-3502.698488	-3502.698526		-3504.74608	-3503.978298	-26.8
**RR-Pathway**										
I-Re	-3194.602948	0.787369	0.63523	-3193.815579	-3193.967718	-3193.966477		-3195.742412	-3195.105941	-3.4
TS-Re	-3503.402038	0.93787	0.775315	-3502.464168	-3502.626723	-3502.630225	-135	-3504.692519	-3503.920706	9.3
II-R	-3503.414264	0.941287	0.776336	-3502.472977	-3502.637928	-3502.640212		-3504.699439	-3503.925387	6.4
TS-RR	-3503.397771	0.937564	0.773639	-3502.460207	-3502.624161	-3502.626894	-296	-3504.682222	-3503.911345	15.2
TS-Rrot	-3503.400187	0.940001	0.774285	-3502.460186	-3502.625902	-3502.627267	-17	-3504.683508	-3503.910588	15.6
II-R'	-3503.419561	0.941459	0.778775	-3502.478102	-3502.640786	-3502.644352		-3504.709534	-3503.934325	0.7
TS-RS	-3503.391335	0.938133	0.775512	-3502.453202	-3502.615823	-3502.619629	-246	-3504.681902	-3503.910196	15.9
Epoxide RR	-3503.472037	0.938491	0.769328	-3502.533546	-3502.702709	-3502.703597		-3504.752757	-3503.984317	-30.6
Epoxide RS	-3503.469091	0.938159	0.767861	-3502.530932	-3502.70123	-3502.701329		-3504.748971	-3503.981209	-28.7

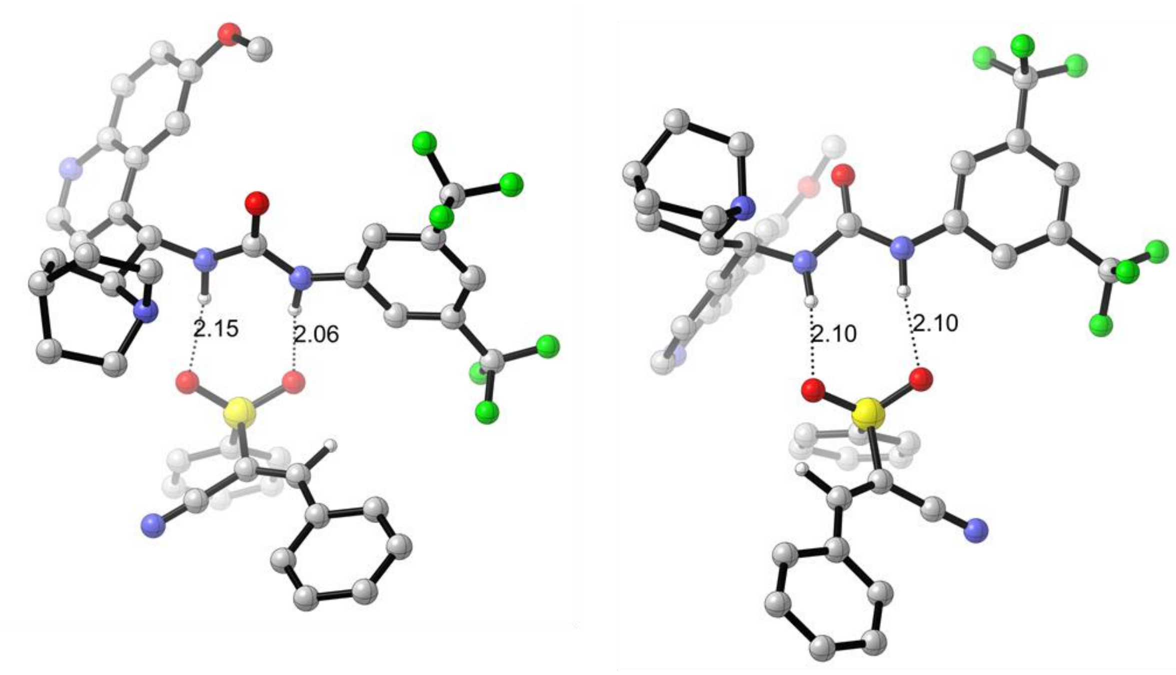

I-*Si* **I**-*Re*

Figure S2. Electrophilic complexes **I**-*Si* and **I**-*Re*. Most hydrogens were omitted for the sake of clarity. Distances in Å. Calculations at the IEFPCM B3LYP-GD3(BJ)/6-311+G(2d,p)//B3LYP/6-31G(d).

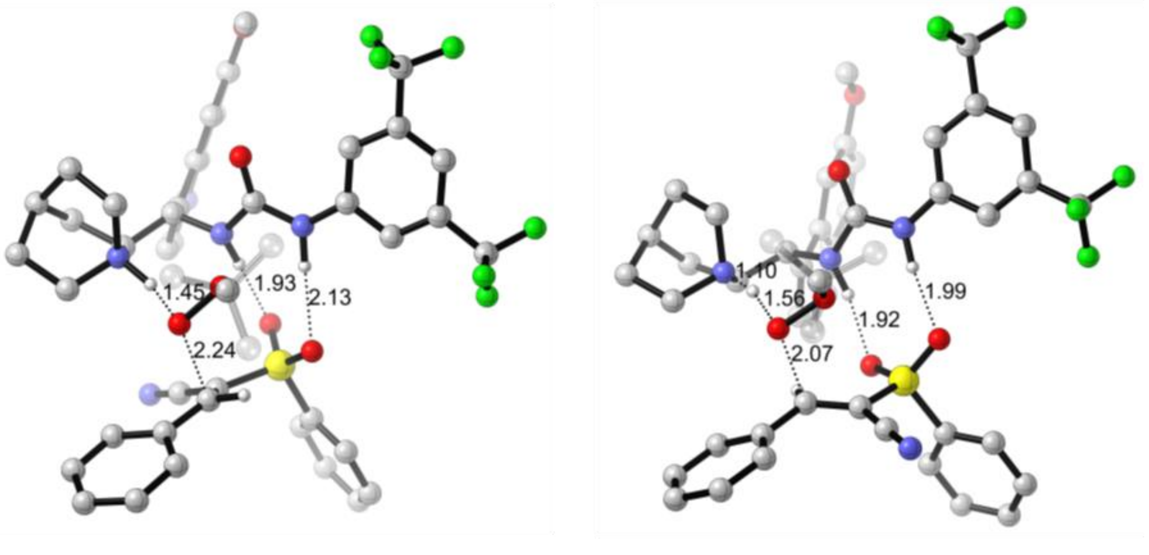

TS-*Si* **TS**-*Re*

Figure S3. TS geometries for the addition of TBHP to alkene. Distances in Å. Calculations at the IEFPCM B3LYP-GD3(BJ)/6-311+G(2d,p)//B3LYP/6-31G(d).

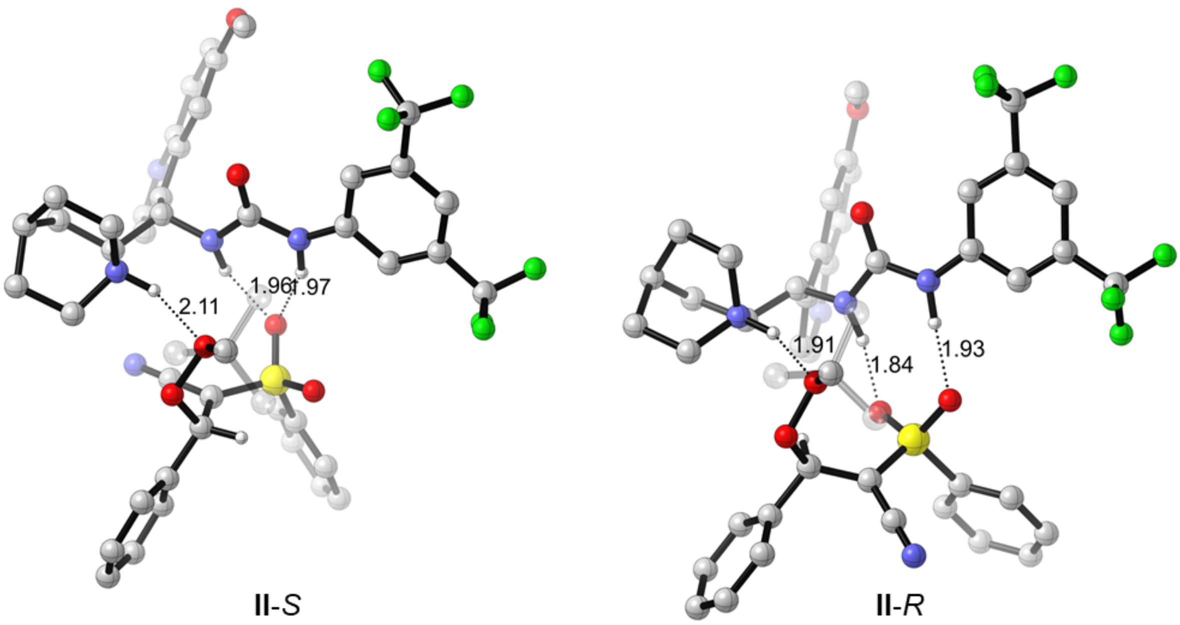

Figure S4. Reaction intermediates after the addition of TBHP. Most hydrogens were omitted for the sake of clarity, and the three methyls of *tert*-butyl moiety have been shaded. Distances in Å. Calculations at the IEFPCM B3LYP-GD3(BJ)/6-311+G(2d,p)//B3LYP/6-31G(d).

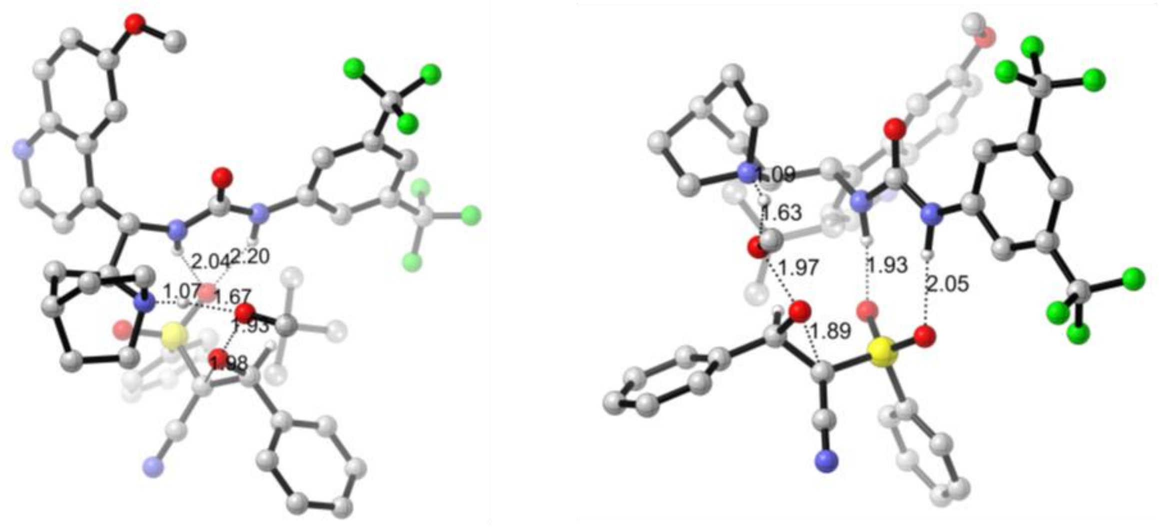

TS-*SS* **TS**-*RR*

Figure S5. TS structures for the ring closure to *S*,*S* and *R*,*R* stereoisomers of epoxide **2a**. Distances in Å. Calculations at the IEFPCM B3LYP-GD3(BJ)/6-311+G(2d,p)//B3LYP/6-31G(d).

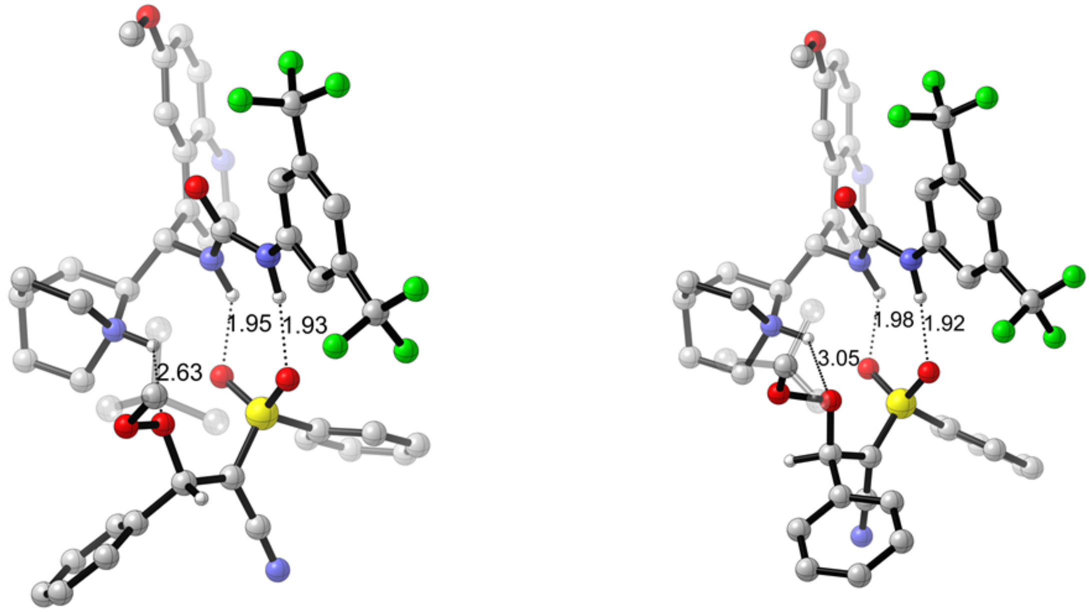

TS-S_{rot} **TS**-R_{rot}

Figure S6. TSs structures for the C_{α}-C_{β} bond rotation. Most hydrogens were omitted for the sake of clarity, and the three methyls of *tert*-butyl moiety have been shaded. Distances in Å. Calculations at the IEFPCM B3LYP-GD3(BJ)/6-311+G(2d,p)//B3LYP/6-31G(d).

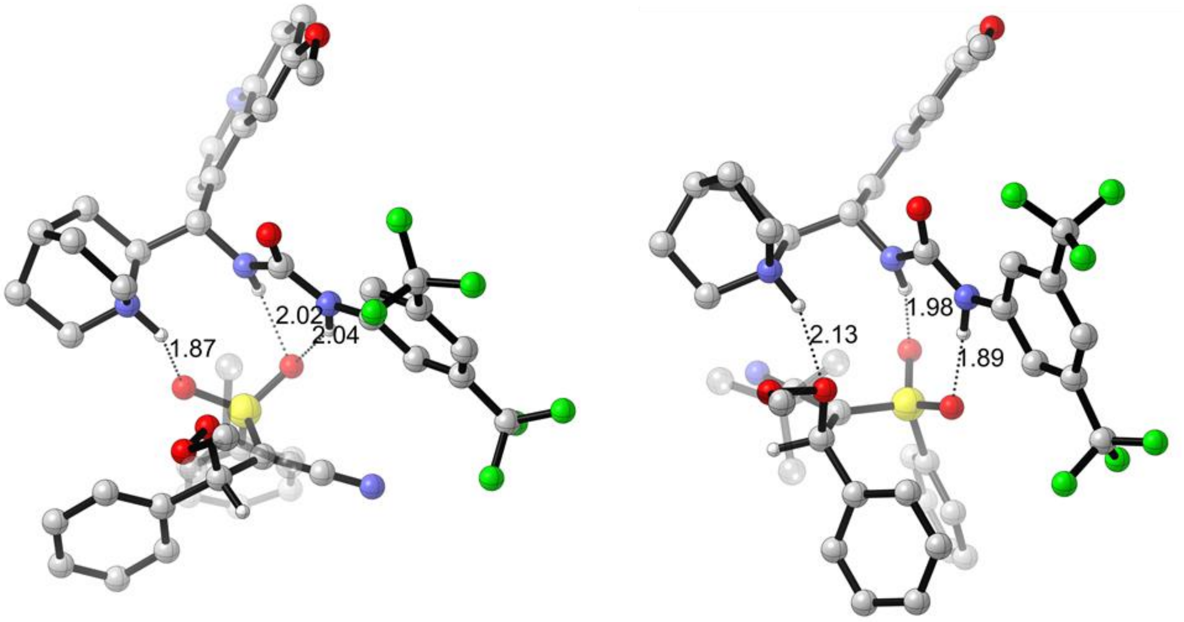

II-*S'* **II**-*R'*

Figure S7. Reaction intermediates after the addition of TBHP and C_{α}-C_{β} bond rotation. Most hydrogens were omitted for the sake of clarity, and the three methyls of *tert*-butyl moiety have been

shaded. Distances in Å. Calculations at the IEFPCM B3LYP-GD3(BJ)/6-311+G(2d,p)//B3LYP/6-31G(d).

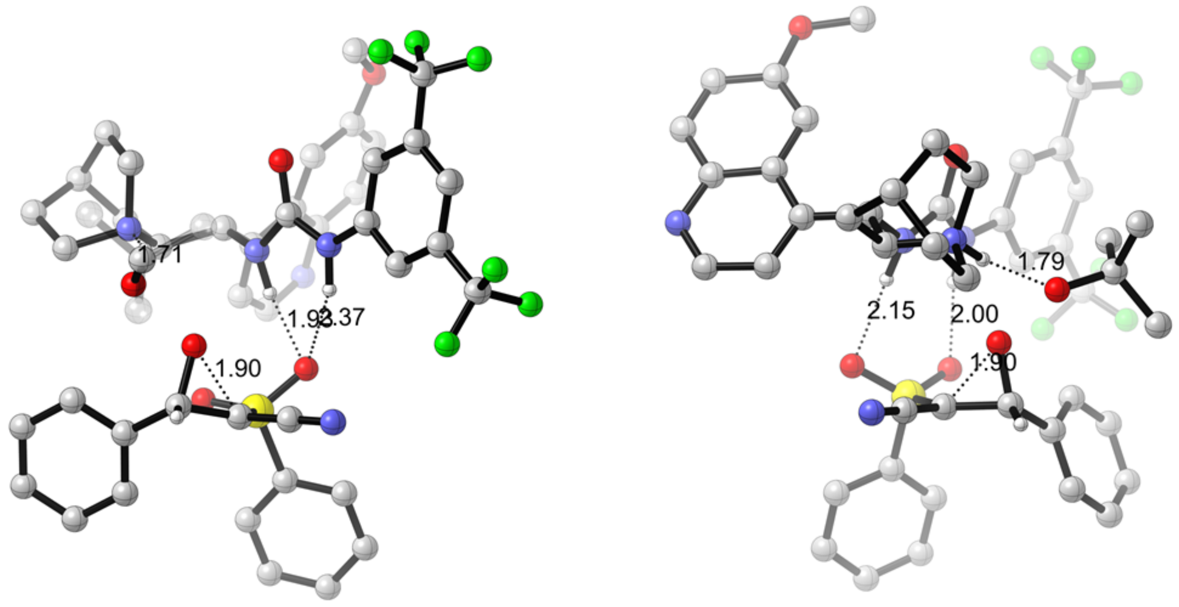

TS-*SR*   **TS**-*RS*

Figure S8. TSs structures for ring closure to *R*, *S* and *S*, *R* stereoisomers. Most hydrogens were omitted for the sake of clarity, and the three methyls of *tert*-butyl moiety have been shaded. Distances in Å. Calculations at the IEFPCM B3LYP-GD3(BJ)/6-311+G(2d,p)//B3LYP/6-31G(d).

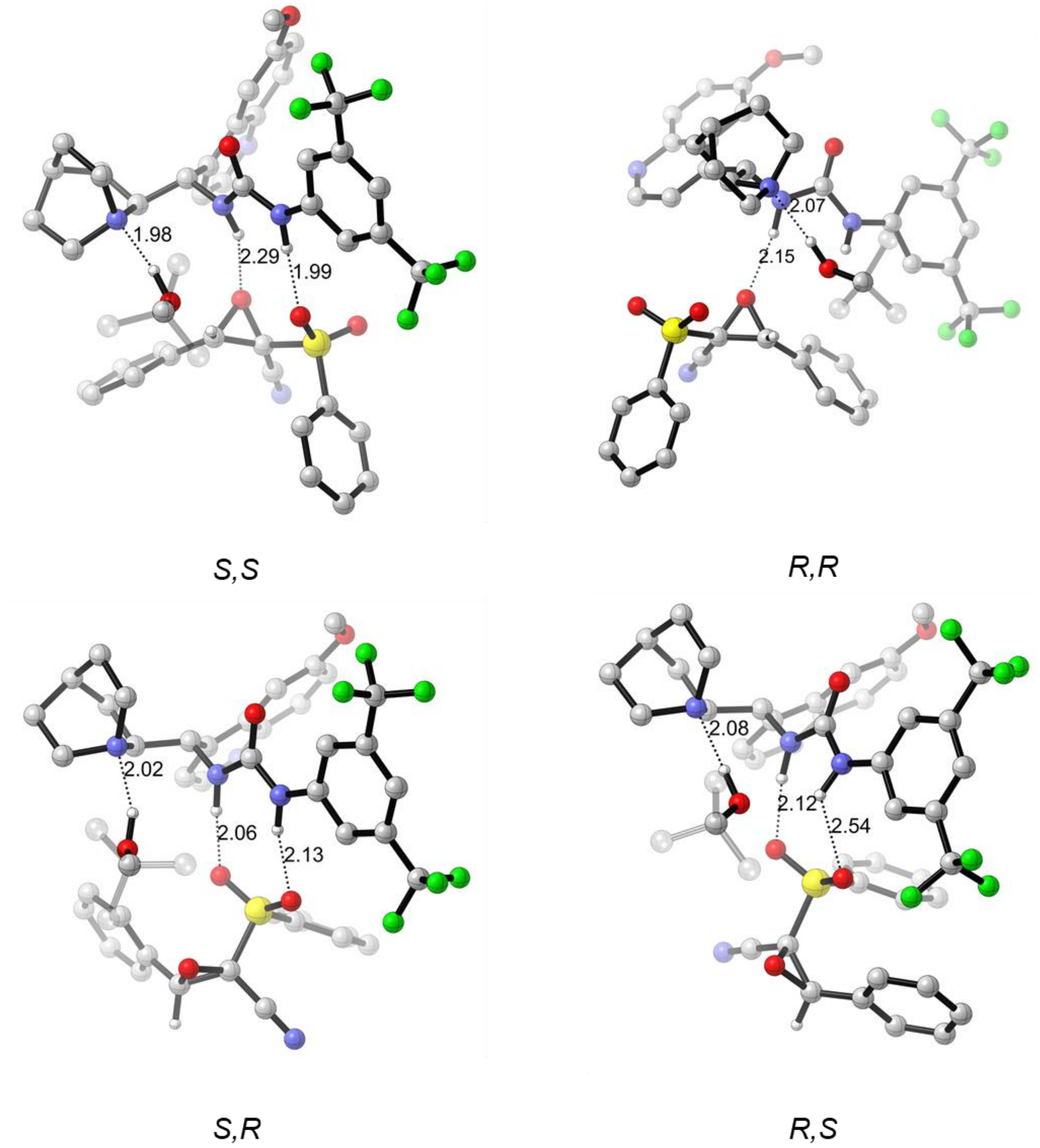

Figure S9. GSs structures for the four stereoisomers of **2a**, complexed with the catalyst. Most hydrogens were omitted for the sake of clarity, and the three methyls of *tert*-butyl moiety have been shaded. Distances in Å. Calculations at the IEFPCM B3LYP-GD3(BJ)/6-311+G(2d,p)//B3LYP/6-31G(d).

XYZ-coordinates.

TBHP

0 1

O	0.72816500	-0.03800500	-0.89286900
C	-0.38788600	-0.00009400	0.03635100
C	-0.30485000	-1.17814600	1.01297200
H	-1.18445200	-1.19985600	1.66530500
H	-0.26023200	-2.12766600	0.46734600
H	0.58319900	-1.09401300	1.64650400
C	-0.41775700	1.34540500	0.76782400
H	0.47897700	1.47048600	1.38063500
H	-0.46365000	2.16924600	0.04783400
H	-1.29488100	1.40660500	1.42173700
C	-1.58350600	-0.14610900	-0.91241200
H	-2.51682500	-0.12105000	-0.34054200
H	-1.60100100	0.67169400	-1.63986900
H	-1.53608100	-1.09515000	-1.45645600
O	1.97773400	0.11241100	-0.15980700
H	2.31174300	-0.80187800	-0.19949800

Organocatalyst

0 1

N	1.26089600	0.45987000	-0.97077500
C	2.55806800	0.62967900	-0.31030600
C	3.01034000	2.11070800	-0.31353400
H	2.39322300	0.33336500	0.72644600
C	3.56634100	-0.34018400	-0.93086900
H	3.16470000	2.40938800	-1.35896100
C	4.33305500	2.33682500	0.48678400
N	1.93970900	3.00797600	0.17508800
C	3.53959100	-1.73077500	-0.57752300
C	4.49181700	0.05941000	-1.87413100
C	4.16138900	3.59868600	1.35702100
H	5.18553400	2.44927400	-0.19254800
H	4.55575100	1.47391300	1.12854400
C	1.76948700	2.91120700	1.64242300
C	2.32439800	4.39474000	-0.15500500
C	2.62537400	-2.28542900	0.36126900
C	4.48871200	-2.59328300	-1.21785300
C	5.37972800	-0.88010200	-2.44790200
H	4.56470600	1.09403600	-2.19124800
C	3.06684500	3.32102600	2.40466500
H	5.10897800	3.84127200	1.85088500
C	3.70830700	4.77078100	0.46292600
H	1.45585300	1.89445300	1.89018000

H	0.93258400	3.56583200	1.90930500
H	2.33727900	4.49789900	-1.24658100
H	1.53282200	5.05231000	0.22103300
C	2.65945600	-3.63709100	0.65265600
H	1.88417500	-1.65088100	0.82904000
C	4.49495900	-3.97709100	-0.88575100
N	5.39500400	-2.16101600	-2.14127500
H	6.10541800	-0.54610100	-3.18886500
H	3.39036700	2.52347700	3.08500500
H	2.89830600	4.21496500	3.01792200
H	3.63634900	5.69141500	1.05450500
C	3.60805200	-4.48985700	0.02337200
O	1.82646700	-4.26378900	1.52626400
H	5.22561200	-4.60879000	-1.38104900
H	3.60292000	-5.54398500	0.28286000
C	0.86860500	-3.47411100	2.22732000
H	0.31048600	-4.17164800	2.85353300
H	1.36056900	-2.72432000	2.85891400
H	0.18449400	-2.96475500	1.53910600
N	-1.04858800	0.40787700	-1.01725300
O	0.09671000	-0.05005600	0.93435800
H	1.19586100	0.83938900	-1.90666000
H	-0.93296900	0.66631000	-1.98818000
C	-2.37625000	0.20923200	-0.61711800
C	-2.75618300	-0.10629800	0.69673500
C	-3.37053700	0.33696100	-1.60087400
C	-4.10661200	-0.28868000	0.99609200
H	-1.99824500	-0.21359700	1.45830200
C	-4.71184400	0.15384400	-1.27832200
H	-3.09336800	0.56789700	-2.62509500
C	-5.09784300	-0.16300600	0.02444100
H	-6.14067500	-0.31997600	0.27042000
C	-5.76275300	0.35366200	-2.33825900
C	-4.49751100	-0.57257600	2.42371900
F	-6.20646600	1.63277300	-2.36413100
F	-5.29266700	0.07882800	-3.57609100
F	-6.84182800	-0.43084200	-2.13020500
F	-4.64255100	0.57038900	3.13524400
F	-5.67237500	-1.23559000	2.50325500
F	-3.56887200	-1.31592900	3.06424800
C	0.10741300	0.26611800	-0.25717000
H	4.45312600	4.95363600	-0.32199400

Alkene 1a

0 1

S	1.22684100	-0.80364200	1.13217000
O	0.95358300	-2.23145600	0.89435500
O	1.63346200	-0.32350600	2.46137600
C	2.44668000	-0.25490700	-0.06232300
C	3.21971100	0.87260100	0.22654000
C	2.59823600	-0.96788500	-1.25461400
C	4.16208300	1.29639700	-0.71085600
H	3.09338600	1.39409500	1.16879300
C	3.54608100	-0.53311400	-2.17990000
H	1.99625100	-1.85068300	-1.44102600
C	4.32259200	0.59732100	-1.90953800
H	4.77260200	2.16941500	-0.50111300
H	3.68146100	-1.07945600	-3.10853900
H	5.05928600	0.93144900	-2.63449900
C	-0.21236700	1.50234100	0.86063200
N	-0.09620800	2.65277200	1.00777300
C	-0.29201800	0.09739700	0.68146500
C	-1.35699700	-0.63025300	0.26344400
C	-2.70456500	-0.22939300	-0.11440700
C	-3.58739400	-1.25933100	-0.50314900
C	-3.17670200	1.10029400	-0.11829600
C	-4.89511500	-0.97582400	-0.88378200
H	-3.23529600	-2.28751400	-0.50156600
C	-4.48453200	1.37868100	-0.49985700
H	-2.52614700	1.91491900	0.17591800
C	-5.34672600	0.34570000	-0.88310400
H	-5.56003600	-1.78200700	-1.17902800
H	-4.83511200	2.40651000	-0.49799400
H	-6.36716200	0.57209700	-1.17904400
H	-1.16539100	-1.70040300	0.20959100

I-Si

0 1

N	-1.87019500	-0.12019800	0.01819900
C	-3.03035700	-0.47042500	0.84004500
C	-2.62710900	-1.39095800	2.01835200
H	-3.39105200	0.46971100	1.25900100
C	-4.12869600	-1.04331000	-0.05900600
H	-2.23697000	-2.32297300	1.59262800
C	-3.82229300	-1.71611100	2.96979100
N	-1.48985800	-0.82703800	2.78084900
C	-4.95915900	-0.16475400	-0.83197800
C	-4.33303000	-2.40087100	-0.20333200

C	-3.32592000	-1.60073900	4.42580500
H	-4.21422400	-2.72061400	2.77360000
H	-4.65491000	-1.01801300	2.80835300
C	-1.90847200	0.30582400	3.63419700
C	-0.95687800	-1.89310100	3.65130300
C	-4.83573800	1.25290500	-0.80879100
C	-5.95526000	-0.76294200	-1.67104500
C	-5.34193000	-2.88418300	-1.06824100
H	-3.73166700	-3.12160700	0.33989800
C	-2.96308900	-0.12858500	4.69816200
H	-4.11053400	-1.93367200	5.11482900
C	-2.05979900	-2.46496500	4.59790500
H	-2.28453800	1.10486900	2.99060300
H	-1.00397100	0.69625700	4.11410600
H	-0.53353700	-2.67783600	3.01405300
H	-0.12960300	-1.46299700	4.22740100
C	-5.67228200	2.03835800	-1.58033500
H	-4.06275100	1.71386100	-0.20788200
C	-6.80223300	0.08117000	-2.44253700
N	-6.13774000	-2.11093300	-1.77862800
H	-5.49105400	-3.95927600	-1.16622600
H	-3.86167300	0.49832500	4.63898100
H	-2.56534900	-0.02155500	5.71530800
H	-1.72854400	-2.44621100	5.64347600
C	-6.66964900	1.44412100	-2.40163700
O	-5.62768400	3.39781400	-1.63580400
H	-7.55238000	-0.39539300	-3.06599400
H	-7.30840000	2.09697100	-2.98859700
C	-4.67584500	4.07171500	-0.81614900
H	-4.80274900	5.13477200	-1.02675600
H	-4.86484300	3.88179800	0.24760700
H	-3.65041100	3.76623500	-1.05325500
N	-0.06498100	1.19795700	-0.54698500
O	-1.84675600	2.09290700	0.62243200
H	-1.28957600	-0.90815400	-0.25444400
H	0.29028900	0.35098700	-0.98267900
C	0.80413900	2.28868600	-0.60092800
C	0.53687800	3.54141700	-0.02199100
C	2.02347000	2.10321700	-1.27697200
C	1.48229400	4.56232200	-0.12012800
H	-0.40246800	3.69904400	0.48703700
C	2.95402200	3.13541000	-1.35433600
H	2.23273000	1.14924800	-1.74959600
C	2.69851200	4.37975200	-0.77775200
H	3.41842200	5.18506900	-0.85110600
C	4.27765700	2.87043300	-2.01564000
C	1.19928900	5.87700400	0.55914900

F	5.16325400	2.30135600	-1.14800900
F	4.16739700	2.01636800	-3.05702500
F	4.86022900	3.99730500	-2.47131400
F	1.57967700	5.85736200	1.85955000
F	1.86194200	6.90099400	-0.02497000
F	-0.11564100	6.18519800	0.54263800
C	-1.30049900	1.12196200	0.08875200
S	1.15934700	-2.52067700	-1.00144300
O	1.41899400	-1.22943500	-1.67857700
O	-0.15215900	-2.73437600	-0.35859800
C	1.44051600	-3.83925100	-2.17486200
C	0.81317000	-5.07094700	-1.96808400
C	2.28695800	-3.60858500	-3.26310600
C	1.04804800	-6.09860900	-2.88133300
H	0.14773900	-5.21397100	-1.12406800
C	2.50724300	-4.64659600	-4.16676600
H	2.74495200	-2.63585600	-3.40502700
C	1.89254700	-5.88714500	-3.97385500
H	0.56645500	-7.06120500	-2.74039600
H	3.15434800	-4.48443900	-5.02324600
H	2.06906900	-6.69141100	-4.68198400
C	2.25574300	-3.85230500	1.09701100
N	2.07515700	-4.80836300	1.73869300
C	2.43136000	-2.70803400	0.27748000
C	3.39296600	-1.75226800	0.36241800
C	4.50700700	-1.59193400	1.28015300
C	5.30029800	-0.43509700	1.11598200
C	4.83975000	-2.50401200	2.30504700
C	6.38818300	-0.19520300	1.94887400
H	5.05436500	0.27722300	0.33330300
C	5.92932900	-2.25882300	3.13199200
H	4.25178000	-3.40139600	2.45519900
C	6.70506600	-1.10689400	2.95827500
H	6.98652100	0.70024100	1.81128000
H	6.17660000	-2.96744700	3.91670400
H	7.55423900	-0.92241000	3.61010600
H	3.30303900	-0.96464500	-0.38210300
H	-2.28373100	-3.51108900	4.35330200

TS-*Si*

0 1

N	-0.09618000	1.26615900	0.16198900
C	0.23360100	2.66675400	-0.06762600
C	1.59214100	2.86103900	-0.78916700
H	-0.54359600	3.05230400	-0.72678900
C	0.18371500	3.45345600	1.24880200
H	2.39993100	2.49355300	-0.15198800
C	1.81496700	4.35565700	-1.17605900
N	1.71344700	2.02898600	-2.04792100
C	-1.03156200	4.08141600	1.68116900
C	1.28626100	3.54770100	2.07659600
C	1.84092700	4.50357800	-2.70970000
H	2.74965700	4.72597000	-0.74173200
H	1.01091000	4.96932000	-0.75777000
C	0.61966600	2.33190200	-3.03466600
C	3.04583400	2.32166100	-2.69171500
C	-2.26134600	4.00829600	0.96698100
C	-0.98682800	4.81593200	2.91286300
C	1.20830500	4.28004100	3.28297800
H	2.22873300	3.06833400	1.83278800
C	0.57104800	3.85688100	-3.29692200
H	1.88507400	5.56352300	-2.97864400
C	3.07003700	3.75810100	-3.25728200
H	-0.31560300	1.93369700	-2.64104800
H	0.86776500	1.76874800	-3.93565500
H	3.80257200	2.17020600	-1.91935200
H	3.19242700	1.55160400	-3.45091800
C	-3.38659400	4.65330200	1.44745000
H	-2.32830000	3.40231500	0.07281500
C	-2.16257100	5.48042400	3.36172600
N	0.12676200	4.91085400	3.69332300
H	2.08743900	4.34803700	3.92237800
H	-0.32145200	4.29935300	-2.83993900
H	0.50620600	4.04799500	-4.37334000
H	3.04951300	3.74482700	-4.35266000
C	-3.33107700	5.40795100	2.65154800
O	-4.60894500	4.63100100	0.85122000
H	-2.09921400	6.03756900	4.29113400
H	-4.23555200	5.90468400	2.98869200
C	-4.76076100	3.87748800	-0.34870900
H	-5.80757900	3.98485300	-0.63642600
H	-4.11824700	4.26883300	-1.14732900
H	-4.52821100	2.81848800	-0.18890900
N	-1.48705300	-0.56440300	0.15046700
O	-2.03593900	1.32670700	-1.06801200
H	0.33522600	0.83092000	0.97722400

H	-0.67856000	-1.06028500	0.51946700
C	-2.62895900	-1.34804900	-0.02268700
C	-3.81887900	-0.88273300	-0.60456700
C	-2.57860500	-2.66904500	0.45818800
C	-4.92451500	-1.72934800	-0.68635600
H	-3.86472000	0.12613300	-0.98638400
C	-3.69329800	-3.49646000	0.36046700
H	-1.66658600	-3.03594700	0.91846000
C	-4.88254100	-3.03833100	-0.20978400
H	-5.75273100	-3.68009700	-0.26872800
C	-3.59699100	-4.92428900	0.82713300
C	-6.17196000	-1.22067400	-1.35891300
F	-2.70691400	-5.07292600	1.83319400
F	-4.78650600	-5.39262800	1.26633900
F	-3.19827900	-5.74979800	-0.17128500
F	-6.12145800	-1.39134400	-2.70232600
F	-7.28018300	-1.86159700	-0.92559700
F	-6.36078300	0.10164100	-1.14401400
H	1.77228800	0.90540000	-1.86398100
C	-1.26673800	0.72192300	-0.31133800
S	1.83899800	-1.30448300	1.87689100
O	0.96768200	-2.25603100	1.14686300
O	1.22232400	-0.07276900	2.42938200
C	2.58375300	-2.17987500	3.25316200
C	3.04853300	-1.45100000	4.35098700
C	2.69533400	-3.57101900	3.18989600
C	3.64360600	-2.14012100	5.40782100
H	2.93140100	-0.37343500	4.38207400
C	3.28961200	-4.24473300	4.25634100
H	2.30784300	-4.11054400	2.33247500
C	3.76530100	-3.53102800	5.35969300
H	4.00588200	-1.58919200	6.27050100
H	3.37586000	-5.32680100	4.22691800
H	4.22718800	-4.06156600	6.18734300
C	3.90363200	0.29297500	1.25594100
N	4.47070100	1.23502300	1.65436000
C	3.16726000	-0.81894600	0.79270800
C	3.32759500	-1.48744400	-0.40836400
O	2.08127800	-0.50881800	-1.99626600
O	0.71162100	-0.97723000	-2.11433100
C	0.54962300	-1.85388400	-3.25343400
C	0.95532400	-1.14862300	-4.55343100
H	1.98982000	-0.80295300	-4.48268900
H	0.30357700	-0.28950800	-4.74791600
H	0.87294000	-1.83251400	-5.40580900
C	1.35864300	-3.14158100	-3.06112100
H	1.11369500	-3.60050000	-2.09654600

H	2.42883300	-2.92583100	-3.09221600
H	1.13068000	-3.86432800	-3.85320400
C	-0.95422200	-2.15289500	-3.24168100
H	-1.23916200	-2.69004400	-2.33205000
H	-1.22200900	-2.77166400	-4.10512400
H	-1.53215400	-1.22410400	-3.28723000
C	4.53354000	-1.47715500	-1.25067800
C	4.86552800	-2.66648200	-1.92748700
C	5.39914900	-0.37575200	-1.37890800
C	6.01897700	-2.75472300	-2.70321500
H	4.21965800	-3.53342400	-1.82584900
C	6.54615400	-0.46169700	-2.16544100
H	5.18351200	0.55079300	-0.86124000
C	6.86233700	-1.64915300	-2.82981400
H	6.25843500	-3.68653000	-3.20777600
H	7.19944600	0.40193400	-2.25413800
H	7.76067800	-1.71293700	-3.43743800
H	2.66976600	-2.33627000	-0.54788600
H	3.99419000	4.26283100	-2.95730200

II-*S*

0 1

N	-0.54402000	1.36531500	0.13093100
C	-0.57692100	2.79935900	-0.13505600
C	0.67168600	3.24993200	-0.92278800
H	-1.44931200	2.96244200	-0.76781700
C	-0.75211400	3.61156100	1.15115200
H	1.56366700	3.09996300	-0.31190400
C	0.58226200	4.70790000	-1.44313000
N	0.91747400	2.33682300	-2.12221600
C	-2.06189300	3.96029700	1.61939200
C	0.33427000	3.99698200	1.91383300
C	0.85783200	4.74375900	-2.95749400
H	1.30209600	5.33249200	-0.90609900
H	-0.41226500	5.12061400	-1.23913500
C	-0.15972700	2.45186000	-3.17784100
C	2.26814200	2.67143800	-2.72329400
C	-3.26906000	3.57319600	0.97086800
C	-2.13896500	4.73891900	2.82172400
C	0.13536500	4.74797800	3.09515600
H	1.34965300	3.72768900	1.63872900
C	-0.22270700	3.91450500	-3.67730600
H	0.82897600	5.77782400	-3.31364200
C	2.24164400	4.12927300	-3.23874700
H	-1.09100500	2.09812600	-2.73274900
H	0.12137700	1.75385300	-3.96836200

H	3.00383000	2.50529200	-1.93671200
H	2.44472600	1.93743400	-3.50972500
C	-4.49328700	3.95794400	1.48701100
H	-3.23160300	2.93619400	0.09683900
C	-3.41805700	5.12815300	3.30951900
N	-1.04648700	5.12671200	3.53849500
H	0.99911400	5.04991200	3.68591800
H	-1.21319900	4.33675000	-3.47956300
H	-0.06772600	3.94686000	-4.76046100
H	2.45360000	4.15318400	-4.31261300
C	-4.56578900	4.75376700	2.66344300
O	-5.69957200	3.62733600	0.95320600
H	-3.44803000	5.72408800	4.21616800
H	-5.54654600	5.03889400	3.03105700
C	-5.71968700	2.81832400	-0.21937100
H	-6.77332200	2.66184700	-0.45467200
H	-5.22707800	3.32456400	-1.05906300
H	-5.23192000	1.85178600	-0.04798400
N	-1.55652800	-0.68520100	0.35799200
O	-2.45104000	0.90845900	-1.06600500
H	0.07007000	1.02576400	0.87378200
H	-0.76268800	-0.85075300	0.97955300
C	-2.41348800	-1.77031400	0.14351100
C	-3.63123200	-1.67536600	-0.54669300
C	-2.02651800	-3.00985700	0.68315300
C	-4.43876700	-2.80600400	-0.67742000
H	-3.93187100	-0.72787000	-0.96876300
C	-2.85212900	-4.12166400	0.54491200
H	-1.07570800	-3.09844300	1.19971500
C	-4.06879600	-4.03473700	-0.13475200
H	-4.71326900	-4.89994000	-0.22848600
C	-2.39891900	-5.45435500	1.08051500
C	-5.71485400	-2.69259100	-1.46843300
F	-1.57480200	-5.32362200	2.14119500
F	-3.44440600	-6.21894900	1.46981900
F	-1.72566500	-6.16435200	0.14345000
F	-6.62093100	-3.62820300	-1.10949700
F	-6.30041300	-1.48298100	-1.31467000
F	-5.49428700	-2.84965700	-2.79660100
H	0.94941300	1.35355100	-1.80134400
C	-1.59258300	0.55214200	-0.24742600
S	2.02513400	-1.20616400	1.47662600
O	1.70777200	-2.51957900	0.86559400
O	0.86888800	-0.38152600	1.97134200
C	2.96965600	-1.55440800	2.97633000
C	3.06265300	-0.58397700	3.97817400
C	3.63166900	-2.77881300	3.09043500

C	3.83594200	-0.84857700	5.10861200
H	2.52631900	0.35354300	3.87945900
C	4.39884600	-3.03302200	4.22819300
H	3.52916100	-3.52148600	2.30657400
C	4.50410300	-2.06959500	5.23411300
H	3.91008100	-0.10241300	5.89465200
H	4.90967900	-3.98644100	4.32962100
H	5.10204000	-2.27179700	6.11841000
C	3.15067300	1.06857100	0.67471900
N	3.28779600	2.22025000	0.88165700
C	2.98248500	-0.28252400	0.38787900
C	3.54436400	-0.91428600	-0.84171600
O	2.95369000	-0.37815900	-2.08534700
O	1.52070500	-0.65806900	-2.10021400
C	1.20892500	-1.64343300	-3.13725800
C	1.58901600	-1.09856100	-4.51784500
H	2.65946700	-0.88197200	-4.56946000
H	1.03389300	-0.18178400	-4.74727000
H	1.35055800	-1.83470900	-5.29307200
C	1.90797400	-2.97669600	-2.85271900
H	1.68327400	-3.31736200	-1.83804400
H	2.99237400	-2.89373000	-2.96980000
H	1.55403700	-3.73444300	-3.56040600
C	-0.30859000	-1.79496100	-2.99131400
H	-0.56165900	-2.21508400	-2.01486900
H	-0.68410100	-2.47135000	-3.76615600
H	-0.82318800	-0.83440900	-3.10012200
C	5.04883200	-0.74173300	-1.07453600
C	5.91024700	-0.44411400	-0.01144500
C	5.60500300	-0.95414000	-2.34642700
C	7.28931100	-0.35584600	-0.21218000
H	5.49893900	-0.28817000	0.97995200
C	6.98220900	-0.85960700	-2.54850700
H	4.95663500	-1.18995900	-3.18307700
C	7.83128200	-0.55923400	-1.48149300
H	7.93842700	-0.12451800	0.62824800
H	7.39183300	-1.02465400	-3.54188300
H	8.90434100	-0.48717100	-1.63806000
H	3.30445400	-1.97926400	-0.80650300
H	3.02181400	4.71388600	-2.74148800

TS-*SS*

0 1

N	-0.91350600	-1.20342100	-0.09896000
C	-1.30941900	-2.48332900	0.45920700
C	-0.19781000	-3.06399400	1.36203400
H	-2.18119000	-2.28161600	1.08122400
C	-1.72366300	-3.46019400	-0.64803300
H	0.73215200	-3.10534000	0.79101100
C	-0.54924100	-4.45616400	1.95265600
N	0.13398800	-2.13779700	2.52759800
C	-3.09178600	-3.55480100	-1.06537400
C	-0.79155000	-4.24030800	-1.30509700
C	-0.14253000	-4.51594700	3.43541900
H	-0.03746700	-5.23293500	1.37795800
H	-1.62446000	-4.64846100	1.85593100
C	-0.93947700	-2.14199900	3.58660500
C	1.44062600	-2.59116300	3.14088800
C	-4.14189600	-2.75182300	-0.53623400
C	-3.39783700	-4.51273400	-2.08882800
C	-1.20550500	-5.13482800	-2.31798100
H	0.26581500	-4.17511800	-1.07216900
C	-1.04115500	-3.54679400	4.22480900
H	-0.26588600	-5.53481400	3.81464900
C	1.32529800	-4.07130200	3.57068000
H	-1.86462200	-1.81091300	3.11304100
H	-0.65307900	-1.37690600	4.30921700
H	2.21319500	-2.40890000	2.39470300
H	1.62676800	-1.92132100	3.98207800
C	-5.43835900	-2.91111400	-0.99046000
H	-3.91473900	-1.97393000	0.18105100
C	-4.74886600	-4.66021300	-2.51168100
N	-2.45858300	-5.29238600	-2.69522500
H	-0.46164100	-5.74848000	-2.82455100
H	-2.07991100	-3.89148100	4.20510500
H	-0.73204300	-3.51130700	5.27490200
H	1.66814300	-4.18747000	4.60394300
C	-5.74542200	-3.88697600	-1.97865000
O	-6.50418800	-2.18063800	-0.56631200
H	-4.95581600	-5.39957100	-3.27899300
H	-6.77750800	-3.98502900	-2.30054500
C	-6.28006400	-1.16878500	0.41205400
H	-7.24931200	-0.69678600	0.57887800
H	-5.91542600	-1.60104800	1.35236200
H	-5.55964400	-0.42209800	0.05909800
N	-1.13273500	1.00834400	-0.63802800
O	-2.64553500	-0.00209300	0.80322700
H	-0.12555700	-1.18287700	-0.74054400

H	-0.22719100	0.85077200	-1.06957400
C	-1.60520900	2.31976700	-0.67851200
C	-2.82490500	2.72880800	-0.11416900
C	-0.81743700	3.27039700	-1.35091800
C	-3.22434200	4.06066000	-0.22647300
H	-3.43899900	2.00641500	0.40283700
C	-1.23824800	4.59288500	-1.45361300
H	0.12819300	2.97063800	-1.79189300
C	-2.44674200	5.00706900	-0.89186400
H	-2.77058200	6.03730100	-0.97093100
C	-0.40719500	5.57363200	-2.23692500
C	-4.50125300	4.49043600	0.44715100
F	-0.53965800	6.83366700	-1.76873600
F	0.90929200	5.26422800	-2.20154700
F	-0.76550900	5.60142200	-3.54284900
F	-5.43476100	3.51274900	0.43429900
F	-4.29510900	4.81493800	1.74614500
F	-5.04567700	5.57651900	-0.14519700
H	0.28282000	-1.11836200	2.23517900
C	-1.64098600	-0.05923800	0.08693200
S	2.58894400	-1.32124000	-1.43346900
O	1.47303800	-0.38157700	-1.73081400
O	2.24494200	-2.71748600	-1.08098600
C	3.63076800	-1.38437900	-2.89282900
C	4.47347100	-2.48374300	-3.07900000
C	3.59034200	-0.32576700	-3.80409400
C	5.29627100	-2.51466300	-4.20481400
H	4.47506200	-3.29926300	-2.36450600
C	4.41520700	-0.37440900	-4.92712500
H	2.91497700	0.50680300	-3.64099600
C	5.26810200	-1.46367900	-5.12475300
H	5.95554000	-3.36266700	-4.36380100
H	4.38881900	0.43672900	-5.64866900
H	5.90981700	-1.49510900	-6.00056000
C	4.68395300	-1.34344800	0.26427200
N	5.60296400	-1.99592600	0.58077700
C	3.57912300	-0.59275700	-0.16222900
C	3.08287400	0.65362300	0.48648400
O	2.25977000	-0.19345100	1.26530200
O	0.67541300	0.50463200	2.11691100
C	0.68350900	1.53160600	3.09106200
C	1.57496700	1.18772000	4.29685300
H	2.61173300	1.04919700	3.97936200
H	1.23507900	0.26440800	4.78260200
H	1.55168000	1.98613400	5.04823200
C	1.11856300	2.87050300	2.46732900
H	0.49888200	3.09482500	1.59269900

H	2.16441400	2.83798200	2.15375700
H	1.00977800	3.69239000	3.18517300
C	-0.78890200	1.66769500	3.55522000
H	-1.44745400	1.83397400	2.69846600
H	-0.88943800	2.51217200	4.24792600
H	-1.12794800	0.76513400	4.07377100
C	4.14073100	1.47699300	1.18771000
C	4.53849200	2.69776500	0.62904000
C	4.76768200	1.02513500	2.35478300
C	5.54562200	3.45675900	1.22622700
H	4.05406500	3.05809800	-0.27595400
C	5.77246500	1.78488500	2.95492900
H	4.46376400	0.07882400	2.78984800
C	6.16452100	3.00163800	2.39245300
H	5.84214500	4.40396000	0.78396400
H	6.25116400	1.42452800	3.86144700
H	6.94653400	3.59273900	2.86122000
H	2.49303800	1.27026200	-0.19929500
H	1.96687500	-4.70071300	2.94519900

TS-S_{rot}

0 1

N	1.14015300	-1.21494700	0.41466100
C	1.46442500	-2.49266800	-0.19856600
C	0.20705100	-3.21303300	-0.75196600
H	2.11139200	-2.25471300	-1.04163500
C	2.22421500	-3.43272700	0.74873900
H	-0.38836100	-3.59608100	0.07730500
C	0.55877700	-4.34494100	-1.75621700
N	-0.74578800	-2.25016300	-1.44364300
C	3.63760800	-3.63362100	0.63154700
C	1.55972600	-4.10299700	1.75872800
C	-0.17848600	-4.10757700	-3.08909800
H	0.28217600	-5.31647700	-1.33582200
H	1.64025400	-4.36959200	-1.92760400
C	-0.14282500	-1.61966300	-2.67320800
C	-2.01988500	-2.97770200	-1.81469300
C	4.46761400	-2.93825700	-0.29304800
C	4.23641100	-4.59082200	1.51917900
C	2.25775200	-4.99701300	2.59972400
H	0.49725500	-3.95749800	1.93087100
C	0.24213800	-2.74208800	-3.66841400
H	0.07603100	-4.90362900	-3.79489500
C	-1.69619000	-4.09541500	-2.83136700
H	0.71285300	-1.02388200	-2.35430800
H	-0.90281400	-0.94083000	-3.06269500

H	-2.43664700	-3.35250900	-0.87889400
H	-2.69855000	-2.21685100	-2.20079200
C	5.82269100	-3.20631200	-0.35220100
H	4.04848700	-2.14167400	-0.89241200
C	5.63068900	-4.85472100	1.40730200
N	3.54378300	-5.26172100	2.48100300
H	1.72031000	-5.52182900	3.38838000
H	1.32093200	-2.72789500	-3.85449400
H	-0.25287600	-2.57314600	-4.62972700
H	-2.23781100	-3.92248000	-3.76664300
C	6.40511700	-4.18971200	0.49504500
O	6.70158600	-2.58007300	-1.17860000
H	6.05631400	-5.59198000	2.08044300
H	7.47116600	-4.37541400	0.40902600
C	6.20658000	-1.55545200	-2.03659700
H	7.07464700	-1.17456100	-2.57627300
H	5.47723300	-1.95636700	-2.75186000
H	5.74114200	-0.74485700	-1.46418000
N	1.35126600	1.05597000	0.73001600
O	2.77079000	-0.07045400	-0.71539200
H	0.33050700	-1.17265800	1.03273400
H	0.42262900	0.98293800	1.15810800
C	1.80659000	2.36830200	0.54433900
C	3.07963500	2.68531200	0.04561100
C	0.93936700	3.40948800	0.91443100
C	3.45381600	4.02257300	-0.08844600
H	3.75742200	1.89297900	-0.23538200
C	1.33328100	4.73692900	0.76698900
H	-0.04323300	3.17184300	1.30887300
C	2.59341500	5.06181700	0.26393200
H	2.89901200	6.09538100	0.15990000
C	0.34681700	5.82909900	1.08092500
C	4.79887500	4.34375200	-0.68446300
F	0.95712100	6.99495700	1.38402300
F	-0.46694300	6.07973500	0.02144800
F	-0.45596700	5.50752500	2.11758600
F	4.74383900	4.39926200	-2.03795000
F	5.26649200	5.54024700	-0.26453200
F	5.72856800	3.41380000	-0.37227700
H	-1.02287900	-1.53319500	-0.74451500
C	1.83091200	-0.07193400	0.08600400
S	-2.35905800	-0.11839500	1.56573500
O	-1.46608900	1.09256500	1.52255200
O	-1.61433900	-1.33572300	1.03498700
C	-2.68099900	-0.47214600	3.29748600
C	-2.94975600	-1.78234100	3.69833200
C	-2.60704000	0.57496200	4.21697300

C	-3.16429700	-2.04204900	5.05122000
H	-2.98734100	-2.57926500	2.96387700
C	-2.82146700	0.30134600	5.56799300
H	-2.38003700	1.57916800	3.87639100
C	-3.10211200	-1.00233600	5.98288800
H	-3.37920200	-3.05525000	5.37806100
H	-2.76894000	1.10666100	6.29463300
H	-3.27062700	-1.20997600	7.03578300
C	-5.02282000	0.02608300	1.67068000
N	-6.00742300	0.00164000	2.30997600
C	-3.89170400	0.05981700	0.84878700
C	-4.06273000	0.50895200	-0.59885600
O	-2.75421900	0.38730800	-1.23052800
O	-2.82035000	1.11369600	-2.49745300
C	-2.02505900	2.34188100	-2.45320300
C	-2.46048000	3.27148800	-1.31697500
H	-2.32237700	2.79064300	-0.34492200
H	-3.50924300	3.56686200	-1.42697000
H	-1.85053000	4.18103700	-1.32703900
C	-0.53917000	1.99637600	-2.34396400
H	-0.21882600	1.38474100	-3.19390900
H	-0.34248900	1.45472900	-1.41586300
H	0.06631100	2.90845500	-2.33614700
C	-2.35164400	2.94754300	-3.82418500
H	-2.08437700	2.25674700	-4.63065000
H	-1.78199600	3.87272000	-3.95960900
H	-3.41771200	3.18198400	-3.90405000
H	-2.02289700	-5.06501000	-2.44230900
C	-5.12224600	-0.27564000	-1.36576300
C	-5.88780200	0.37546800	-2.34063100
C	-5.33526600	-1.64224700	-1.14512300
C	-6.83508600	-0.32344400	-3.09037500
H	-5.73893300	1.43792800	-2.51246500
C	-6.28023700	-2.34554700	-1.89315000
H	-4.77352200	-2.14219400	-0.36173600
C	-7.03149800	-1.68811900	-2.87045300
H	-7.42341300	0.19853000	-3.84050300
H	-6.44230800	-3.40351800	-1.70265600
H	-7.77427100	-2.23314900	-3.44713600
H	-4.32812400	1.56970800	-0.64670400

II-*S′*

0 1

N	0.61995400	1.63808000	-0.39804000
C	0.76665500	2.94735100	0.22120700
C	-0.52257200	3.40691800	0.94561400
H	1.53694700	2.82016500	0.98156200
C	1.24923800	4.00243700	-0.78178500
H	-1.29533700	3.63912100	0.21231800
C	-0.27334400	4.60159100	1.90610200
N	-1.14871500	2.28030900	1.75167900
C	2.65148900	4.22126500	-0.98996800
C	0.36358100	4.74151800	-1.54231700
C	-0.71740900	4.22709600	3.33394100
H	-0.81836400	5.48390900	1.55679300
H	0.79015200	4.86374800	1.90323400
C	-0.25871200	1.79024500	2.86775800
C	-2.47187900	2.75318500	2.31571800
C	3.67655400	3.49237100	-0.32421900
C	3.02188800	5.23468700	-1.93627800
C	0.84219300	5.70156500	-2.46264300
H	-0.71021800	4.60844300	-1.45914500
C	0.06422800	2.98279400	3.80095700
H	-0.52112600	5.06448100	4.01000200
C	-2.22101300	3.89859900	3.32194400
H	0.63049700	1.34985600	2.41634300
H	-0.81293300	0.99106700	3.36287700
H	-3.07825900	3.05206000	1.46032200
H	-2.94356200	1.87750200	2.76185800
C	5.00782200	3.77000800	-0.57863500
H	3.41524000	2.68440700	0.34635400
C	4.40259800	5.50159600	-2.15480500
N	2.11933600	5.96123800	-2.65352700
H	0.13095100	6.27774100	-3.05278800
H	1.13941800	3.18990700	3.79005600
H	-0.20491000	2.73395500	4.83229900
H	-2.55111800	3.60412000	4.32327900
C	5.37218900	4.79455800	-1.49566200
O	6.05702200	3.12030100	-0.01104700
H	4.65562400	6.27878300	-2.86886800
H	6.42815300	4.98445500	-1.66046700
C	5.78152600	2.06934700	0.91266900
H	6.75368300	1.67592500	1.21250400
H	5.25013500	2.44783100	1.79473700
H	5.18609300	1.27439100	0.44994700
N	1.32091400	-0.50166700	-0.89343000
O	2.28376100	0.67833800	0.85062100
H	0.01483600	1.53659200	-1.21063600

H	0.53851200	-0.46678900	-1.54834300
C	1.98550600	-1.73376300	-0.78795400
C	2.96801300	-2.00885800	0.17373800
C	1.63281200	-2.73485000	-1.70979700
C	3.55904300	-3.27296900	0.21435900
H	3.25125400	-1.24568200	0.88246300
C	2.24922200	-3.98106700	-1.66075500
H	0.85936500	-2.54895800	-2.44674700
C	3.21808900	-4.26866700	-0.69664000
H	3.69543400	-5.24014900	-0.66371400
C	1.82666200	-5.06213300	-2.62204000
C	4.52640700	-3.56705100	1.32768400
F	0.87133500	-5.85748600	-2.09241200
F	1.33836800	-4.55943300	-3.77331900
F	2.86801300	-5.86915500	-2.94341000
F	5.32823700	-2.51197600	1.59836600
F	3.87454200	-3.86015200	2.48472900
F	5.32376800	-4.62115100	1.05351700
H	-1.38000500	1.51243300	1.09171200
C	1.47576900	0.60198900	-0.08559000
S	-2.43269800	0.12873700	-1.34778900
O	-1.22712800	0.30304900	-2.22658900
O	-2.61894500	1.13697500	-0.25743900
C	-3.81691600	0.38187700	-2.47869900
C	-4.96753900	1.03345100	-2.02946100
C	-3.72927600	-0.12340300	-3.77979100
C	-6.04424900	1.18783400	-2.90419000
H	-5.01076900	1.41606100	-1.01646700
C	-4.81527500	0.03173400	-4.64090300
H	-2.82359900	-0.61787000	-4.11327200
C	-5.97070900	0.68658500	-4.20574000
H	-6.94037800	1.70114400	-2.56675500
H	-4.75527800	-0.35527400	-5.65400200
H	-6.81222700	0.80783000	-4.88212400
C	-2.01678100	-2.46141700	-1.52324200
N	-1.67912200	-3.31605500	-2.25404500
C	-2.43514400	-1.43661800	-0.66413300
C	-2.80495000	-1.72097700	0.76153100
O	-1.68713200	-1.27729700	1.59046900
O	-1.76672600	-1.98398200	2.88439300
C	-0.59329200	-2.82570700	3.06711200
C	-0.47461800	-3.86939100	1.95281100
H	-0.32719800	-3.39414300	0.97984200
H	-1.37277400	-4.49471100	1.90692700
H	0.38479500	-4.52154500	2.14267300
C	0.66704700	-1.96049200	3.16345100
H	0.59624200	-1.27135400	4.01297700

H	0.80745200	-1.37764800	2.25035000
H	1.55586300	-2.58590500	3.29978300
C	-0.90756700	-3.49258800	4.41306300
H	-1.04511200	-2.74058100	5.19715200
H	-0.07788900	-4.14567400	4.70322600
H	-1.81797900	-4.09653300	4.34532100
C	-4.15193500	-1.18255800	1.24854500
C	-5.29643500	-1.42090500	0.47508900
C	-4.30217600	-0.53676900	2.48103900
C	-6.55526500	-1.00503800	0.91031500
H	-5.19725700	-1.93218400	-0.47846000
C	-5.56159500	-0.11513000	2.91636800
H	-3.43540000	-0.39715300	3.11606500
C	-6.69321600	-0.34297900	2.13241300
H	-7.42797700	-1.19903800	0.29208500
H	-5.65824600	0.38251400	3.87855600
H	-7.67273700	-0.01830000	2.47357400
H	-2.83706200	-2.81218800	0.85481000
H	-2.80150500	4.78158200	3.03636400

TS-*SR*

0 1

N	-0.05349700	1.16954800	0.08910300
C	0.43156800	2.53359900	-0.05832100
C	1.65963100	2.61602300	-0.99751600
H	-0.37812600	3.09805900	-0.51938400
C	0.72273200	3.14681400	1.31622200
H	2.45361600	1.97617200	-0.60747500
C	2.14092000	4.08491600	-1.19706500
N	1.37298900	2.03910600	-2.37786200
C	-0.29517400	3.86976900	2.02093300
C	1.94618500	2.98211700	1.93681900
C	1.95937800	4.51477600	-2.66413000
H	3.19019600	4.18113600	-0.89998200
H	1.56855400	4.75047300	-0.54423700
C	0.22658500	2.74099800	-3.06524500
C	2.61902800	2.17454300	-3.22910700
C	-1.62094100	4.05406200	1.53520300
C	0.06058200	4.42347600	3.29570300
C	2.17881600	3.55654500	3.20699700
H	2.74238000	2.40582600	1.47767900
C	0.49717100	4.26298800	-3.07995800
H	2.20304300	5.57609600	-2.77062100
C	2.88261300	3.66066900	-3.54878700
H	-0.69402700	2.46612000	-2.54819000
H	0.18511300	2.32205700	-4.07191300

H	3.42760700	1.71251500	-2.66025800
H	2.44345200	1.56053900	-4.11215200
C	-2.54309600	4.77005700	2.27682700
H	-1.92432700	3.59003300	0.60587500
C	-0.91252200	5.17033100	4.01753200
N	1.28756300	4.26751200	3.86807700
H	3.14922900	3.42331500	3.68328900
H	-0.18260200	4.78169200	-2.39498600
H	0.30826800	4.66176300	-4.08181200
H	2.69312400	3.86818800	-4.60758800
C	-2.17858200	5.34439400	3.52603500
O	-3.83463600	4.98486700	1.91119000
H	-0.61531100	5.58636300	4.97490700
H	-2.93066000	5.90500800	4.07231900
C	-4.28875500	4.43025100	0.67935300
H	-5.34323600	4.69889900	0.60336600
H	-3.73999900	4.85305900	-0.17155400
H	-4.18325900	3.33937700	0.66755000
N	-1.70333800	-0.40810700	0.32005300
O	-2.12184500	1.56492000	-0.82316100
H	0.46061100	0.57420000	0.73733900
H	-0.96929700	-0.91496500	0.80538200
C	-2.91629100	-1.09247400	0.16604200
C	-4.07144100	-0.50389100	-0.37047300
C	-2.96493800	-2.42929300	0.59668200
C	-5.24562200	-1.25136700	-0.46250100
H	-4.03867100	0.52039400	-0.71037900
C	-4.15140300	-3.15201200	0.50161400
H	-2.07142900	-2.90797200	0.98803700
C	-5.30587300	-2.57392700	-0.02816700
H	-6.22486700	-3.14177100	-0.10223800
C	-4.20620600	-4.55982700	1.03476500
C	-6.45431000	-0.62126200	-1.10202500
F	-3.02140800	-5.19292200	0.92466200
F	-4.54359300	-4.57951000	2.34759900
F	-5.12927900	-5.30598200	0.38575500
F	-6.53074900	0.70440500	-0.84369000
F	-6.43340700	-0.75289500	-2.45028800
F	-7.60641600	-1.18254600	-0.67270300
H	1.17833900	0.98700500	-2.37944800
C	-1.35650500	0.83342200	-0.18226500
S	2.43517700	-1.17803200	1.43281300
O	1.09854400	-0.77699300	1.95677300
O	3.31479500	-0.13397800	0.87768400
C	3.29053500	-1.97086700	2.79653100
C	4.68234200	-1.86722100	2.86841500
C	2.55355100	-2.67459300	3.75365000

C	5.34766600	-2.49012400	3.92428000
H	5.22368400	-1.30166800	2.11810400
C	3.23501900	-3.29536000	4.80022300
H	1.47258500	-2.72348800	3.68663400
C	4.62679800	-3.20472100	4.88455400
H	6.42835000	-2.41263600	3.99868300
H	2.67637700	-3.84442400	5.55214000
H	5.15062000	-3.68814000	5.70418000
C	1.18398800	-3.39402900	0.57007800
N	0.38340800	-4.18174700	0.89710200
C	2.15362700	-2.43771900	0.21236200
C	2.67402000	-2.35041300	-1.17726800
O	1.60528000	-1.43389200	-1.31065000
O	1.35649800	-0.57186100	-3.07038300
C	0.40666400	-1.28329600	-3.83933700
C	0.85187100	-2.74527200	-4.04103900
H	0.80722900	-3.29409700	-3.09590800
H	1.87923800	-2.78232200	-4.42087700
H	0.20154800	-3.26221800	-4.75677300
C	-0.98579100	-1.25359100	-3.18605500
H	-1.33994500	-0.22404600	-3.05615100
H	-0.94221300	-1.72532900	-2.20077100
H	-1.72556000	-1.79052600	-3.79253600
C	0.35379200	-0.59545800	-5.22447000
H	0.00611900	0.44013200	-5.13794400
H	-0.33676200	-1.12145900	-5.89501100
H	1.34713300	-0.59152100	-5.68712500
C	4.09862900	-1.92374900	-1.46194100
C	5.15379000	-2.49696300	-0.74231700
C	4.38334600	-1.06997500	-2.53260100
C	6.47734400	-2.19394800	-1.06569200
H	4.94046400	-3.18199500	0.07452500
C	5.70883200	-0.76740500	-2.85344300
H	3.55244100	-0.67601400	-3.10842200
C	6.75938800	-1.32252400	-2.12003400
H	7.28738200	-2.64123900	-0.49540700
H	5.92225000	-0.10304200	-3.68759800
H	7.78967900	-1.08660300	-2.37302000
H	2.46712500	-3.29053900	-1.70570500
H	3.93268200	3.90163000	-3.35487600

Epoxide ***SS***

0 1

N	0.14576200	-1.40037800	-0.05902300
C	0.77213500	-2.71829800	0.02753100
C	1.94679600	-2.77579800	1.03641500
H	-0.00766800	-3.37917600	0.40517700
C	1.16581800	-3.19661000	-1.37571600
H	2.73990000	-2.10532900	0.68816000
C	2.50307100	-4.23414000	1.15364000
N	1.58172600	-2.24894400	2.38100200
C	0.21210600	-3.85895700	-2.21924300
C	2.42575000	-2.96999000	-1.89466400
C	2.57951100	-4.62415500	2.64069700
H	3.49107100	-4.30805500	0.68551800
H	1.85294200	-4.94078000	0.62266300
C	0.60592800	-3.13161300	3.06822600
C	2.81988300	-2.19519300	3.19868100
C	-1.13349800	-4.12238300	-1.83633600
C	0.65568700	-4.26789000	-3.52074500
C	2.75118700	-3.40469500	-3.19949900
H	3.18983000	-2.45966200	-1.31868000
C	1.15771400	-4.57935900	3.23253200
H	2.99861200	-5.63172600	2.73943200
C	3.46343200	-3.60194500	3.37709900
H	-0.33435300	-3.10900900	2.51259200
H	0.40423100	-2.67792400	4.04473100
H	3.50964800	-1.49602300	2.71573600
H	2.55076600	-1.76210400	4.16709300
C	-1.99237400	-4.77161100	-2.70450400
H	-1.49548600	-3.77914300	-0.87616400
C	-0.25635100	-4.94395400	-4.37885200
N	1.91416200	-4.04017300	-3.99382800
H	3.75099400	-3.21944000	-3.59101400
H	0.51538000	-5.30242500	2.71499300
H	1.17887000	-4.87100300	4.28956300
H	3.54067600	-3.86410500	4.43923500
C	-1.54454600	-5.19330800	-3.98666700
O	-3.29537700	-5.05774200	-2.43838400
H	0.10636600	-5.24797700	-5.35571100
H	-2.24966600	-5.70408100	-4.63509600
C	-3.82428300	-4.67300600	-1.17185500
H	-4.87170200	-4.97803000	-1.18254200
H	-3.30109100	-5.18217400	-0.35295600
H	-3.75534300	-3.59001600	-1.01960100
N	-1.63114200	0.06990200	-0.01776700
O	-1.88418900	-2.09993300	0.73856700
H	0.64886100	-0.68120000	-0.56674700

H	-0.94117300	0.75609200	-0.31315000
C	-2.93095500	0.56381900	0.13721100
C	-4.01759500	-0.20886400	0.57824800
C	-3.14608700	1.91666000	-0.18322400
C	-5.28137400	0.37347300	0.68265800
H	-3.86164700	-1.24569200	0.83550600
C	-4.41554100	2.47468900	-0.06899800
H	-2.31425700	2.52691000	-0.51870600
C	-5.50148900	1.71184800	0.36394800
H	-6.48801900	2.14984800	0.45080300
C	-4.63317200	3.90595600	-0.48119200
C	-6.41561900	-0.46039000	1.21743900
F	-5.62659400	4.48817800	0.22765900
F	-3.52218400	4.65828000	-0.31171100
F	-4.97649800	4.00519400	-1.78725100
F	-6.31737300	-1.75376500	0.83561600
F	-6.44370600	-0.45635000	2.57226900
F	-7.61906500	-0.00704600	0.80097700
H	1.16406900	-0.31390700	2.47025100
C	-1.17415700	-1.20957900	0.25682100
S	1.17500200	2.78302600	-1.76368700
O	0.24443900	2.29484400	-0.72360700
O	0.83921100	2.65693000	-3.18620700
C	1.61649000	4.47254000	-1.39664200
C	2.02215100	5.29894700	-2.44914500
C	1.54621200	4.92793100	-0.07606000
C	2.37225800	6.61768500	-2.16209200
H	2.04840700	4.91966000	-3.46457200
C	1.89984300	6.24937400	0.19065800
H	1.20740900	4.26986900	0.71646500
C	2.31320000	7.08858000	-0.84809700
H	2.68637700	7.27605200	-2.96587000
H	1.84565300	6.62488200	1.20779800
H	2.58566400	8.11752800	-0.63238900
C	3.80203200	2.22053400	-2.38639900
N	4.62893800	2.59607800	-3.10974100
C	2.74342300	1.81293400	-1.48786800
C	2.98949500	1.16008300	-0.16668000
O	2.50247000	0.43043500	-1.33261600
O	1.20403200	0.67507500	2.45575700
C	0.47368900	1.23331400	3.56195100
C	1.23448000	0.98430000	4.87504900
H	2.25576000	1.37484600	4.80496400
H	1.29292700	-0.08753500	5.09707300
H	0.73591300	1.47202200	5.72105100
C	0.39014300	2.73653100	3.27579100
H	-0.12133600	2.90880100	2.32262400

H	1.39550200	3.17072500	3.21614300
H	-0.16101800	3.25845300	4.06540900
C	-0.93469700	0.62513300	3.63356700
H	-1.48478000	0.81742300	2.70805000
H	-1.50446100	1.04984900	4.46819900
H	-0.88557800	-0.46023000	3.77998900
C	4.34593700	0.82831300	0.34421600
C	4.62617000	1.11592200	1.68774400
C	5.33176100	0.23703900	-0.45926400
C	5.88959000	0.84307100	2.21294500
H	3.84327700	1.52638000	2.31838700
C	6.58994200	-0.04031500	0.07324200
H	5.11364800	-0.01698100	-1.49151800
C	6.87392800	0.26862000	1.40606300
H	6.10082000	1.07062500	3.25390300
H	7.34883200	-0.49903100	-0.55389100
H	7.85666400	0.05352500	1.81610800
H	2.21727200	1.30714100	0.58939800
H	4.48100500	-3.62043400	2.96818400

Epoxide *SR*

0 1

N	-0.16781300	1.32482100	0.11746800
C	-0.90480300	2.54396100	-0.20825900
C	-1.80454500	3.05276400	0.94592200
H	-0.14127800	3.30260800	-0.37783700
C	-1.68152600	2.34181200	-1.51709100
H	-2.60759800	2.32651800	1.10886400
C	-2.41395300	4.44942400	0.58579700
N	-1.09334200	3.11195800	2.24839500
C	-1.04546300	2.54900300	-2.78680500
C	-2.99195300	1.90469000	-1.52684800
C	-2.10375300	5.43601800	1.72700400
H	-3.49632800	4.37130600	0.43043600
H	-1.99092300	4.82812400	-0.35327400
C	-0.01620100	4.13072200	2.23675200
C	-2.07715400	3.48521900	3.29489800
C	0.31022300	2.95753300	-2.93256600
C	-1.83035800	2.31779800	-3.96530600
C	-3.66838700	1.69938000	-2.75038600
H	-3.52873500	1.70446800	-0.60615300
C	-0.57421200	5.54376500	1.88596000
H	-2.52991000	6.41853800	1.49481300
C	-2.70536100	4.88553600	3.03228200
H	0.76019200	3.81201700	1.53767600
H	0.43485200	4.12393800	3.23494700

H	-2.83626000	2.69783600	3.33283800
H	-1.55126000	3.46288800	4.25425700
C	0.85664500	3.13369300	-4.19071500
H	0.92689700	3.09429300	-2.05434900
C	-1.23470200	2.52497300	-5.24111900
N	-3.12824600	1.89959000	-3.93569400
H	-4.70414600	1.36031400	-2.73624600
H	-0.12872800	5.91977900	0.95642500
H	-0.32716100	6.26720100	2.67243200
H	-2.49572600	5.57020100	3.86307600
C	0.07088500	2.92251300	-5.35709000
O	2.14275400	3.50414500	-4.43252000
H	-1.85213700	2.35118500	-6.11690200
H	0.53576200	3.07883700	-6.32562200
C	2.99350600	3.74949700	-3.31437500
H	3.96897200	4.00028400	-3.73364700
H	2.62392000	4.59013400	-2.71414900
H	3.08071600	2.86582600	-2.67241100
N	1.72106600	-0.00384900	0.18945700
O	1.90419300	2.28542700	-0.05987000
H	-0.70822500	0.46983900	0.21036500
H	1.06867700	-0.78269100	0.17862600
C	3.07069200	-0.37476800	0.22370500
C	4.13464600	0.53961400	0.28394200
C	3.35796400	-1.75156800	0.20941000
C	5.44756200	0.06812500	0.32533600
H	3.92584400	1.59891400	0.28913900
C	4.67557000	-2.19660500	0.25173800
H	2.54568300	-2.46933700	0.16691400
C	5.73894400	-1.29428500	0.31008400
H	6.76297000	-1.64492800	0.34271300
C	4.96031300	-3.67199900	0.16259400
C	6.56756100	1.06639500	0.45581400
F	3.95645100	-4.41648300	0.67586900
F	5.12554000	-4.07170700	-1.12209200
F	6.09185200	-4.00728500	0.82185500
F	6.30518900	2.21266100	-0.21121600
F	6.78333600	1.40976600	1.74868100
F	7.73698900	0.58209500	-0.01951400
H	-0.91772500	1.22546400	2.93895200
C	1.19857300	1.27786000	0.07277900
S	-1.37480200	-2.46636100	-0.32431400
O	0.08053900	-2.64279100	-0.14291100
O	-1.96195700	-1.13372200	-0.12415200
C	-1.85699800	-3.09745000	-1.92042000
C	-3.02592200	-2.60377500	-2.50956100
C	-1.05191300	-4.05601200	-2.54435200

C	-3.39576200	-3.09510900	-3.76024800
H	-3.61403400	-1.84233300	-2.00926800
C	-1.44092900	-4.53599600	-3.79401300
H	-0.14111200	-4.40338300	-2.06964700
C	-2.60794300	-4.05864600	-4.39648600
H	-4.29279300	-2.71794600	-4.24116100
H	-0.82860900	-5.27727800	-4.29761800
H	-2.90168300	-4.43471600	-5.37205100
C	-1.67490600	-4.98401400	0.72843000
N	-1.30881000	-6.07506900	0.57411700
C	-2.16032900	-3.62697700	0.89718600
C	-3.48038100	-3.38171300	1.57210800
O	-2.21888600	-3.13828000	2.21718200
O	-1.27774100	0.39821800	3.33564400
C	-0.38688800	-0.08972400	4.35278800
C	-1.18129700	-1.14853900	5.12428600
H	-1.49249900	-1.95491600	4.45268500
H	-2.07880800	-0.70188300	5.56669300
H	-0.57656300	-1.58201000	5.92868600
C	0.85245900	-0.71936200	3.69706700
H	1.41187400	0.02999000	3.12554300
H	0.55145200	-1.51825100	3.01072000
H	1.52930800	-1.14631200	4.44696300
C	0.02623500	1.05585000	5.29043700
H	0.57045600	1.83337600	4.74010600
H	0.68199500	0.69409700	6.09091700
H	-0.85732800	1.51224300	5.75028000
C	-4.42441000	-2.24975700	1.34832600
C	-5.62417400	-2.51642200	0.67384100
C	-4.18122400	-0.97423800	1.87255200
C	-6.56924000	-1.50591400	0.50011000
H	-5.82114800	-3.51467100	0.28917700
C	-5.14120900	0.02581700	1.70991500
H	-3.25251300	-0.76145500	2.39343000
C	-6.32929800	-0.23225200	1.02149800
H	-7.49473100	-1.71663800	-0.02806700
H	-4.96031400	1.01114200	2.13088900
H	-7.07092000	0.55257900	0.90036000
H	-3.95180000	-4.30755700	1.90720500
H	-3.79668900	4.81553900	2.94431800

I-*Re*

0 1

N	-0.45531300	1.46142700	-0.24936500
C	-0.55764600	2.91832900	-0.28193200
C	0.48624400	3.58795600	-1.21106700
H	-1.54216200	3.12843000	-0.69946000
C	-0.51322500	3.47809800	1.14754100
H	1.48298400	3.44064400	-0.78273400
C	0.18942200	5.11640100	-1.36487200
N	0.56363400	2.93661600	-2.54560600
C	-1.70484700	3.55926200	1.94366500
C	0.67133000	3.87747200	1.73566400
C	0.05486800	5.45009000	-2.86297300
H	0.98862600	5.71334200	-0.91050700
H	-0.73683000	5.38587000	-0.84253200
C	-0.70052500	3.09372500	-3.30791500
C	1.65789500	3.58363200	-3.31486400
C	-2.98890800	3.14705600	1.48816400
C	-1.57599100	4.08196700	3.27377500
C	0.68461000	4.36647300	3.06128300
H	1.61244100	3.82853100	1.19905400
C	-1.08112900	4.59681900	-3.46112900
H	-0.16683700	6.51582400	-2.98735900
C	1.37334100	5.09198200	-3.57194100
H	-1.48519300	2.52159900	-2.80859100
H	-0.53807400	2.62587700	-4.28441800
H	2.58601000	3.44280600	-2.75038700
H	1.76550000	3.03041800	-4.25344600
C	-4.09267900	3.25809300	2.31423800
H	-3.09363400	2.70753500	0.50508500
C	-2.73883400	4.19212700	4.08665000
N	-0.38951900	4.47998300	3.81561900
H	1.62881900	4.68162400	3.50500700
H	-2.02303800	4.81495700	-2.94276900
H	-1.23177700	4.85402100	-4.51640200
H	1.29154200	5.29656700	-4.64615800
C	-3.96542600	3.79467000	3.62502300
O	-5.35487700	2.87916800	1.97792700
H	-2.61507500	4.59707600	5.08617400
H	-4.85772500	3.86998900	4.23880000
C	-5.57024500	2.33313600	0.67828700
H	-6.63084800	2.08121800	0.63471600
H	-5.33393500	3.06643500	-0.10270500
H	-4.96766400	1.43281200	0.51499300
N	-1.32374200	-0.67987800	-0.28802300
O	-2.55714700	1.10148100	-1.08669600
H	0.35001800	1.06325700	0.22781400

H	-0.43198500	-0.95371700	0.11558700
C	-2.24918100	-1.72036700	-0.42721700
C	-3.49719800	-1.58384500	-1.05656400
C	-1.89079100	-2.97344300	0.10425800
C	-4.35604400	-2.68147600	-1.13294500
H	-3.78385600	-0.62629000	-1.46470800
C	-2.76187200	-4.05416500	0.00852600
H	-0.93551400	-3.08916300	0.60482200
C	-4.00758700	-3.92399500	-0.60833900
H	-4.68921600	-4.76334100	-0.66516700
C	-2.33185900	-5.39768900	0.53404600
C	-5.66797300	-2.51425800	-1.85288200
F	-1.47012800	-5.28813400	1.56945300
F	-3.38427400	-6.13211600	0.95987500
F	-1.70629200	-6.13005200	-0.41892500
F	-5.52044700	-2.63062900	-3.19494700
F	-6.57703900	-3.44132800	-1.47751400
F	-6.21635200	-1.29822200	-1.62868500
H	1.40008200	1.30992200	-2.34347200
C	-1.51654300	0.66284500	-0.57865400
S	2.12198100	-0.94220700	1.66710800
O	1.10074500	-1.93635800	1.27344700
O	1.91168900	0.47245300	1.29143100
C	2.32763200	-1.02712000	3.44176600
C	2.77232100	0.10993200	4.12246100
C	2.02951700	-2.22059600	4.10493200
C	2.92578100	0.04127600	5.50614700
H	2.97184200	1.02818800	3.58121600
C	2.19163300	-2.27174300	5.48912700
H	1.66695700	-3.08019700	3.55247700
C	2.63974600	-1.14642900	6.18515800
H	3.26106600	0.91674300	6.05367600
H	1.96272700	-3.18942700	6.02192900
H	2.76080100	-1.19291700	7.26347900
C	4.23793500	-2.65537400	1.47712700
N	4.62841100	-3.65534600	1.93147300
C	3.71936500	-1.43886000	0.96545300
C	4.22653800	-0.66212900	-0.02634100
O	2.18638500	0.68930800	-2.32766300
O	1.57447700	-0.59885600	-2.03949300
C	1.47232400	-1.38610200	-3.25828900
C	0.52160300	-0.70531600	-4.24843600
H	0.93060000	0.25370500	-4.58042900
H	-0.45406300	-0.52817700	-3.78338500
H	0.37238700	-1.33486000	-5.13265900
C	2.85938800	-1.60708600	-3.86939400
H	3.52334800	-2.09590800	-3.14917200

H	3.30817600	-0.65498400	-4.16332400
H	2.78502600	-2.24412900	-4.75791600
C	0.88537100	-2.70462500	-2.74220200
H	1.52416900	-3.13116700	-1.96215100
H	0.81323800	-3.42528900	-3.56345900
H	-0.11581800	-2.55519700	-2.32841100
H	3.60613800	0.18748100	-0.30323500
H	2.19137300	5.71388100	-3.18891100
C	5.45726900	-0.79256000	-0.79122500
C	5.62332400	0.09806200	-1.87407800
C	6.48506400	-1.71765200	-0.50954900
C	6.77424400	0.06072300	-2.65506200
H	4.82439800	0.79653900	-2.10469300
C	7.63538700	-1.74485100	-1.29002100
H	6.39292600	-2.40650100	0.32156500
C	7.78379600	-0.85929400	-2.36316400
H	6.88495600	0.74752800	-3.48904400
H	8.42168500	-2.45796600	-1.06086600
H	8.68536100	-0.88803000	-2.96868500

TS-*Re*

0 1

N	-0.12958400	1.30593500	0.14111600
C	0.11711500	2.74025600	0.12154800
C	1.43894300	3.12155500	-0.59239900
H	-0.69703400	3.16691900	-0.46232400
C	0.05991600	3.33904800	1.53340600
H	2.29024400	2.76889600	-0.00585800
C	1.52993400	4.65495800	-0.84717700
N	1.59828300	2.41296300	-1.92183300
C	-1.16120600	3.88458800	2.05158500
C	1.16537700	3.33903000	2.36213700
C	1.51421700	4.93274500	-2.36333700
H	2.43970200	5.06419100	-0.39531800
H	0.68625700	5.15891500	-0.36508700
C	0.45458400	2.70411300	-2.85857800
C	2.88797000	2.85831000	-2.57019700
C	-2.39620000	3.88297900	1.34316100
C	-1.11712300	4.45241200	3.36910300
C	1.08803100	3.90435600	3.65459600
H	2.10845300	2.90267500	2.05003100
C	0.28982400	4.23823300	-2.99151800
H	1.46816400	6.01140100	-2.54097400
C	2.78904300	4.34090400	-2.98954600
H	-0.43977200	2.21426200	-2.47307900
H	0.71642400	2.23333100	-3.80750000

H	3.68126300	2.67886900	-1.84223300
H	3.05797100	2.17957400	-3.40743100
C	-3.52805800	4.43772500	1.91271900
H	-2.46337800	3.39319300	0.38063000
C	-2.29934800	5.03236200	3.90907000
N	0.00098800	4.46169300	4.14815300
H	1.96982900	3.90027200	4.29411900
H	-0.62830400	4.56966300	-2.49394800
H	0.19716300	4.51107400	-4.04767500
H	2.75153100	4.43154800	-4.08044800
C	-3.47311800	5.03137400	3.20415400
O	-4.75528700	4.46652300	1.32944000
H	-2.23679500	5.46403600	4.90294800
H	-4.38276800	5.46255900	3.61032000
C	-4.90629500	3.86916900	0.04374500
H	-5.95981100	3.98399500	-0.21472800
H	-4.28674900	4.37878200	-0.70479800
H	-4.64345800	2.80548600	0.06248700
N	-1.52642500	-0.52717200	-0.00129700
O	-2.11961100	1.48061000	-0.98045200
H	0.44121300	0.74784000	0.77659900
H	-0.75504900	-1.03683700	0.43154800
C	-2.66348700	-1.30104400	-0.26078700
C	-3.80448700	-0.82757300	-0.92551600
C	-2.64783400	-2.62964000	0.20455300
C	-4.90136500	-1.67356200	-1.10249600
H	-3.82563400	0.18923800	-1.28779300
C	-3.75121600	-3.45345000	0.01233300
H	-1.77211600	-3.00384300	0.72392500
C	-4.89468000	-2.98708100	-0.64143400
H	-5.75770500	-3.62771700	-0.77405300
C	-3.70096000	-4.88775700	0.46738700
C	-6.09329800	-1.14682400	-1.85617200
F	-3.35108800	-5.71936300	-0.54285600
F	-2.80747100	-5.07921800	1.46152300
F	-4.90466900	-5.31136600	0.91744300
F	-7.21523200	-1.85384500	-1.59764500
F	-6.35254600	0.14627900	-1.55090500
F	-5.89864800	-1.19424900	-3.19641600
H	1.75076400	1.33284200	-1.77465600
C	-1.31995100	0.79655700	-0.32511400
S	1.83669600	-1.61536400	1.56486100
O	0.57675200	-2.30927400	1.19365300
O	1.74344700	-0.15667400	1.85343800
C	2.43721200	-2.41838100	3.05425600
C	3.18511900	-1.67371600	3.96971300
C	2.15503300	-3.77044000	3.26263900

C	3.65885500	-2.30257900	5.12020000
H	3.37502400	-0.62149400	3.78832300
C	2.64124700	-4.38704200	4.41611200
H	1.55866100	-4.32186500	2.54456200
C	3.39072300	-3.65647700	5.34070400
H	4.23370700	-1.73446900	5.84565500
H	2.42924500	-5.43736400	4.59189600
H	3.76380100	-4.14182800	6.23805400
C	3.39411900	-3.17835500	0.04811700
N	3.62941400	-4.30830500	-0.14084100
C	3.08996200	-1.83295300	0.33307100
C	3.61897800	-0.68681100	-0.25751000
O	2.39908700	-0.08346700	-1.81941700
O	1.24550800	-0.94322500	-1.82161500
C	1.14358700	-1.68701700	-3.06667800
C	0.87244500	-0.72207700	-4.22819700
H	1.71845500	-0.03982200	-4.35899300
H	-0.03410900	-0.13698100	-4.03757500
H	0.73132400	-1.27219200	-5.16538700
C	2.40076100	-2.51569900	-3.33827200
H	2.57043500	-3.24227800	-2.53895900
H	3.27497100	-1.86555400	-3.41674700
H	2.29052100	-3.06514400	-4.28046200
C	-0.06620500	-2.59372600	-2.81675100
H	0.11135000	-3.23492300	-1.94806000
H	-0.24414900	-3.23160700	-3.68924000
H	-0.96842600	-2.00214000	-2.63365200
C	4.90196500	-0.58385400	-0.97769800
C	5.57917600	0.64808700	-0.91341900
C	5.50539900	-1.63473200	-1.69189600
C	6.80934000	0.83339800	-1.54185100
H	5.14086800	1.46246000	-0.34049400
C	6.73385600	-1.44838700	-2.32283000
H	5.01906900	-2.59976000	-1.75583200
C	7.39080200	-0.21692700	-2.25387600
H	7.31552600	1.79230000	-1.46935300
H	7.18203900	-2.27340700	-2.86956200
H	8.34986200	-0.08035900	-2.74582500
H	3.33517500	0.22306400	0.25527600
H	3.67591400	4.88579100	-2.65025300

II-*R*

0 1

N	-0.19423500	1.24304900	0.03455700
C	0.13890300	2.66071800	0.01674400
C	1.43106700	2.92717700	-0.79024400
H	-0.67915600	3.15447400	-0.50579100
C	0.23760800	3.25503300	1.42597500
H	2.28062000	2.45549000	-0.29249500
C	1.69398100	4.43822000	-1.03558400
N	1.38430100	2.23287000	-2.14745500
C	-0.87307900	3.94575600	2.01265100
C	1.38621400	3.11975900	2.18186700
C	1.71646000	4.72909700	-2.54765100
H	2.64170000	4.73374000	-0.57516500
H	0.90984800	5.03056100	-0.55328700
C	0.24892500	2.73698100	-3.01290700
C	2.70148300	2.44182900	-2.86781300
C	-2.14468400	4.08564800	1.38763500
C	-0.67364200	4.51226100	3.31637000
C	1.46214000	3.69752700	3.46878100
H	2.24152100	2.55973500	1.81849000
C	0.37995200	4.27076900	-3.16377200
H	1.85384600	5.80145600	-2.71352100
C	2.87084600	3.94262000	-3.19501700
H	-0.68807400	2.42392600	-2.54980900
H	0.34714100	2.21603200	-3.96756100
H	3.48174100	2.04673600	-2.21636200
H	2.66843900	1.80960700	-3.75582200
C	-3.16213400	4.77432700	2.02277800
H	-2.33590100	3.59684400	0.44138100
C	-1.73931500	5.23445000	3.92358700
N	0.48594100	4.38955200	4.02085800
H	2.37501500	3.58703100	4.05228900
H	-0.45457600	4.77353100	-2.66380600
H	0.33284400	4.54375100	-4.22242000
H	2.87392800	4.09736700	-4.27870400
C	-2.94957400	5.36841500	3.29772900
O	-4.41492500	4.94110100	1.52330000
H	-1.55942200	5.66203800	4.90476900
H	-3.77246800	5.90744400	3.75660700
C	-4.72551600	4.34446600	0.26671200
H	-5.77568200	4.57331800	0.08072000
H	-4.10972500	4.76889600	-0.53625700
H	-4.58212200	3.25819600	0.29315600
N	-1.71094400	-0.48878100	0.02115700
O	-2.26970500	1.56508400	-0.89153500
H	0.37315000	0.65297600	0.65409300

H	-0.92608800	-1.04028600	0.38347700
C	-2.88905500	-1.21736600	-0.19232200
C	-4.06656600	-0.67107000	-0.72366800
C	-2.87075200	-2.57389500	0.17990400
C	-5.19578400	-1.47890200	-0.86965300
H	-4.09004000	0.36965300	-1.01033000
C	-4.00899600	-3.35809900	0.02398200
H	-1.96457100	-3.00062900	0.59739400
C	-5.18688700	-2.82206500	-0.50125500
H	-6.07467100	-3.43316000	-0.60695600
C	-3.95685500	-4.82166100	0.37494200
C	-6.42995100	-0.88209800	-1.49214000
F	-3.03775200	-5.08538400	1.32788800
F	-5.15008900	-5.27105100	0.82603800
F	-3.63743100	-5.58020400	-0.70101900
F	-6.36715700	-0.90449200	-2.84611600
F	-7.55117200	-1.54920200	-1.14178200
F	-6.60172500	0.41176300	-1.13579200
H	1.27682700	1.20639100	-2.00714400
C	-1.46351600	0.82461100	-0.31139000
S	1.76119400	-1.73150600	1.21777600
O	0.46239400	-2.32289500	0.74934700
O	1.66279000	-0.27564700	1.57450300
C	2.15024500	-2.62498400	2.73156200
C	2.87429900	-1.97136600	3.73161200
C	1.74123900	-3.95158700	2.87706600
C	3.18907500	-2.66263100	4.90039900
H	3.17175600	-0.93711700	3.59609800
C	2.07120300	-4.63570400	4.04834600
H	1.16782900	-4.43267900	2.09268900
C	2.79184900	-3.99384000	5.05717000
H	3.74352700	-2.16208600	5.68915600
H	1.76047200	-5.66912700	4.17136400
H	3.04307900	-4.52977800	5.96823500
C	3.56337100	-3.22606700	-0.05321000
N	3.97275900	-4.31221300	-0.22814100
C	3.09255700	-1.92538500	0.15249000
C	3.56726300	-0.74359700	-0.61431000
O	3.16257300	-0.67054100	-2.04165200
O	1.69807500	-0.65174800	-2.13117200
C	1.27052100	-1.57282700	-3.19158100
C	1.93429100	-1.19057300	-4.51888200
H	3.02149000	-1.27236200	-4.44842200
H	1.67655700	-0.16604900	-4.81293900
H	1.58971500	-1.86179100	-5.31272500
C	1.58578100	-3.01721200	-2.80340800
H	1.12350300	-3.26634700	-1.84527000

H	2.66156000	-3.18033100	-2.71782700
H	1.19556400	-3.69489400	-3.57121300
C	-0.24318300	-1.34452100	-3.24031200
H	-0.70284700	-1.58584600	-2.27919300
H	-0.68258600	-1.99406400	-4.00412100
H	-0.49007600	-0.30873600	-3.49971700
H	3.16050800	0.14427300	-0.12356000
H	3.83330500	4.29878500	-2.81527600
C	5.07600000	-0.59184900	-0.72392400
C	5.71579900	0.44688800	-0.03490300
C	5.85106900	-1.46903900	-1.49626600
C	7.10133900	0.60174100	-0.10092600
H	5.12574000	1.12914500	0.57362700
C	7.23541400	-1.31100600	-1.56737200
H	5.37155600	-2.27839200	-2.03574700
C	7.86552600	-0.27731200	-0.87049400
H	7.58198000	1.40786700	0.44737700
H	7.82367000	-2.00088400	-2.16652800
H	8.94444400	-0.15947800	-0.92530400

TS-*RR*

0 1

N	0.05132400	-1.25729000	0.15021600
C	-0.33251500	-2.66271900	0.17777300
C	-1.62128800	-2.93656000	-0.63352400
H	0.47926100	-3.20638200	-0.30466100
C	-0.45130700	-3.16160600	1.62313600
H	-2.44325300	-2.35082600	-0.21611100
C	-1.98130400	-4.45248000	-0.67025300
N	-1.52027400	-2.44562000	-2.06717400
C	0.69317300	-3.68886300	2.30785000
C	-1.63848800	-3.07973400	2.32430300
C	-1.96289400	-4.96810300	-2.11987900
H	-2.96446300	-4.61663100	-0.21766100
H	-1.26180000	-5.01659900	-0.06886800
C	-0.40695700	-3.12664800	-2.82497500
C	-2.83619200	-2.71360600	-2.76634900
C	1.99137100	-3.78054400	1.73098300
C	0.49870700	-4.13864600	3.65657200
C	-1.71149100	-3.53822500	3.65867700
H	-2.53410400	-2.66341400	1.87560900
C	-0.58295900	-4.66018600	-2.73097200
H	-2.14605200	-6.04683100	-2.12942100
C	-3.04827100	-4.23456500	-2.92701800
H	0.54118400	-2.76869800	-2.42039400
H	-0.48925000	-2.77141600	-3.85271500

H	-3.60922300	-2.23462400	-2.16205400
H	-2.78978300	-2.17542400	-3.71245700
C	3.04316200	-4.30685700	2.45933400
H	2.16777100	-3.39488500	0.73544300
C	1.60347100	-4.68965600	4.36473200
N	-0.69433800	-4.06312400	4.31101000
H	-2.65612100	-3.47080700	4.19685200
H	0.20644100	-5.10184900	-2.11278800
H	-0.49577900	-5.10438900	-3.72767900
H	-2.99304600	-4.52105600	-3.98274400
C	2.84176900	-4.77593700	3.78711500
O	4.31927100	-4.42130400	2.00728400
H	1.42804000	-5.02995100	5.38031000
H	3.69268900	-5.18772800	4.32066000
C	4.61328000	-3.96434100	0.68904200
H	5.68282100	-4.12749000	0.55029200
H	4.05381100	-4.53624100	-0.06168500
H	4.38301800	-2.89933300	0.57275600
N	1.63084800	0.42150700	0.06224000
O	2.05239600	-1.63976800	-0.89845100
H	-0.47437400	-0.64319000	0.77288700
H	0.91487600	0.98802900	0.51645700
C	2.80969300	1.11344500	-0.24632800
C	3.90890100	0.53924300	-0.90178700
C	2.88160300	2.45872900	0.15788600
C	5.05104600	1.30769400	-1.13428900
H	3.86274400	-0.49259700	-1.21596500
C	4.03060900	3.20371200	-0.08584000
H	2.03812000	2.91078500	0.66908300
C	5.13158000	2.63888500	-0.73341400
H	6.02943500	3.21872400	-0.90757200
C	4.07141400	4.65698900	0.30626200
C	6.19697200	0.67982400	-1.88233600
F	3.25450800	4.92741100	1.34744300
F	5.31843200	5.04524700	0.65858100
F	3.68783200	5.46058200	-0.71376400
F	6.35675200	-0.62474500	-1.55925800
F	6.00507000	0.72505500	-3.22263700
F	7.37010400	1.30249800	-1.63341400
H	-1.40730500	-1.36827400	-2.15331800
C	1.30531200	-0.87651000	-0.27037200
S	-1.67169700	1.79327900	1.63161600
O	-0.35450500	2.39041700	1.30046200
O	-1.69284300	0.33105400	1.90119400
C	-2.28692000	2.63722100	3.08623500
C	-3.12867200	1.94567400	3.96154000
C	-1.91640900	3.96531300	3.31200100

C	-3.60953600	2.60706600	5.09042700
H	-3.38416700	0.90979500	3.76741900
C	-2.41074600	4.61384400	4.44364000
H	-1.24831500	4.47226300	2.62479700
C	-3.25445800	3.93783700	5.32810800
H	-4.25779400	2.08217900	5.78568800
H	-2.13224200	5.64575900	4.63415600
H	-3.63388800	4.44844900	6.20851100
C	-3.24756700	3.42706300	0.12776300
N	-3.56739500	4.54172000	-0.02317300
C	-2.86430100	2.09496500	0.35382300
C	-3.28657900	0.93417700	-0.49057000
O	-2.13035400	1.29350900	-1.19685800
O	-1.81141300	0.09644500	-2.73089200
C	-1.06583700	0.85176600	-3.66748100
C	-1.81947000	2.14142300	-4.04708100
H	-1.91223000	2.79926800	-3.17981400
H	-2.82452300	1.89749300	-4.40790000
H	-1.29029200	2.68556900	-4.83869600
C	0.33041400	1.20306500	-3.12399500
H	0.89833200	0.29311100	-2.89706600
H	0.23990600	1.78963400	-2.20565300
H	0.90798800	1.78797300	-3.85060700
C	-0.91942300	-0.00851600	-4.94508500
H	-0.31261500	-0.90088700	-4.75734500
H	-0.42321600	0.56143200	-5.73993400
H	-1.90397900	-0.31995300	-5.31247400
H	-4.04486100	-4.51298400	-2.56928800
H	-3.17548500	-0.00766000	0.05855500
C	-4.62458000	0.97899900	-1.18459900
C	-4.92619500	1.92028200	-2.17553400
C	-5.60465900	0.05405100	-0.80302400
C	-6.18824700	1.93499300	-2.76913100
H	-4.17199200	2.63379300	-2.48424200
C	-6.86898300	0.06938600	-1.39442600
H	-5.37914700	-0.67839700	-0.02985400
C	-7.16280500	1.01187300	-2.38116200
H	-6.41136100	2.67070700	-3.53700700
H	-7.62018600	-0.65297400	-1.08617500
H	-8.14482900	1.02715400	-2.84618300

TS-R_{rot}

0 1

N	1.79171300	-0.81486300	0.35820700
C	2.71259300	-1.68888600	-0.35241900
C	1.98757900	-2.88426600	-1.02665100
H	3.14286700	-1.07501400	-1.14287700
C	3.85599200	-2.19417200	0.53939900
H	1.67310200	-3.60075600	-0.26747600
C	2.84781200	-3.57102000	-2.12089200
N	0.67246100	-2.45692700	-1.65877500
C	5.13747300	-1.55188200	0.53657700
C	3.67883800	-3.27095700	1.38750700
C	2.04903800	-3.64574700	-3.43702600
H	3.13715800	-4.57345900	-1.79185200
H	3.77392000	-3.00750900	-2.27797800
C	0.86708500	-1.50372700	-2.81099400
C	-0.09074000	-3.67675000	-2.12828100
C	5.44003700	-0.38221700	-0.21642000
C	6.16192600	-2.13428500	1.35718600
C	4.75093600	-3.74075900	2.17817500
H	2.72270300	-3.77867200	1.46995100
C	1.70096400	-2.21757700	-3.90432700
H	2.64808500	-4.14976100	-4.20104800
C	0.74591300	-4.42754300	-3.18916100
H	1.35765700	-0.61124200	-2.42107700
H	-0.13557000	-1.22323400	-3.13876000
H	-0.30390300	-4.26909200	-1.23757200
H	-1.03862400	-3.30394100	-2.52093000
C	6.71018800	0.16351300	-0.17607800
H	4.65340900	0.12170500	-0.76183100
C	7.46149000	-1.55391200	1.35184500
N	5.96016000	-3.21742200	2.15830000
H	4.59630500	-4.59295500	2.83858700
H	2.61784600	-1.65361400	-4.10364000
H	1.13616100	-2.25717000	-4.84124600
H	0.17589900	-4.52099100	-4.11859900
C	7.73520000	-0.44050900	0.60375100
O	7.09241400	1.28693400	-0.83844100
H	8.22103100	-2.02069100	1.97080900
H	8.72009100	0.01587100	0.59964800
C	6.11837700	1.97149600	-1.62240500
H	6.62790100	2.84398400	-2.03351000
H	5.75415300	1.33886800	-2.44166000
H	5.26711200	2.29252600	-1.01149000
N	0.71395200	1.17441000	0.78162900
O	2.49718100	1.09254800	-0.69711900
H	1.10628600	-1.24594500	0.97606200

H	-0.04103800	0.58927400	1.15358400
C	0.44864700	2.54966900	0.79629300
C	1.35298700	3.52324300	0.34496300
C	-0.78141200	2.95974400	1.34001000
C	1.01697100	4.87392200	0.44137100
H	2.30052600	3.21998900	-0.07466500
C	-1.09254600	4.31364400	1.42833700
H	-1.48581600	2.21301200	1.69142200
C	-0.19946700	5.28849000	0.98114700
H	-0.44114000	6.34093300	1.06275900
C	-2.43952300	4.72714100	1.95890500
C	1.97470300	5.89951100	-0.10451900
F	-2.39859200	5.94627400	2.54197900
F	-3.36429600	4.79822200	0.97113300
F	-2.91429800	3.85558800	2.87550400
F	1.77152400	6.11775600	-1.42707300
F	1.84351000	7.09503400	0.51192100
F	3.26373300	5.51457400	0.03041100
H	0.09999200	-2.03771400	-0.89861700
C	1.72556300	0.53405100	0.09113100
S	-1.86819300	-1.73765500	1.26587900
O	-1.74325600	-0.25502700	1.43597900
O	-0.53796400	-2.34471200	0.82446600
C	-2.17438300	-2.45863100	2.88619600
C	-1.48241400	-3.60110400	3.28731000
C	-3.07814400	-1.81575500	3.73581400
C	-1.71034800	-4.11592000	4.56438800
H	-0.78050100	-4.07174500	2.60873500
C	-3.29612800	-2.33835000	5.00938500
H	-3.59662300	-0.92241000	3.40451300
C	-2.61497000	-3.48712000	5.42195700
H	-1.18174800	-5.00812600	4.88730000
H	-3.99673800	-1.84875000	5.67932400
H	-2.78993000	-3.89189200	6.41478700
C	-3.81518500	-3.40353900	0.61780200
N	-4.35614700	-4.42621100	0.81720600
C	-3.21635600	-2.16968700	0.32798400
C	-3.49406200	-1.59104100	-1.06294900
O	-2.52474700	-0.54521600	-1.30736700
O	-2.45414300	-0.39918900	-2.76901700
C	-2.46142400	1.01363200	-3.13405000
C	-3.77950000	1.68592900	-2.74476400
H	-3.90191300	1.70510300	-1.65995500
H	-4.63224700	1.15742400	-3.17951100
H	-3.78537800	2.71925000	-3.10937400
C	-1.26855800	1.73465500	-2.50101800
H	-0.31918000	1.28846300	-2.81439100

H	-1.33120600	1.69393800	-1.41148600
H	-1.26219200	2.78752700	-2.80279600
C	-2.32127200	0.92750900	-4.65991100
H	-1.38688800	0.43292500	-4.94625700
H	-2.31611600	1.93712500	-5.08305100
H	-3.15876700	0.37443300	-5.09697900
H	-3.31200600	-2.36325400	-1.82407000
H	0.97254300	-5.44144000	-2.84458300
C	-4.92890400	-1.11092300	-1.21781900
C	-5.68952100	-1.51697000	-2.31774100
C	-5.50236200	-0.24811300	-0.27503400
C	-7.00218800	-1.06578100	-2.48239000
H	-5.25316400	-2.19338900	-3.04937300
C	-6.80674700	0.21267400	-0.44059300
H	-4.91711100	0.05226500	0.58950900
C	-7.56125200	-0.19619400	-1.54556000
H	-7.58544400	-1.39548700	-3.33828900
H	-7.24075900	0.88475800	0.29515500
H	-8.58214200	0.15583900	-1.66873100

II-*R′*

0 1

N	-1.66940400	-0.37844700	-0.20922900
C	-2.93059800	-0.86475600	0.33779500
C	-2.70527700	-1.93779500	1.43083200
H	-3.40317600	-0.00456900	0.81174200
C	-3.85693200	-1.37778300	-0.76762700
H	-2.24269500	-2.82559200	0.99762900
C	-4.00577600	-2.29776900	2.20047100
N	-1.67322400	-1.47729800	2.45704800
C	-4.78553900	-0.49149500	-1.40595400
C	-3.80092300	-2.68513200	-1.21026400
C	-3.85333900	-1.95133000	3.69386600
H	-4.22821700	-3.36205600	2.07696100
H	-4.85061200	-1.74534000	1.77606100
C	-2.09408500	-0.22538500	3.19711300
C	-1.43919200	-2.59738400	3.45808500
C	-4.89917900	0.89422900	-1.10017500
C	-5.63373500	-1.05232100	-2.41746500
C	-4.67408900	-3.12425800	-2.23160600
H	-3.09063200	-3.39599500	-0.80147300
C	-3.48647500	-0.46082900	3.82482600
H	-4.79414300	-2.15160300	4.21508700
C	-2.71984400	-2.79929100	4.29848200
H	-2.07412700	0.60663300	2.49226400
H	-1.31814500	-0.06056300	3.94571200

H	-1.17608500	-3.47846500	2.87179800
H	-0.57170400	-2.29499500	4.04465500
C	-5.82654700	1.67998100	-1.76007800
H	-4.22389800	1.34519000	-0.38472300
C	-6.58780100	-0.21305400	-3.05855600
N	-5.57344900	-2.35555900	-2.81180300
H	-4.62285200	-4.15851500	-2.56866300
H	-4.23940300	0.15696400	3.32366600
H	-3.47197900	-0.16007600	4.87721300
H	-2.54657100	-2.50785100	5.33999300
C	-6.68783600	1.11521800	-2.74097500
O	-5.99994000	3.01309100	-1.55514800
H	-7.22413500	-0.66362300	-3.81363600
H	-7.40624500	1.76605900	-3.22947500
C	-5.16991500	3.66076200	-0.59501500
H	-5.45681200	4.71305200	-0.61562500
H	-5.33599000	3.25445000	0.41061700
H	-4.10853000	3.56125100	-0.84987500
N	-0.14399600	1.24933500	-0.78115700
O	-1.98277900	1.77495800	0.52123400
H	-1.21042000	-0.98934100	-0.88918400
H	0.39966700	0.45739600	-1.13617300
C	0.55336200	2.46315900	-0.77202000
C	0.04699400	3.66385600	-0.24968800
C	1.84218300	2.45450400	-1.33475100
C	0.83191600	4.81712300	-0.29017100
H	-0.93785200	3.67952500	0.19289800
C	2.60921300	3.61576700	-1.35441100
H	2.23751100	1.53274300	-1.74900300
C	2.11564200	4.81282600	-0.83422800
H	2.71478600	5.71448700	-0.85239900
C	4.01802300	3.54640400	-1.87942400
C	0.24559300	6.10888600	0.21546800
F	4.13139700	2.69448400	-2.92007400
F	4.47129600	4.75105100	-2.28554900
F	4.88196900	3.10756400	-0.92389700
F	-0.59801900	5.91069400	1.25365000
F	1.20059200	6.97069000	0.63118700
F	-0.46461800	6.74594900	-0.74616800
H	-0.77399300	-1.29258700	1.97643500
C	-1.31637900	0.95156600	-0.12155600
S	1.10916900	-2.26590500	-1.36518300
O	1.67371900	-0.89047900	-1.48717100
O	-0.28697300	-2.45584100	-1.85410400
C	2.13250600	-3.31404200	-2.41463700
C	1.57604500	-4.47312700	-2.96211500
C	3.47084700	-2.97633900	-2.63001900

C	2.37872400	-5.30574800	-3.74206900
H	0.53022900	-4.70492000	-2.79281000
C	4.26111000	-3.81771500	-3.41390200
H	3.87990200	-2.06791400	-2.20189400
C	3.71872000	-4.98022500	-3.96708000
H	1.95416900	-6.20540000	-4.17839200
H	5.30109100	-3.56079600	-3.59403800
H	4.33906900	-5.63062300	-4.57750900
C	0.30780100	-3.81317100	0.61070600
N	-0.43634400	-4.66903100	0.92564400
C	1.17999000	-2.77276100	0.28153400
C	2.04905000	-2.17164300	1.34510500
O	1.33676800	-0.99290800	1.91254200
O	1.42189700	-1.03835000	3.38643200
C	2.09458300	0.11866700	3.97730800
C	3.61693500	-0.03799300	3.91906900
H	3.99161900	0.03465800	2.89737900
H	3.91681200	-1.00752700	4.33052200
H	4.09467900	0.74944800	4.51327600
C	1.63163900	1.42440000	3.32886600
H	0.54289300	1.52937400	3.37742600
H	1.93563700	1.47441400	2.28117700
H	2.07861300	2.27434700	3.85558900
C	1.61390800	0.02179100	5.43403400
H	0.53070000	0.16422300	5.50730800
H	2.10195900	0.79637800	6.03424800
H	1.86852900	-0.95315900	5.86287400
H	2.08546600	-2.88583300	2.17233800
H	-2.99417900	-3.85868600	4.29951300
C	3.47838500	-1.83898400	0.94033200
C	4.45069900	-2.84268700	1.04087900
C	3.85953200	-0.58033900	0.46026300
C	5.77123300	-2.59935600	0.66007800
H	4.17040300	-3.82510900	1.41462900
C	5.17956700	-0.33270200	0.07800900
H	3.11904800	0.20592000	0.38404000
C	6.14006200	-1.34247200	0.17519000
H	6.51122700	-3.39055200	0.74720800
H	5.44855500	0.65125400	-0.29598800
H	7.16813600	-1.14976600	-0.11974800

TS-*RS*

0 1

N	-1.57839400	-0.28270600	-0.14249400
C	-2.90662500	-0.70603200	0.28138400
C	-2.84195700	-1.76679600	1.40543100
H	-3.39619500	0.18144600	0.68195200
C	-3.70622300	-1.19701300	-0.92862600
H	-2.24027900	-2.61058200	1.06596700
C	-4.25042800	-2.24554200	1.87026900
N	-2.10213000	-1.26810600	2.63869400
C	-4.47176400	-0.27312700	-1.71277800
C	-3.67131500	-2.51688500	-1.33458000
C	-4.43636000	-2.00049100	3.37822300
H	-4.37380100	-3.30748200	1.63645600
H	-5.02691800	-1.70883900	1.31573700
C	-2.79522200	-0.10706700	3.31063100
C	-1.97657000	-2.41919600	3.61725600
C	-4.54117800	1.12394400	-1.44847100
C	-5.19393800	-0.80667500	-2.83094700
C	-4.41104500	-2.92867700	-2.46657600
H	-3.08149800	-3.25945700	-0.80711300
C	-4.24976700	-0.49723100	3.65249400
H	-5.44022200	-2.31517100	3.67956700
C	-3.37286500	-2.79240700	4.16034100
H	-2.71179800	0.75297700	2.64434900
H	-2.20046300	0.10363400	4.19883200
H	-1.50701400	-3.23541500	3.06560100
H	-1.27136100	-2.08000400	4.37503500
C	-5.30725100	1.94840100	-2.25238100
H	-3.95717900	1.55005000	-0.64308700
C	-5.98451800	0.07337300	-3.62231300
N	-5.16059200	-2.12128800	-3.18965800
H	-4.37964000	-3.97325600	-2.77316500
H	-4.95663000	0.08340700	3.04978800
H	-4.45865100	-0.26825700	4.70257800
H	-3.44358300	-2.56312300	5.22921300
C	-6.04504600	1.41286800	-3.34429900
O	-5.42536400	3.29366500	-2.09520500
H	-6.52835000	-0.35589600	-4.45783500
H	-6.63885000	2.09402900	-3.94579200
C	-4.71234700	3.91285700	-1.02785900
H	-4.92678600	4.97951700	-1.10647800
H	-5.05426800	3.54013900	-0.05422900
H	-3.63276400	3.74494600	-1.11603900
N	0.07638300	1.24435800	-0.60433600
O	-1.80725700	1.87836700	0.58733800
H	-1.10125100	-0.90716100	-0.78866200

H	0.59390800	0.42410300	-0.91938600
C	0.81059100	2.43475100	-0.62763300
C	0.31751400	3.67398000	-0.18947000
C	2.11902300	2.36325700	-1.13598200
C	1.13527000	4.80236700	-0.25724600
H	-0.68243500	3.73741500	0.21345500
C	2.91982500	3.50088400	-1.18216100
H	2.50488800	1.41278600	-1.49054000
C	2.43987600	4.73579500	-0.74524000
H	3.06512800	5.61886600	-0.78272500
C	4.34555100	3.36556200	-1.64403500
C	0.56531200	6.13439900	0.15407300
F	4.47150900	2.46778700	-2.64522600
F	4.85498800	4.53678000	-2.07968400
F	5.15601000	2.93855400	-0.63825400
F	-0.32876000	6.01529800	1.16057600
F	1.52585900	6.99157200	0.56597900
F	-0.08275600	6.73746200	-0.87223700
H	-1.10684500	-0.96160400	2.47118600
C	-1.15087200	1.01722800	-0.01191400
S	1.19745700	-2.39382700	-1.37140800
O	1.84997800	-1.06585200	-1.37564800
O	-0.17598400	-2.48645600	-1.91783800
C	2.24319600	-3.51466300	-2.29690500
C	1.65124500	-4.57777900	-2.98393200
C	3.62654300	-3.31140300	-2.29656800
C	2.47184600	-5.45795600	-3.68912900
H	0.57355100	-4.69846500	-2.97710100
C	4.43105300	-4.20158600	-3.00673000
H	4.05820300	-2.47059400	-1.76455900
C	3.85645100	-5.27156500	-3.69854300
H	2.02771100	-6.28516000	-4.23458600
H	5.50686800	-4.05518400	-3.02278300
H	4.49001600	-5.95966600	-4.25080700
C	0.08259500	-3.95659800	0.50385600
N	-0.77209300	-4.72614700	0.72364400
C	1.10733900	-3.01656900	0.28818900
C	1.89818600	-2.53534400	1.47004800
O	0.86065400	-1.58951000	1.52134500
O	0.46024500	-0.70680500	3.29312500
C	1.21233900	0.33239900	3.86737000
C	2.59118300	-0.18195700	4.32693700
H	3.19109500	-0.50569600	3.47333300
H	2.46235800	-1.03885600	4.99871100
H	3.15317400	0.59412200	4.86146800
C	1.36168400	1.53105500	2.91457200
H	0.37585600	1.88427700	2.59248900

H	1.92560000	1.24387700	2.02362700
H	1.88819900	2.36588900	3.39369500
C	0.43158600	0.79626100	5.12666500
H	-0.52304100	1.25544800	4.84898600
H	1.01031600	1.54195000	5.68578300
H	0.23696200	-0.05402300	5.78996500
H	1.83213800	-3.26460000	2.28756000
H	-3.53999900	-3.86835000	4.04844900
C	3.33861800	-2.10636800	1.28765800
C	4.34245200	-3.05082800	1.54114700
C	3.70474700	-0.82127600	0.87665500
C	5.68708100	-2.72191500	1.36696200
H	4.07027600	-4.04966100	1.87613900
C	5.04931400	-0.48698300	0.70611700
H	2.93178200	-0.08915000	0.68933100
C	6.04372900	-1.43810500	0.94624900
H	6.45456300	-3.46485300	1.56659100
H	5.31208000	0.51609200	0.38171900
H	7.09065400	-1.17844700	0.81432000

Epoxide *RR*

0 1

N	0.33312300	1.40468600	0.28375300
C	-0.21621300	2.73288800	0.52737500
C	-1.32550900	2.69593800	1.60864300
H	0.61306200	3.32841700	0.91118700
C	-0.69290300	3.36690500	-0.78816300
H	-2.17030900	2.12304800	1.21530300
C	-1.79188200	4.13948400	1.98542100
N	-0.91073800	1.93896900	2.82419700
C	0.21725200	4.08202200	-1.63756600
C	-1.99867700	3.24072700	-1.22013700
C	-1.74724300	4.29335700	3.51629800
H	-2.80388800	4.32627200	1.60942300
H	-1.14159700	4.89141500	1.52065500
C	0.15721000	2.65550900	3.56509900
C	-2.09832200	1.82049800	3.70792100
C	1.60178400	4.24852700	-1.35142500
C	-0.31528000	4.65250700	-2.84169600
C	-2.40952300	3.82239000	-2.44123800
H	-2.74310600	2.70397300	-0.64434400
C	-0.29552100	4.08517500	3.98822200
H	-2.09746700	5.29179000	3.80097900
C	-2.64107800	3.21233400	4.14985100
H	1.05643900	2.68018300	2.94420000
H	0.40124700	2.04787600	4.44245400

H	-2.85778300	1.24812700	3.16582400
H	-1.79805600	1.22711100	4.57842600
C	2.41138800	4.96596500	-2.21323900
H	2.02941400	3.76514600	-0.48287000
C	0.54917700	5.39554100	-3.69325900
N	-1.61597000	4.51808100	-3.22981400
H	-3.44317500	3.70362100	-2.76332200
H	0.35478800	4.84773900	3.54241700
H	-0.23064000	4.20718700	5.07625700
H	-2.63128500	3.30634400	5.24252300
C	1.87550200	5.55499200	-3.39090300
O	3.74612800	5.16889600	-2.03695200
H	0.11853100	5.82400900	-4.59290400
H	2.54460400	6.11555400	-4.03653500
C	4.36418400	4.60401800	-0.88379800
H	5.42054600	4.86835400	-0.95358900
H	3.94056900	5.02222400	0.03787800
H	4.25390600	3.51415400	-0.86253400
N	1.83904600	-0.30899400	0.43718700
O	2.53187000	1.89185400	0.72705600
H	-0.31256100	0.69364200	-0.03821400
H	1.04284900	-0.86545200	0.75055200
C	3.04212000	-0.98477400	0.23133300
C	4.29730300	-0.35452800	0.18145400
C	2.97497600	-2.37337500	0.02724300
C	5.43974800	-1.11155000	-0.07545200
H	4.36133000	0.71101600	0.34807300
C	4.12986200	-3.11026200	-0.22291000
H	2.01113000	-2.87118100	0.03810200
C	5.37787600	-2.49069700	-0.28025300
H	6.27392300	-3.06401100	-0.48153500
C	3.99545000	-4.58072800	-0.51343700
C	6.78143300	-0.42716300	-0.06024200
F	3.58973400	-4.80416000	-1.78996800
F	5.16012600	-5.24344100	-0.35170400
F	3.07804500	-5.17058800	0.28738600
F	7.31182100	-0.39604600	1.18641500
F	7.67968500	-1.05850400	-0.84943200
F	6.70326600	0.85421900	-0.48205100
H	-0.55951900	-0.06308500	2.44603700
C	1.63811100	1.06951600	0.50230200
S	-4.50862100	-0.13989800	-0.45773700
O	-4.78306100	1.06080500	-1.26085900
O	-4.10609900	-0.02932200	0.95378100
C	-5.88750400	-1.26859100	-0.59403900
C	-6.10560900	-2.19793800	0.42802200
C	-6.71610200	-1.19238400	-1.71776800

C	-7.18100900	-3.07708700	0.31183400
H	-5.45925900	-2.21456300	1.29892300
C	-7.78586500	-2.08125600	-1.81794400
H	-6.53299000	-0.44786100	-2.48452000
C	-8.01465800	-3.02003900	-0.80876300
H	-7.37150000	-3.80118700	1.09803500
H	-8.44127400	-2.03644700	-2.68207900
H	-8.85086600	-3.70795900	-0.89300200
C	-3.34316200	-1.31411200	-2.69555300
N	-3.58345900	-1.53999100	-3.80874700
C	-3.10450500	-1.03796300	-1.29661900
C	-2.16503700	-1.84155200	-0.45726400
O	-1.84064000	-0.51832200	-0.93590500
O	-0.41906800	-1.00602400	2.17754400
C	-0.25609100	-1.83266600	3.35412000
C	-1.62991000	-2.06103800	4.00352700
H	-2.31059200	-2.54576300	3.29464700
H	-2.08199600	-1.11206200	4.31040100
H	-1.54640000	-2.69961600	4.89068300
C	0.32616100	-3.16133300	2.86439500
H	1.32769100	-3.01949200	2.44473200
H	-0.31075500	-3.60251000	2.09085700
H	0.40533000	-3.87453400	3.69190500
C	0.71237300	-1.16268500	4.33890000
H	1.66597200	-0.94230900	3.84761600
H	0.90935000	-1.81499300	5.19708800
H	0.29909000	-0.22345200	4.72278400
H	-2.39152900	-1.84883900	0.60651200
H	-3.68018700	3.34503300	3.82573700
C	-1.36865200	-2.98355800	-0.98382700
C	-1.57090000	-4.25654000	-0.43292900
C	-0.40168100	-2.80355600	-1.98085000
C	-0.81015000	-5.33889100	-0.87348200
H	-2.31863700	-4.39801900	0.34387900
C	0.36690400	-3.88711100	-2.40821300
H	-0.23656700	-1.81583800	-2.39902500
C	0.16692200	-5.15362000	-1.85475400
H	-0.96975500	-6.32198400	-0.44027000
H	1.13301500	-3.73868600	-3.16295600
H	0.77920500	-5.98947200	-2.17921300

Epoxide *RS*

0 1

N	-0.16781300	1.32482100	0.11746800
C	-0.90480300	2.54396100	-0.20825900
C	-1.80454500	3.05276400	0.94592200
H	-0.14127800	3.30260800	-0.37783700
C	-1.68152600	2.34181200	-1.51709100
H	-2.60759800	2.32651800	1.10886400
C	-2.41395300	4.44942400	0.58579700
N	-1.09334200	3.11195800	2.24839500
C	-1.04546300	2.54900300	-2.78680500
C	-2.99195300	1.90469000	-1.52684800
C	-2.10375300	5.43601800	1.72700400
H	-3.49632800	4.37130600	0.43043600
H	-1.99092300	4.82812400	-0.35327400
C	-0.01620100	4.13072200	2.23675200
C	-2.07715400	3.48521900	3.29489800
C	0.31022300	2.95753300	-2.93256600
C	-1.83035800	2.31779800	-3.96530600
C	-3.66838700	1.69938000	-2.75038600
H	-3.52873500	1.70446800	-0.60615300
C	-0.57421200	5.54376500	1.88596000
H	-2.52991000	6.41853800	1.49481300
C	-2.70536100	4.88553600	3.03228200
H	0.76019200	3.81201700	1.53767600
H	0.43485200	4.12393800	3.23494700
H	-2.83626000	2.69783600	3.33283800
H	-1.55126000	3.46288800	4.25425700
C	0.85664500	3.13369300	-4.19071500
H	0.92689700	3.09429300	-2.05434900
C	-1.23470200	2.52497300	-5.24111900
N	-3.12824600	1.89959000	-3.93569400
H	-4.70414600	1.36031400	-2.73624600
H	-0.12872800	5.91977900	0.95642500
H	-0.32716100	6.26720100	2.67243200
H	-2.49572600	5.57020100	3.86307600
C	0.07088500	2.92251300	-5.35709000
O	2.14275400	3.50414500	-4.43252000
H	-1.85213700	2.35118500	-6.11690200
H	0.53576200	3.07883700	-6.32562200
C	2.99350600	3.74949700	-3.31437500
H	3.96897200	4.00028400	-3.73364700
H	2.62392000	4.59013400	-2.71414900
H	3.08071600	2.86582600	-2.67241100
N	1.72106600	-0.00384900	0.18945700
O	1.90419300	2.28542700	-0.05987000
H	-0.70822500	0.46983900	0.21036500
H	1.06867700	-0.78269100	0.17862600
C	3.07069200	-0.37476800	0.22370500
C	4.13464600	0.53961400	0.28394200
C	3.35796400	-1.75156800	0.20941000

C	5.44756200	0.06812500	0.32533600
H	3.92584400	1.59891400	0.28913900
C	4.67557000	-2.19660500	0.25173800
H	2.54568300	-2.46933700	0.16691400
C	5.73894400	-1.29428500	0.31008400
H	6.76297000	-1.64492800	0.34271300
C	4.96031300	-3.67199900	0.16259400
C	6.56756100	1.06639500	0.45581400
F	3.95645100	-4.41648300	0.67586900
F	5.12554000	-4.07170700	-1.12209200
F	6.09185200	-4.00728500	0.82185500
F	6.30518900	2.21266100	-0.21121600
F	6.78333600	1.40976600	1.74868100
F	7.73698900	0.58209500	-0.01951400
H	-0.91772500	1.22546400	2.93895200
C	1.19857300	1.27786000	0.07277900
S	-1.37480200	-2.46636100	-0.32431400
O	0.08053900	-2.64279100	-0.14291100
O	-1.96195700	-1.13372200	-0.12415200
C	-1.85699800	-3.09745000	-1.92042000
C	-3.02592200	-2.60377500	-2.50956100
C	-1.05191300	-4.05601200	-2.54435200
C	-3.39576200	-3.09510900	-3.76024800
H	-3.61403400	-1.84233300	-2.00926800
C	-1.44092900	-4.53599600	-3.79401300
H	-0.14111200	-4.40338300	-2.06964700
C	-2.60794300	-4.05864600	-4.39648600
H	-4.29279300	-2.71794600	-4.24116100
H	-0.82860900	-5.27727800	-4.29761800
H	-2.90168300	-4.43471600	-5.37205100
C	-1.67490600	-4.98401400	0.72843000
N	-1.30881000	-6.07506900	0.57411700
C	-2.16032900	-3.62697700	0.89718600
C	-3.48038100	-3.38171300	1.57210800
O	-2.21888600	-3.13828000	2.21718200
O	-1.27774100	0.39821800	3.33564400
C	-0.38688800	-0.08972400	4.35278800
C	-1.18129700	-1.14853900	5.12428600
H	-1.49249900	-1.95491600	4.45268500
H	-2.07880800	-0.70188300	5.56669300
H	-0.57656300	-1.58201000	5.92868600
C	0.85245900	-0.71936200	3.69706700
H	1.41187400	0.02999000	3.12554300
H	0.55145200	-1.51825100	3.01072000
H	1.52930800	-1.14631200	4.44696300
C	0.02623500	1.05585000	5.29043700
H	0.57045600	1.83337600	4.74010600
H	0.68199500	0.69409700	6.09091700
H	-0.85732800	1.51224300	5.75028000
C	-4.42441000	-2.24975700	1.34832600
C	-5.62417400	-2.51642200	0.67384100

C	-4.18122400	-0.97423800	1.87255200
C	-6.56924000	-1.50591400	0.50011000
H	-5.82114800	-3.51467100	0.28917700
C	-5.14120900	0.02581700	1.70991500
H	-3.25251300	-0.76145500	2.39343000
C	-6.32929800	-0.23225200	1.02149800
H	-7.49473100	-1.71663800	-0.02806700
H	-4.96031400	1.01114200	2.13088900
H	-7.07092000	0.55257900	0.90036000
H	-3.95180000	-4.30755700	1.90720500
H	-3.79668900	4.81553900	2.94431800
